# Design, synthesis and biological evaluation of donepezil-safinamide hybrids as dual AChE and MAO-B inhibitor for Alzheimer’s disease treatment

**DOI:** 10.1080/14756366.2026.2622769

**Published:** 2026-02-06

**Authors:** Wei Li, Yan Guo, Xiaoli Wang, Chunyan Yang, Jiang Zhu, Zhongcheng Cao

**Affiliations:** ^a^ School of Pharmacy, North Sichuan Medical College, Nanchong, China; ^b^ Institute of Basic Medicine and Forensic Medicine, North Sichuan Medical College, Nanchong, China; ^c^ Department of pharmacy, Meishan People’s Hospital, Meishan, China; ^d^ Sichuan Key Laboratory of Medical Imaging, North Sichuan Medical College, Nanchong, China

**Keywords:** Alzheimer’s disease, AChE/MAO-B dual inhibitor, donepezil-safinamide hybrids, neuroprotection

## Abstract

Alzheimer’s disease (AD) still lacks therapies that definitively halt its progression. Dual AChE/MAO-B inhibitors offer a promising strategy to address both symptoms and pathology. Here, we designed and synthesised a series of donepezil-safinamide hybrids. The optimised compound **28c** was identified as a potent inhibitor of AChE (IC_50_ = 1.70 μM) and MAO-B (IC_50_ = 0.18 μM). Mechanistic studies indicated that **28c** acts as a reversible mixed-type inhibitor of AChE and a competitive reversible inhibitor of MAO-B. Molecular docking and molecular dynamic simulations revealed that **28c** could strongly and stably bind to MAO-B and AChE mainly through van der Waals interactions. Moreover, compound **28c** demonstrated effective blood-brain barrier penetration, exhibited suitable stability in mouse plasma and brain homogenate, and showed a favourable safety profile both *in vitro* and *in vivo*. Furthermore, **28c** could attenuate AD-related symptoms and exert hippocampal neuroprotection effect *in vivo*, highlighting its promise as an anti-AD candidate.

## Graphical Abstract

**Figure:**
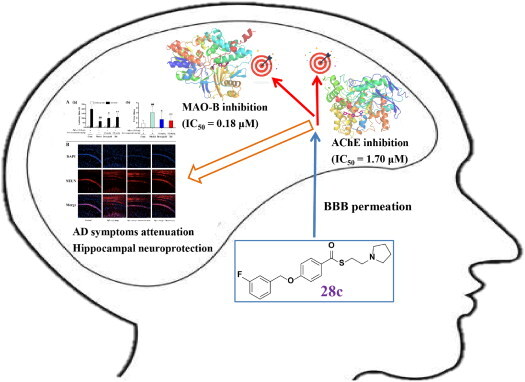

## Introduction

Alzheimer’s disease (AD) is the most common neurodegenerative disorder worldwide, characterised primarily by the accumulation of amyloid-*β* (A*β*) plaques and neurofibrillary tau tangle^1^. Currently, AD affects over 50 million people globally, and this number is increasing rapidly due to rising life expectancy^2^. The disease can progress over decades, placing a growing economic burden on patients, caregivers, and society as a whole^3^. Unfortunately, no currently available therapeutics can definitively halt or reverse AD progression, underscoring the urgent need to develop novel anti-AD agents^4^.

The pathogenesis of AD is complex, various factors are involved in this process, such as cholinergic neurotransmission dysregulation, increase in oxidative stress, abnormal Α*β* deposits, *tau* hyperphosphorylation, neuroinflammation et al^5^. Among them, cholinergic dysregulation was directly linked to the cognitive decline in AD patients, because acetylcholine (ACh), the cholinergic neurotransmitter, mediates memory formation and retrieval in the brain^6^. Physiologically, ACh is synthesised in the presynaptic cholinergic neurons through choline acetylation catalysed by choline acetyltransferase^7^. Upon release into the synaptic cleft, ACh binds to muscarinic (M) or nicotinic (N) receptors on the postsynaptic membrane to execute its neuromodulatory functions^8^. Subsequently, ACh is hydrolysed by the catalytic effects of acetylcholinesterase (AChE) and butyrylcholinesterase (BuChE) ^9^. However, the cholinergic neurons undergo significant degeneration and death in the brain of AD patients, leading to a pronounced reduction in cerebral ACh levels that underlies core clinical AD symptoms^10^. Many studies have revealed that AChE was primarily localised within the central nervous system and more potent than BuChE, which exhibited systemic distribution throughout peripheral tissues^11^. Although recent study showed that AD neuropathology involved a marked shift from AChE to BuChE predominance, current clinical practice continues to rely on AChE inhibitors as cornerstone symptomatic treatments^12^.

Monoamine oxidase-B (MAO-B), a subtype of monoamine oxidase, serves as an additional biomarker for AD^13^. Predominantly expressed in glial cells, platelets, and hepatocytes, MAO-B exhibits significant upregulation surrounding A*β* deposits within the hippocampus and cerebral cortices of AD patients^14^. Critically, the oxidative deamination of endogenous and dietary amines (*e*.*g*., dopamine, *β*-phenylethylamine, and benzylamine) catalysed by overexpressed MAO-B generates excessive reactive oxygen species (ROS) ^15^. This process exacerbates oxidative stress and neuroinflammation, ultimately driving neuronal death and cognitive decline^16–20^. Recent studies further demonstrated that elevated MAO-A and MAO-B activity directly stimulated *β*-secretase and *γ*-secretase, promoting both amyloidogenic and non-amyloidogenic cleavage of amyloid precursor protein (APP), thereby accelerating pathological A*β* deposition^21^. Consequently, MAO-B represents a promising therapeutic target for AD, with its inhibition potentially delaying disease progression^22^.

Three categories of anti-AD drugs are currently approved by the US FDA: acetylcholinesterase inhibitors (donepezil, rivastigmine, and galantamine), the *N*-methyl-*D*-aspartate (NMDA) receptor antagonist memantine, and Α*β* monoclonal antibodies (lecanemab and donanemab) ^23^. Nevertheless, all these drugs demonstrate limited therapeutic efficacy against AD progression, with some posing significant safety risks. Given the multifactorial neurodegenerative pathology of AD, the “multi-target-directed ligands” (MTDLs) strategy has emerged as a promising approach for novel drug design^24^. In particular, dual AChE/MAO-B inhibitors developed *via* MTDLs are anticipated to simultaneously alleviate symptoms and modify disease progression^25^.

Donepezil, a potent and selective AChE inhibitor, is one of approved drugs for the treatment of AD^26^. The co-crystal structure of human AChE with donepezil (PDB ID: *4ey7*) shows that the alkaline tertiary amine moiety of donepezil occupies the catalytic active site (CAS) of AChE, where it forms van der Waals contacts with the residues of the catalytic triad (Ser203, Glu202, and His447) and inhibit AChE directly. In addition, the indanone moiety of donepezil binds to the peripheral anionic site (PAS) of AChE and exhibits two π–π stacking interaction with Trp286 and Tyr431. This dual binding mode results in a mixed-type inhibition of AChE by donepezil^27^. Moreover, safinamide is a newly approved MAO-B inhibitor, that possesses an aryl benzyl ether fragment and a secondary amine side chain^28^. In the MAO-B/safinamide co-crystal structure (PDB ID: *2v5z*), the aryl benzyl ether moiety occupies both the entrance and substrate cavities of MAO-B *via* van der Waals interaction and hydrophobic interactions, thereby blocking substrate access to the FAD cofactor. Additionally, the secondary amine side chain lies in close proximity to FAD and occupies a hydrophilic region of the enzyme, forming a hydrogen bond with Gln206^29^.

**Figure 1.**
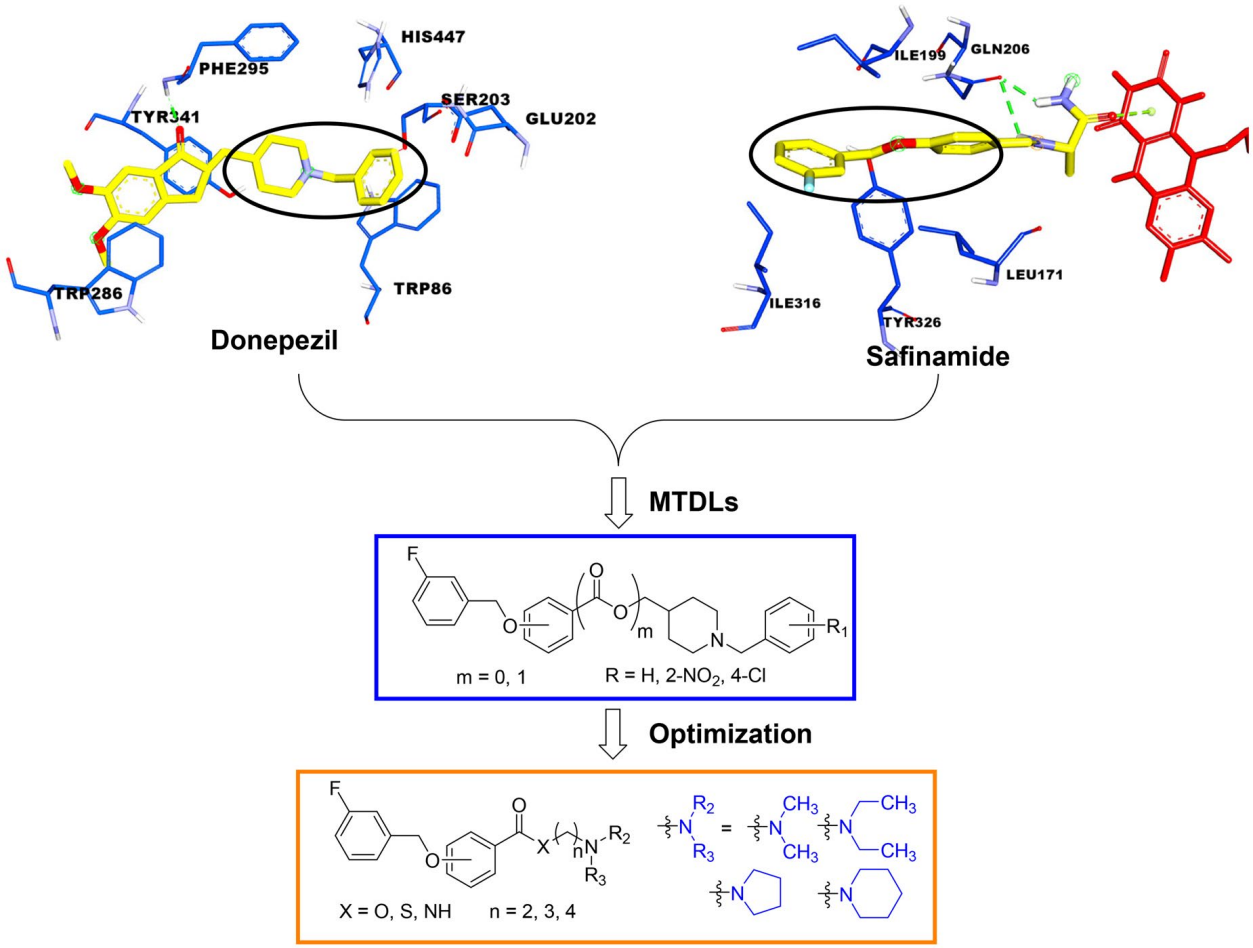
Design strategy of donepezil-safinamide hybrids.

The structural modification of donepezil and safinamide demonstrates significant versatility. Numerous studies have revealed that the indanone moiety of donepezil could be replaced by a simple pyridine or a substituted benzene ring while preserving binding affinity for the PAS of AChE^30–32^. This is likely due to the presence of aromatic residues (e.g., Trp286, Tyr431, Phe295) in the PAS, underscoring the importance of aromatic systems in designing mixed‑type AChE inhibitors. Furthermore, research demonstrates that the benzylpiperidine group of donepezil can be substituted with alkylamine or benzylamine moieties, which also interact with the CAS of AChE and exhibit potent inhibitory activity^33–35^. For safinamide, the aryl benzyl ether fragment is particularly critical, while its amide side chain can be replaced by a coumarin ring, formyl group, or other heterocycles without loss of MAO‑B inhibition^29^^,^^36^^,^^37^. This is attributed to the specific size and orientation of the aryl benzyl ether group, enabling it to occupy both the “entrance cavity” and “substrate cavity” of MAO‑B. Based on these insights, we first replaced the indanone moiety of donepezil with the aryl benzyl ether fragment of safinamide-a group expected to occupy both cavities of MAO‑B and interact with the PAS of AChE through π-π stacking and hydrophobic interactions. Subsequently, to reduce the molecular length, benzylpiperidine group was further optimised to alkylamine moieties, yielding a series of donepezil-safinamide hybrids (Figure 1). To explore the structure-activity relationship, systematic modifications were introduced to the linker, substitution position, and tertiary amine group.

## Results and discussion

### Chemistry

**Scheme 1.**
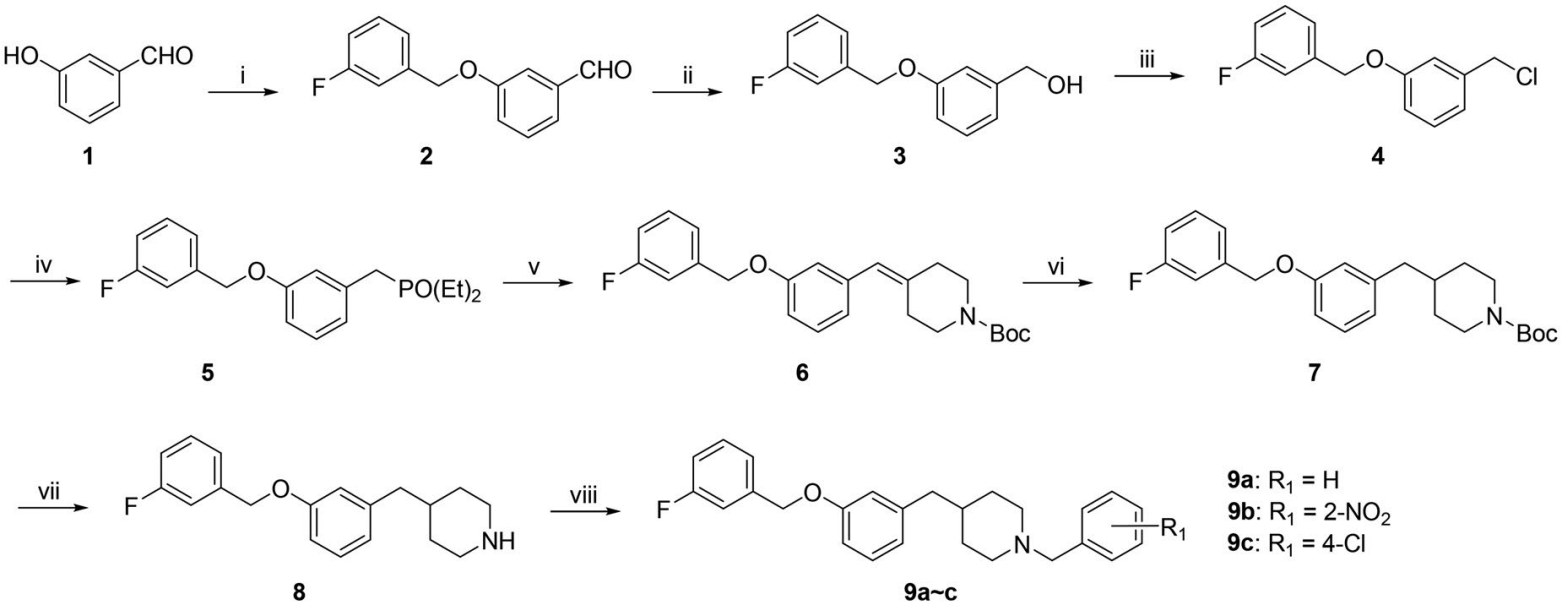
The synthesis of target compounds **9a**∼**c**. (i) K_2_CO_3_, KI, DMF, 1-(chloromethyl)-3-fluorobenzene, at rt for 24.0 h; (ii) NaBH_4_, THF, at rt for 12.0 h; (iii) SOCl_2_, CH_2_Cl_2_, reflux for 5.0 h; (iv) triethyl phosphite, at 130 °C for 4.0 h; (v) *N*-tert-Butoxycarbonyl-4-piperidone, 60% NaH, THF, at rt for 12.0 h; (vi) 5%Pd/C, hydrogen, at rt for 12.0 h; (vii) trifluoroacetic acid, CH_2_Cl_2_, at rt for 5.0 h; (viii) corresponding benzaldehydes, sodium triacetoxyborohydride, CH_2_Cl_2_, at rt for 2.0 h.

The target compounds **9a**∼**c** were synthesised according to the route illustrated in Scheme 1. Initially, condensation of 3-hydroxybenzaldehyde (**1**) with 3-fluorobenzyl chloride in the presence of K_2_CO_3_/DMF afforded 3-((3-fluorobenzyl)oxy)benzaldehyde (**2**) ^38^. This aldehyde was subsequently reduced with NaBH_4_ to give (3-((3-fluorobenzyl)oxy)phenyl)methanol (**3**) ^39^. Intermediate **3** was then treated with thionyl chloride to yield 1-(chloromethyl)-3-((3-fluorobenzyl)oxy)benzene (**4**), which was reacted with triethyl phosphite to form diethyl(3-((3-fluorobenzyl)oxy)benzyl)phosphonate (**5**) ^40^. Next, a Horner-Wadsworth-Emmons reaction between intermediate **5** and *N*-tert-butoxycarbonyl-4-piperidone using NaH/THF produced intermediate **6**^41^. Catalytic hydrogenation of **6** over Pd/C afforded intermediate **7**, which was subjected to Boc deprotection to furnish the key intermediate **8**^42^. Finally, reductive amination of **8** with the corresponding aldehydes yielded the target compounds **9a**∼**c**^43^.

**Scheme 2.**
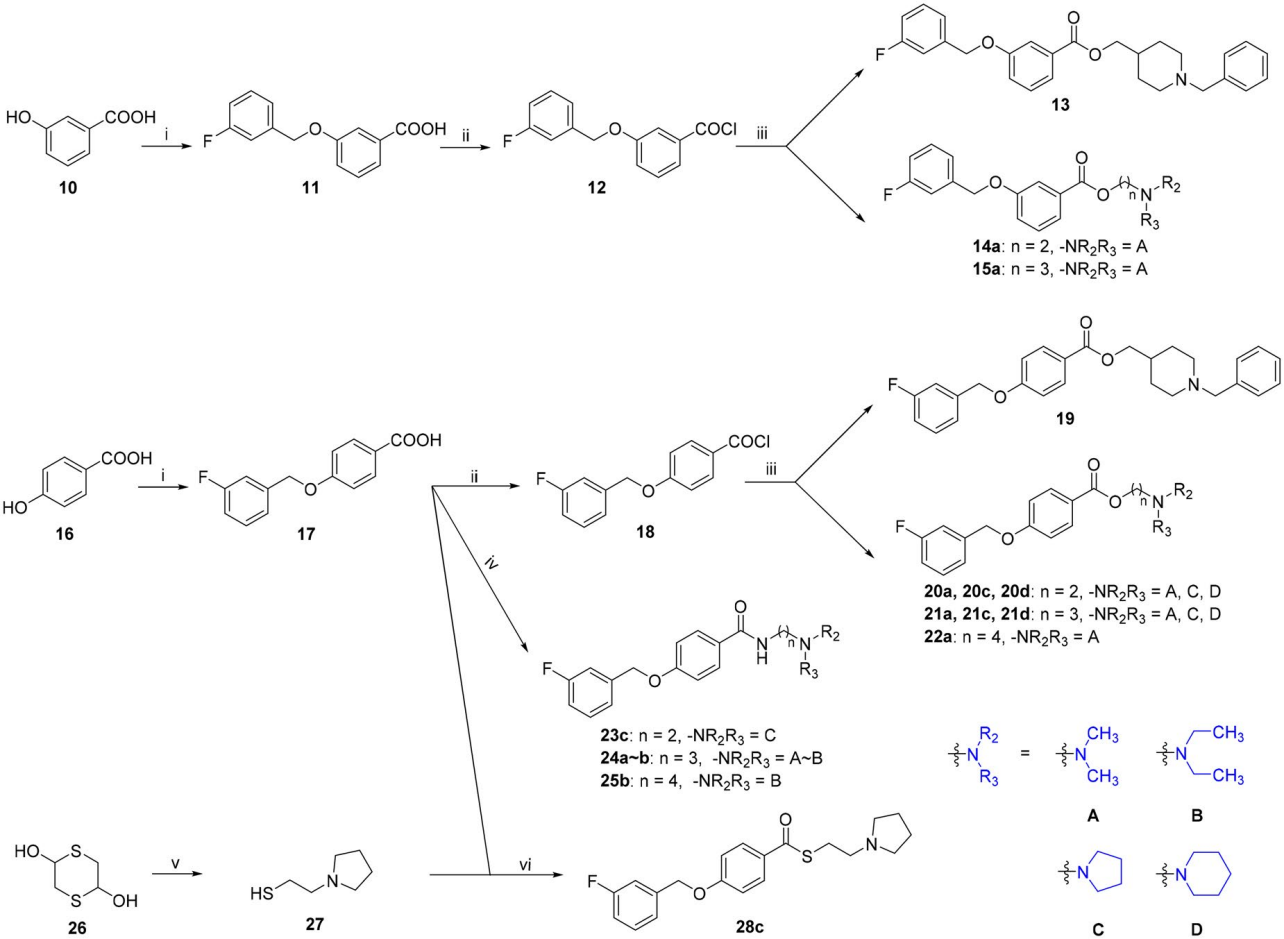
The synthesis of target compounds **13**∼**15**, **19**∼**25** and **28**. (i) KOH, KI, 1-(chloromethyl)-3-fluorobenzene, EtOH; (ii) SOCl_2_, DMF, CH_2_Cl_2_, reflux for 12.0 h; (iii) tertiary amine alkyl-1-ols, CH_2_Cl_2_, at rt for 12.0 h; (iv) EDCI, HOBT, THF, corresponding tertiary amine alkyl diamines, at rt for 12.0 h; (v) pyrrolidine, sodium triacetoxyborohydride, CH_2_Cl_2_, at rt for 2.0 h; (vi) EDCI, HOBT, THF, at rt for 12.0 h.

The target compounds **13**∼**15** and **19**∼**22** were synthesised using 3-hydroxybenzoic acid (**10**) or 4-hydroxybenzoic acid (**16**) as starting materials. Condensation of compounds **10** or **16** with 3-fluorobenzyl chloride in the presence of KOH/EtOH afforded 3-((3-fluorobenzyl)oxy)benzoic acid (**11**) or 4-((3-fluorobenzyl)oxy)benzoic acid (**17**), which were subsequently converted to 3-((3-fluorobenzyl)oxy) benzoyl chloride (**12**) or 4-((3-fluorobenzyl)oxy)benzoyl chloride (**18**) by the treatment with sulphonyl chloride. Reaction of intermediates of **12** or **18** with corresponding tertiary amine alkyl-1-ols produced target ester compounds **13**∼**15** and **19**∼**22**^44^.

Additionally, the target amide compounds **23**∼**25** were synthesised by condensation of intermediates of **11** or **17** with corresponding tertiary amine alkyl diamines using EDCI/HOBT as coupling agents^45^. As for the synthesis of target thioester compound **28c**, the 2-(pyrrolidin-1-yl)ethane-1-thiol (**27**) was first prepared *via* reductive amination of 2,5-dihydroxy-1,4-dithiane (**26**) with pyrrolidine. Subsequent condensation of intermediate **17** with **26** in the presence of EDCI/HOBT/THF produced the target thioester compound **28c**.

All the target compounds were characterised by ^1^H NMR,^13^C NMR and HR-MS. The purity was determined by high-performance liquid chromatography (HPLC) to be over 95.0%.

### Pharmacology

#### Evaluation of MAOs and ChEs inhibitory activities

The MAOs inhibitory activities of donepezil-safinamide hybrids were measured by kynuramine method. Safinamide and clorgyline were employed as positive controls^46^. As summarised in Table 1, all hybrids exhibited weak inhibition of MAO-A, along with varying levels of MAO-B inhibition. Among them, compound **20c** demonstrated the most potent MAO-B inhibitory activity, with an IC_50_ value of 0.0087 μM, approximately five-fold higher than that of safinamide. Additionally, compound **28c** also displayed considerable MAO-B inhibition (IC_50_ = 0.18 μM).

**Table 1.** Inhibitory effects on MAOs and ChEs of donepezil-safinamide hybrids and reference compounds *in vitro.*

Compound.	Structure	Inhibition rates (%) at 10.0 μM^a^				IC_50_ (μM)^b^	
		MAO-A	MAO-B	AChE	*h*BuChE	MAO-B	AChE
9a	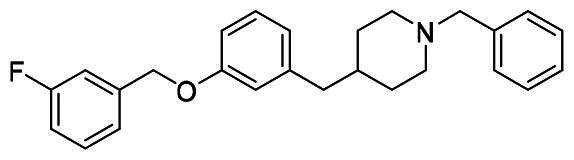	5.48 ± 0.12	37.96 ± 0.23	NT^c^	NT^c^	> 10.00	NT^c^
9b	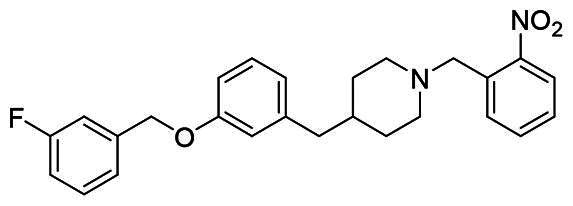	6.67 ± 0.35	22.85 ± 0.29	NT^c^	NT^c^	> 10.00	NT^c^
9c	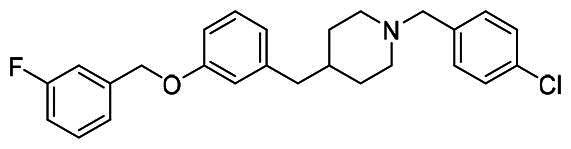	7.53 ± 0.44	51.73 ± 0.43	5.23 ± 0.12	n.a.^d^	8.73 ± 0.057	> 10.00
13	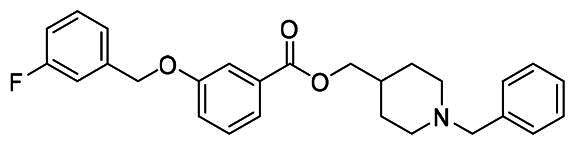	9.07 ± 0.87	32.34 ± 0.52	NT^c^	NT^c^	> 10.00	NT^c^
14a	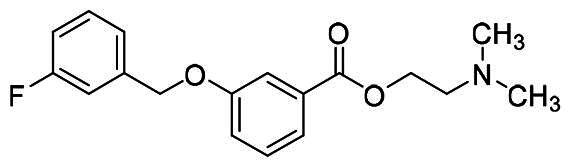	20.51 ± 0.14	92.45 ± 0.93	24.88 ± 0.55	n.a.^d^	0.42 ± 0.046	> 10.00
15a	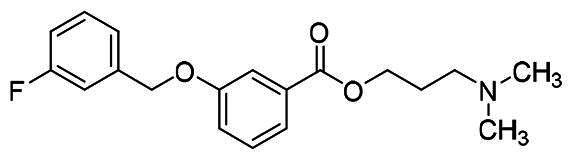	9.15 ± 0.42	59.81 ± 0.86	33.37 ± 0.64	n.a.^d^	3.16 ± 0.099	> 10.00
19	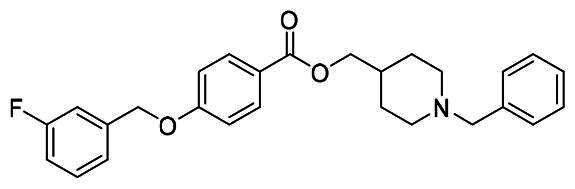	8.76 ± 0.85	48.42 ± 0.15	NT^c^	NT^c^	> 10.00	NT^c^
20a	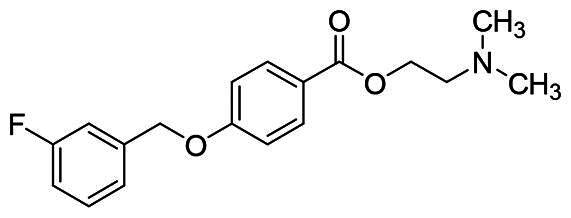	13.17 ± 0.68	98.38 ± 0.32	12.94 ± 0.87	n.a.^d^	0.073 ± 0.0067	> 10.00
20c	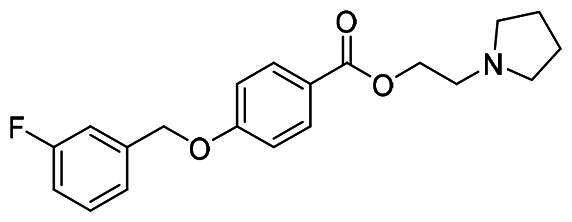	19.64 ± 0.07	99.99 ± 0.001	41.92 ± 0.93	n.a.^d^	0.0087 ± 0.00031	> 10.00
20d	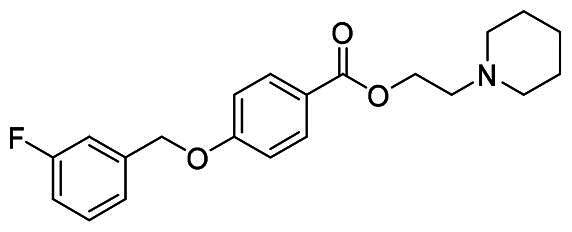	7.38 ± 0.19	57.49 ± 0.62	35.38 ± 0.25	n.a.^d^	6.48 ± 0.032	> 10.00
21a	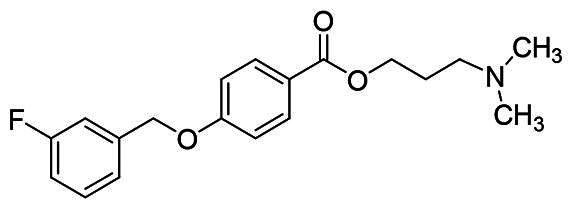	n.a.^d^	88.20 ± 0.61	58.82 ± 0.77	n.a.^d^	1.10 ± 0.016	7.31 ± 0.35
21c	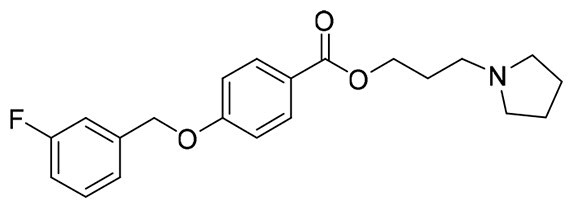	n.a.^d^	51.21 ± 0.23	59.37 ± 0.43	n.a.^d^	7.22 ± 0.082	6.45 ± 0.51
21d	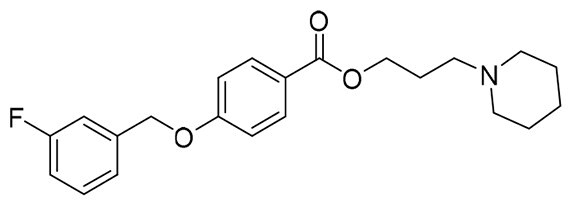	8.33 ± 0.66	33.38 ± 0.12	NT^c^	NT^c^	> 10.00	NT^c^
22a	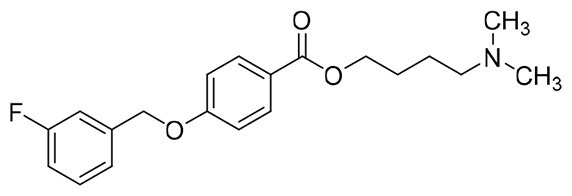	n.a.^d^	76.13 ± 0.89	60.92 ± 0.38	n.a.^d^	2.15 ± 0.099	3.96 ± 0.14
23c	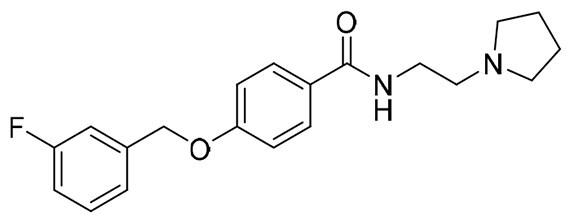	21.64 ± 0.35	86.21 ± 7.67	45.87 ± 0.67	n.a.^d^	1.01 ± 0.017	> 10.00
24a	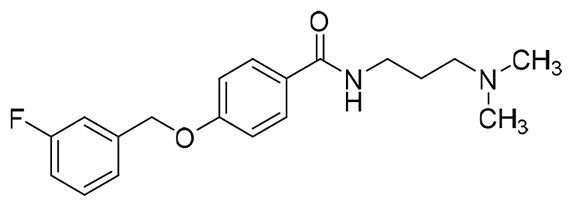	21.09 ± 0.38	21.27 ± 0.10	NT^c^	NT^c^	> 10.00	NT^c^
24b	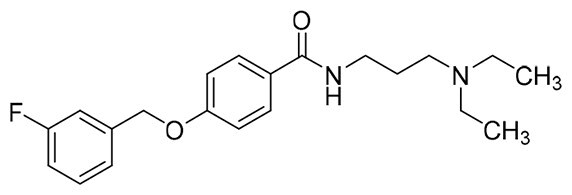	25.49 ± 0.71	26.65 ± 0.57	NT^c^	NT^c^	> 10.00	NT^c^
25b	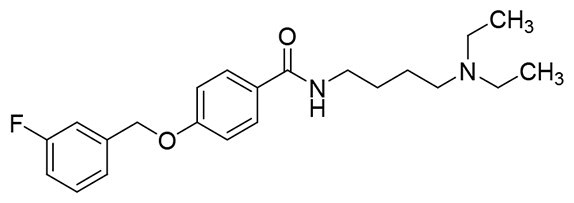	21.17 ± 0.98	20.97 ± 0.86	NT^c^	NT^c^	> 10.00	NT^c^
28c	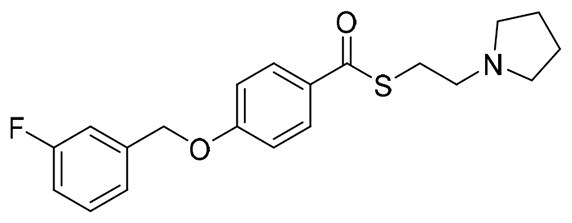	22.64 ± 0.35	98.44 ± 0.33	75.77 ± 0.94	n.a.^d^	0.18 ± 0.059	1.70 ± 0.11
Safinamide	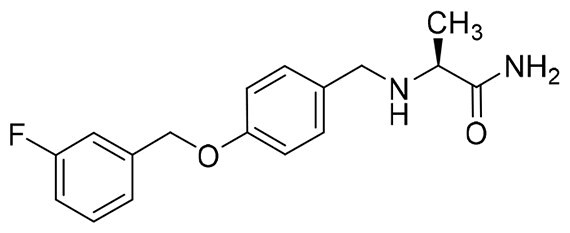	33.46 ± 0.95	99.98 ± 0.001	NT^c^	NT^c^	0.049 ± 0.0016	NT^c^
Clorgyline	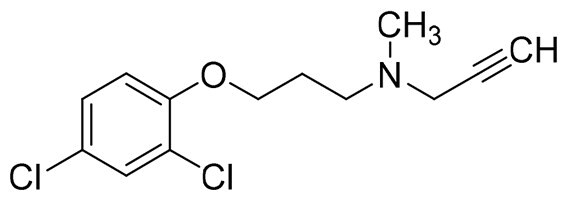	97.77 ± 0.74	64.23 ± 0.29	NT^c^	NT^c^	3.46 ± 0.079	NT^c^
Donepezil	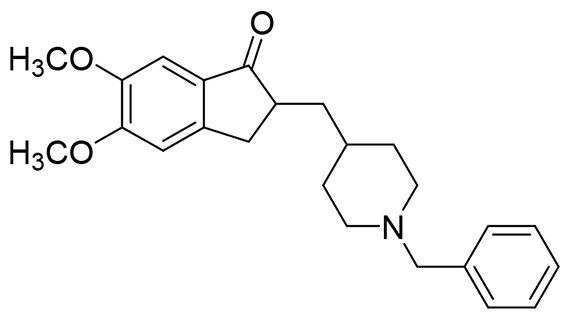	NT^c^	NT^c^	99.53 ± 0.21	41.6 ± 0.65	NT^c^	0.016 ± 0.0010
Rivastigmine	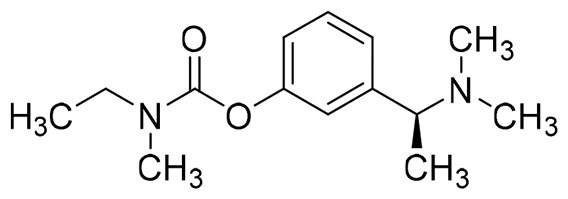	NT^c^	NT^c^	43.3 ± 0.74	58.92 ± 0.44	NT^c^	25.28 ± 0.23

^a^ Percent inhibition was determined at the concentration of 10.0 μM

^b^ the IC_50_ value is the concentration when the compounds inhibit enzyme activity by 50%.

^c^ NT = not tested.

^d^ n.a. = no active, meaning the percent inhibition is less than 5.0%.

Structure-activity relationship (SAR) analysis revealed that linking the benzyl pyridine pharmacophore of donepezil to the aryl benzyl ether moiety of safinamide generally resulted in weak MAO-B inhibition, regardless of whether the linker was alkyl or ester, or whether the substitution was at the para- or meta-position. However, MAO-B inhibitory activity was significantly enhanced upon simplifying the benzyl pyridine pharmacophore to a smaller basic tertiary amine moiety. Compounds **19**, **20a**, and **21a** exhibited stronger MAO-B inhibition compared to **13**, **14a**, and **15a**, which carry identical substituents and linkers, indicating that para-substitution is more favourable for MAO-B inhibition. Furthermore, the MAO-B inhibitory activities of **20a**∼**20d** were superior to those of **21a**∼**21d** and **22a** with the same substitutions, suggesting that extending the carbon chain length from two to three or four reduced MAO-B inhibitory potency. Moreover, compounds **20**∼**22** generally showed more potent MAO-B inhibition than **23**∼**25** and **28**, which share the same substituents and carbon chain length, implying that an ester linker facilitates interaction with MAO-B. Additionally, compounds containing a piperidyl group **(20d** and **21d**) consistently displayed weaker MAO-B inhibitory activity compared to analogues with the same linkers and substitution patterns, indicating that the introduction of a piperidyl group is detrimental to occupying the catalytic centre.

The ChE inhibitory activities of selected compounds, which exhibited good MAO-B inhibition (IC_50_ < 10.0 μM), were determined by using Ellman’s method^47^. Donepezil (AChE inhibition: IC_50_ = 0.016 μM) and rivastigmine (AChE inhibition: IC_50_ = 25.28 μM; BuChE inhibition: IC_50_ = 8.53 μM) were used as positive controls. As summarised in Table 1, all selected hybrids exhibited not activity to inhibit BuChE, along with varying levels of AChE inhibition. Among these compounds, **28c** showed the most potent AChE inhibitory activity (IC_50_ = 1.70 μM).

Compound **9c** showed weak AChE inhibition, revealing that linking the benzyl pyridine pharmacophore of donepezil to the aryl benzyl ether moiety of safinamide through methylene also resulted in weak AChE inhibition. Among these easter compounds (**14**∼**15** and **20**∼**22**), compound **20a** exhibited the poorest AChE inhibitory activity, while **22a** displayed best AChE inhibitory activity, reflecting that the extending the carbon chain length from two to three or four increased AChE inhibitory potency, which is inconsistent with the trend of their MAO-B inhibitory activities. Besides, the AChE inhibitory activities of **20c** and **21c** were stronger than those of compounds which possess the same linkers and substitution patterns, indicating that the introduction of a pyrrolidine group is good for the improvement of AChE inhibitory activity. Furthermore, compound **28c** exhibited the most potent AChE inhibition among the compounds **20c**, **23c** and **28c**, implying that a thioester linker facilitates inhibition of AChE.

#### Evaluation of MAO-B inhibitory mechanism

**Figure 2.**
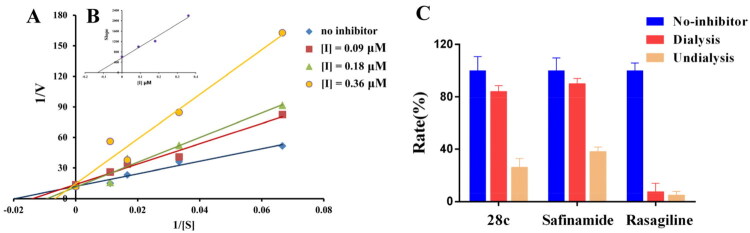
The MAO-B inhibitory mechanism of **28c**. (A) Lineweaver-Burk plots of MAO-B activities in the absence and presence of various concentrations of **28c** (0.09 μM, 0.18 μM and 0.36 μM); (B) The slopes of the Lineweaver-Burk plots versus the **28c** concentrations; (C)Reversibility study of **28c**, safinamide, and rasagiline.

To characterise the inhibitory mechanism of donepezil-safinamide hybrids on MAO-B, enzymatic kinetic analyses were performed^48^. Compound **28c** was selected for detailed investigation. The reciprocal plot (Lineweaver–Burk) presented in Figure 2(A) revealed that increasing concentrations of **28c** led to rises in both slopes (indicating decreased V_max_) and intercepts (reflecting increased K_m_). The linear fits converged on the Y-axis, consistent with a competitive inhibition mechanism. Using the slope replot of Lineweaver–Burk data against inhibitor concentration **(**Figure 2B), the competitive inhibition constant (K_i_) of **28c** was determined to be 0.13 μM.

To characterise the inhibition mode of donepezil-safinamide hybrids on MAO-B, a dialysis-based assay was conducted using compound **28c** as the representative test compound^49^. As established in previous studies, the inhibitory mechanism can be categorised into three types based on the recovery of MAO-B catalytic activity after dialysis: reversible inhibition (≥ 80% activity recovery), irreversible inhibition (≤ 20% recovery), and quasi-reversible inhibition (20–80% recovery). In this experiment, safinamide (a reversible inhibitor) and rasagiline (an irreversible inhibitor) were employed as reference compounds. As shown in Figure 2(C), the residual MAO-B activities before dialysis were 26.1% for **28c**, 38.3% for safinamide, and 4.9% for rasagiline. After dialysis, enzyme activity in the safinamide group recovered to 90.1%, consistent with reversible inhibition, while the rasagiline group showed only 7.5% recovery, confirming irreversible inhibition. Similarly, the activity in the **28c** dialysis group rebounded to 84.2%, demonstrating that it also acts as a reversible inhibitor of MAO-B.

**Figure 3.**
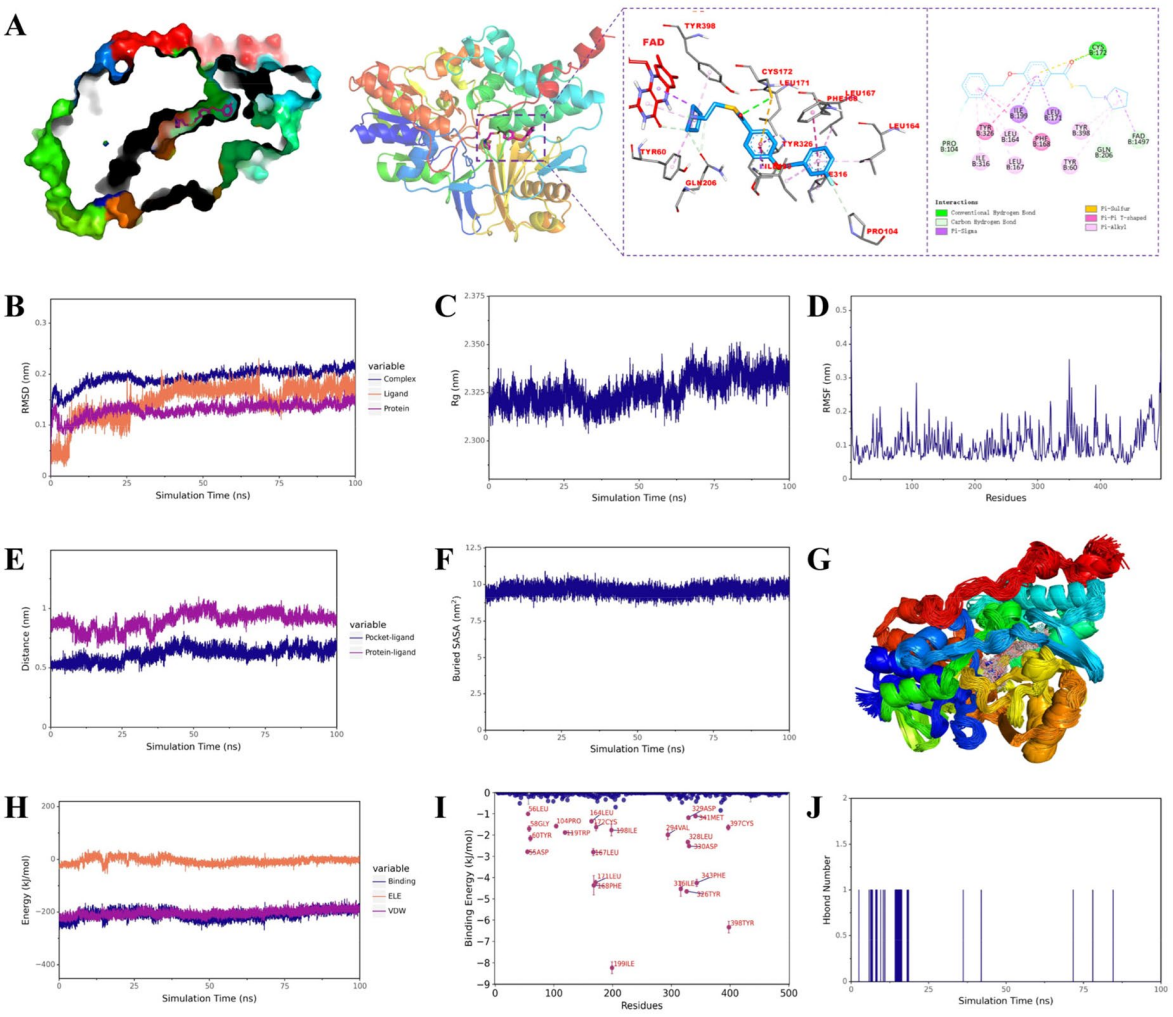
Molecular docking and molecular dynamic simulations of **28c** with MAO-B. (A) Molecular docking result of **28c** with MAO-B (PDB ID: *2v5z*); (B) RMSD of **28c**, MAO-B, and **28c**/MAO-B complex; (C) Rg of **28c**/MAO-B complex; (D) RMSF of MAO-B in the complex; (E) between the ligand and both the protein centre and binding site; (F) Buried SASA between **28c** and MAO-B; (G) Conformational overlap of **28c** throughout the trajectory in MAO-B; (H) The ELE, VDW and binding energy between **28c** and MAO-B; (I) The binding energy contributions of each animo acid residues; (H) Hbond number.

Molecular docking was performed to elucidate the interaction mode of **28c** with MAO-B (PDB ID: *2v5z*) by using Autodock 4.2 software^50^, and the result was shown in Figure 3(A). Similar with safinamide, the aryl benzyl ether moiety of **28c** occupies both the entrance and substrate cavities of MAO-B *via* van der Waals interaction and hydrophobic interaction with Leu 171, Ile199, Phe168, Leu164, Leu167, Tyr326, Ile316, Pro104, thereby blocking substrate access to the FAD cofactor. Additionally, its tertiary amine side chain also lies in close proximity to FAD, occupies a hydrophilic region of the enzyme and interacts with Tyr398, Tyr60, Gln206. However, in the binding mode of **28c** with MAO-B, **28c** forming a hydrogen bond with Cys172 rather than Gln206. These finding revealed that **28c** showed strong and similar binding mode with safinamide, which is also consistent with the finding of kinetic and reversible studies.

To evaluate the dynamic behaviour and stability of the complex formed by **28c** and MAO-B, molecular dynamics (MD) simulations were conducted for 100 ns using Gromacs2022^51^. The root mean square deviation (RMSD) values for the ligand, protein, and complex remained below 0.3 nm throughout the simulation. After initial fluctuations, these curves stabilised beyond 25 ns (Figure 3B), indicating increasing structural stability and suggesting a tight binding interaction between **28c** and MAO-B. Similarly, the radius of gyration (Rg) stabilised over time (Figure 3C), reflecting compaction and conformational stability of the complex. Furthermore, low root mean square fluctuation (RMSF) values were observed for residues within the binding pocket (Figure 3D), indicating reduced flexibility around the ligand and reinforcing the stability of the binding interface. This interpretation is corroborated by the B-factor profile derived from the simulation (Figure S1A), which supports the presence of rigid and well-defined interactions. Additionally, the distance between the ligand and both the protein centre and binding site converged to stable values during the simulation (Figure 3E), confirming the stability of binding within the target pocket. The consistently stable buried solvent-accessible surface area (SASA) (Figure 3F) and high degree of conformational overlap of **28c** throughout the trajectory (Figure 3G) further substantiate the stability of the complex. Although the hydrogen bond count between **28c** and MAO-B fluctuated between 0 and 1 and was frequently near zero (Figure 3J), suggesting limited hydrogen bonding contribution. Van der Waals (VDW) and electrostatic (ElE) interactions remained stable throughout the simulation (Figure 3H), indicating their dominant role in binding stability. The binding energies were further calculated by using molecular mechanics-poisson boltzmann surface area (MM-PBSA) method, and the results were shown in Table 2. Obviously, the van der Waals interaction energy (ΔE_vdw_ = −209.31 kJ/mol) contributes more significantly than the hydrophobic interaction energy (ΔE_nonpol_ = −26.72 kJ/mol), and both are substantially greater than the electrostatic interaction energy (ΔE_ele_ = −1.07 kJ/mol). Thus, within the binding energy composition, van der Waals interactions serve as the dominant factor, hydrophobic interactions play a secondary role, and electrostatic effects provide additional stabilisation. Besides, the calculated ΔE_MMPBSA_ value was −167.07 kJ/mol, indicating a strong binding affinity between **28c** with MAO-B. The binding energy contributions of each animo acid residues were further analysed (Figure 3I), and the results showed that Ile199 and Tyr398 exhibited the most favourable binding energy contributions, suggesting that these residues play a key role in the interaction between MAO-B and **28c**. Further considering the results of surface electrostatic potential analysis (Figure S1B), the MD simulation results revealed that **28c** could strongly and stably bind to MAO-B and van der Waals interactions are the dominant contributor to the binding energy.

**Table 2.** Calculated binding energies and its compositions.

Complex	ΔE_vdw_	ΔE_ele_	ΔE_pol_	ΔE_nonpol_	ΔE_MMPBSA_^a^
**28c/MAO-B**	−209.31 ± 1.57	−1.07 ± 0.25	70.03 ± 1.67	−26.72 ± 0.05	−167.07 ± 0.93
**28c/AChE**	−186.21 ± 5.22	−12.38 ± 1.50	71.97 ± 4.47	−25.18 ± 0.51	−151.81 ± 2.95

^a^ ΔE_MMPBSA_ = ΔE_vdw_ + ΔE_ele_ + ΔE_pol_ + ΔE_nonpol_

#### Evaluation of AChE inhibitory mechanism

**Figure 4.**
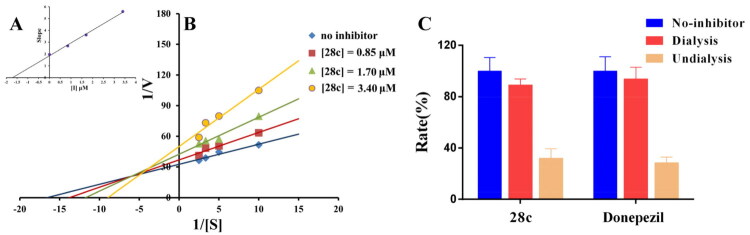
The AChE inhibitory mechanism of **28c**. (A) The slopes of the Lineweaver-Burk plots versus the **28c** concentrations; (B) Lineweaver-Burk plots of AChE activities in the absence and presence of various concentrations of **28c** (0.85 μM, 1.70 μM and 3.40 μM); (C) Reversibility study of **28c** and donepezil.

To elucidate the inhibitory mechanism of donepezil-safinamide hybrids against AChE, enzymatic kinetic assays were carried out^52^. Compound **28c** was chosen for further detailed analysis. As depicted in the Lineweaver-Burk plot (Figure 4B), increasing concentrations of **28c** resulted in elevated slopes (suggesting a decrease in V_max_) and increased intercepts (indicating a rise in K_m_). The linear fittings converged in the fourth quadrant, consistent with a mixed-type inhibition mode. Using the slope replot of the Lineweaver-Burk data versus inhibitor concentration (Figure 4A), the Ki for **28c** was calculated to be 1.71 μM.

To further clarify the inhibition mode, a dialysis-based experiment was performed with compound **28c** as a representative inhibitor^53^. As illustrated in Figure 4(C), the residual AChE activities before dialysis were 32.0% for **28c** and 28.4% for donepezil. After dialysis, the enzyme activity in the donepezil group recovered to 93.8%, confirming its reversible inhibition profile. Similarly, the **28c**-treated group showed a recovery of activity to 89.1%, demonstrating that **28c** also functions as a reversible inhibitor of AChE.

**Figure 5.**
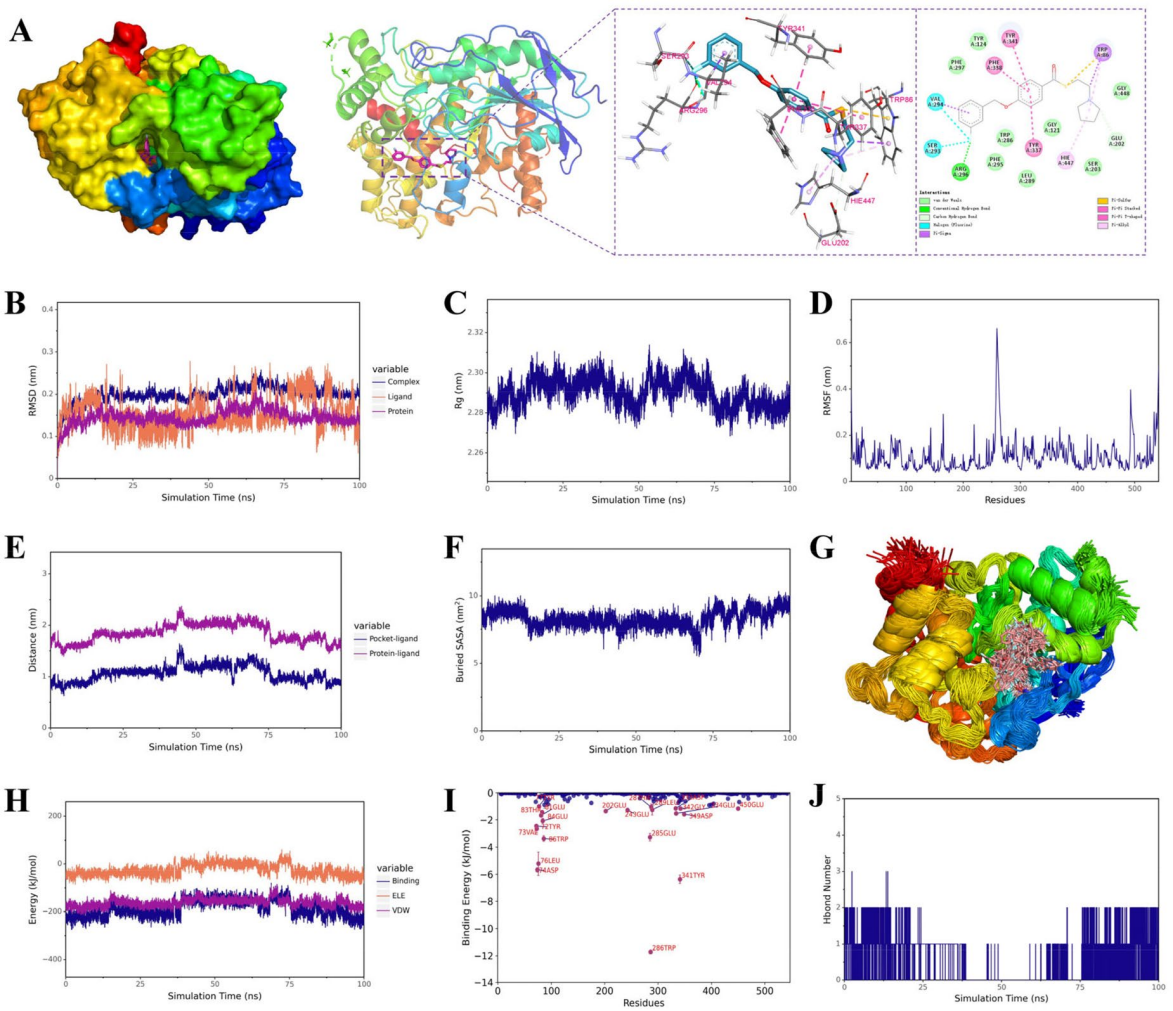
Molecular docking and molecular dynamic simulations of **28c** with AChE. (A) Molecular docking result of **28c** with AChE (PDB ID: *4ey7*); (B) RMSD of **28c**, AChE, and **28c**/AChE complex; (C) Rg of **28c**/AChE complex; (D) RMSF of AChE in the complex; (E) between the ligand and both the protein centre and binding site; (F) Buried SASA between **28c** and AChE; (G) Conformational overlap of **28c** throughout the trajectory in AChE; (H) The ELE, VDW and binding energy between **28c** and AChE; (I) The binding energy contributions of each animo acid residues; (H) H bond number.

To gain insights into the binding mode of **28c**, a molecular docking study was performed against AChE (PDB ID: *4ey7*) ^54^. As illustrated in Figure 5(A), the tertiary amine side chain of **28c** occupies the catalytic anionic site (CAS), engaging the catalytic triad (Ser203, Glu202, His447) through van der Waals and π-alkyl interactions. Concurrently, its aryl benzyl ether moiety binds to the peripheral anionic site (PAS), forming π-π stacking with Tyr337, Phe338, and Tyr341. This dual-site binding mode correlates well with the observed mixed-type inhibition, consistent with the kinetic analysis. Additionally, **28c** forms extensive interactions with other residues (Ser293, Val294, Arg296, Phe297, Phe295, Trp286, Leu289, Tyr124, Gly121, Gly448, and Trp86) *via* van der Waals forces, hydrogen bond, halogen bonding (fluorine), π-sigma, and π-sulfur interactions. Notably, the binding pose of **28c** closely resembles that of donepezil, which aligns with the kinetic and reversible binding data.

Molecular dynamics (MD) simulations were performed using Gromacs2022 to assess the dynamic behaviour and stability of the complex between compound **28c** and AChE^55^. The root mean square deviation (RMSD) values for the ligand, protein, and complex all remained below 0.3 nm, though their trajectories displayed fluctuations over the course of the simulation (Figure 5B). Similar trends were observed in the radius of gyration (Rg) curves (Figure 5C), the distance between the ligand and both the protein centre and binding site (Figure 5E), the buried (SASA) curve (Figure 5F), as well as in the VDW, ELE, and total binding energy profiles (Figure 5H). Although most binding pocket residues exhibited low root mean square fluctuation (RMSF) values (Figure 5D) and the ligand conformation remained highly consistent throughout the trajectory (Figure 5G), the B-factor analysis derived from the simulation indicated elevated flexibility around the catalytic anionic site (CAS) (Figure S1C). The ΔE_MMPBSA_ was calculated to be −151.81 kJ/mol (Table 2). Together with the observations above, it can be concluded that although compound **28c** binds stably to AChE, the complex displays greater conformational flexibility compared to the **28c**/MAO-B complex. Additionally, the number of hydrogen bonds between **28c** and AChE fluctuated between 0 and 2, indicating a limited contribution from hydrogen bonding. The ΔEᵥ_dw_ (−186.21 kJ/mol) was found to play a more substantial role than ΔG_nonpol_ (−25.18 kJ/mol), and both were significantly greater than the ΔEₑₗₑ = (−12.38 kJ/mol). Further supported by the surface electrostatic potential analysis (Figure S1D), these results indicate that van der Waals interactions are the dominant contributor to the binding energy. The binding energy contributions of each animo acid residues were further analysed (Figure 5I), and the results showed that Trp286 and Tyr341 exhibited the most favourable binding energy contributions.

#### Blood-brain barrier permeation study

The drug-like properties and blood-brain barrier (BBB) permeation ability of **28c** was firstly predicted by using two different platform (ADMETlab and SwissADME) ^56–57^. The results shown in Table 3 revealed that **28c** followed Lipinski’s rules and could cross the BBB.

**Table 3.** Prediction of drug-like properties and BBB permeation for **28c**.

Compound	Platform	MW*^c^*	HBDs*^c^*	HBAs*^c^*	Log P*^c^*	TPSA*^c^*	BBB (±)*^c^*
**28c**	ADMETlab*^a^*	359.46	0	4	4.37	29.54	+
**28c**	SwissADME*^b^*	359.46	0	4	4.22	54.84	+
**CNS (+) drugs**		≤ 450	≤ 5	≤ 10	≤ 5	≤ 90	

^a^ The ADMETlab web platform is freely accessible at http://admet.scbdd.com/.

^b^ SwissADME is freely accessible at http://www.swissadme.ch.

^c^ MW: molecular weight; HBDs: H-bond donors; HBAs: H-bond acceptors; Log P: log octanol/water partition coefficient; TPSA: topological polar surface area; BBB (±): BBB permeability.

The BBB permeability of compound **28c** was further predicted using a parallel artificial membrane permeability assay (PAMPA). To establish a reference standard, the permeabilities of 11 known drugs were determined (Table S1). The obtained values showed a good linear correlation with literature data: *Pe* (exp.) = 0.8792 × *Pe* (bibl.) − 0.0616 (*R*^2^ = 0.9555) (Figure S2). With reference to the permeability limit set by Di et al.^58^, compounds exhibiting a value exceeding 3.46 × 1 0 ^–6 ^cm/s were classified as BBB-penetrant. (Table S2). The predicted BBB permeability of **28c** was then measured and is presented in Table 4. The *Pe* value of **28c** (11.92 × 1 0 ^–6 ^cm/s) was significantly higher than the threshold, indicating that it can cross the BBB and inhibit both MAO-B and AChE in the brain.

**Table 4.** Permeability results *P*_e_ (× 1 0 ^–6 ^cm/s) for **28c** and its predicted penetration into CNS.

Compound^a^	*Pe (× 10^–6^ cm/s)b*	Prediction
**28c**	11.92 ± 0.11	CNS +

^a^ **28c** was dissolved in DMSO at 5 mg/mL and diluted with PBS/EtOH (70:30). The final concentration of **28c** was 100 μg/mL.

^b^ Data are the mean ± SD of three independent experiments.

Besides, the level of **28c** in mice brain 10 min after intragastric administration of 50 mg/kg was also detected. And compound **28c** displayed 12.8 ng/g exposure in brain tissue, confirming **28c** had ability to penetrate the BBB *in vivo*.

#### Stability study

**Figure 6.**
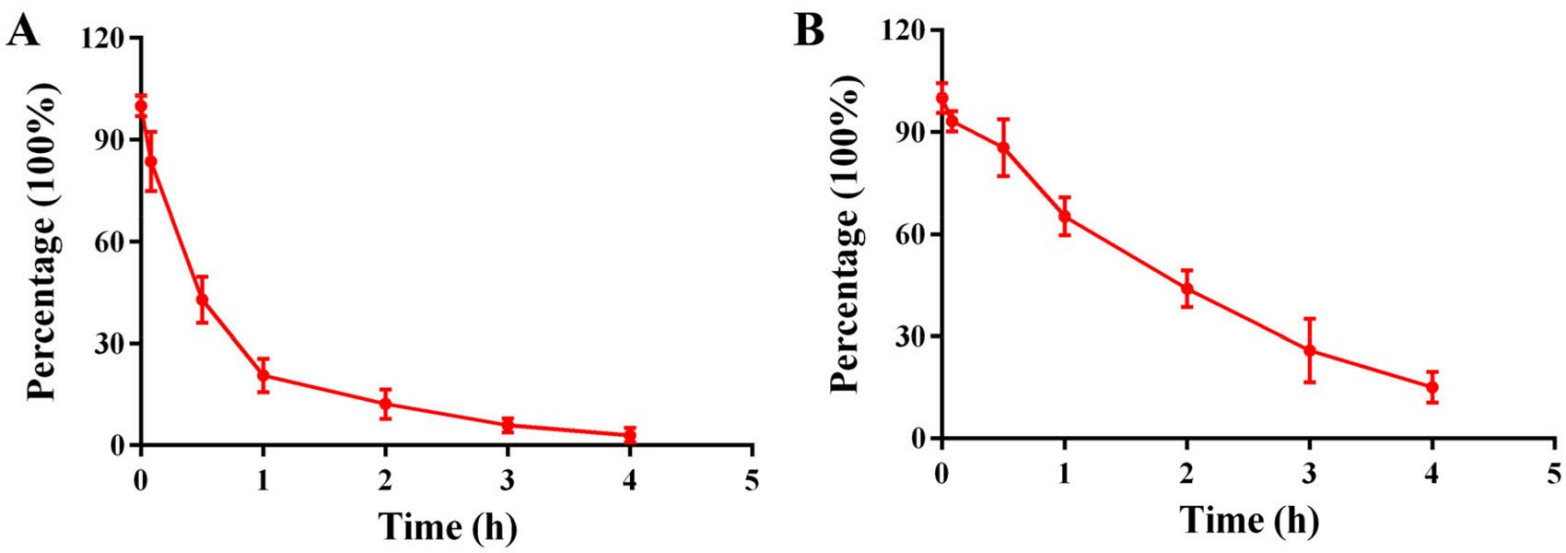
Degradation of compound **28c** in mouse plasma and brain homogenate. (**A**) Concentration of **28c** remaining in plasma. (**B**) Concentration of **28c** remaining in brain homogenate.

The stability of compound **28c** was further evaluated in mouse plasma and brain homogenate using HPLC^59^. As shown in Figure 6(A), 28c underwent hydrolysis in plasma with a half-life of approximately 0.5 h, indicating a relatively slow degradation process. In contrast, the compound remained more stable in brain homogenate, exhibiting a longer half-life of about 2 h (Figure 6B). Given its effective BBB penetration, **28c** is expected to have sufficient time in the brain to exert its inhibitory effects on AChE and MAO-B.

#### Safety study

**Figure 7.**
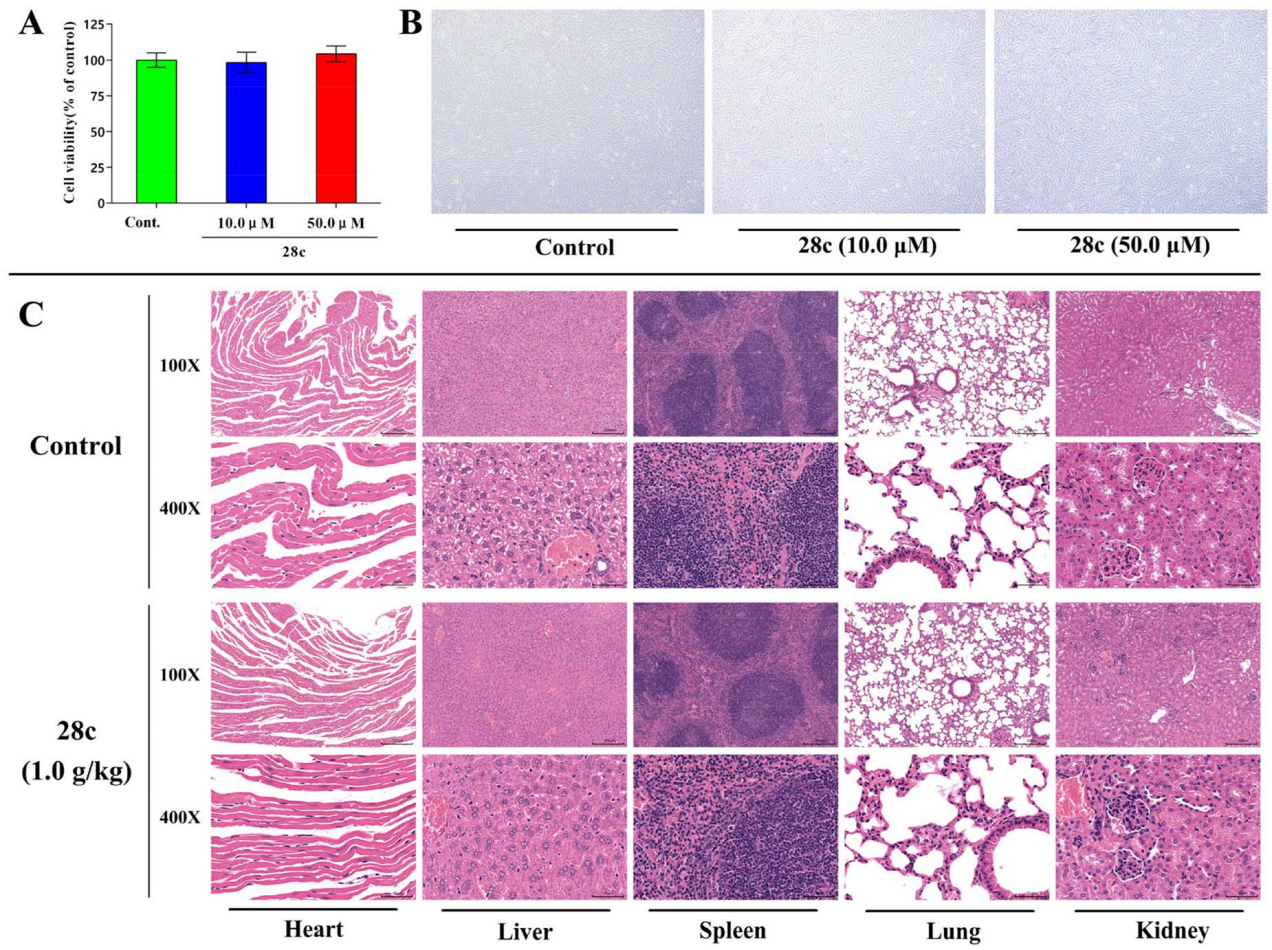
Safety study of **28c**. (**A**) Cytotoxicity of **28c** towards PC-12 cells. (**B**) The morphology of PC-12 cells with or without **28c**. (**C**) H&E staining of heart, liver spleen, lung and kidney from **28c**-treated mice.

The cytotoxicity of **28c** on PC12 cells was firstly measured by using Cell Counting Kit-8 (CCK-8), and the morphology of PC12 cells was also recorded^60^. As shown in Figure 7(A) and 7B, neither cell viability nor morphology exhibited significant changes even at **28c** concentrations up to 50.0 μM, indicating an absence of cytotoxicity below this concentration. These results suggest that **28c** has a broad therapeutic window.

In addition, the acute toxicity of **28c** was evaluated in male C57BL/6J mice at a dose of 1.0 g/kg^61^. Throughout the 14-day observation period, no abnormal behaviours or toxic symptoms were observed. The mice displayed no abnormal secretions from the eyes, nose, or mouth, and no swelling was detected. Body weight increased normally, and no mortality occurred. At the end of the study, the mice were euthanized and subjected to gross anatomical examination, which revealed no abnormalities in the morphology, position, or colour of any organs. Furthermore, hematoxylin-eosin (HE) staining of vital organs (brain, heart, liver, spleen, lung, and kidney) showed no discernible pathological changes, indicating that **28c** is well-tolerated at doses up to 1.0 g/kg without signs of acute toxicity (Figure 7C).

#### Pharmacodynamics study in vivo

**Figure 8.**
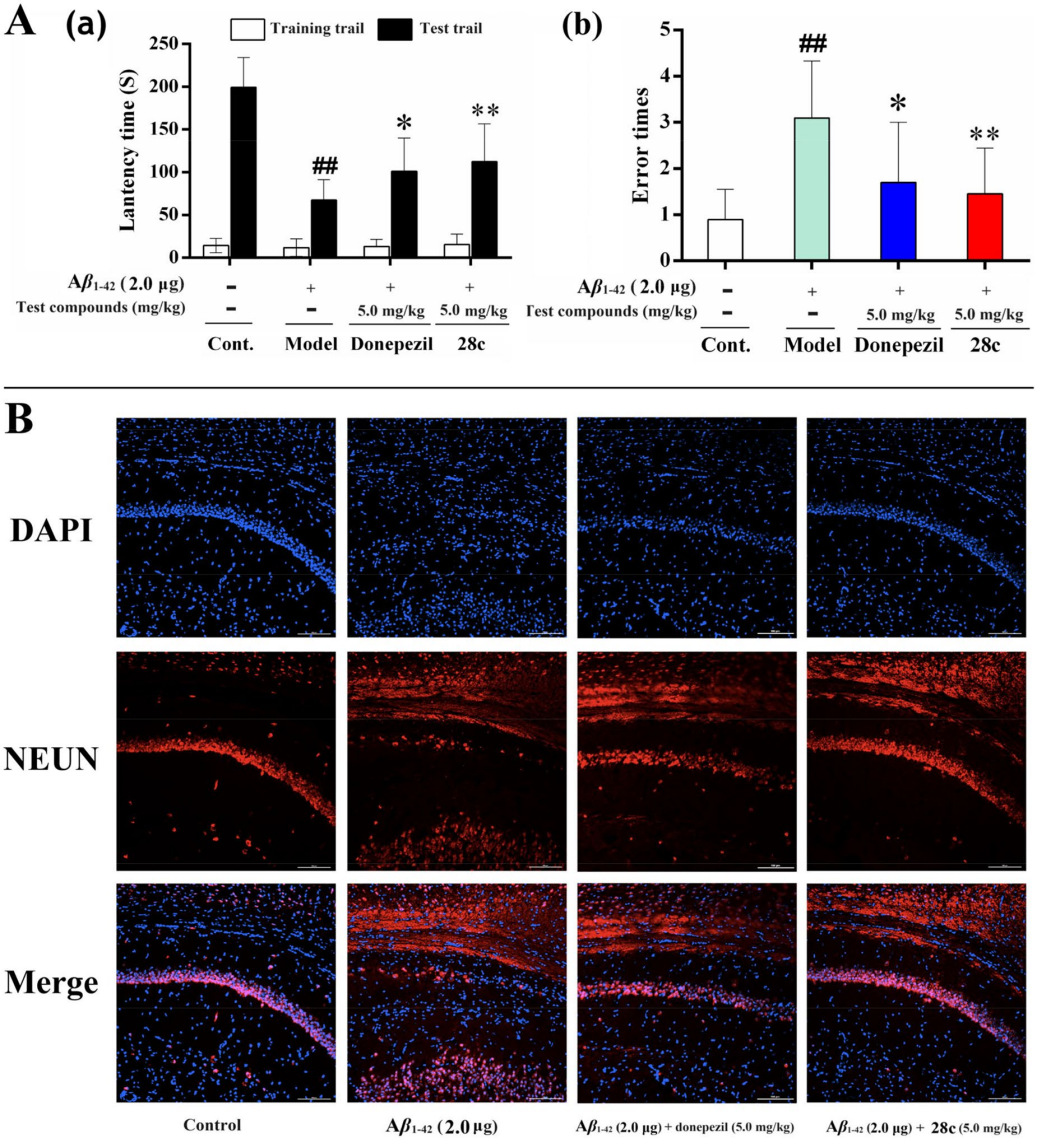
Effects of **28c** on A*β*_1-42_ induced mice mode. (A) Step-down passive avoidance test; (B) immunofluorescence analysis. ^##^*p* < 0.01 *vs* control group; *p < 0.05 *vs* model group; **p < 0.01 *vs* model group.

Given the promising anti-AD properties demonstrated by compound **28c** *in vitro*, the *in vivo* anti-AD efficacy of **28c** was further evaluated in an A*β*_1-42_-induced AD mouse model^62^. In the step-down passive avoidance test, mice receiving an intracerebroventricular injection of A*β*_1-42_ (2.0 μg) exhibited a significant decrease in latency time and an increase in error frequency (Figure 8A), confirming the successful induction of memory and learning deficits. Treatment with either donepezil (5.0 mg/kg) or compound **28c** (5.0 mg/kg) *via* intragastric administration effectively prolonged the latency period and reduced error counts, indicating that both compounds ameliorated A*β*_1-42_-induced AD-like symptoms. Additionally, compound **28c** produced similar effects with donepezil at an equal dose.

Subsequent immunofluorescence analysis further supported these findings (Figure 8B)^63^. A*β*_1-42_ injection resulted in a marked reduction of hippocampal neurons, indicating substantial hippocampal damage. Consistent with the behavioural outcomes, both donepezil and compound **28c** treatments conferred neuroprotective effects, preserving neuronal density in the hippocampus. Notably, compound **28c** outperformed donepezil at the same concentration in terms of protective efficacy.

In summary, these *in vivo* results demonstrate the efficacy of compound **28c** in alleviating AD symptoms and providing neuroprotection in the hippocampus.

## Conclusion

Taking together, a series of donepezil-safinamide hybrids were designed and synthesised in this study. Through systematic optimisation, compound **28c** was identified as a potent inhibitor of AChE (IC_50_ = 1.70 μM) and MAO-B (IC_50_ = 0.18 μM). Mechanistic studies indicated that **28c** acts as a reversible mixed-type inhibitor of AChE and a competitive reversible inhibitor of MAO-B. Molecular docking and molecular dynamic simulations revealed that **28c** could strongly and stably bind to MAO-B and AChE mainly through van der Waals interactions. Moreover, compound **28c** demonstrated effective blood-brain barrier penetration, exhibited suitable stability in mouse plasma and brain homogenate, and showed a favourable safety profile both *in vitro* and *in vivo*. Furthermore, *in vivo* evaluations demonstrated that **28c** can attenuate AD-related symptoms and exert hippocampal neuroprotection effect. These findings highlight **28c** as a highly promising candidate for further development as an anti-AD agent.

## Experimental section

### Chemistry

Reagents were obtained from commercial suppliers (Adamas, Aladdin, and InnoChem). Reaction progress was monitored by thin-layer chromatography (TLC), and spots were visualised under iodine vapour or UV light (254 nm). Column chromatography was performed using silica gel (230–400 mesh) purchased from Adamas.^1^H NMR and^13^C NMR spectra were recorded in CDCl_3_ on a Varian INOVA spectrometer at 25 °C, with tetramethylsilane (TMS) as the internal standard. The purity of the target compound was determined using a Wayeal LC-3200 system equipped with a Welchmat C18 column (4.6 mm × 150 mm, 5 μm). Mass spectra were acquired on either a Shimadzu LC-MS 2020 spectrometer or a Thermo Scientific Q Exactive Plus mass spectrometer.

#### Synthesis of intermediate 2

To a round-bottom flask, *m*-hydroxybenzaldehyde (12.2 g, 99.9 mmol), K_2_CO_3_ (20.7 g, 149.8 mmol), KI (0.8 g, 4.8 mmol), and DMF (75.0 ml) were added. The mixture was stirred at r.t. for 15.0 min. Then, 1-(chloromethyl)-3-fluorobenzene (12.0 ml, 99.0 mmol) was added slowly. After the addition was complete, the reaction mixture was stirred at r.t. for 24.0 h. It was then quenched with a cold saturated aqueous solution of Na_2_CO_3_ (225.0 ml). The resulting mixture was extracted with ethyl acetate (150.0 ml × 3). The combined organic layers were washed successively with water (300.0 ml) and saturated brine (300.0 ml). After drying over anhydrous Na_2_SO_4_, the solution was concentrated under reduced pressure to afford intermediate **2**.

#### Synthesis of intermediate 3

To a round-bottom flask, intermediate **2** (18.4 g, 79.9 mmol) and anhydrous THF (75.0 ml) were added. The mixture was kept at 0 ∼ 5 °C for 10.0 min, and then NaBH_4_ (7.5 g, 198.3 mmol) was in three times, with a half-hour interval between each addition. After the addition was complete, the reaction mixture was stirred at r.t. for 12.0 h. It was then quenched with 10% HCl aqueous solution (125.0 ml) at 0 ∼ 5 °C. The resulting mixture was extracted with ethyl acetate (150.0 ml × 3). The combined organic layers were washed successively with water (300.0 ml) and saturated brine (300.0 ml). After drying over anhydrous Na_2_SO_4_, the solution was concentrated under reduced pressure to afford intermediate **3**. ^1^H NMR (400 MHz, CDCl_3_) *δ* 7.35 (td, *J*_1_ = 8.0 Hz, *J*_2_ = 5.6 Hz, 1H), 7.29 (t, *J* = 8.0 Hz, 1H), 7.21-7.10 (m, 2H), 7.04-6.99 (m, 2H), 6.97 (d, *J* = 7.6 Hz, 1H), 6.89 (dd, *J*_1_ = 7.6 Hz, *J*_2_ = 2.8 Hz, 1H), 5.08 (s, 2H), 4.69 (s, 2H).

#### Synthesis of intermediate 4

To a round-bottom flask, intermediate **3** (16.2 g, 69.8 mmol) and anhydrous CH_2_Cl_2_ (75.0 ml) were added. The mixture was stirred at r.t. for 15.0 min. Then, SOCl_2_ (7.6 ml, 104.6 mmol) was added slowly. After the addition was complete, the reaction mixture was refluxed at argon atmosphere for 5 h. The resulting solution was then concentrated under reduced pressure to afford intermediate **4**. ^1^H NMR (400 MHz, CDCl_3_) *δ* 7.36 (td, *J*_1_ = 8.0 Hz, *J*_2_ = 6.0 Hz, 1H), 7.28 (t, *J* = 8.0 Hz, 1H), 7.21-7.14 (m, 2H), 7.04-6.99 (m, 3H), 6.92 (dd, *J*_1_ = 8.4 Hz, *J*_2_ = 2.0 Hz, 1H), 5.07 (s, 2H), 4.56 (s, 2H).

#### Synthesis of intermediate 5

To a round-bottom flask, intermediate **4** (17.3 g, 69.0 mmol) and triethyl phosphite (14.3 ml, 82.8 mmol) were added. The mixture was stirred at 130 °C for 4.0 h. Then, it was purified by using column chromatography to afford pure intermediate **5**. ^1^H NMR (400 MHz, CDCl_3_) *δ* 7.34 (td, *J*_1_ = 8.0 Hz, *J*_2_ = 6.0 Hz, 1H), 7.23 (t, *J* = 8.0 Hz, 1H), 7.19 (d, *J* = 8.0 Hz, 1H), 7.16-7.14 (m, 1H), 7.00 (td, *J*_1_ = 8.0 Hz, *J*_2_ = 2.4 Hz, 1H), 6.94 (brs, 1H), 6.91 (d, *J* = 8.0 Hz, 1H), 6.85 (d, *J* = 8.0 Hz, 1H), 5.06 (s, 2H), 4.06-3.94 (m, 4H), 3.13 (d, *J* = 21.6 Hz, 2H), 1.24 (t, *J* = 7.2 Hz, 6H).

#### Synthesis of intermediate 6

To a round-bottom flask, intermediate **5** (10.6 g, 30.1 mmol), *N*-tert-Butoxycarbonyl-4-piperidone (7.2 g, 36.1 mmol) and anhydrous THF (50.0 ml) were added. The mixture was kept at 0 ∼ 5 °C for 10.0 min, and then 60% NaH (3.0 g, 75.0 mmol) was added slowly. After the addition was complete, the reaction mixture was stirred at r.t. for 12.0 h. It was then quenched with H_2_O (125.0 ml) and 10% HCl aqueous solution at 0 ∼ 5 °C to pH = 3 ∼ 4. The resulting mixture was extracted with ethyl acetate (150.0 ml × 3). The combined organic layers were washed successively with water (300.0 ml) and saturated brine (300.0 ml). After drying over anhydrous Na_2_SO_4_, the solution was concentrated under reduced pressure and purified by using column chromatography to afford intermediate **6**. ^1^H NMR (400 MHz, CDCl_3_) *δ* 7.35 (td, *J*_1_ = 8.0 Hz, *J*_2_ = 6.0 Hz, 1H), 7.24 (t, *J* = 8.0 Hz, 1H), 7.19 (d, *J* = 8.0 Hz, 1H), 7.17-7.12 (m, 1H), 7.01 (td, *J*_1_ = 8.4 Hz, *J*_2_ = 2.4 Hz, 1H), 6.84-6.78 (m, 3H), 6.32 (s, 1H), 5.06 (s, 2H), 3.50 (t, *J* = 6.0 Hz, 2H), 3.37 (t, *J* = 6.0 Hz, 2H), 2.41 (t, *J* = 6.0 Hz, 2H), 2.32 (t, *J* = 6.0 Hz, 2H), 1.48 (s, 9H). ^13^C NMR (100 MHz, CDCl_3_) *δ* 164.25-161.80 (d, *J*_C-F_ = 244.6 Hz), 158.32, 154.78, 139.76-139.69 (d, *J*_C-F_ = 7.6 Hz), 138.95, 138.88, 130.19-130.11 (d, *J*_C-F_ = 8.1 Hz), 129.26, 124.28, 122.67-122.64 (d, *J*_C-F_ = 2.9 Hz), 121.92, 115.40, 114.88-114.67 (d, *J*_C-F_ = 21.1 Hz), 114.29-114.07 (d, *J*_C-F_ = 22.0 Hz), 112.81, 79.60, 69.14-69.12 (d, *J*_C-F_ = 2.0 Hz), 36.19 (2 C), 29.25 (2 C), 28.47 (3 C).

#### Synthesis of intermediate 7

To a round-bottom flask, intermediate **6** (10.0 g, 25.2 mmol), 5%Pd/C (500 mg), and EtOH (50 ml) were added. The mixture was stirred under hydrogen (ballon) for 12.0 h at r.t., and then filtered. The filtrate was concentrated under reduced pressure and purified by using column chromatography to afford intermediate **7**. ^1^H NMR (400 MHz, CDCl_3_) *δ* 7.35 (td, *J*_1_ = 8.0 Hz, *J*_2_ = 6.0 Hz, 1H), 7.22-7.14 (m, 3H), 7.01 (td, *J*_1_ = 8.0 Hz, *J*_2_ = 2.0 Hz, 1H), 6.81-6.75 (m, 3H), 5.05 (s, 2H), 4.06 (d, *J* = 13.2 Hz, 2H), 2.62 (td, *J*_1_ = 13.2 Hz, *J*_2_ = 2.0 Hz, 2H), 2.50 (d, *J* = 7.2 Hz, 2H), 1.67-1.59 (m, 3H), 1.45 (s, 9H), 1.12 (m, 2H). ^13^C NMR (100 MHz, CDCl_3_) *δ* 164.23-161.78 (d, *J*_C-F_ = 244.8 Hz), 158.45, 154.87, 142.01, 139.78-139.71 (d, *J*_C-F_ = 7.3 Hz), 130.14-130.06 (d, *J*_C-F_ = 8.2 Hz), 129.27, 122.75-122.72 (d, *J*_C-F_ = 2.9 Hz), 122.08, 115.90, 114.86-114.65 (d, *J*_C-F_ = 21.0 Hz), 114.37-114.15 (d, *J*_C-F_ = 21.9 Hz), 112.06, 79.29, 69.10–69.08 (d, *J*_C-F_ = 1.9 Hz), 43.96, 43.15, 38.08 (2 C), 31.99 (2 C), 28.48 (3 C).

#### Synthesis of intermediate 8

To a round-bottom flask, intermediate **7** (3.0 g, 7.5 mmol) and anhydrous CH_2_Cl_2_ (40 ml) were added. Then, trifluoroacetic acid (5.6 ml, 75.4 mmol) was added. The mixture was stirred at r.t. for 5.0 h, and concentrated under reduced pressure. Saturated aqueous solution of Na_2_CO_3_ (50.0 ml) was added. The resulting mixture was extracted with ethyl acetate (50.0 ml × 3). The combined organic layers were washed successively with water (100.0 ml) and saturated brine (100.0 ml). After drying over anhydrous Na_2_SO_4_, the solution was concentrated under reduced pressure and purified by using column chromatography to afford intermediate **8**. ^1^H NMR (400 MHz, CDCl_3_) *δ* 7.35 (td, *J*_1_ = 8.0 Hz, *J*_2_ = 6.0 Hz, 1H), 7.22-7.15 (m, 3H), 7.01 (td, *J*_1_ = 8.0 Hz, *J*_2_ = 2.0 Hz, 1H),6.81-6.75 (m, 3H), 5.05 (s, 2H), 3.15 (d, *J* = 11.6 Hz, 2H), 2.59 (t, *J* = 12.4 Hz, 2H), 2.51 (d, *J* = 6.8 Hz, 2H), 1.68-1.59 (m, 3H), 1.32-1.22 (m, 2H). ^13^C NMR (100 MHz, CDCl_3_) *δ* 164.22-161.77 (d, *J*_C-F_ = 244.7 Hz), 158.46, 141.91, 139.78-139.70 (d, *J*_C-F_ = 7.4 Hz), 130.14-130.06 (d, *J*_C-F_ = 8.2 Hz), 129.28, 122.76-122.73 (d, *J*_C-F_ = 2.9 Hz), 122.08, 115.86, 114.86-114.64 (d, *J*_C-F_ = 21.1 Hz), 114.36-114.14 (d, *J*_C-F_ = 21.8 Hz), 112.09, 69.10-69.08 (d, *J*_C-F_ = 1.8 Hz), 45.91 (2 C), 43.42, 37.72, 32.04 (2 C).

#### Synthesis of compounds 9a ∼ c

To a round-bottom flask, intermediate **8** (60 mg, 0.2 mmol), corresponding substituted benzaldehydes (0.2 mmol), anhydrous CH_2_Cl_2_ (5 ml), and sodium triacetoxyborohydride (63.6 mg, 0.3 mmol) were added in success. The mixture was stirred at r.t. for 2.0 h, then 10% HCl aqueous solution (3.0 ml) was added to quench this reaction. The mixture was further basified by saturated aqueous solution of Na_2_CO_3_ (20.0 ml), and extracted with ethyl acetate (20.0 ml × 3). The combined organic layers were washed successively with water (50.0 ml) and saturated brine (50.0 ml). After drying over anhydrous Na_2_SO_4_, the solution was concentrated under reduced pressure and purified by using column chromatography to afford compounds **9a ∼ c**.

##### 1-benzyl-4-(3-((3-fluorobenzyl)oxy)benzyl)piperidine (9a)

White solid, mp 49.9-51.1 °C. ^1^H NMR (400 MHz, CDCl_3_) *δ* 7.36-7.13 (m, 9H), 6.98 (td, *J*_1_ = 8.4 Hz, *J*_2_ = 2.4 Hz, 1H), 6.78-6.73 (m, 3H), 5.02 (s, 2H), 3.47 (s, 2H), 2.85 (d, *J* = 11.6 Hz, 2H), 2.49 (d, *J* = 7.2 Hz, 2H), 1.89 (td, *J*_1_ = 11.6 Hz, *J*_2_ = 2.0 Hz, 2H), 1.58 (d, *J* = 13.2 Hz, 2H), 1.53-1.42 (m, 1H), 1.34-1.25 (m, 2H). ^13^C NMR (100 MHz, CDCl_3_) *δ* 164.21-161.76 (d, *J*_C-F_ = 244.7 Hz), 158.38, 142.55, 139.80-139.73 (d, *J*_C-F_ = 7.2 Hz), 138.23, 130.16-130.08 (d, *J*_C-F_ = 8.3 Hz), 129.34 (2 C), 129.19, 128.18 (2 C), 127.00, 122.81-122.78 (d, *J*_C-F_ = 2.8 Hz), 122.12, 115.83, 114.87-114.66 (d, *J*_C-F_ = 21.1 Hz), 114.40-114.18 (d, *J*_C-F_ = 21.9 Hz), 111.88, 69.06-69.04 (d, *J*_C-F_ = 1.9 Hz), 63.42, 53.78 (2 C), 43.23, 37.79, 32.10 (2 C). HRMS (ESI) m/z calcd. for C_26_H_28_FNO [M + H]^+^: 390.22332, found 390.22226.

##### 4-(3-((3-fluorobenzyl)oxy)benzyl)-1-(2-nitrobenzyl)piperidine (9b)

Yellow oil. ^1^H NMR (400 MHz, CDCl_3_) *δ* 7.80 (dd, *J*_1_ = 8.0 Hz, *J*_2_ = 1.2 Hz, 1H), 7.62 (d, *J* = 7.6 Hz, 1H), 7.52 (td, *J*_1_ = 7.6 Hz, *J*_2_ = 1.2 Hz, 1H), 7.39-7.29 (m, 2H), 7.20-7.14 (m, 3H), 6.99 (td, *J*_1_ = 8.4 Hz, *J*_2_ = 2.0 Hz, 1H), 6.71–6.74 (m, 3H), 5.04 (s, 2H), 3.74 (s, 2H), 2.62 (d, *J* = 10.8 Hz, 2H), 2.49 (d, *J* = 7.2 Hz, 2H), 1.96 (t, *J* = 11.6 Hz, 2H), 1.56 (t, *J* = 13.2 Hz, 2H), 1.51-1.44 (m, 1H), 1.31-1.19 (m, 2H). ^13^C NMR (100 MHz, CDCl_3_) *δ* 164.21-161.77 (d, *J*_C-F_ = 244.6 Hz), 158.40-158.38 (d, *J*_C-F_ = 1.9 Hz), 149.80, 142.52, 139.84-139.76 (d, *J*_C-F_ = 7.4 Hz), 134.59, 132.35, 130.87, 130.14-130.05 (d, *J*_C-F_ = 8.5 Hz), 129.18, 127.66, 124.28, 122.77-122.74 (d, *J*_C-F_ = 2.7 Hz), 122.08, 115.81-115.80 (d, *J*_C-F_ = 1.8 Hz), 114.84-114.62 (d, *J*_C-F_ = 21.7 Hz), 114.37-114.15 (d, *J*_C-F_ = 21.5 Hz), 111.98-111.95 (d, *J*_C-F_ = 2.6 Hz), 69.06, 59.23, 53.95 (2 C), 43.20, 37.71, 32.26 (2 C). HRMS (ESI) m/z calcd. for C_26_H_27_FN_2_O_3_ [M + H]^+^: 435.20840, found 435.20755.

##### 1-(4-chlorobenzyl)-4-(3-((3-fluorobenzyl)oxy)benzyl)piperidine (9c)

Slightly yellow solid, mp 51.4-52.0 °C. ^1^H NMR (400 MHz, CDCl_3_) *δ* 7.34 (td, *J*_1_ = 8.0 Hz, *J*_2_ = 5.6 Hz, 1H), 7,29-7.27 (m, 2H), 7.26-7.23 (m, 2H), 7.21–7.14 (m, 3H), 7.00 (td, *J*_1_ = 8.4 Hz, *J*_2_ = 2.8 Hz, 1H), 6.80-6.74 (m, 3H), 5.04 (s, 2H), 3.47 (s, 2H), 2.85 (d, *J* = 6.8 Hz, 2H), 1.92 (t, *J* = 10.8 Hz, 2H), 1.60 (d, *J* = 12.8 Hz, 2H), 1.52-1.47 (m, 1H), 1.36-1.30 (m, 2H). ^13^C NMR (100 MHz, CDCl_3_) *δ* 164.22-161.77 (d, *J*_C-F_ = 244.8 Hz), 158.42, 142.41, 139.81-139.74 (d, *J*_C-F_ = 7.4 Hz), 130.58 (2 C), 130.13-130.05 (d, *J*_C-F_ = 8.1 Hz), 129.20, 128.69, 128.34 (2 C), 128.28, 122.76-122.73 (d, *J*_C-F_ = 2.9 Hz), 122.09, 115.85, 114.84-114.63 (d, *J*_C-F_ = 21.0 Hz), 114.37-114.15 (d, *J*_C-F_ = 21.9 Hz), 111.94, 69.06, 62.45, 53.66 (2 C), 43.11, 37.67, 31.94 (2 C). HRMS (ESI) m/z calcd. for C_26_H_27_ClFNO [M + H]^+^: 424.18435, found 424.18360.

#### Synthesis of intermediates 11 and 17

To a round-bottom flask, 3-hydroxybenzoic acid or 4-hydroxybenzoic acid (5.6 g, 40.5 mmol), KOH (2.3 g, 41.0 mmol), and EtOH (30.0 ml) were added. The mixture was refluxed at argon atmosphere for 30.0 min, and then KI (332.0 mg, 2.0 mmol) and 1-(chloromethyl)-3-fluorobenzene (5.0 ml, 41.2 mmol) was added slowly. The mixture was refluxed for 12.0 h, and then acidized by 10% HCl aqueous solution at 0 ∼ 5 °C to pH = 1 ∼ 2. The resulting mixture was extracted with ethyl acetate (50 ml × 3). The combined organic layers were washed successively with water (100.0 ml) and saturated brine (100.0 ml). After drying over anhydrous Na_2_SO_4_, the solution was concentrated under reduced pressure to afford intermediates **11** and **17**.

#### Synthesis of compounds 13 ∼ 15 and 19 ∼ 22

To a round-bottom flask, intermediates **11** and **17** (123.0 mg, 0.5 mmol), anhydrous CH_2_Cl_2_ (5.0 ml), catalytic amount DMF and SOCl_2_ (43.6 μL, 0.6 mmol) were added in success. The mixture was refluxed at argon atmosphere for 12.0 h, and then corresponding tertiary amine alkyl-1-ols (1.0 mmol) was added. The mixture was stirred at r.t. for another 12.0 h, and then quenched by saturated aqueous solution of Na_2_CO_3_ (20.0 ml). The mixture was extracted with ethyl acetate (20.0 ml × 3), and the combined organic layers were washed successively with water (50.0 ml) and saturated brine (50.0 ml). After drying over anhydrous Na_2_SO_4_, the solution was concentrated under reduced pressure and purified by using column chromatography to afford compounds **13**∼**15** and **19** ∼**22**.

##### (1-benzylpiperidin-4-yl)methyl 3-((3-fluorobenzyl)oxy)benzoate (13)

White solid, mp 43.3-54.1 °C. ^1^H NMR (400 MHz, CDCl_3_) *δ* 7.66 (dt, *J*_1_ = 7.6 Hz, *J*_2_ = 1.2 Hz, 1H), 7.61 (dd, *J*_1_ = 2.4 Hz, *J*_2_ = 1.2 Hz, 1H), 7.37-7.24 (m, 7H), 7.20 (d, *J* = 7.6 Hz, 1H), 7.18-7.14 (m, 2H), 7.01 (td, *J*_1_ = 8.4 Hz, *J*_2_ = 2.0 Hz, 1H), 5.01 (s, 2H), 4.17 (d, *J* = 6.0 Hz, 2H), 3.52 (s, 2H), 2.93 (d, *J* = 11.2 Hz, 2H), 2.00 (t, *J* = 11.2 Hz, 2H), 1.76 (d, *J* = 13.2 Hz, 2H), 1.71-1.59 (m, 1H), 1.48-1.37 (m, 2H). ^13^C NMR (100 MHz, CDCl_3_) *δ* 166.33, 164.22-161.77 (d, *J*_C-F_ = 245.0 Hz), 158.39, 139.18-139.11 (d, *J*_C-F_ = 7.2 Hz), 131.72, 130.27-130.19 (d, *J*_C-F_ = 8.3 Hz), 129.55 (2 C), 129.36, 128.27 (2 C), 127.17, 122.83-122.80 (d, *J*_C-F_ = 3.0 Hz), 122.46, 120.01, 115.13, 115.10-114.89 (d, *J*_C-F_ = 21.0 Hz), 114.41-114.19 (d, *J*_C-F_ = 22.0 Hz), 69.33, 69.31, 63.31, 53.16 (2 C), 35.38, 28.81 (2 C). HRMS (ESI) m/z calcd. for C_27_H_28_FNO_3_ [M + H]^+^: 434.21315, found 434.21234.

##### 2-(Dimethylamino)ethyl 3-((3-fluorobenzyl)oxy)benzoate (14a)

White solid, mp 42.2-42.5 °C.^1^H NMR (400 MHz, CDCl_3_) *δ* 7.67 (d, *J* = 8.0 Hz, 1H), 7.64-7.63 (m, 1H), 7.38-7.32 (m, 2H), 7.20 (d, *J* = 8.0 Hz, 1H), 7.18-7.14 (m, 2H), 7.02 (td, *J*_1_ = 8.4 Hz, *J*_2_ = 2.4 Hz, 1H), 5.10 (s, 2H), 4.43 (t, *J* = 6.0 Hz, 2H), 2.72 (t, *J* = 6.0 Hz, 2H), 2.34 (s, 6H). ^13^C NMR (100 MHz, CDCl_3_) *δ* 166.35, 164.21-161.76 (d, *J*_C-F_ = 244.8 Hz), 158.38, 139.18-139.11 (d, *J*_C-F_ = 7.3 Hz), 131.55, 130.26-130.18 (d, *J*_C-F_ = 8.1 Hz), 129.54, 122.82-122.79 (d, *J*_C-F_ = 2.9 Hz), 122.57, 120.14, 115.15, 115.09-114.88 (d, *J*_C-F_ = 21.1 Hz), 114.40-114.18 (d, *J*_C-F_ = 22.0 Hz), 69.30-69.28 (d, *J*_C-F_ = 1.9 Hz), 63.14, 57.76, 45.85 (2 C). HRMS (ESI) m/z calcd. for C_18_H_20_FNO_3_ [M + H]^+^: 318.15055, found 318.14993.

##### 3-(Dimethylamino)propyl 3-((3-fluorobenzyl)oxy)benzoate (15a)

Slightly yellow oil. ^1^H NMR (400 MHz, CDCl_3_) *δ* 7.66 (d, *J* = 8.0 Hz, 1H), 7.64-7.63 (m, 1H), 7.37-7.32 (m, 2H), 7.20 (d, *J* = 8.0 Hz, 1H), 7.17–7.15 (m, 2H), 7.01 (td, *J*_1_ = 8.4 Hz, *J*_2_ = 2.0 Hz, 1H), 5.10 (s, 2H), 4.37 (t, *J* = 6.8 Hz, 2H), 2.43 (t, *J* = 7.2 Hz, 2H), 2.26 (s, 6H), 1.94 (p, *J* = 6.8 Hz, 2H). ^13^C NMR (100 MHz, CDCl_3_) *δ* 166.31, 164.21-161.77 (d, *J*_C-F_ = 244.8 Hz), 158.42, 139.22-139.15 (d, *J*_C-F_ = 7.3 Hz), 131.80, 130.23-130.15 (d, *J*_C-F_ = 8.1 Hz), 129.51, 122.79-122.76 (d, *J*_C-F_ = 3.0 Hz), 122.44, 120.01, 115.13, 115.05-114.84 (d, *J*_C-F_ = 21.0 Hz), 114.37-114.15 (d, *J*_C-F_ = 22.0 Hz), 69.31-69.29 (d, *J*_C-F_ = 1.9 Hz), 63.51, 56.30, 45.47 (2 C), 27.04. HRMS (ESI) m/z calcd. for C_19_H_22_FNO_3_ [M + H]^+^: 332.16620, found 332.16538.

##### (1-benzylpiperidin-4-yl)methyl 4-((3-fluorobenzyl)oxy)benzoate (19)

White solid, mp 82.0-83.2 °C. ^1^H NMR (400 MHz, CDCl_3_) *δ* 7.99 (d, *J* = 8.8 Hz, 2H), 7.36 (td, *J*_1_ = 8.0 Hz, *J*_2_ = 6.0 Hz, 1H), 7.32-7.24 (m, 5H), 7.19 (d, *J* = 7.6 Hz, 1H), 7.15 (d, *J* = 9.6 Hz, 1H), 7.03 (td, *J*_1_ = 8.4 Hz, *J*_2_ = 2.4 Hz, 1H), 6.97 (d, *J* = 8.8 Hz, 2H), 5.11 (s, 2H), 4.15 (d, *J* = 6.4 Hz, 2H), 3.53 (s, 2H), 2.94 (d, *J* = 11.6 Hz, 2H), 2.00 (t, *J* = 10.4 Hz, 2H), 1.76 (d, *J* = 12.0 Hz, 2H), 1.49-1.39 (m, 2H), 1.35-1.27 (m, 1H). ^13^C NMR (100 MHz, CDCl_3_) *δ* 166.25, 164.24-161.78 (d, *J*_C-F_ = 245.2 Hz), 162.12, 138.89-138.82 (d, *J*_C-F_ = 7.3 Hz), 138.18, 131.65 (2 C), 130.31-130.22 (d, *J*_C-F_ = 8.1 Hz), 129.26 (2 C), 128.20 (2 C), 127.03, 123.30, 122.74-122.71 (d, *J*_C-F_ = 2.9 Hz), 115.19-114.98 (d, *J*_C-F_ = 21.0 Hz), 114.43 (2 C), 114.36-114.14 (d, *J*_C-F_ = 23.1 Hz), 69.24-69.22 (d, *J*_C-F_ = 1.9 Hz), 69.04, 63.34, 53.20 (2 C), 35.52, 28.95 (2 C). HRMS (ESI) m/z calcd. for C_27_H_28_FNO_3_ [M + H]^+^: 434.21315, found 434.21238.

##### 2-(Dimethylamino)ethyl 4-((3-fluorobenzyl)oxy)benzoate (20a)

White solid, mp 42.9-43.4 °C. ^1^H NMR (400 MHz, CDCl_3_) *δ* 7.91 (d, *J* = 8.8 Hz, 2H), 7.25 (td, *J*_1_ = 8.0 Hz, *J*_2_ = 6.0 Hz, 1H), 7.09 (d, *J* = 8.0 Hz, 1H), 7.04 (d, *J* = 9.2 Hz, 1H), 6.92 (td, *J*_1_ = 8.4 Hz, *J*_2_ = 2.4 Hz, 1H), 6.87 (d, *J* = 8.8 Hz, 2H), 5.00 (s, 2H), 4.31 (t, *J* = 6.0 Hz, 2H), 2.62 (t, *J* = 6.0 Hz, 2H), 2.25 (s, 6H). ^13^C NMR (100 MHz, CDCl_3_) *δ* 166.17, 164.21-161.75 (d, *J*_C-F_ = 245.1 Hz), 162.17, 138.91-138.84 (d, *J*_C-F_ = 7.3 Hz), 131.73 (2 C), 130.72-130.19 (d, *J*_C-F_ = 8.1 Hz), 123.12, 122.72-122.69 (d, *J*_C-F_ = 2.9 Hz), 115.13-114.92 (d, *J*_C-F_ = 21.0 Hz), 114.42 (2 C), 114.32-114.10 (d, *J*_C-F_ = 21.9 Hz), 69.20-69.18 (d, *J*_C-F_ = 1.8 Hz), 62.68, 57.82, 45.75 (2 C). HRMS (ESI) m/z calcd. for C_18_H_20_FNO_3_ [M + H]^+^: 318.15055, found 318.14970.

##### 2-(Pyrrolidin-1-yl)ethyl 4-((3-fluorobenzyl)oxy)benzoate (20c)

Yellow solid, mp 46.8-47.2 °C. ^1^H NMR (400 MHz, CDCl_3_) *δ* 8.01 (d, *J* = 8.8 Hz, 2H), 7.34 (td, *J*_1_ = 8.0 Hz, *J*_2_ = 6.0 Hz, 1H), 7.18 (d, *J* = 7.6 Hz, 1H), 7.14 (d, *J* = 9.6 Hz, 1H), 7.02 (td, *J*_1_ = 8.4 Hz, *J*_2_ = 2.4 Hz, 1H), 6.97 (d, *J* = 8.8 Hz, 2H), 5.10 (s, 2H), 4.44 (t, *J* = 6.0 Hz, 2H), 2.87 (t, *J* = 6.0 Hz, 2H), 2.63 (t, *J* = 6.8 Hz, 4H), 1.80 (p, *J* = 6.8 Hz, 4H). ^13^C NMR (100 MHz, CDCl_3_) *δ* 166.14, 164.21-161.76 (d, *J*_C-F_ = 245.1 Hz), 162.15, 138.90-138.82 (d, *J*_C-F_ = 7.3 Hz), 131.72 (2 C), 130.29-130.21 (d, *J*_C-F_ = 8.1 Hz), 123.17, 122.73-122.70 (d, *J*_C-F_ = 2.9 Hz), 115.15-114.94 (d, *J*_C-F_ = 21.0 Hz), 114.43 (2 C), 114.33-114.11 (d, *J*_C-F_ = 22.1 Hz), 69.20-69.18 (d, *J*_C-F_ = 2.1 Hz), 63.99, 54.72 (2 C), 54.54, 23.55 (2 C). HRMS (ESI) m/z calcd. for C_20_H_22_FNO_3_ [M + H]^+^: 344.16620, found 344.16549.

##### 2-(Piperidin-1-yl)ethyl 4-((3-fluorobenzyl)oxy)benzoate (20d)

Yellow solid, mp 41.5-41.8 °C. ^1^H NMR (400 MHz, CDCl_3_) *δ* 8.00 (d, *J* = 8.8 Hz, 2H), 7.36 (td, *J*_1_ = 8.0 Hz, *J*_2_ = 6.0 Hz, 1H), 7.19 (d, *J* = 7.6 Hz, 1H), 7.15 (d, *J* = 9.6 Hz, 1H), 7.03 (td, *J*_1_ = 8.4 Hz, *J*_2_ = 2.4 Hz, 1H), 6.98 (d, *J* = 8.8 Hz, 2H), 5.12 (s, 2H), 4.44 (t, *J* = 6.0 Hz, 2H), 2.76 (t, *J* = 6.0 Hz, 2H), 2.53 (brs, 4H), 1.61 (p, *J* = 5.6 Hz, 4H), 1.47-1.42 (m, 2H). ^13^C NMR (100 MHz, CDCl_3_) *δ* 166.14, 164.22-161.77 (d, *J*_C-F_ = 245.0 Hz), 162.16, 138.85-138.77 (d, *J*_C-F_ = 7.3 Hz), 131.72 (2 C), 130.33-130.24 (d, *J*_C-F_ = 8.3 Hz), 123.11, 122.76-122.73 (d, *J*_C-F_ = 3.1 Hz), 115.21-115.00 (d, *J*_C-F_ = 21.0 Hz), 114.44 (2 C), 114.37-114.15 (d, *J*_C-F_ = 22.1 Hz), 69.23-69.21 (d, *J*_C-F_ = 2.1 Hz), 62.45, 57.34, 54.77 (2 C), 25.81 (2 C), 24.04. HRMS (ESI) m/z calcd. for C_21_H_24_FNO_3_ [M + H]^+^: 358.18185, found 358.18114.

##### 3-(Dimethylamino)propyl 4-((3-fluorobenzyl)oxy)benzoate (21a)

Slightly yellow oil. ^1^H NMR (400 MHz, CDCl_3_) *δ* 8.00 (d, *J* = 8.8 Hz, 2H), 7.36 (td, *J*_1_ = 8.0 Hz, *J*_2_ = 6.0 Hz, 1H), 7.19 (d, *J* = 7.6 Hz, 1H), 7.15 (d, *J* = 9.2 Hz, 1H), 7.03 (td, *J*_1_ = 8.4 Hz, *J*_2_ = 2.0 Hz, 1H), 6.98 (d, *J* = 8.8 Hz, 2H), 5.11 (s, 2H), 4.34 (t, *J* = 6.4 Hz, 2H), 2.47 (t, *J* = 8.0 Hz, 2H), 2.29 (s, 6H), 1.96 (p, *J* = 8.0 Hz, 2H). ^13^C NMR (100 MHz, CDCl_3_) *δ* 166.22, 164.23–161.78 (d, *J*_C-F_ = 245.0 Hz), 162.13, 138.89-138.82 (d, *J*_C-F_ = 7.2 Hz), 131.64 (2 C), 130.30-130.22 (d, *J*_C-F_ = 8.1 Hz), 123.24, 122.74-122.71 (d, *J*_C-F_ = 3.0 Hz), 115.18-114.96 (d, *J*_C-F_ = 21.0 Hz), 114.44 (2 C), 114.35-114.13 (d, *J*_C-F_ = 22.0 Hz), 69.24-69.22 (d, *J*_C-F_ = 2.0 Hz), 63.01, 56.29, 45.30 (2 C), 26.94. HRMS (ESI) m/z calcd. for C_19_H_22_FNO_3_ [M + H]^+^: 332.16620, found 332.16541.

##### 3-(Pyrrolidin-1-yl)propyl 4-((3-fluorobenzyl)oxy)benzoate (21c)

Yellow oil. ^1^H NMR (400 MHz, CDCl_3_) *δ* 8.00 (d, *J* = 8.8 Hz, 2H), 7.36 (td, *J*_1_ = 8.0 Hz, *J*_2_ = 5.6 Hz, 1H), 7.19 (d, *J* = 7.6 Hz, 1H), 7.15 (d, *J* = 9.2 Hz, 1H), 7.03 (td, *J*_1_ = 8.4 Hz, *J*_2_ = 2.4 Hz, 1H), 6.98 (d, *J* = 8.8 Hz, 2H), 5.11 (s, 2H), 4.35 (t, *J* = 6.4 Hz, 2H), 2.62 (t, *J* = 7.6 Hz, 2H), 2.55 (t, *J* = 6.4 Hz, 4H), 2.00 (p, *J* = 6.4 Hz, 2H), 1.80 (p, *J* = 3.2 Hz, 4H). ^13^C NMR (100 MHz, CDCl_3_) *δ* 166.26, 164.21-161.76 (d, *J*_C-F_ = 245.1 Hz), 162.10, 138.87-138.79 (d, *J*_C-F_ = 7.3 Hz), 131.66 (2 C), 130.32-130.24 (d, *J*_C-F_ = 8.1 Hz), 123.23, 122.76-122.73 (d, *J*_C-F_ = 2.9 Hz), 115.19-114.98 (d, *J*_C-F_ = 21.0 Hz), 114.41 (2 C), 114.36-114.14 (d, *J*_C-F_ = 22.1 Hz), 69.21-69.19 (d, *J*_C-F_ = 2.0 Hz), 63.21, 54.23 (2 C), 53.16, 28.30, 23.44 (2 C). HRMS (ESI) m/z calcd. for C_21_H_24_FNO_3_ [M + H]^+^: 358.18185, found 358.18115.

##### 3-(Piperidin-1-yl)propyl 4-((3-fluorobenzyl)oxy)benzoate (21d)

Yellow oil. ^1^H NMR (400 MHz, CDCl_3_) *δ* 7.99 (d, *J* = 8.8 Hz, 2H), 7.35 (td, *J*_1_ = 8.0 Hz, *J*_2_ = 6.0 Hz, 1H), 7.19 (d, *J* = 7.6 Hz, 1H), 7.15 (d, *J* = 10.0 Hz, 1H), 7.02 (td, *J*_1_ = 8.4 Hz, *J*_2_ = 2.0 Hz, 1H), 6.97 (d, *J* = 8.8 Hz, 2H), 5.10 (s, 2H), 4.33 (t, *J* = 6.4 Hz, 2H), 2.49-2.42 (m, 6H), 1.97 (p, *J* = 6.4 Hz, 2H), 1.60 (p, *J* = 5.2 Hz, 4H), 1.47-1.42 (m, 2H). ^13^C NMR (100 MHz, CDCl_3_) *δ* 166.22, 164.22-161.77 (d, *J*_C-F_ = 245.0 Hz), 162.15, 138.88-138.81 (d, *J*_C-F_ = 7.3 Hz), 131.66 (2 C), 130.30-130.22 (d, *J*_C-F_ = 8.2 Hz), 123.18, 122.74-122.71 (d, *J*_C-F_ = 2.9 Hz), 115.17-114.96 (d, *J*_C-F_ = 20.9 Hz), 114.44 (2 C), 114.34-114.12 (d, *J*_C-F_ = 22.0 Hz), 69.23-69.21 (d, *J*_C-F_ = 1.8 Hz), 63.14, 55.73, 54.33 (2 C), 25.89, 25.37 (2 C), 24.04. HRMS (ESI) m/z calcd. for C_22_H_26_FNO_3_ [M + H]^+^: 372.19750, found 372.19668.

##### 4-(Dimethylamino)butyl 4-((3-fluorobenzyl)oxy)benzoate (22a)

Slightly yellow oil. ^1^H NMR (400 MHz, CDCl_3_) *δ* 7.99 (d, *J* = 8.8 Hz, 2H), 7.35 (td, *J*_1_ = 8.0 Hz, *J*_2_ = 5.6 Hz, 1H), 7.19 (d, *J* = 8.0 Hz, 1H), 7.15 (d, *J* = 9.6 Hz, 1H), 7.02 (td, *J*_1_ = 8.8 Hz, *J*_2_ = 2.0 Hz, 1H), 6.97 (d, *J* = 8.8 Hz, 2H), 5.11 (s, 2H), 4.31 (t, *J* = 6.4 Hz, 2H), 2.41 (t, *J* = 7.6 Hz, 2H), 2.30 (s, 6H), 1.79 (p, *J* = 6.4 Hz, 2H), 1.70-1.62 (m, 2H). ^13^C NMR (100 MHz, CDCl_3_) *δ* 166.30, 164.20-161.75 (d, *J*_C-F_ = 245.1 Hz), 162.09, 138.86-138.79 (d, *J*_C-F_ = 7.2 Hz), 131.64 (2 C), 130.31-130.23 (d, *J*_C-F_ = 8.2 Hz), 123.24, 122.77-122.74 (d, *J*_C-F_ = 3.0 Hz), 115.18-114.97 (d, *J*_C-F_ = 20.9 Hz), 114.40 (2 C), 114.36-114.14 (d, *J*_C-F_ = 22.0 Hz), 69.20-69.18 (d, *J*_C-F_ = 2.0 Hz), 64.47, 59.02, 45.10 (2 C), 25.64, 23.91. HRMS (ESI) m/z calcd. for C_20_H_24_FNO_3_ [M + H]^+^: 346.18185, found 346.18111.

#### Synthesis of compounds 23 ∼ 25

To a round-bottom flask, intermediate **17** (123.0 mg, 0.5 mmol), EDCI (153.0 mg, 0.8 mmol), HOBT (108.0 mg, 0.8 mmol), and THF (5.0 ml) were added in success. The mixture was stirred at r.t. for 30.0 min, and then corresponding tertiary amine alkyl diamines (0.6 mmol) were added. The reaction was kept in r.t. for 12.0 h, and then the solvent was removed under reduced pressure. Saturated aqueous solution of Na_2_CO_3_ (30.0 ml) was added to the residues, and the mixture was extracted with ethyl acetate (20.0 ml × 3). The combined organic layers were washed successively with water (50.0 ml) and saturated brine (50.0 ml). After drying over anhydrous Na_2_SO_4_, the solution was concentrated under reduced pressure and purified by using column chromatography to afford compounds **23**∼**25**.

##### 4-((3-fluorobenzyl)oxy)-N-(2-(pyrrolidin-1-yl)ethyl)benzamide (23c)

White solid, mp 88.5-88.6 °C. ^1^H NMR (400 MHz, CDCl_3_) *δ* 7.79 (d, *J* = 8.8 Hz, 2H), 7.34 (td, *J*_1_ = 8.0 Hz, *J*_2_ = 6.0 Hz, 1H), 7.18 (d, *J* = 8.0 Hz, 1H), 7.14 (d, *J* = 9.2 Hz, 1H), 7.05-7.01 (m, 1H), 6.97 (d, *J* = 8.8 Hz, 2H), 5.08 (s, 2H), 3.55 (q, *J* = 5.6 Hz, 2H), 2.72 (t, *J* = 5.6 Hz, 2H), 2.59 (brs, 4H), 1.80 (brs, 4H). ^13^C NMR (100 MHz, CDCl_3_) *δ* 166.98, 164.20-161.75 (d, *J*_C-F_ = 244.7 Hz), 160.90, 139.10-139.03 (d, *J*_C-F_ = 7.3 Hz), 130.26-130.18 (d, *J*_C-F_ = 8.2 Hz), 128.95 (2 C), 127.44, 122.73-122.70 (d, *J*_C-F_ = 2.9 Hz), 115.07-114.87 (d, *J*_C-F_ = 20.8 Hz), 114.51 (2 C), 114.30-114.08 (d, *J*_C-F_ = 22.1 Hz), 69.19-69.17 (d, *J*_C-F_ = 2.1 Hz), 54.84, 53.94 (2 C), 38.39, 23.48 (2 C). HRMS (ESI) m/z calcd. for C_20_H_23_FN_2_O_2_ [M + H]^+^: 343.18218, found 343.18146.

##### N-(3-(dimethylamino)propyl)-4-((3-fluorobenzyl)oxy)benzamide (24a)

White solid, mp 90.7-91.8 °C. ^1^H NMR (400 MHz, CDCl_3_) *δ* 8.29 (brs, 1H), 7.75 (d, *J* = 8.8 Hz, 2H), 7.35 (td, *J*_1_ = 8.0 Hz, *J*_2_ = 6.0 Hz, 1H), 7.19 (d, *J* = 7.6 Hz, 1H), 7.15 (d, *J* = 9.6 Hz, 1H), 7.02 (td, *J*_1_ = 8.4 Hz, *J*_2_ = 2.0 Hz, 1H), 6.98 (d, *J* = 8.8 Hz, 2H), 5.09 (s, 2H), 3.54 (q, *J* = 6.0 Hz, 2H), 2.50 (t, *J* = 6.0 Hz, 2H), 2.30 (s, 6H), 1.77 (p, *J* = 6.0 Hz, 2H). ^13^C NMR (100 MHz, CDCl_3_) *δ* 166.54, 164.20-161.75 (d, *J*_C-F_ = 244.8 Hz), 160.70, 139.11-139.04 (d, *J*_C-F_ = 7.4 Hz), 130.28-130.19 (d, *J*_C-F_ = 8.3 Hz), 128.69 (2 C), 127.65, 122.75–122.72 (d, *J*_C-F_ = 2.9 Hz), 115.10-114.89 (d, *J*_C-F_ = 21.0 Hz), 114.47 (2 C), 114.334-114.115 (d, *J*_C-F_ = 21.9 Hz), 69.17-69.15 (d, *J*_C-F_ = 2.0 Hz), 59.26, 45.40 (2 C), 40.44, 25.28. HRMS (ESI) m/z calcd. for C_19_H_23_FN_2_O_2_ [M + H]^+^: 331.18218, found 331.18138.

##### N-(3-(diethylamino)propyl)-4-((3-fluorobenzyl)oxy)benzamide (24b)

White solid, mp 47.3-48.1 °C. ^1^H NMR (400 MHz, CDCl_3_) *δ* 8.56 (brs, 1H), 7.78 (d, *J* = 8.8 Hz, 2H), 7.35 (td, *J*_1_ = 8.0 Hz, *J*_2_ = 5.6 Hz, 1H), 7.19 (d, *J* = 8.0 Hz, 1H), 7.15 (d, *J* = 9.2 Hz, 1H), 7.02 (td, *J*_1_ = 8.4 Hz, *J*_2_ = 2.4 Hz, 1H), 6.97 (d, *J* = 8.8 Hz, 2H), 5.09 (s, 2H), 3.55 (q, *J* = 5.6 Hz, 2H), 2.66-2.58 (m, 6H), 1.78 (p, *J* = 6.0 Hz, 2H), 1.06 (t, *J* = 7.2 Hz, 6H). ^13^C NMR (100 MHz, CDCl_3_) *δ* 166.61, 164.22–161.75 (d, *J*_C-F_ = 244.8 Hz), 160.70, 139.11-139.04 (d, *J*_C-F_ = 7.4 Hz), 130.26-130.18 (d, *J*_C-F_ = 8.3 Hz), 128.80 (2 C), 127.63, 122.77-122.74 (d, *J*_C-F_ = 3.0 Hz), 115.09-114.88 (d, *J*_C-F_ = 21.0 Hz), 114.37 (2 C), 114.14, 69.17-69.15 (d, *J*_C-F_ = 1.9 Hz), 52.98, 46.71 (2 C), 40.74, 24.68, 11.20 (2 C). HRMS (ESI) m/z calcd. for C_21_H_27_FN_2_O_2_ [M + H]^+^: 359.21348, found 359.21254.

##### N-(4-(diethylamino)butyl)-4-((3-fluorobenzyl)oxy)benzamide (25b)

White solid, mp 43.5-44.4 °C. ^1^H NMR (400 MHz, CDCl_3_) *δ* 7.74 (d, *J* = 8.8 Hz, 2H), 7.35 (td, *J*_1_ = 8.0 Hz, *J*_2_ = 6.0 Hz, 1H), 7.19 (d, *J* = 8.0 Hz, 1H), 7.15 (d, *J* = 9.6 Hz, 1H), 7.02 (td, *J*_1_ = 8.8 Hz, *J*_2_ = 2.0 Hz, 1H), 6.97 (d, *J* = 8.8 Hz, 2H), 5.10 (s, 2H), 3.44 (q, *J* = 6.4 Hz, 2H), 2.54 (q, *J* = 7.2 Hz, 4H), 2.47 (t, *J* = 6.8 Hz, 2H), 1.69-1.55 (m, 4H), 1.00 (t, *J* = 7.2 Hz, 6H). ^13^C NMR (100 MHz, CDCl_3_) *δ* 167.72, 164.21-161.76 (d, *J*_C-F_ = 244.6 Hz), 160.74, 139.05-138.98 (d, *J*_C-F_ = 7.5 Hz), 130.28-130.19 (d, *J*_C-F_ = 8.2 Hz), 128.83 (2 C), 127.83, 122.77-122.74 (d, *J*_C-F_ = 3.0 Hz), 115.12-114.91 (d, *J*_C-F_ = 21.0 Hz), 114.45 (2 C), 114.36–114.14 (d, *J*_C-F_ = 21.9 Hz), 69.18, 52.27, 46.59 (2 C), 39.88, 27.62, 24.73, 10.96 (2 C). HRMS (ESI) m/z calcd. for C_22_H_29_FN_2_O_2_ [M + H]^+^: 373.22913, found 373.22847.

#### Synthesis of compounds 27

To a round-bottom flask, 2,5-dihydroxy-1,4-dithiane (304.5 mg, 2.0 mmol), pyrrolidine (197.1 μL, 2.4 mmol), anhydrous CH_2_Cl_2_ (10 ml), and sodium triacetoxyborohydride (635.8 mg, 3.0 mmol) were added in success. The mixture was stirred at r.t. for 2.0 h, then 10% HCl aqueous solution (6.0 ml) was added to quench this reaction. The mixture was further basified by saturated aqueous solution of Na_2_CO_3_ (20.0 ml), and extracted with ethyl acetate (20.0 ml × 3). The combined organic layers were washed successively with water (50.0 ml) and saturated brine (50.0 ml). After drying over anhydrous Na_2_SO_4_, the solution was concentrated under reduced pressure to afford crude intermediate **27**.

#### Synthesis of compounds 28c

To a round-bottom flask, intermediate **17** (123.0 mg, 0.5 mmol), EDCI (153.0 mg, 0.8 mmol), HOBT (108.0 mg, 0.8 mmol), and THF (5.0 ml) were added in success. The mixture was stirred at r.t. for 30.0 min, and then intermediate **27** (0.6 mmol) was added. The reaction was kept in r.t. for 12.0 h, and then the solvent was removed under reduced pressure. Saturated aqueous solution of Na_2_CO_3_ (30.0 ml) was added to the residues, and the mixture was extracted with ethyl acetate (20.0 ml × 3). The combined organic layers were washed successively with water (50.0 ml) and saturated brine (50.0 ml). After drying over anhydrous Na_2_SO_4_, the solution was concentrated under reduced pressure and purified by using column chromatography to afford compound **28c**.

##### S-(2-(pyrrolidin-1-yl)ethyl) 4-((3-fluorobenzyl)oxy)benzothioate (28c)

Grey solid, mp 56.4-56.9 °C. ^1^H NMR (400 MHz, CDCl_3_) *δ* 7.96 (d, *J* = 8.8 Hz, 2H), 7.36 (td, *J*_1_ = 8.0 Hz, *J*_2_ = 6.0 Hz, 1H), 7.19 (d, *J* = 8.0 Hz, 1H), 5.07.15 (d, *J* = 9.6 Hz, 1H), 7.03 (td, *J*_1_ = 8.4 Hz, *J*_2_ = 2.4 Hz, 1H), 6.98 (d, *J* = 8.8 Hz, 2H), 5.12 (s, 2H), 3.23 (t, *J* = 7.2 Hz, 2H), 2.76 (t, *J* = 7.2 Hz, 2H), 2.64 (t, *J* = 6.4 Hz, 4H), 1.82 (p. *J* = 6.0 Hz, 4H). ^13^C NMR (100 MHz, CDCl_3_) *δ* 190.36, 164.22-161.77 (d, *J*_C-F_ = 245.0 Hz), 162.53, 138.73-138.66 (d, *J*_C-F_ = 7.3 Hz), 131.67, 130.33-130.24 (d, *J*_C-F_ = 8.3 Hz), 129.47 (2 C), 122.74-122.72 (d, *J*_C-F_ = 2.9 Hz), 115.24-115.03 (d, *J*_C-F_ = 20.9 Hz), 114.56 (2 C), 114.36–114.13 (d, *J*_C-F_ = 22.1 Hz), 69.31-69.29 (d, *J*_C-F_ = 2.0 Hz), 55.60, 54.01 (2 C), 27.71, 23.51 (2 C). HRMS (ESI) m/z calcd. for C_20_H_22_FNO_2_S [M + H]^+^: 360.14335, found 360.14257.

### Pharmacology

#### Animals

The study received ethical approval from the Institutional Animal Care and Use Committee of North Sichuan Medical College (Approval No. 2024-014), and all experiments were performed in accordance with the ARRIVE guidelines. Adult male C57BL/6J mice were sourced from the Laboratory Animal Centre of North Sichuan Medical College (Certification No. SYXK-Sichuan 2018–76). The animals were maintained under controlled conditions: temperature was kept at 22–24 °C, humidity at 50–60%, and a 12-h light/dark cycle was implemented. Food and water were provided *ad libitum* throughout the study. Mice were anaesthetised via intraperitoneal injection of pentobarbital sodium (300 mg/kg) prior to euthanasia by cervical dislocation.

#### Inhibition experiments of MAOs and ChE

Human MAO-A and MAO-B were obtained from Sigma-Aldrich, while kynuramine was sourced from Aladdin Biochemical Technology Co., Ltd. In the assay, 10 μL of test compound solution and 30 μL of either MAO-A or MAO-B (12.5 μg/mL in PBS) were introduced into a black 96-well plate. The mixture was incubated at 37 °C for 30 min, followed by the addition of 10 μL of kynuramine (150 μM in PBS). After further incubation at 37 °C for 30 min, the reaction was quenched with 40 μL of 2 M NaOH aqueous solution and 100 μL of water. Fluorescence was measured using a multifunctional enzyme marker (Thermo Scientific) at excitation and emission wavelengths of 310 nm and 400 nm, respectively. The percentage inhibition was calculated according to the formula: 100-(IF_i_-IF_0_)/(IF_c_-IF_0_)*100. If the inhibition exceeded 50% at 10 μM, the IC_50_ value was subsequently determined.

Human AChE was obtained from Sigma-Aldrich, while BuChE was sourced from rat serum (Baidi Biotech Ltd. F835-050), which further calibrated using rivastigmine (Adamas life, 19279B). Add the following reagents sequentially to a 96-well plate: 40.0 μL of PBS (50.0 mM, pH 8.0), 330.0 μL of acetylthiocholine iodide (J&K Scientific, R10L15) solution (1.0 mM in PBS), 20.0 μL of test compound solution (prepared from a 2.5 mM DMSO stock and diluted to the desired concentration with PBS), and 10.0 μL of *hu*AChE solution (0.05 U/mL in PBS). After the addition of all components, incubate the plate at 37 °C for 15 min. Then, add 30.0 μL of DTNB (5,5′-dithiobis-(2-nitrobenzoic acid), Adamas life, 77668 G) solution (0.2% m/v in PBS), and incubate the plate at 37 °C for 45.0 min, then measure the absorbance of each well at 412 nm using a Thermo Scientific microplate reader. For the blank control, replace the test compound solution with 20.0 μL of PBS buffer. The percentage inhibition of cholinesterase by the compound at 10 μM is calculated using the formula: % inhibition = (1-OD_sample_/OD_blank_) × 100%. If the inhibition exceeds 50%, further dilute the compound and measure its inhibition at multiple concentrations. The IC_50_ value against AChE is then determined by linear regression of the logarithm of compound concentration versus the percentage inhibition. The assay for measuring BuChE inhibitory activity is very similar to that used for evaluating AChE inhibition, with the main differences being the substitution of AChE with BuChE and the replacement of acetylthiocholine iodide with butyrylthiocholine iodide (TCI (Shanghai) Development Co., Ltd., 28IFN-KC).

#### Evaluation of MAO-B inhibitory mechanism

The kinetic analysis of compound **28c** with MAO-B was conducted using a method analogous to that employed in the MAO inhibition assays. Various concentrations of **28c** (0.08 μM, 0.15 μM, and 0.30 μM) and kynuramine (15 μM, 30 μM, 60 μM, and 90 μM) were used. A control experiment was carried out in the absence of **28c**. The data analysis procedure followed previously established protocols.

The reversibility study of **28c** with MAO-B was performed according to the following procedure: MAO-B solution (0.3 ml, 0.06 mg/mL in PBS containing 5% sucrose) and the solution of **28c** (0.3 ml, 4 × IC_50_) were combined in a vial and incubated at 37 °C for 30.0 min. The resulting mixture was transferred into a dialysis bag (Baoke Scientific, MWCO: 10,000; flat width: 24.0 mm; length: 8.0–10.0 cm). The bag was then dialysed against 200.0 ml of PBS for 24.0 h, with the outer PBS buffer being replaced with fresh buffer at 3.0 and 7.0 h. For the non-dialysis group, the mixture was kept at 4.0 °C for 24.0 h without dialysis. After dialysis (or storage for the non-dialysis group), 40.0 μL of the content from the dialysis bag was mixed with 40.0 μL of kynuramine solution (100.0 μM) in a black 96-well plate. The plate was incubated at 37.0 °C for 30 min. The reaction was then quenched by adding 40.0 μL of 2.0 M NaOH aqueous solution and 100.0 μL of water. Fluorescence was measured using a multifunctional microplate reader (Thermo Scientific) at excitation and emission wavelengths of 310 nm and 400 nm, respectively. Safinamide and rasagiline were used as positive and negative controls, respectively. In the blank group, the test compound solution was replaced with the same volume of PBS buffer.

The molecular docking study was performed using the AutoDock 4.2 software package. The crystal structures of MAO-A (PDB ID: *2z5x*) and MAO-B (PDB ID: *2v5z*) were first obtained from the RCSB Protein Data Bank (https://www.rcsb.org/). Subsequently, all original ligands and water molecules were removed from the protein structures, and polar hydrogen atoms were added. For ligand preparation, the chemical structures of the test compounds were drawn using ChemDraw, and their energy-minimized conformations were generated with Chem3D prior to docking. Following the preparation of both macromolecular and ligand files, the torsion trees of the ligands were defined within AutoDock 4.2. The ligands were then positioned into the respective binding sites of the macromolecular receptors using AutoGrid. Upon completion of these steps, the AutoDock program was executed to perform molecular docking, with each ligand typically subjected to 100 independent docking runs. The resulting conformation belonging to the most populated cluster and exhibiting the lowest binding energy was selected for further analysis and discussion.

Molecular dynamics simulations were performed using the Gromacs2022 software package. The GAFF force field was applied for small molecules, while the AMBER14SB force field and TIP3P water model were used for proteins. The simulation system for the complex was constructed by merging the protein and small molecule ligand files. Simulations were conducted under constant temperature and pressure with periodic boundary conditions. During MD simulations, all hydrogen bonds were constrained using the LINCS algorithm with an integration time step of 2 fs. Electrostatic interactions were calculated using the Particle-mesh Ewald (PME) method with a cut-off of 1.2 nm. The non-bonded interaction cut-off was set to 10 Å and updated every 10 steps. The V-rescale temperature coupling method maintained the simulation temperature at 298 K, while the Berendsen method controlled the pressure at 1 bar. After 100 ps of NVT and NPT equilibrium simulations at 298 K, a 100 ns MD simulation was performed for the complex system, with conformations saved every 10 ps. Following the simulation, trajectories were analysed using VMD and PyMOL, and the binding free energy between the protein and small molecule ligand was calculated using the g_mmpbsa program based on the MMPBSA method.

#### Evaluation of AChE inhibitory mechanism

The kinetic properties of compound **28c** against AChE were assessed according to an established procedure. To each well of a 96-well plate, the following reagents were sequentially added: 20.0 μL of the test compound at four graded concentrations, 20.0 μL of phosphate-buffered saline (PBS, pH 8.0), 10.0 μL of AChE solution (0.05 U/mL in PBS), and 30.0 μL of DTNB (0.2% in PBS). The plate was incubated at 37 °C for 15.0 min. Thereafter, 20.0 μL of thioacetylcholine iodide substrate was introduced to achieve final concentrations ranging from 0.1 to 0.4 mM. The change in absorbance at 412 nm was continuously recorded for 15.0 min using a multifunctional microplate reader (Thermo Scientific). Control assays were conducted in the absence of **28c**. Data processing was carried out as described in previous reports.

The reversibility of AChE inhibition by compound **28c** was evaluated using a dialysis method at a concentration equivalent to two times its IC_50_, with donepezil serving as the positive control. Following a 15-min preincubation of **28c** or donepezil with AChE, the remaining enzymatic activities in both dialysed and non-dialysed samples were measured and normalised to the activity of inhibitor-free control groups. The degree of reversibility was determined by comparing the activities before and after dialysis and was further contrasted with results from reference inhibitors.

The molecular docking and molecular dynamics simulation procedures used to study the interaction between **28c** and AChE were similar to those applied for **28c** with MAO-B, with the only difference being that the macromolecule targeted in this case was AChE (PDB ID: *4ey7*).

#### Blood-brain barrier permeation study

The drug-like properties and BBB permeation ability of **28c** was firstly predicted by using two different platform (ADMETlab and SwissADME) at http://admet.scbdd.com/ and http://www.swissadme.ch. Parallel artificial membrane permeability assay (PAMPA) protocol was further carried out to predict the BBB permeation ability of **28c**. Briefly, an artificial lipid membrane was formed by applying porcine brain lipid solution (4 μL, 20 mg/mL in n-dodecane) onto a filter membrane, followed by incubation at r.t. for 5 min. The receptor well was filled with 200 μL of PBS/ethanol (70:30, v/v) mixture. Subsequently, 350 μL **28c** (100 μg/mL) was added to the donor well, which was then carefully assembled over the receptor well to form a “sandwich” configuration. The assembly was incubated at 25 °C for 18 h. After incubation, 150 μL aliquots were collected from both the donor and receptor wells and transferred to a 96-well quartz plate. Absorbance was measured across 200–800 nm, and the permeability coefficient (*Pe*) was calculated according to the established formula.

Adult male C57BL/6J mouse was administered compound **28c** (50 mg/kg) by intragastric gavage. Ten minutes post‑dosing, mouse was anaesthetised *via* intraperitoneal injection of pentobarbital sodium (300 mg/kg; Beijing Dingguo Changsheng Biotechnology Co., Ltd.). Blood was collected from the orbital sinus, after which the mouse was decapitated and the brain was removed immediately. The collected blood was transferred into heparin (Adamas life, 013958170) containing tubes, mixed gently, and centrifuged at 3500 rpm for 10 min to obtain plasma. The whole brain was carefully freed from surrounding tissues, placed in a tissue grinder with 1.0 mL of deionised water, and homogenised to prepare brain tissue homogenate. For plasma sample preparation, 100 μL of plasma was combined with 900 μL of acetonitrile in a microcentrifuge tube, vortexed for 3 min, and centrifuged at 13,000 rpm for 15 min at 4 °C. Subsequently, 1 μL of the supernatant was collected for LC‑MS (Agilent-6460) analysis. For brain homogenate sample preparation, an aliquot of 100 μL was accurately measured, mixed with 900 μL of acetonitrile, vortexed for 3 min, and centrifuged under the same conditions (13,000 rpm, 15 min, 4 °C). Then, 1 μL of the resulting supernatant was subjected to LC‑MS (Agilent-6460) analysis.

#### Stability study

The mice plasma and brain tissue homogenate were obtained according to the similar procedures described above. **28c** was dissolved in DMSO to afford 2.5 mM solution, and then this solution (50.0 μL) was mixed with mice plasma (950.0 μL) or brain tissue homogenate (950.0 μL). The obtained mixtures were incubated at 37.0 °C, and 100.0 μL mixtures were taken out at each time points and mixed with combined with 900 μL of acetonitrile in a microcentrifuge tube, vortexed for 3 min, and centrifuged at 13,000 rpm for 15 min at 4 °C. Subsequently, 10 μL of the supernatant was collected for HPLC (Wayeal LC-3200) analysis.

#### Safety study

The cytotoxicity of compound **28c** against PC-12 cells was evaluated. Briefly, PC-12 cells (Shanghai QuiCell Biotechnology Co., Ltd. &Technology Co.,Ltd) were seeded into a 96-well plate at a density of 1 × 10^5^ cells per mL in DMEM (100 μL per well) and incubated at 37 °C for 24 h. After incubation, 10 μL of test compound solutions at various concentrations were added to the corresponding wells, followed by another 2 h of incubation at 37 °C. Subsequently, the cell viability was assessed using a Cell Counting Kit-8 (Adamas life, 04483227). A Varioskan Flash Multimode Reader (Thermo Scientific) was utilised to measure the absorbance at 450 nm. The cell survival rate was subsequently calculated relative to the control group based on the absorbance values.

As for the acute toxic test, compound **28c** (1.0 g) was weighed and dissolved in edible oil to a final volume of 20 ml, followed by thorough mixing. After a 6-h fasting period (with water withheld), six C57BL/6J mice were orally administered the suspension at a dose of 20.0 ml/kg (equivalent to 1 g/kg of **28c**). Toxic symptoms and mortality were monitored and recorded over the following two weeks. The vital organs were removed and then assessed for pathological damage by HE staining on day 14.

#### Pharmacodynamics study in vivo

A total of forty male C57BL/6J mice (20 ± 5 g) were randomly assigned to four experimental groups (*n* = 10 per group): control, model, positive control, and treatment. Following a two-day acclimation period, all animals except those in the control group received an intrahippocampal injection of A*β*_1-42_ (GL Biochem Ltd., 52483, 2.0 μg per mouse) using a stereotaxic instrument; control mice were administered an equivalent volume of saline (Adamas life, C8395-500). To minimise postoperative infection, all mice received intramuscular penicillin (Adamas life, 15306 A). Starting from the day of surgery, the control and model groups were given saline by intragastric administration, the positive control group received donepezil hydrochloride (Adamas life, 103806B, 5.5 mg/kg), and the treatment group was administered compound **28c** (5.0 mg/kg). All treatments were delivered once daily for seven consecutive days. On day 6, a training session was conducted wherein mice were placed on platforms above an electrified grid. The initial escape latency (time until first jump) was recorded. On day 7, one hour after the final treatment, a formal behavioural test was performed. Both latency and number of jumps (errors) within a 5-min period were measured. Upon completion of behavioural testing, the mice were anaesthetised by intraperitoneal injection of pentobarbital sodium (Beijing Dingguo Changsheng Biotechnology Co. LTD, 300 mg/kg). Four mice from each group underwent transcardial perfusion, while the remaining animals were euthanized via cervical dislocation. Mouse brain sections were obtained following perfusion, blocked with BSA (Beijing Solarbio Science & Technology Co., Ltd., SW3015), and sequentially incubated with the primary antibody NeuN Mouse mAb (Millipore, MAB377, diluted 1:500 in PBS) followed by the corresponding secondary antibody Dylight 594-conjugated Goat Anti-Mouse IgG (Abbkine, A23410, diluted 1:500 in PBS). Nuclei were counterstained with DAPI (Southern Biotech, 010020). Fluorescence images were acquired using a Nikon microscope and processed with NIS Elements software.

## Supplementary Material

Supplementary Material anonymous.doc

## Data Availability

This work was supported by Sichuan Science and Technology Program (2026NSFSC1625) and Doctoral Startup Fund of North Sichuan Medical College (CBY25-QDA26).

## References

[1] Mathys H, Peng Z, Boix CA, Victor MB, Leary N, Babu S, Abdelhady G, Jiang X, Ng AP, Ghafari K, et al. Single-cell atlas reveals correlates of high cognitive function, dementia, and resilience to Alzheimer’s disease pathology. Cell. 2023;186(20):4365–4385. e27. 37774677 10.1016/j.cell.2023.08.039 PMC10601493

[2] Princen K, Van Dooren T, van Gorsel M, Louros N, Yang X, Dumbacher M, Bastiaens I, Coupet K, Dupont S, Cuveliers E, et al. Pharmacological modulation of septins restores calcium homeostasis and is neuroprotective in models of Alzheimer’s disease. Science. 2024;384(6699):eadd6260. 38815015 10.1126/science.add6260 PMC11827694

[3] Koch G, Altomare D, Benussi A, Bréchet L, Casula EP, Dodich A, Pievani M, Santarnecchi E, Frisoni GB. The emerging field of non-invasive brain stimulation in Alzheimer’s disease. Brain. 2024;147(12):4003–4016. 39562009 10.1093/brain/awae292 PMC11734340

[4] Tenchov R, Sasso JM, Zhou QA. Alzheimer’s disease: exploring the landscape of cognitive decline. ACS Chem Neurosci. 2024;15(21):3800–3827. 39392435 10.1021/acschemneuro.4c00339 PMC11587518

[5] Zhang J, Zhang Y, Wang J, Xia Y, Zhang J, Chen L. Recent advances in Alzheimer’s disease: mechanisms, clinical trials and new drug development strategies. Signal Transduct Target Ther. 2024;9(1):211. 39174535 10.1038/s41392-024-01911-3 PMC11344989

[6] Zhao X, Hu Q, Wang X, Li C, Chen X, Zhao D, Qiu Y, Xu H, Wang J, Ren L, et al. Dual-target inhibitors based on acetylcholinesterase: novel agents for Alzheimer’s disease. Eur J Med Chem. 2024;279:116810. 39243456 10.1016/j.ejmech.2024.116810

[7] Liu S, Zheng C, Nechanitzky R, Luo P, Ramachandran P, Nguyen D, Elia AJ, Moghadas Jafari S, Law R, Snow BE, et al. Cholinergic regulation of thymocyte negative selection. Nat Immunol. 2025;26(6):881–893. 40399609 10.1038/s41590-025-02152-4

[8] Kelly M, Garner M, Cooper EM, Orsini CA. Cholinergic regulation of decision making under risk of punishment. Neurobiol Learn Mem. 2025;217:108018. 39710058 10.1016/j.nlm.2024.108018 PMC12670459

[9] Kuzu B, Alagoz MA, Demir Y, Gulcin I, Burmaoglu S, Algul O. Structure-based inhibition of acetylcholinesterase and butyrylcholinesterase with 2-Aryl-6-carboxamide benzoxazole derivatives: synthesis, enzymatic assay, and in silico studies. Mol Divers. 2025;29(1):671–693. 38554169 10.1007/s11030-024-10828-6 PMC11785640

[10] Reddi Sree R, Kalyan M, Anand N, Mani S, Gorantla VR, Sakharkar MK, Song B-J, Chidambaram SB. Newer therapeutic approaches in treating Alzheimer’s disease: a comprehensive review. ACS Omega. 2025;10(6):5148–5171. 39989768 10.1021/acsomega.4c05527 PMC11840625

[11] Tang X, Zhang Y, Wang Q, Li Z, Zhang C. Detection of acetylcholinesterase and butyrylcholinesterase in vitro and in vivo using a new fluorescent probe. Chem Commun (Camb). 2024;60(15):2082–2085. 38293842 10.1039/d3cc06055a

[12] Dawood DH, Anwar MM. Recent advances in the therapeutic insights of thiazole scaffolds as acetylcholinesterase inhibitors. Eur J Med Chem. 2025;287:117331. 39938408 10.1016/j.ejmech.2025.117331

[13] Jaisa-Aad M, Muñoz-Castro C, Healey MA, Hyman BT, Serrano-Pozo A. Characterization of monoamine oxidase-B (MAO-B) as a biomarker of reactive astrogliosis in Alzheimer’s disease and related dementias. Acta Neuropathol. 2024;147(1):66. 38568475 10.1007/s00401-024-02712-2 PMC10991006

[14] Liu P-P, Xie Y, Meng X-Y, Kang J-S. History and progress of hypotheses and clinical trials for Alzheimer’s disease. Signal Transduct Target Ther. 2019;4(1):29. 31637009 10.1038/s41392-019-0063-8 PMC6799833

[15] Sánchez-Rodríguez R, Munari F, Angioni R, Venegas F, Agnellini A, Castro-Gil MP, Castegna A, Luisetto R, Viola A, Canton M. Targeting monoamine oxidase to dampen NLRP3 inflammasome activation in inflammation. Cell Mol Immunol. 2021;18(5):1311–1313. 32346102 10.1038/s41423-020-0441-8 PMC8093264

[16] Jones DN, Raghanti MA. The role of monoamine oxidase enzymes in the pathophysiology of neurological disorders. J Chem Neuroanat. 2021;114:101957. 33836221 10.1016/j.jchemneu.2021.101957

[17] Li SL, Ni NJ, Wu XM, Lan T, Yu YF. Protective effect of fangchinoline against glaucoma and neuroinflammation in unilateral ocular hypertension in mice. International J of Pharmacology. 2023;19(1):131–138.

[18] Yi X, Xu C, Huang P, Zhang L, Qing T, Li J, Wang C, Zeng T, Lu J, Han Z. 1-Trifluoromethoxyphenyl-3-(1-Propionylpiperidin-4-yl) Urea Protects the Blood-Brain Barrier Against Ischemic Injury by Upregulating Tight Junction Protein Expression, Mitigating Apoptosis and Inflammation InVivo and In Vitro Model. Front Pharmacol. 2020;11:1197. 32848796 10.3389/fphar.2020.01197 PMC7427473

[19] Qin D, Yue R, Deng P, Wang X, Zheng Z, Lv M, Zhang Y, Pu J, Xu J, Liang Y, et al. 8-Formylophiopogonanone B antagonizes doxorubicin-induced cardiotoxicity by suppressing heme oxygenase-1-dependent myocardial inflammation and fibrosis. Biomed Pharmacother. 2021;140:111779. 34062415 10.1016/j.biopha.2021.111779

[20] Yang Y, Yao Z, Wang H, Jia S, Wang M, Wang S, Yun D. Severe inflammation in C57/BL6 mice leads to prolonged cognitive impairment by initiating the IL-1β/TRPM2 pathway. Int Immunopharmacol. 2024;128:111380. 38176340 10.1016/j.intimp.2023.111380

[21] Chatzipieris FP, Kokkalis A, Georgiou N, Petsas E, Apostolou EV, Vougioukalakis GC, Mavromoustakos T. New prospects in the inhibition of monoamine oxidase-B (MAO-B) utilizing propargylamine derivatives for the treatment of Alzheimer’s disease: a review. ACS Omega. 2025;10(25):26208–26232. 40620970 10.1021/acsomega.5c00134 PMC12223828

[22] Zeynep z, Mehmet Abdullah A, Mer Faruk B, Ecio l, Selim G. Monoamine oxidase-B (MAO-B) inhibitors in the treatment of Alzheimer’s and Parkinson’s disease. Curr. Med. Chem. 2021;28(29):6045–6065. 33538661 10.2174/0929867328666210203204710

[23] Wu C-K, Fuh J-L. A 2025 update on treatment strategies for the Alzheimer’s disease spectrum. J Chin Med Assoc. 2025;88(7):495–502. 40442885 10.1097/JCMA.0000000000001252 PMC12637128

[24] Azam U, Naseer MM, Rochais C. Analysis of skeletal diversity of multi-target directed ligands (MTDLs) targeting Alzheimer’s disease. Eur J Med Chem. 2025;286:117277. 39848035 10.1016/j.ejmech.2025.117277

[25] Zou D, Liu R, Lv Y, Guo J, Zhang C, Xie Y. Latest advances in dual inhibitors of acetylcholinesterase and monoamine oxidase B against Alzheimer’s disease. J Enzyme Inhib Med Chem. 2023;38(1):2270781. 37955252 10.1080/14756366.2023.2270781 PMC10653629

[26] Jon J, Jeong J, Jung J, Cho H, Song K, Kim E-S, Lee SH, Han E, Chung W-H, Moon A, et al. Recent advances in donepezil delivery systems via the nose-to-brain pathway. Pharmaceutics. 2025;17(8):958. 40870981 10.3390/pharmaceutics17080958 PMC12389716

[27] Cheung J, Rudolph MJ, Burshteyn F, Cassidy MS, Gary EN, Love J, Franklin MC, Height JJ. Structures of human acetylcholinesterase in complex with pharmacologically important ligands. J Med Chem. 2012;55(22):10282–10286. 23035744 10.1021/jm300871x

[28] Khalid MB, Shahzad F, Siddiqui MR, Abedin MZU, Hulou S, Shami B, Ahmed SI. Comparative efficacy and safety of irreversible (rasagiline) and reversible (safinamide) monoamine oxidase inhibitors as add-on therapy for Parkinson’s disease. J Neurol. 2025;272(7):486. 40590994 10.1007/s00415-025-13230-w

[29] Binda C, Wang J, Pisani L, Caccia C, Carotti A, Salvati P, Edmondson DE, Mattevi A. Structures of human monoamine oxidase B complexes with selective noncovalent inhibitors: safinamide and coumarin analogs. J Med Chem. 2007;50(23):5848–5852. 17915852 10.1021/jm070677y

[30] Li Q, He S, Chen Y, Feng F, Qu W, Sun H. Donepezil-based multi-functional cholinesterase inhibitors for treatment of Alzheimer’s disease. Eur J Med Chem. 2018;158:463–477. 30243151 10.1016/j.ejmech.2018.09.031

[31] Meena P, Nemaysh V, Khatri M, Manral A, Luthra PM, Tiwari M. Synthesis, biological evaluation and molecular docking study of novel piperidine and piperazine derivatives as multi-targeted agents to treat Alzheimer’s disease. Bioorg Med Chem. 2015;23(5):1135–1148. 25624107 10.1016/j.bmc.2014.12.057

[32] Dias KST, de Paula CT, dos Santos T, Souza INO, Boni MS, Guimarães MJR, da Silva FMR, Castro NG, Neves GA, Veloso CC, et al. Design, synthesis and evaluation of novel feruloyl-donepezil hybrids as potential multitarget drugs for the treatment of Alzheimer’s disease. Eur J Med Chem. 2017;130:440–457. 28282613 10.1016/j.ejmech.2017.02.043

[33] Huang L, Miao H, Sun Y, Meng F, Li X. Discovery of indanone derivatives as multi-target-directed ligands against Alzheimer’s disease. Eur J Med Chem. 2014;87:429–439. 25282266 10.1016/j.ejmech.2014.09.081

[34] Guzior N, Bajda M, Rakoczy J, Brus B, Gobec S, Malawska B. Isoindoline-1,3-dione derivatives targeting cholinesterases: design, synthesis and biological evaluation of potential anti-Alzheimer’s agents. Bioorg Med Chem. 2015;23(7):1629–1637. 25707322 10.1016/j.bmc.2015.01.045

[35] Sang Z, Wang K, Wang H, Yu L, Wang H, Ma Q, Ye M, Han X, Liu W. Design, synthesis and biological evaluation of phthalimide-alkylamine derivatives as balanced multifunctional cholinesterase and monoamine oxidase-B inhibitors for the treatment of Alzheimer’s disease. Bioorg Med Chem Lett. 2017;27(22):5053–5059. 29033232 10.1016/j.bmcl.2017.09.055

[36] Sudevan ST, Rangarajan TM, Al‐Sehemi AG, Nair AS, Koyiparambath VP, Mathew B. Revealing the role of the benzyloxy pharmacophore in the design of a new class of monoamine oxidase‐B inhibitors. Arch Pharm (Weinheim). 2022;355(8):e2200084. 35567313 10.1002/ardp.202200084

[37] Wang Z, Wu J, Yang X, Cai P, Liu Q, Wang KDG, Kong L, Wang X. Neuroprotective effects of benzyloxy substituted small molecule monoamine oxidase B inhibitors in Parkinson’s disease. Bioorg Med Chem. 2016;24(22):5929–5940. 27692996 10.1016/j.bmc.2016.09.050

[38] Cao Z, Zhang T, Fu X, Wang X, Xia Q, Zhong L, Zhu J. 2‐Hydroxy‐4‐benzyloxylimine resveratrol derivatives as potential multifunctional agents for the treatment of Parkinson’s disease. ChemMedChem. 2023;18(6):e202200629. 36622947 10.1002/cmdc.202200629

[39] Jin C-F, Wang Z-Z, Chen K-Z, Xu T-F, Hao G-F. Computational fragment-based design facilitates discovery of potent and selective monoamine oxidase-B (MAO-B) inhibitor. J Med Chem. 2020;63(23):15021–15036. 33210537 10.1021/acs.jmedchem.0c01663

[40] Cao Z, Song Q, Yu G, Liu Z, Cong S, Tan Z, Deng Y. Novel 3-benzylidene/benzylphthalide Mannich base derivatives as potential multifunctional agents for the treatment of Alzheimer’s disease. Bioorg Med Chem. 2021;35:116074. 33640707 10.1016/j.bmc.2021.116074

[41] Harvey JE, Raw SA, Taylor RJK. The first synthesis of the epoxide-containing macrolactone nucleus of oximidine I. Tetrahedron Lett. 2003;44(38):7209–7212.

[42] Lin JG, Zhang WG, Zhao R, Niu ZY, Bao K, Liu DL, Wang NL, Yao XS. Total synthesis of three new dihydrostilbenes from Bulbophllum odoratissimum. Chin. Chem. Lett. 2006;17(3):307–309.

[43] Li W, Hong L, Li L, Yuan Y, Ding Y, Zhu J, Wang C, Cao Z, Tian X. Design, synthesis and biological evaluation of N-substituted nipecotamide derivatives as multifunctional agents for epilepsy treatment. Eur J Med Chem. 2025;292:117613. 40300460 10.1016/j.ejmech.2025.117613

[44] Liu S, Yin D, Li W, Liu D, Zhou X. Structure-supercooling property relationship of phenylethyl phenylacetate derivatives and analogue. J. Mol. Struct. 2021;1241:130680.

[45] Treitler DS, Marriott AS, Chadwick J, Quirk E. Mutagenic impurities in 1-hydroxybenzotriazole (HOBt). Org Process Res Dev. 2019;23(11):2562–2566.

[46] Legoabe LJ, Petzer A, Petzer JP. Selected C7-substituted chromone derivatives as monoamine oxidase inhibitors. Bioorg Chem. 2012;45:1–11. 23064123 10.1016/j.bioorg.2012.08.003

[47] Lu C, Zhou Q, Yan J, Du Z, Huang L, Li X. A novel series of tacrine–selegiline hybrids with cholinesterase and monoamine oxidase inhibition activities for the treatment of Alzheimer’s disease. Eur J Med Chem. 2013;62:745–753. 23454517 10.1016/j.ejmech.2013.01.039

[48] Larit F, Elokely KM, Chaurasiya ND, Benyahia S, Nael MA, León F, Abu-Darwish MS, Efferth T, Wang Y-H, Belouahem-Abed D, et al. Inhibition of human monoamine oxidase A and B by flavonoids isolated from two Algerian medicinal plants. Phytomedicine. 2018;40:27–36. 29496172 10.1016/j.phymed.2017.12.032 PMC5947877

[49] Chaurasiya ND, Gogineni V, Elokely KM, León F, Núñez MJ, Klein ML, Walker LA, Cutler SJ, Tekwani BL. Isolation of acacetin from Calea urticifolia with inhibitory properties against human monoamine oxidase-A and -B. J Nat Prod. 2016;79(10):2538–2544. 27754693 10.1021/acs.jnatprod.6b00440

[50] Zheng L, Qin X, Wang J, Zhang M, An Q, Xu J, Qu X, Cao X, Niu B. Discovery of MAO-B inhibitor with machine learning, topomer CoMFA, molecular docking and multi-spectroscopy approaches. Biomolecules. 2022;12(10):1470. 36291679 10.3390/biom12101470 PMC9599443

[51] Thai KM, Pham DT, Ngo TM, Nguyen HT, Nguyen PV, Pham TQ, Nguyen DN, Nguyen QT, Le MT. Targeting MAO-B selectivity: computational screening, docking, and molecular dynamics insights. SAR QSAR Environ Res. 2025;36(7):583–619. 40799016 10.1080/1062936X.2025.2537248

[52] Pourabdi L, Khoobi M, Nadri H, Moradi A, Moghadam FH, Emami S, Mojtahedi MM, Haririan I, Forootanfar H, Ameri A, et al. Synthesis and structure-activity relationship study of tacrine-based pyrano[2,3-c]pyrazoles targeting AChE/BuChE and 15-LOX. Eur J Med Chem. 2016;123:298–308. 27484515 10.1016/j.ejmech.2016.07.043

[53] Oh JM, Jang H-J, Kang M-G, Song S, Kim D-Y, Kim JH, Noh J-I, Park JE, Park D, Yee S-T, et al. Acetylcholinesterase and monoamine oxidase-B inhibitory activities by ellagic acid derivatives isolated from Castanopsis cuspidata var. sieboldii. Sci Rep. 2021;11(1):13953. 34230570 10.1038/s41598-021-93458-4 PMC8260592

[54] Sağlık BN, Osmaniye D, Acar Çevik U, Levent S, Kaya Çavuşoğlu B, Atlı Eklioğlu Ö, Özkay Y, Koparal AS, Kaplancıklı ZA. Synthesis, in vitro enzyme activity and molecular docking studies of new benzylamine-sulfonamide derivatives as selective MAO-B inhibitors. J Enzyme Inhib Med Chem. 2020;35(1):1422–1432. 32602377 10.1080/14756366.2020.1784892 PMC7821958

[55] Osmaniye D, Evren AE, Sağlık BN, Levent S, Özkay Y, Kaplancıklı ZA. Design, synthesis, biological activity, molecular docking, and molecular dynamics of novel benzimidazole derivatives as potential AChE/MAO-B dual inhibitors. Arch Pharm (Weinheim). 2022;355(3):e2100450. 34931332 10.1002/ardp.202100450

[56] Dong J, Wang N-N, Yao Z-J, Zhang L, Cheng Y, Ouyang D, Lu A-P, Cao D-S. ADMETlab: a platform for systematic ADMET evaluation based on a comprehensively collected ADMET database. J Cheminform. 2018;10(1):29. 29943074 10.1186/s13321-018-0283-x PMC6020094

[57] Daina A, Michielin O, Zoete V. SwissADME: a free web tool to evaluate pharmacokinetics, drug-likeness and medicinal chemistry friendliness of small molecules. Sci Rep. 2017;7(1):42717. 28256516 10.1038/srep42717 PMC5335600

[58] Di L, Kerns EH, Fan K, McConnell OJ, Carter GT. High throughput artificial membrane permeability assay for blood–brain barrier. Eur J Med Chem. 2003;38(3):223–232. 12667689 10.1016/s0223-5234(03)00012-6

[59] Xiang H, Zhang Q, Han Y, Yang L, Zhang Y, Liu Q, Zhang Z, Zhang L. Novel brain-targeting 3-n-butylphthalide prodrugs for ischemic stroke treatment. J Control Release. 2021;335:498–514. 34087248 10.1016/j.jconrel.2021.05.045

[60] Zeng Y, Guo R, Chen S, Lin Y, Cao S, Wang X, Zhang S, Xu H, Qing W, Yang H, et al. Inhibition of diacylglycerol O-acyltransferase 1 provides neuroprotection by inhibiting ferroptosis in ischemic stroke. Mol Med. 2025;31(1):191. 40375180 10.1186/s10020-025-01255-w PMC12082899

[61] Zhang J, Liu B, Ren R, Song S, Bao X, Huan X, Li H, Xu J, Yu T, Wang R, et al. Discovery and optimization of a series of novel morpholine-containing USP1 inhibitors. J Med Chem. 2025;68(3):3673–3699. 10.1021/acs.jmedchem.4c02792. 39902599

[62] Li W, Huang J, Chen Z, Zhang D, He L, Guo Y, Zhong L, Yang C, Yang C, Zeng M, et al. Design, synthesis, biological evaluation and docking studies of 2-hydroxy-4-benzyloxy chalcone derivatives as multifunctional agents for the treatment of Alzheimer’s disease. Curr Med Chem. 2025;33:32. 10.2174/0109298673328877241113091539 39806952

[63] Li Q, Zhao Y, Guo H, Li Q, Yan C, Li Y, He S, Wang N, Wang Q. Impaired lipophagy induced-microglial lipid droplets accumulation contributes to the buildup of TREM1 in diabetes-associated cognitive impairment. Autophagy. 2023;19(10):2639–2656. 37204119 10.1080/15548627.2023.2213984 PMC10472854

