# Supplementary material for: Design, synthesis and biological evaluation of donepezil-safinamide hybrids as dual AChE and MAO-B inhibitor for Alzheimer’s disease treatment
Source: J Enzyme Inhib Med Chem. 2026 Feb 6;41(1):2622769. doi: 10.1080/14756366.2026.2622769 (PMC12885038; doi:10.1080/14756366.2026.2622769)
Supplement: Supplementary Material anonymous.doc [file IENZ_A_2622769_SM1889.doc]

**Design, synthesis and biological evaluation of** **donepezil-safinamide hybrids as dual AChE and MAO-B inhibitor for Alzheimer's disease treatment**

**Table of Contents**

**1.** **Figure S1**. The B-factor analysis and surface electrostatic potential analysis

**2**. **Table S1**. Permeability (*P*_e_ × 10^-6^ cm/s) in the PAMPA-BBB assay for 11 commercial drugs used in the experiment validation

**3.** **Figure S2.** Linear correlation between experimental and reported permeability values of commercial drugs, determined using the PAMPA-BBB assay

**4.** **Table S2**. Ranges of permeability of PAMPA-BBB assays (*P*_e_ × 10^-6^ cm/s)

**5.** The ^1^H NMR, ^13^C NMR, ESI-MS spectra of intermediates target compounds


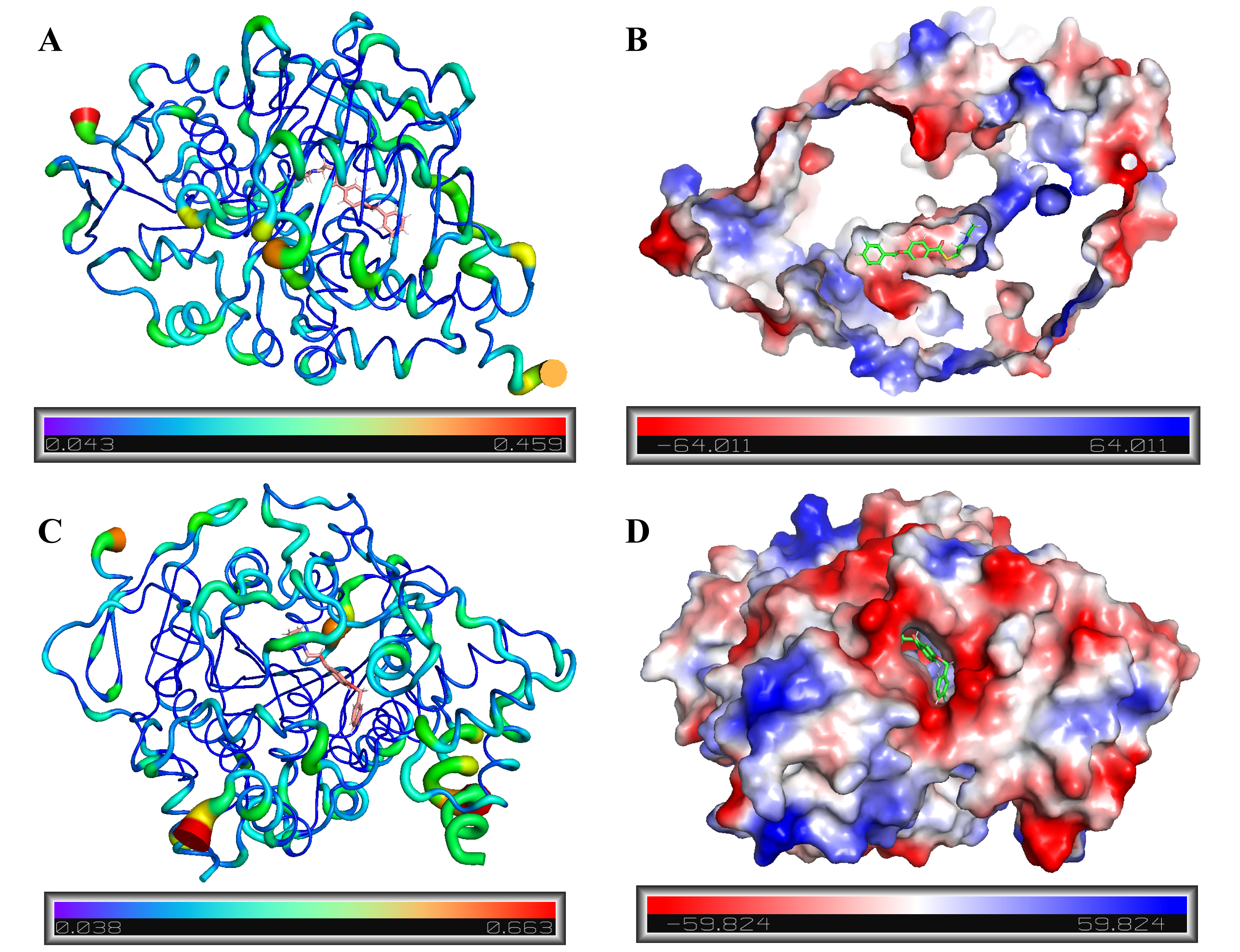


**Figure S1**. (A) B-factor analysis of **28c** with MAO-B; (B) Surface electrostatic potential analysis of **28c** with MAO-B; (C) B-factor analysis of **28c** with AChE; (D) Surface electrostatic potential analysis of **28c** with AChE.

**Table S1**. Permeability *P*_e_ (× 10^-6^ cm/s) in the PAMPA-BBB assay for 11 commercial drugs used in the experiment validation.

| Commercial drugs | Bibl^a^ | PBS/EtOH (70:30)^b^ |
| --- | --- | --- |
| Verapamil | 16 | 16.90 ± 0.36 |
| Oxazepam | 10 | 9.60 ± 0.21 |
| Diazepam | 16 | 11.86 ± 0.23 |
| Clonidine | 5.3 | 5.10 ± 0.16 |
| Imipramine | 13 | 10.10 ± 0.22 |
| Testosterone | 17 | 16.30 ± 0.25 |
| Caffeine | 1.3 | 1.28 ± 0.05 |
| Enoxacine | 0.9 | 0.47 ± 0.01 |
| Piroxicam | 2.5 | 0.72 ± 0.02 |
| Norfloxacin | 0.1 | 0.42 ± 0.01 |
| Theophylline | 0.12 | 0.10 ± 0.003 |

^a^ Taken from Ref.^1^

^b^ Data are the mean ± SD of three independent experiments.


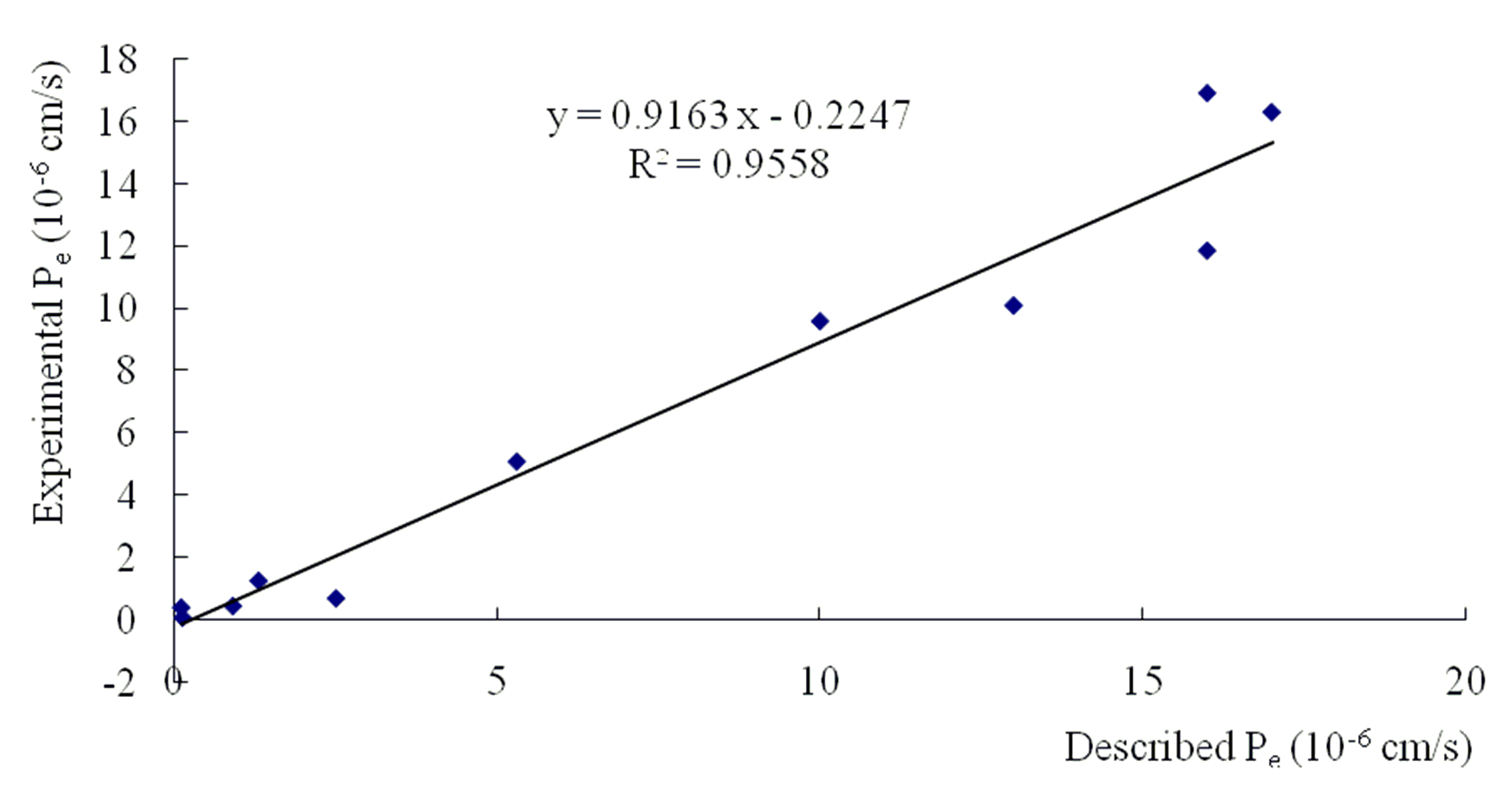


**Figure S2**. Lineal correlation between experimental and reported permeability of commercial drugs using the PAMPA-BBB assay. *P*_e_ (exp.) = 0.9163 × *P*_e_ (bibl.) − 0.2247 (*R*^2^ = 0.9558).

**Table S2**. Ranges of permeability of PAMPA-BBB assays (*P*_e_ × 10^-6^ cm/s).

| High BBB permeation predicted (CNS +) | *P*_e_ > 3.44 |
| --- | --- |
| Uncertain BBB permeation (CNS +/-) | 3.44 > *P*_e_ > 1.61 |
| Low BBB permeation predicted (CNS -) | *P*_e_ < 1.61 |

**References**

1. Di, L.; Kerns, E. H.; Fan, K.; McConnell, O. J.; Carter, G. T. High throughput artificial membrane permeability assay for blood–brain barrier. *Eur. J. Med. Chem*. **2003**, 38, 223-232.


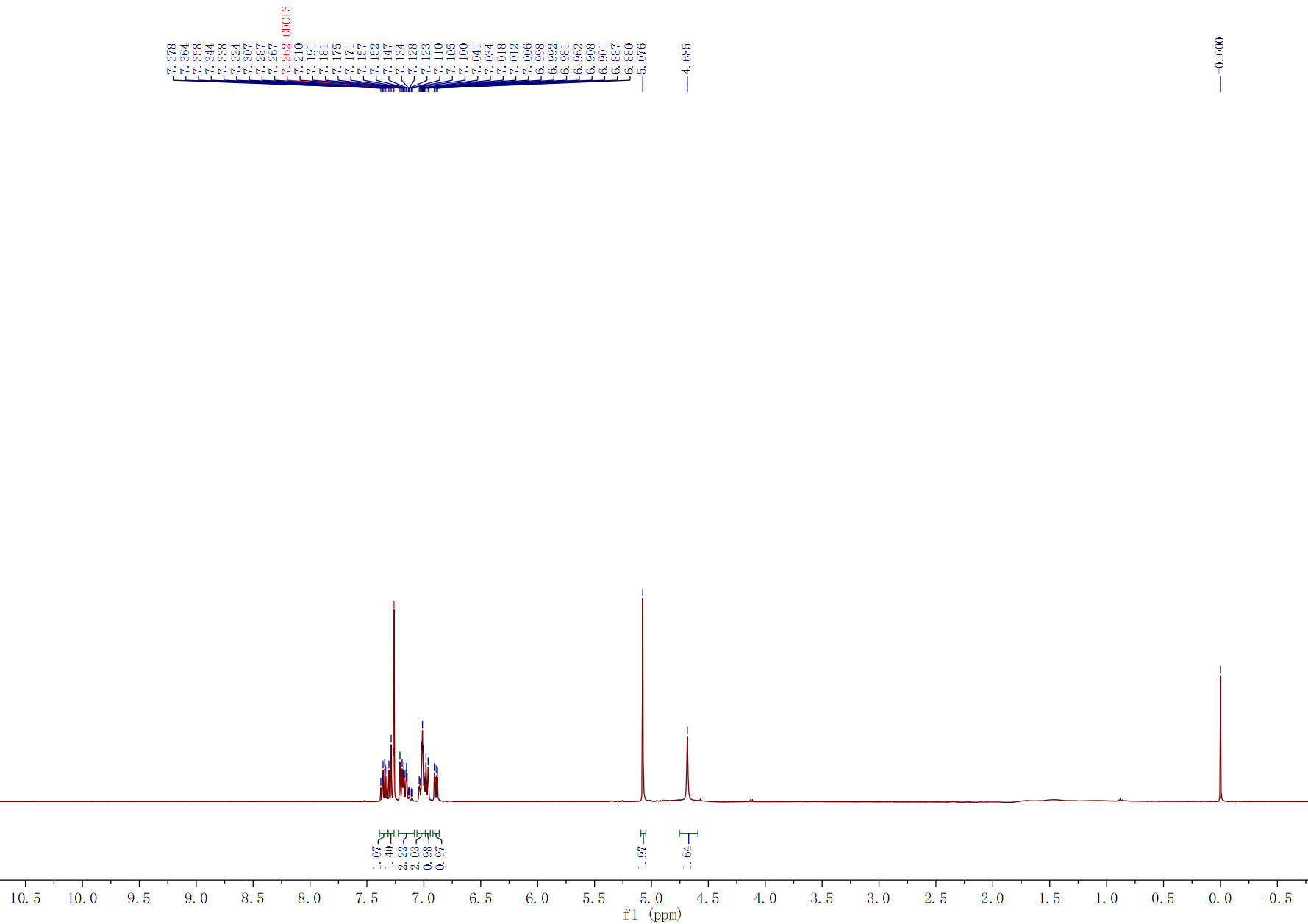


**^1^H NMR spectra of compound 3 (400 MHz, CDCl_3_)**


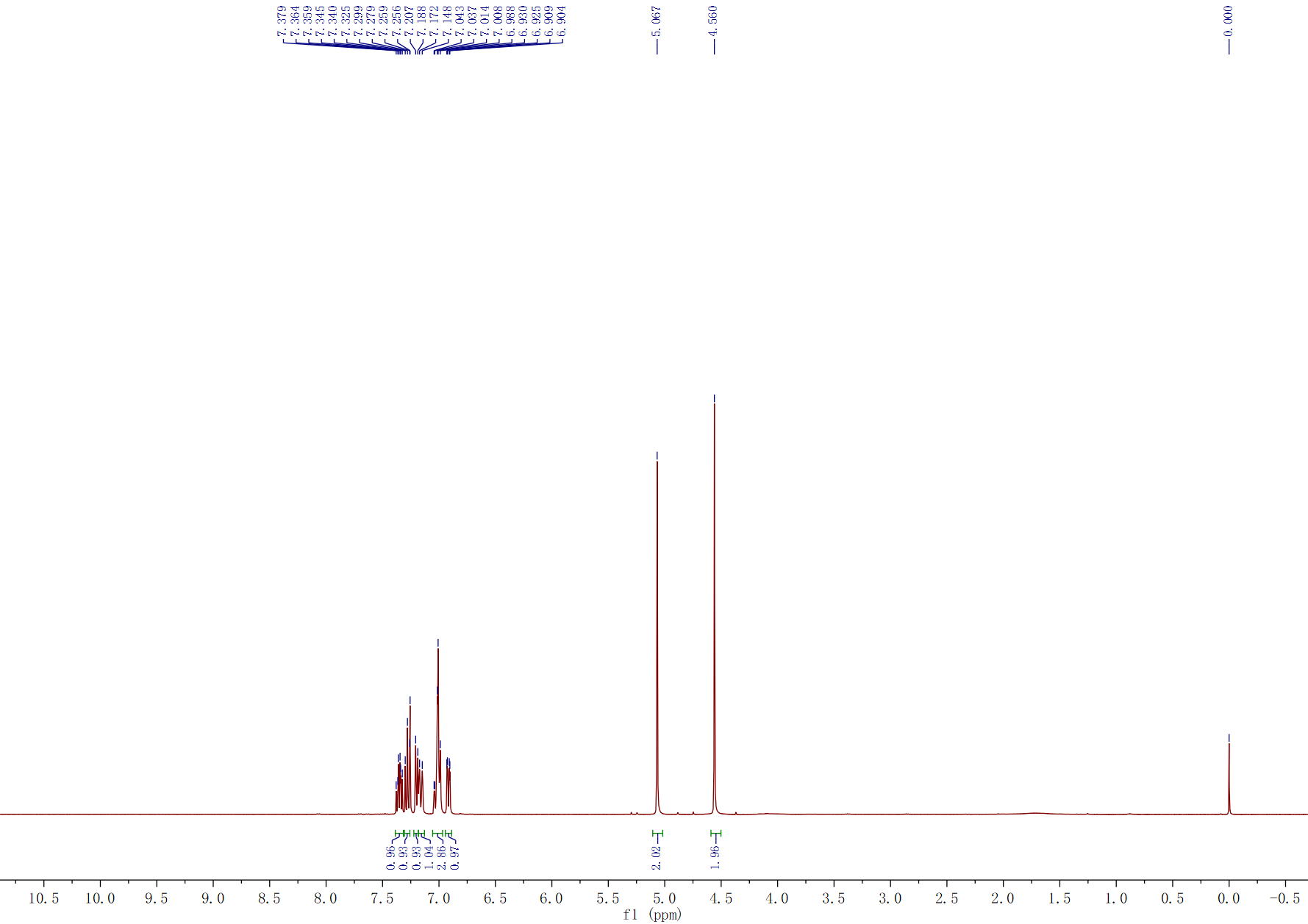


**^1^H NMR spectra of compound 4 (400 MHz, CDCl_3_)**


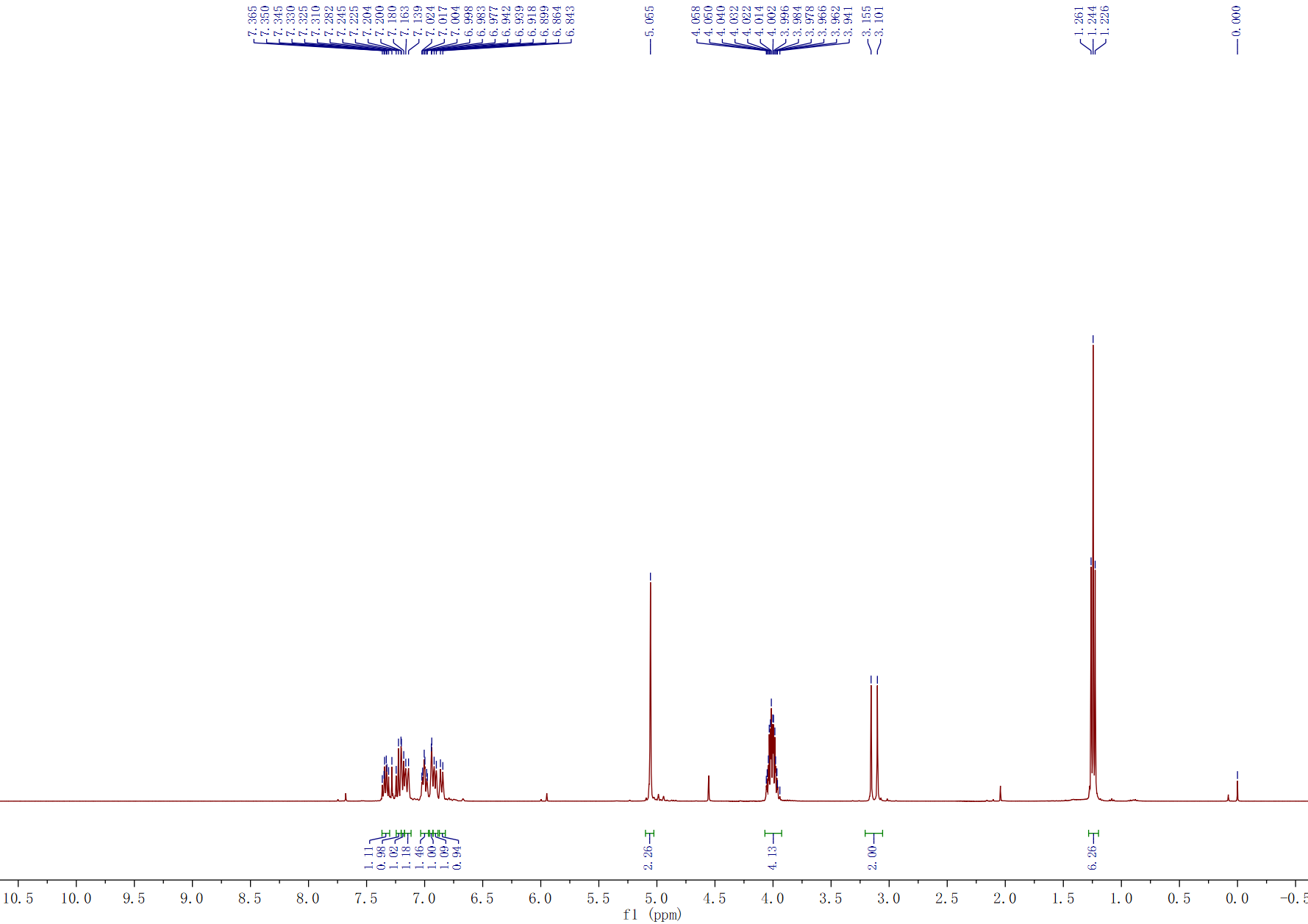


**^1^H NMR spectra of compound 5 (400 MHz, CDCl_3_)**


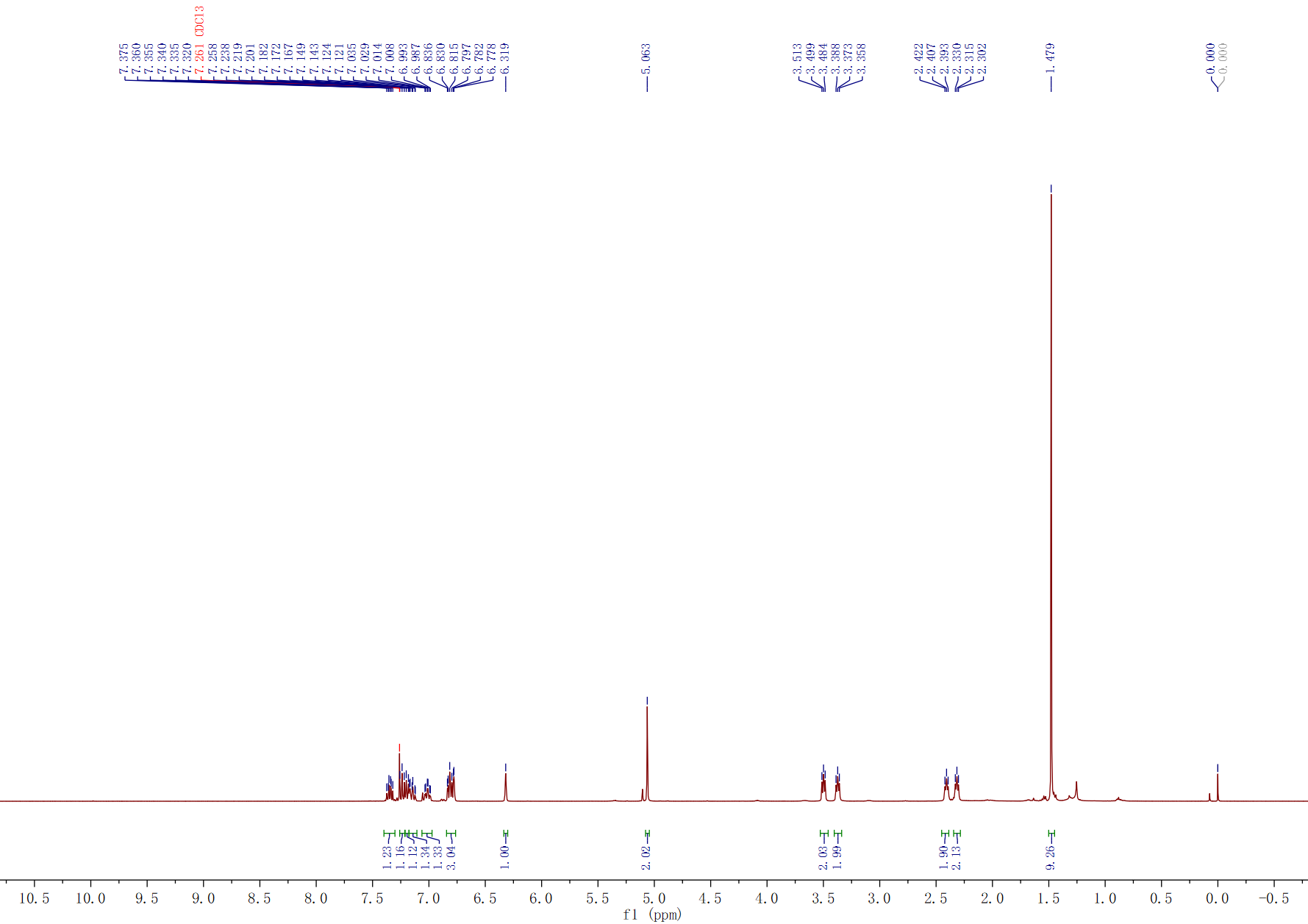


**^1^H NMR spectra of compound 6 (400 MHz, CDCl_3_)**


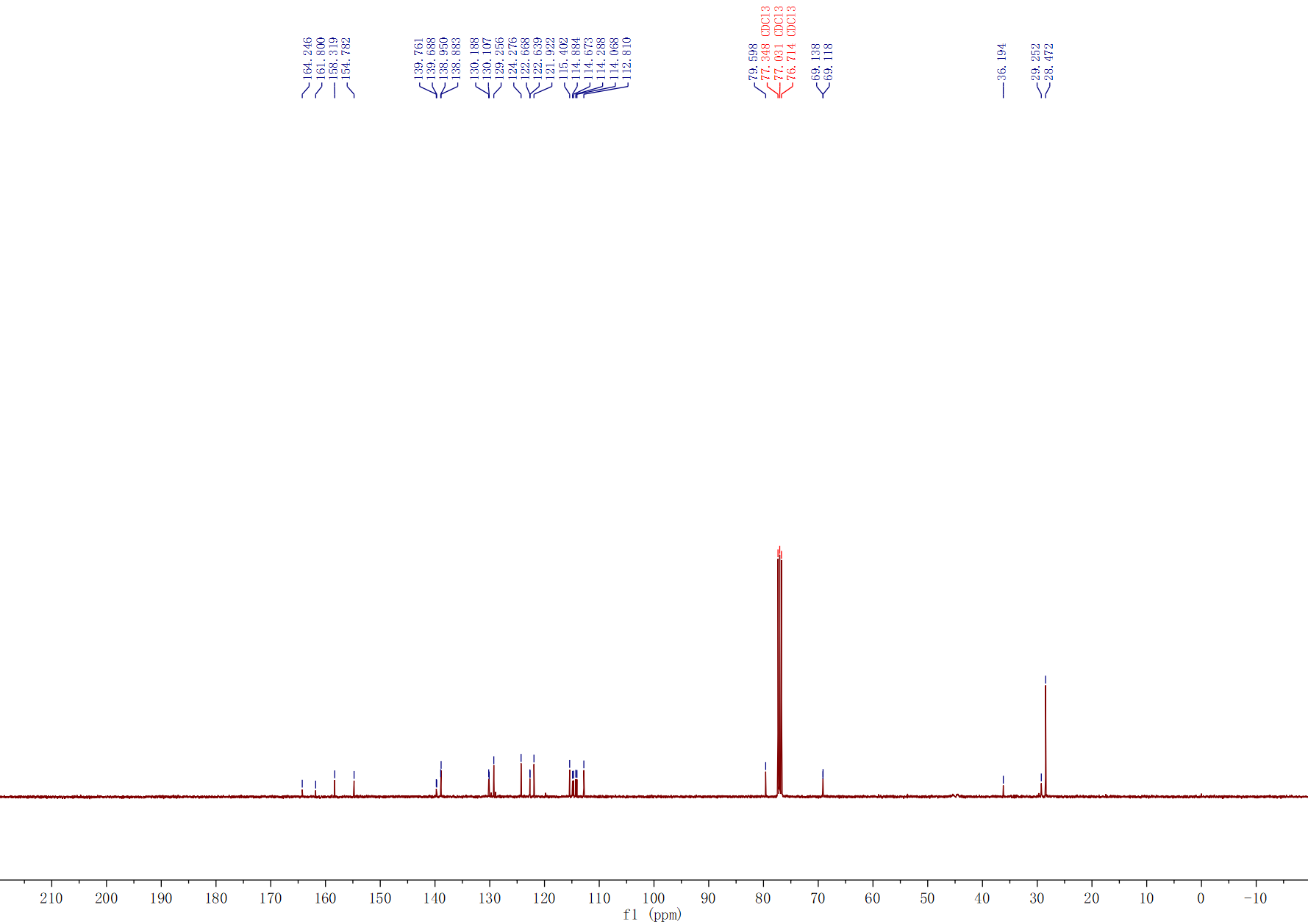


**^13^C NMR of compound 6 (100 MHz, CDCl_3_)**


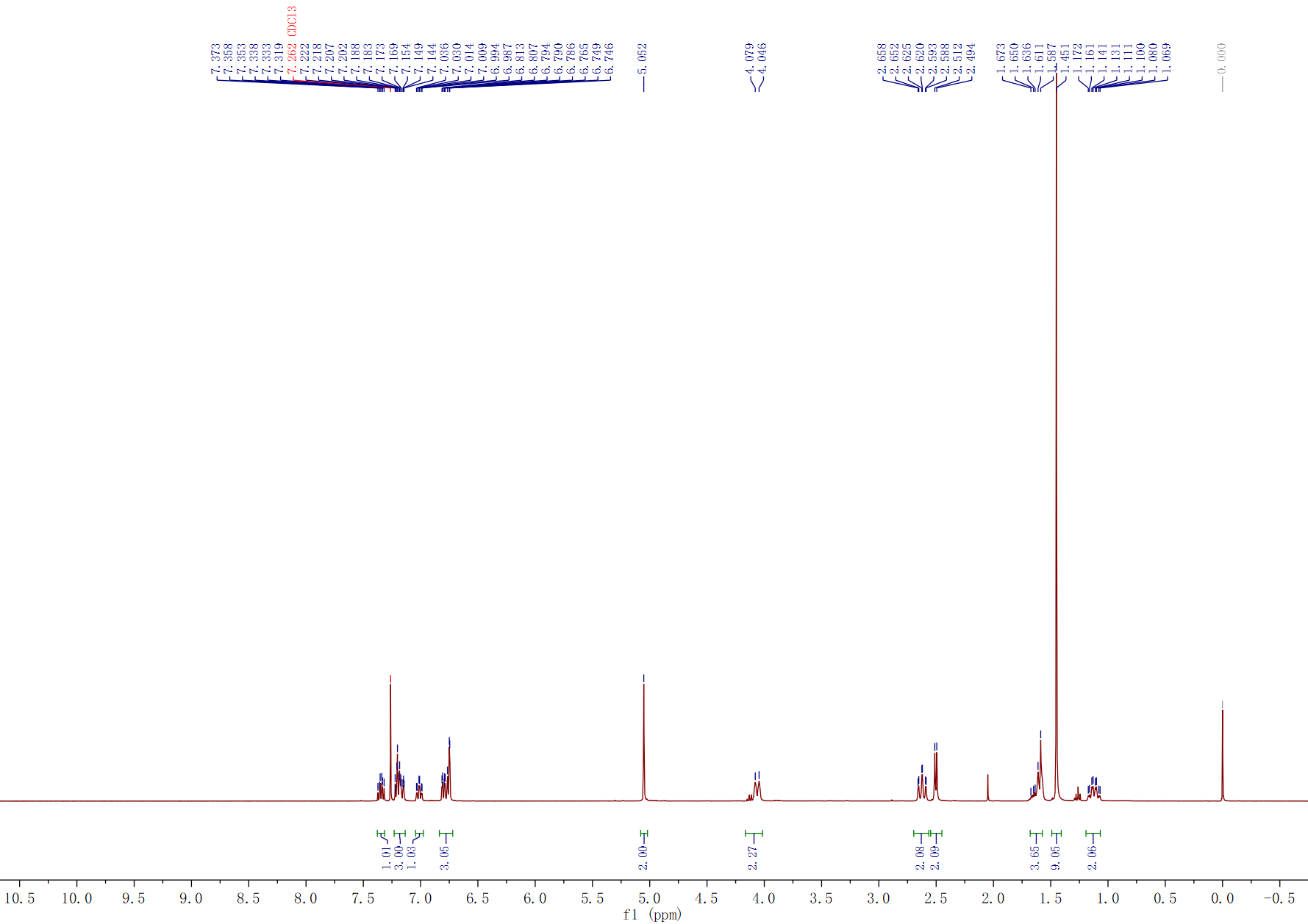


**^1^H NMR spectra of compound 7 (400 MHz, CDCl_3_)**


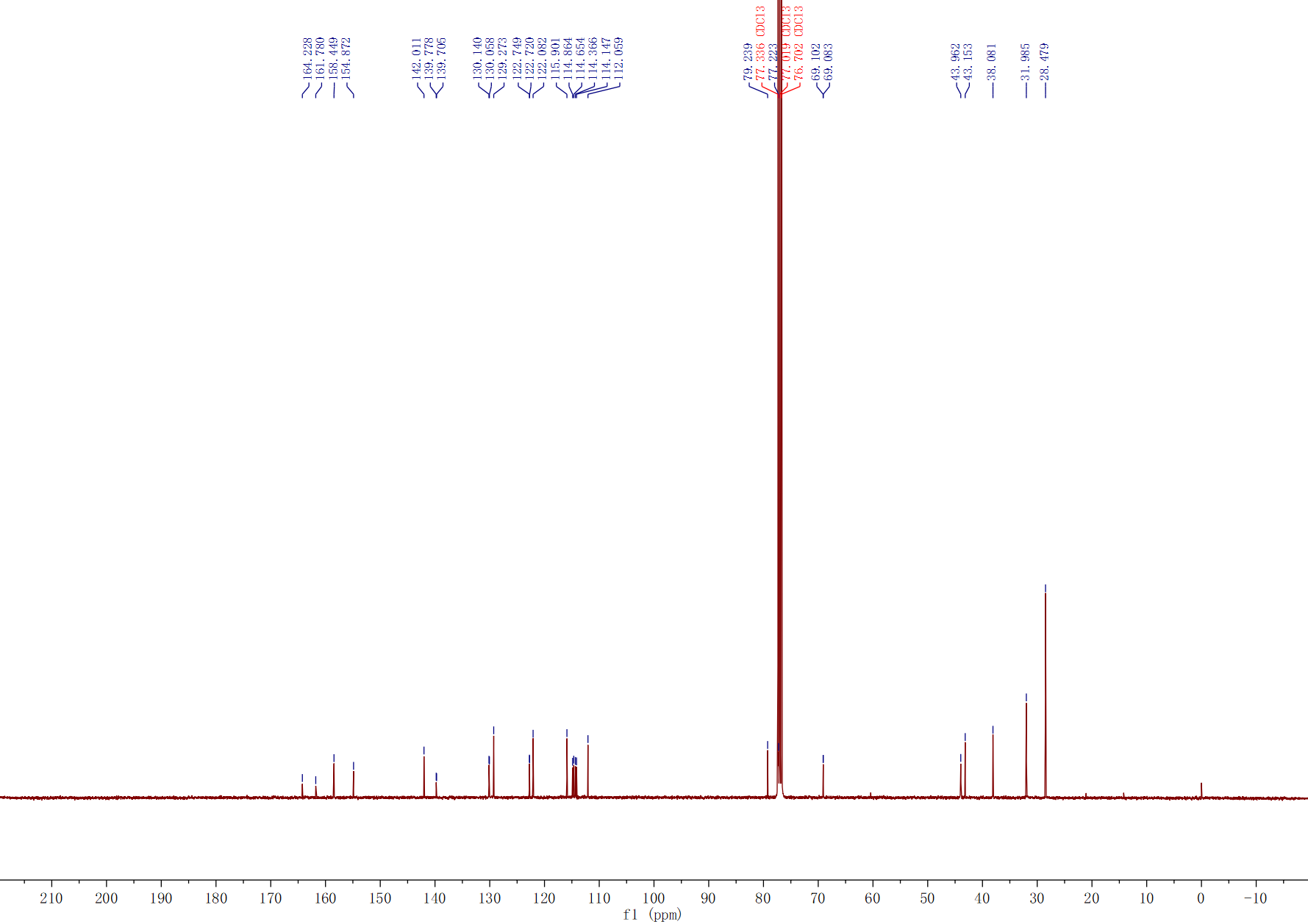


**^13^C NMR of compound 7 (100 MHz, CDCl_3_)**


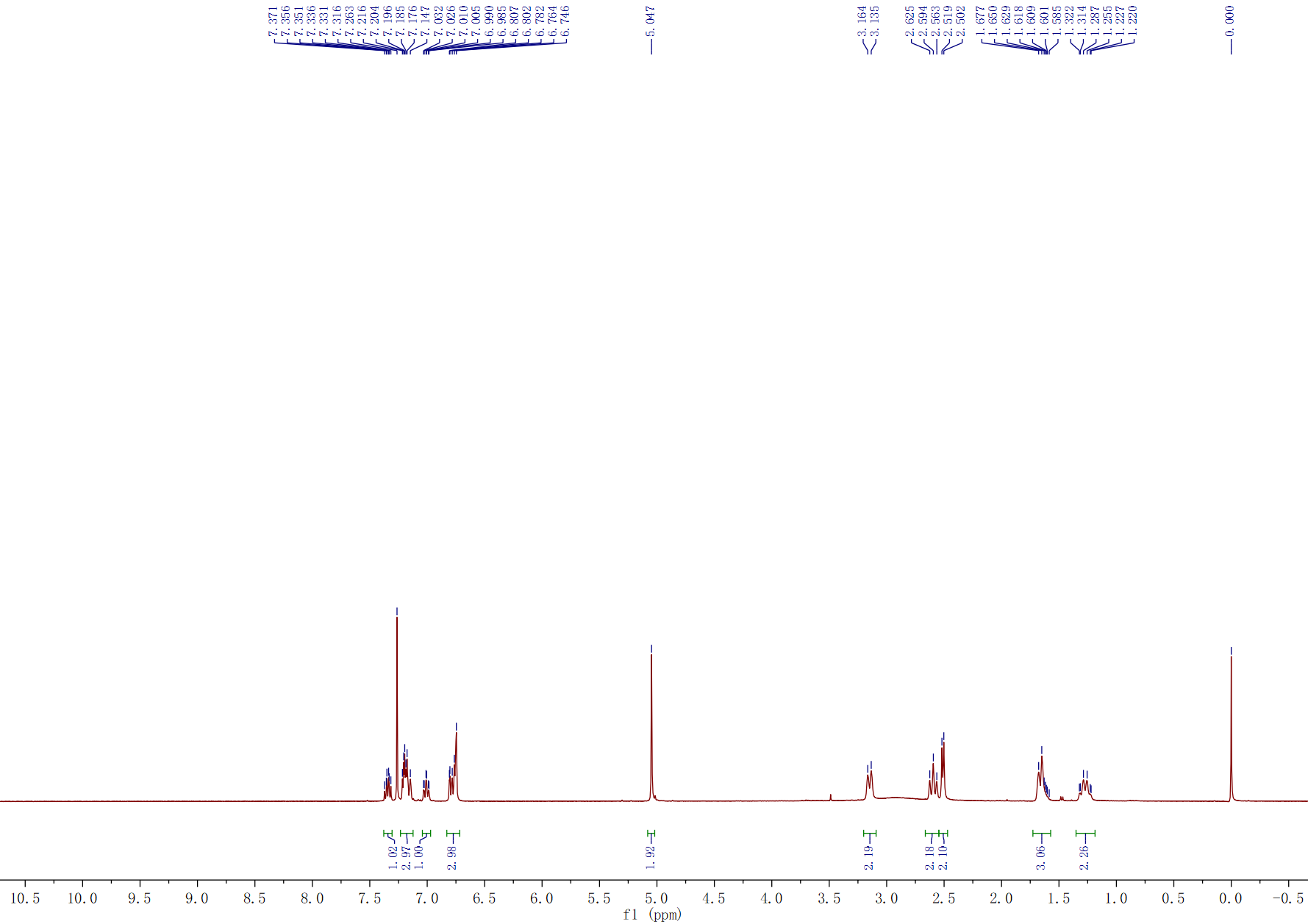


**^1^H NMR spectra of compound 8 (400 MHz, CDCl_3_)**


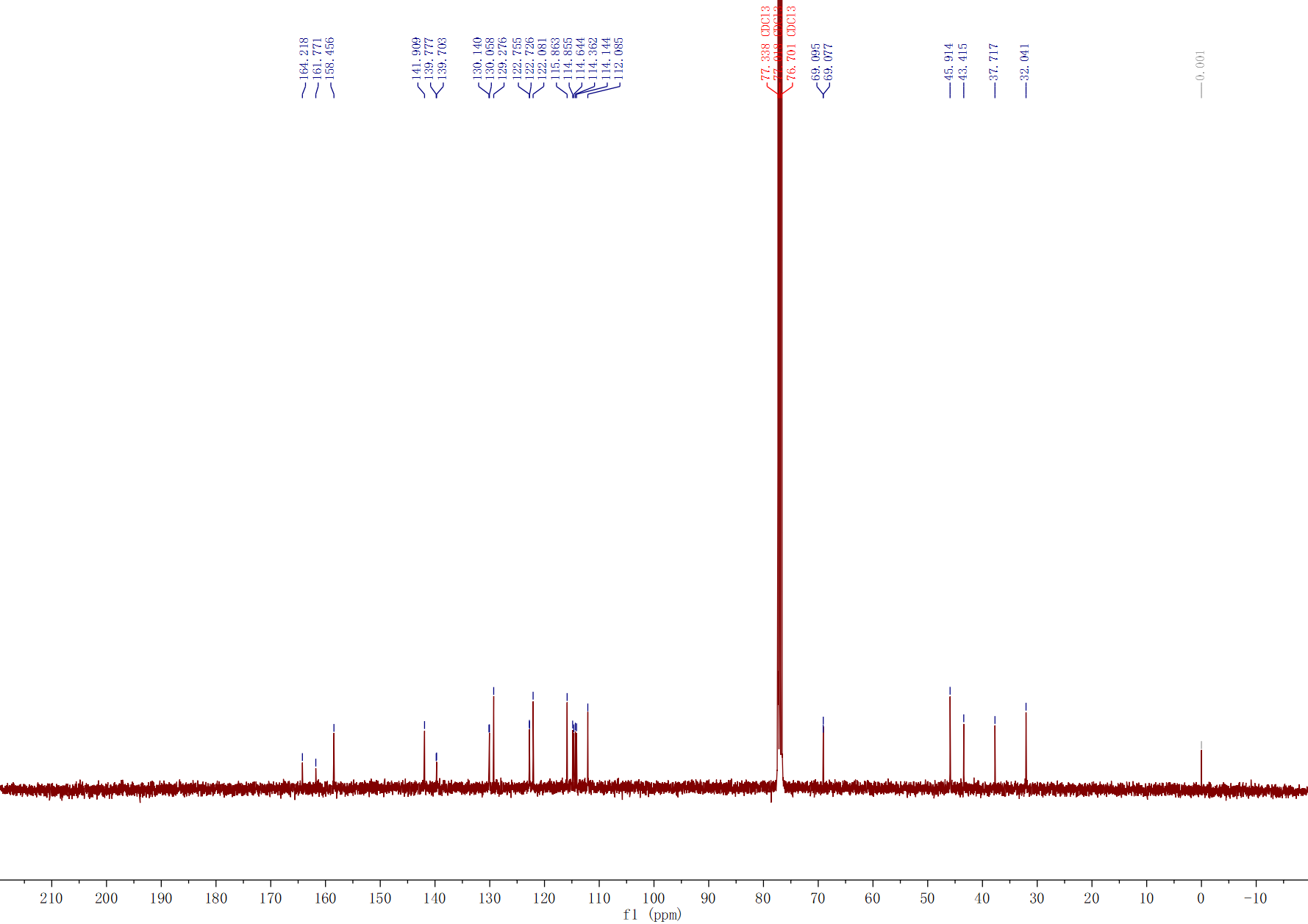


**^13^C NMR of compound 8 (100 MHz, CDCl_3_)**


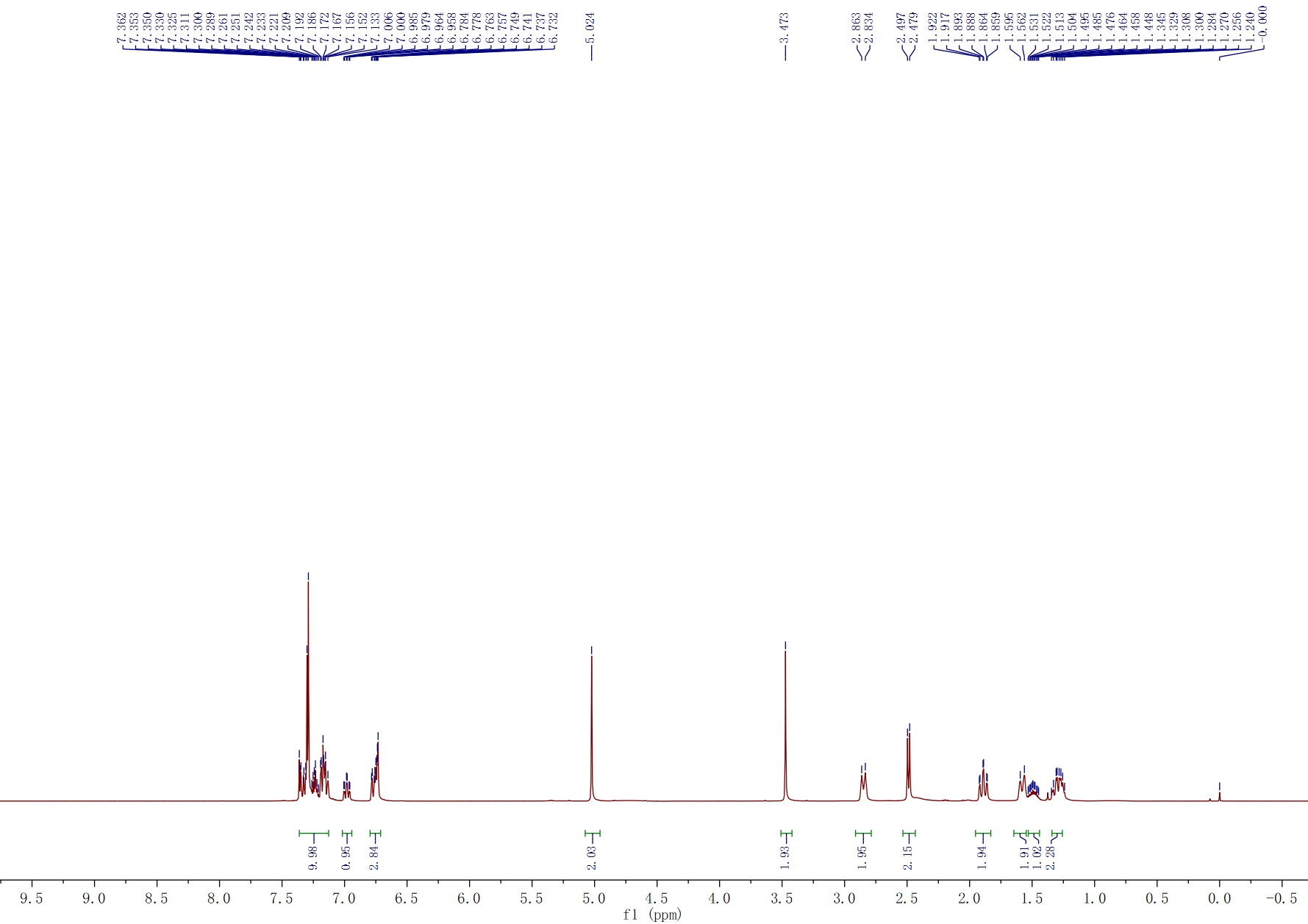


**^1^H NMR spectra of compound 9a (400 MHz, CDCl_3_)**


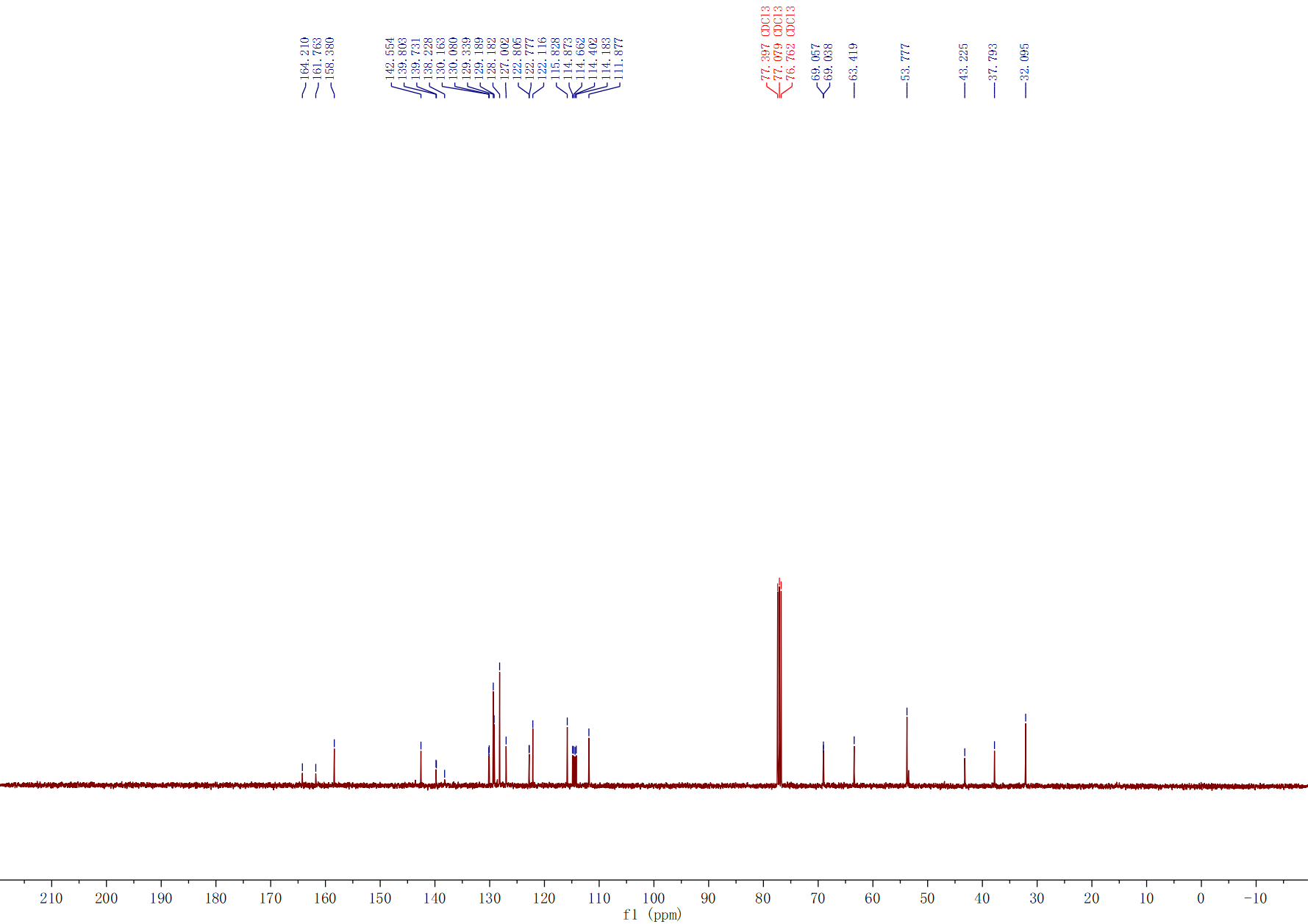


**^13^C NMR of compound 9a (100 MHz, CDCl_3_)**


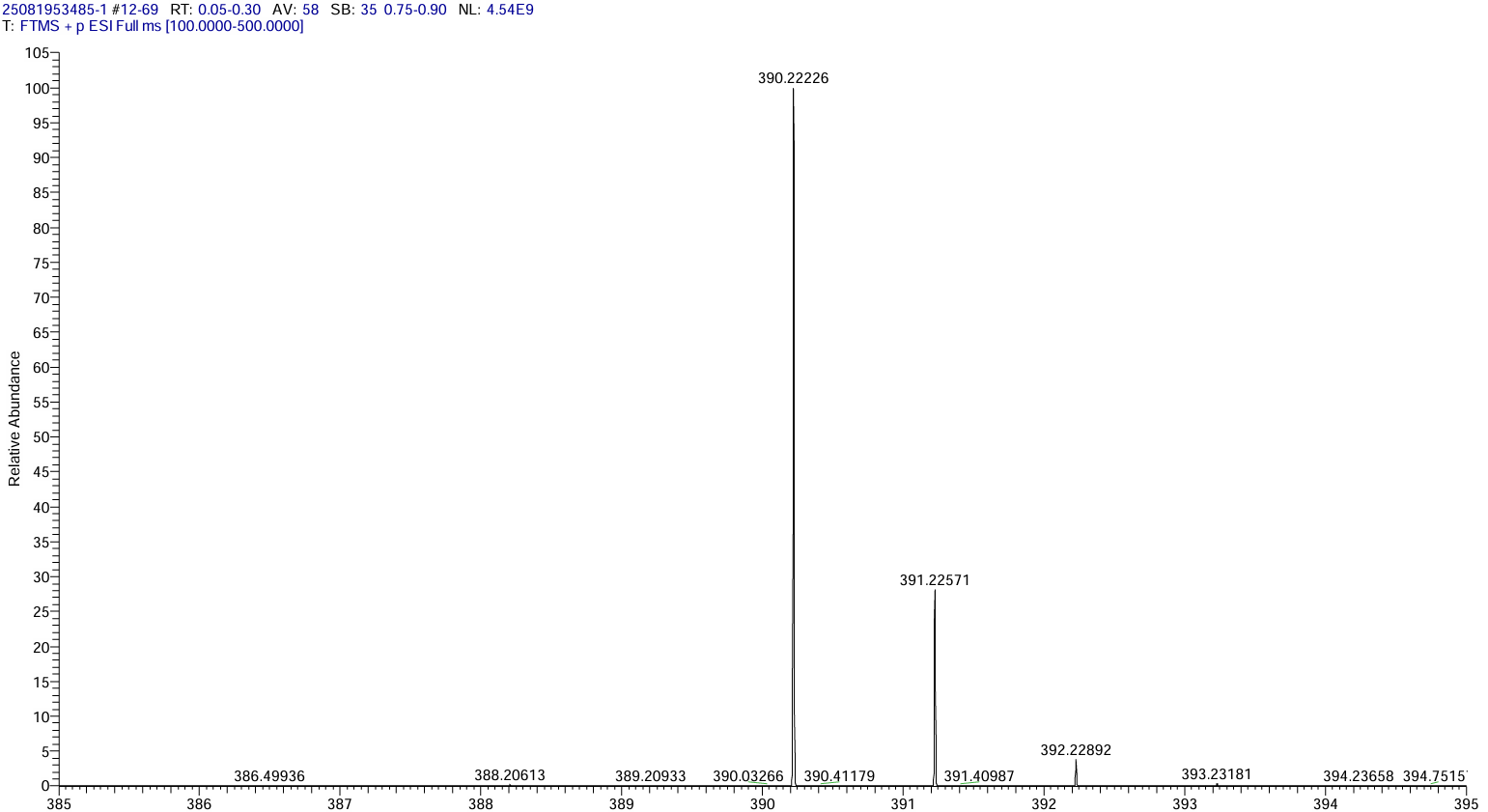


**HR-MS spectra of compound 9a**


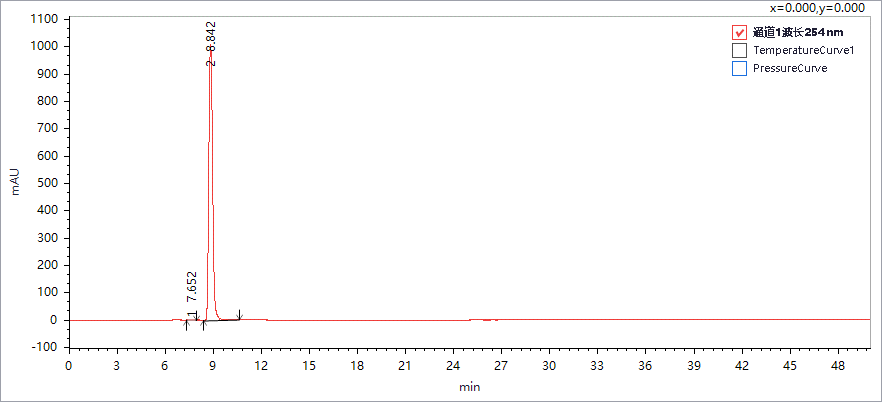


**HPLC purity of compound 9a**


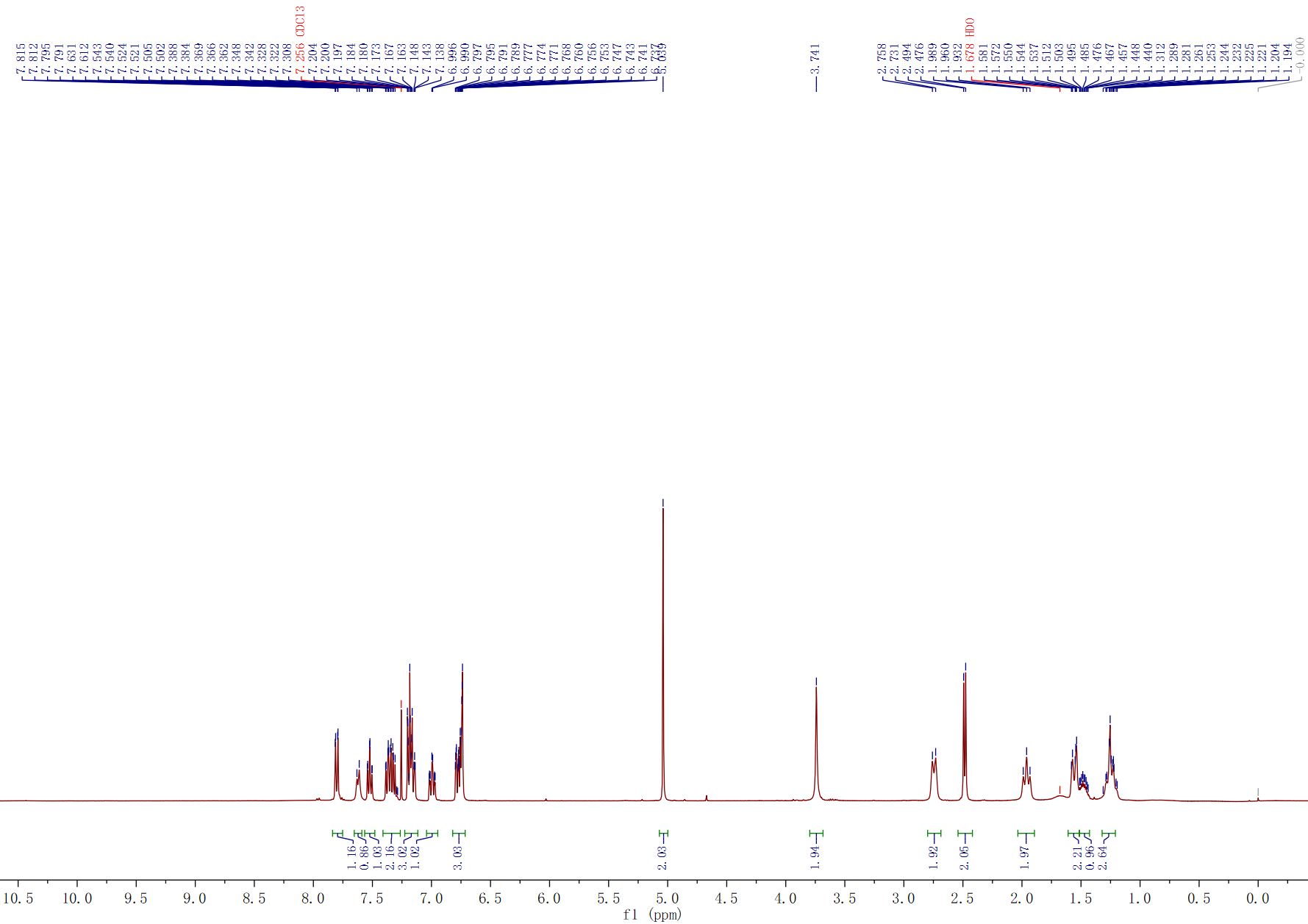


**^1^H NMR spectra of compound 9b (400 MHz, CDCl_3_)**


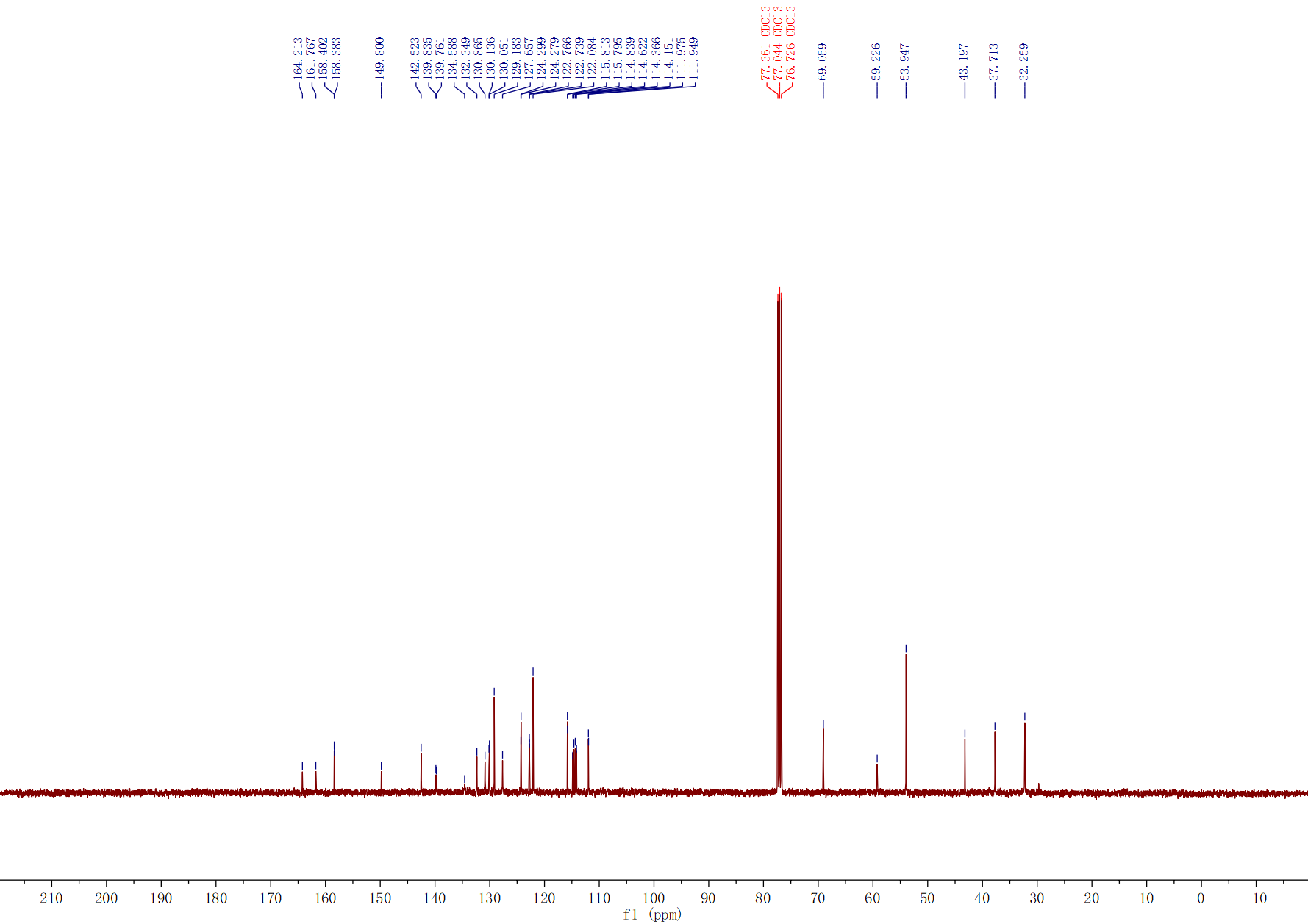


**^13^C NMR of compound 9b (100 MHz, CDCl_3_)**


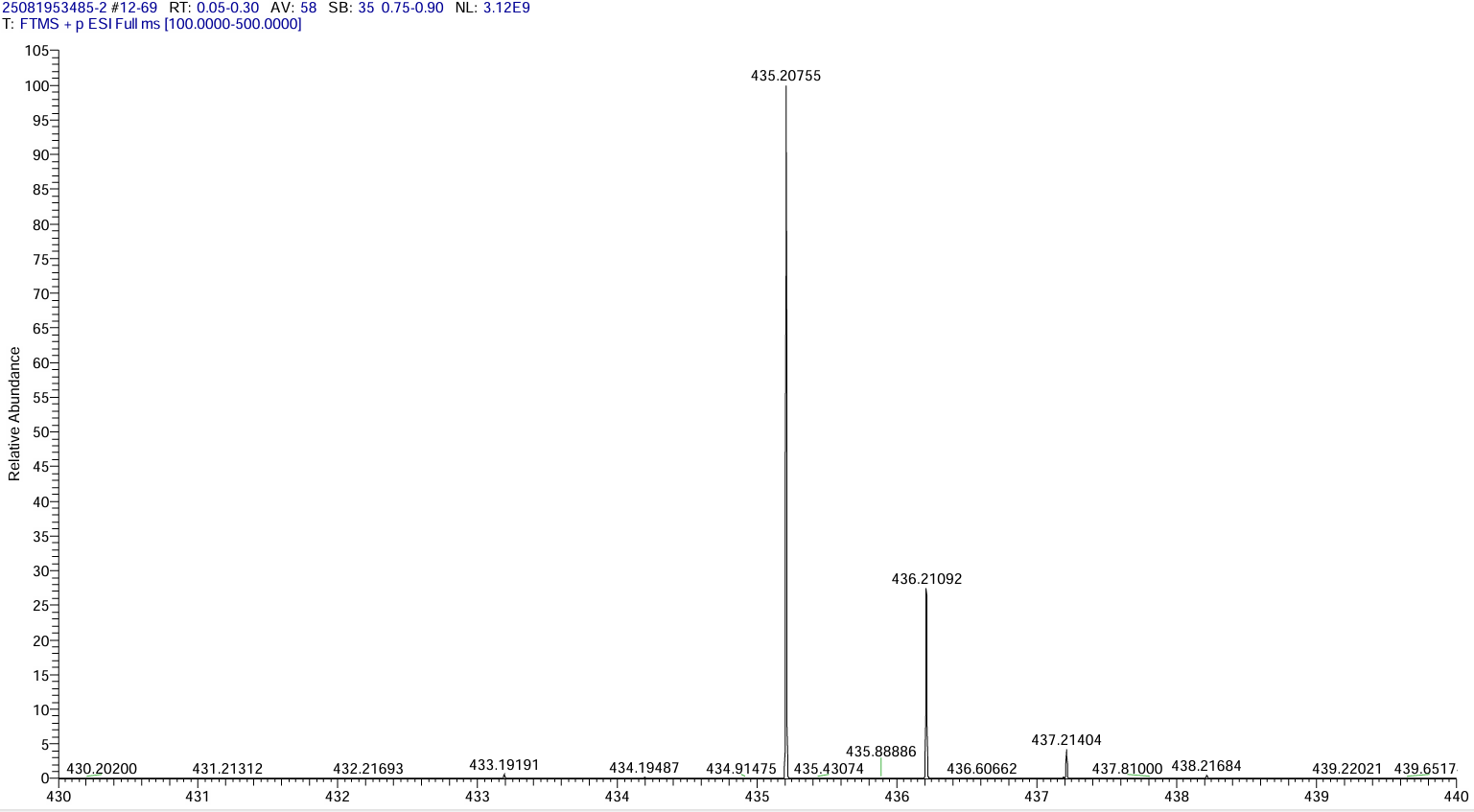


**HR-MS spectra of compound 9b**


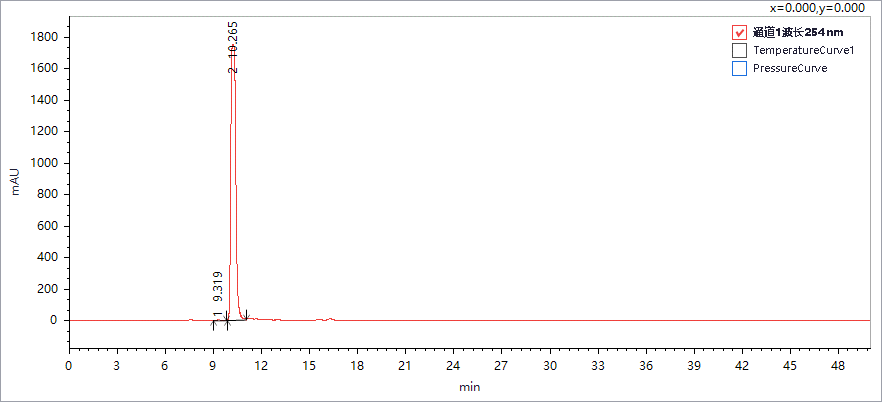


**HPLC purity of compound 9b**


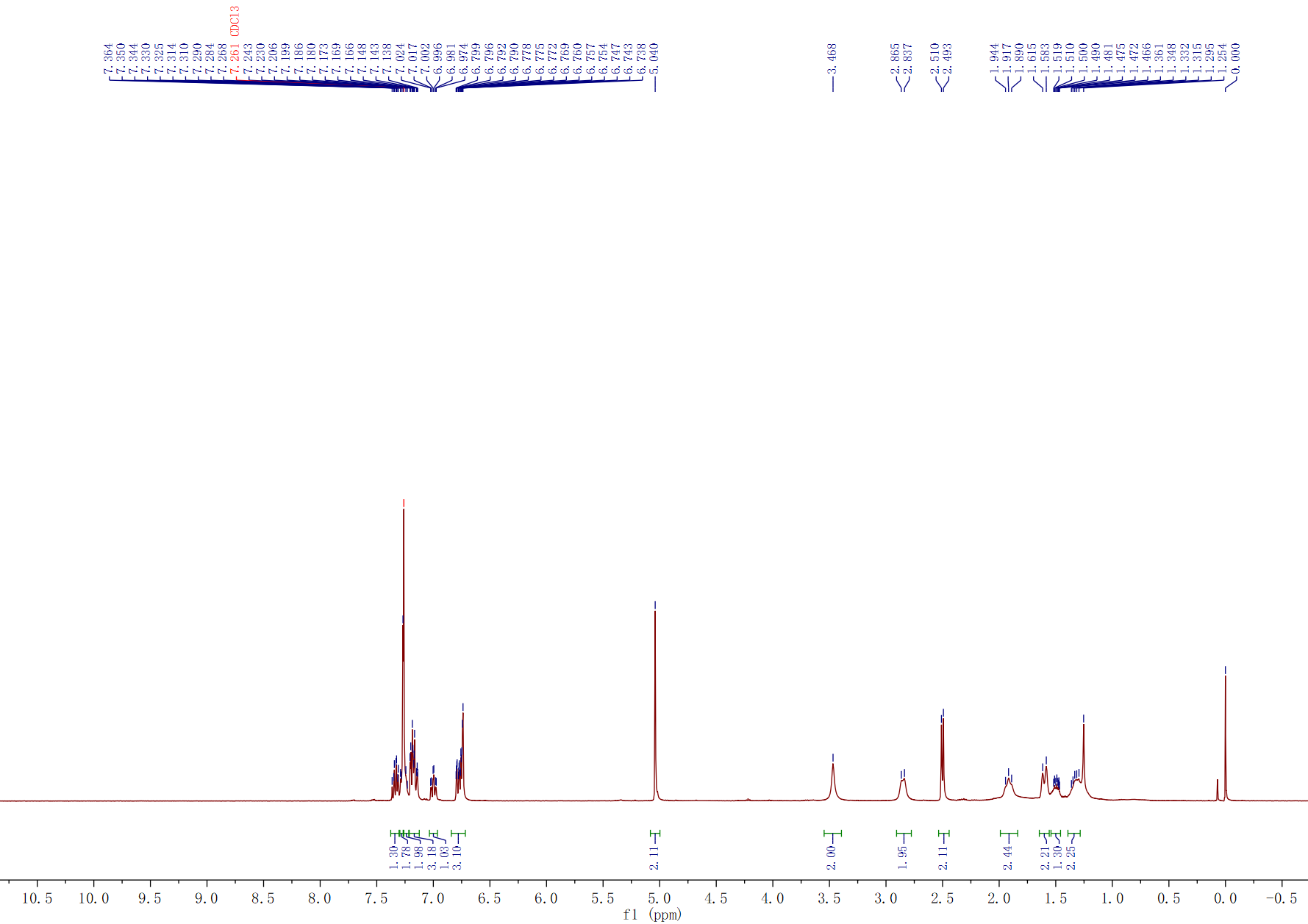


**^1^H NMR spectra of compound 9c (400 MHz, CDCl_3_)**


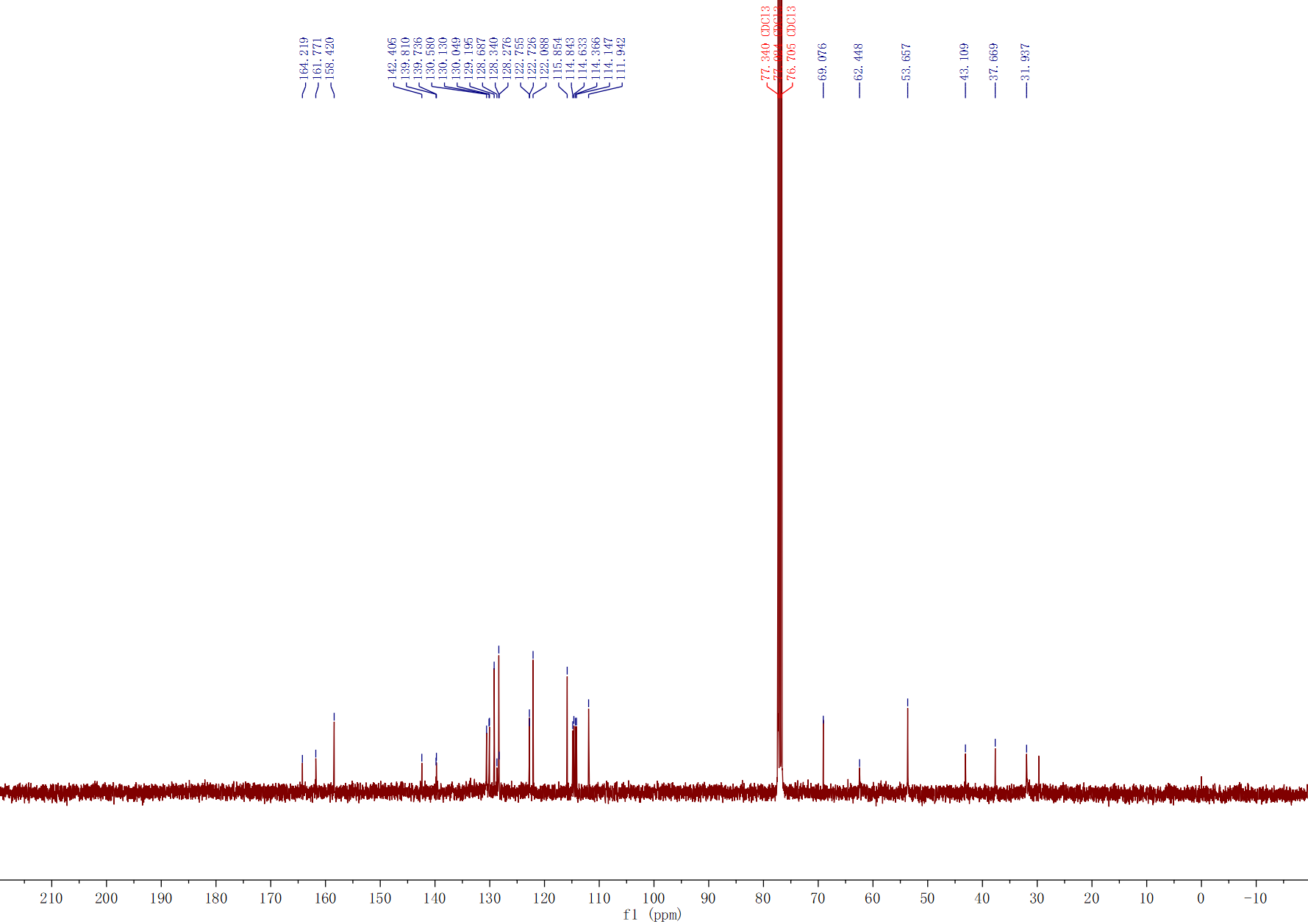


**^13^C NMR of compound 9c (100 MHz, CDCl_3_)**


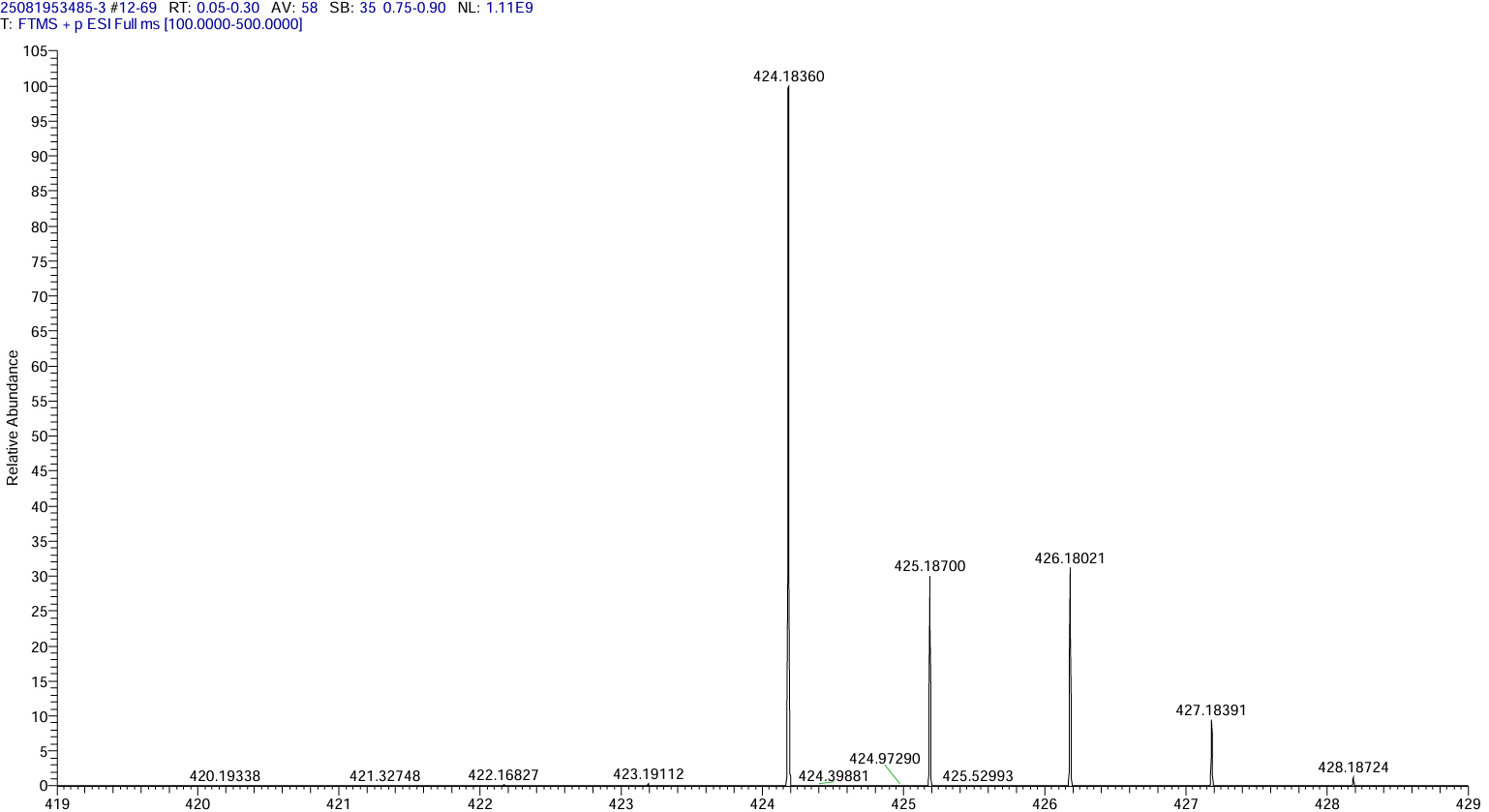


**HR-MS spectra of compound 9c**


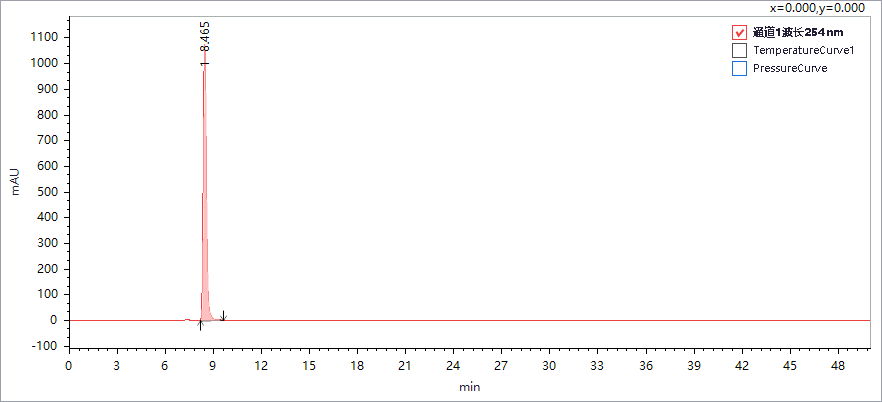


**HPLC purity of compound 9c**


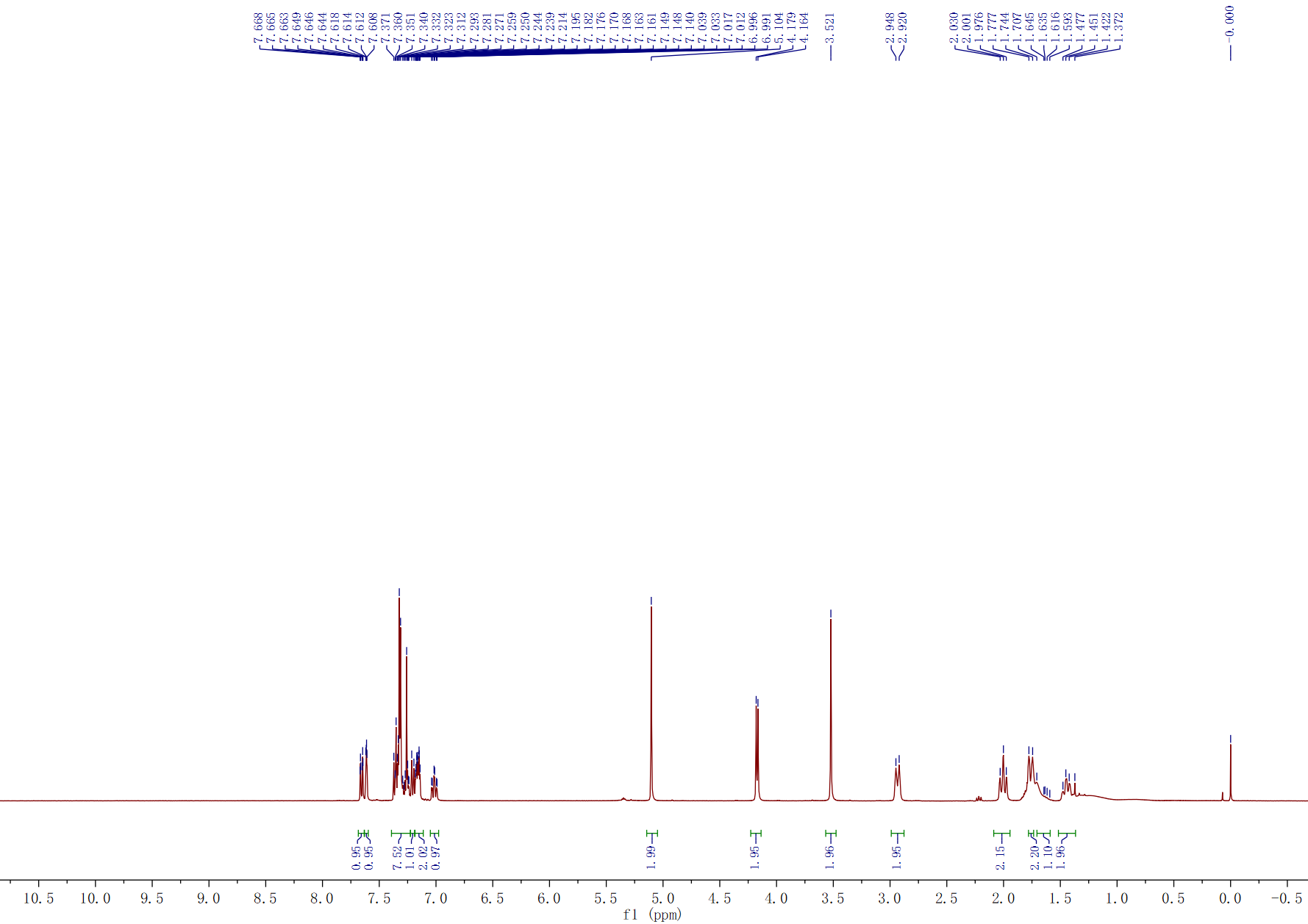


**^1^H NMR spectra of compound 13 (400 MHz, CDCl_3_)**


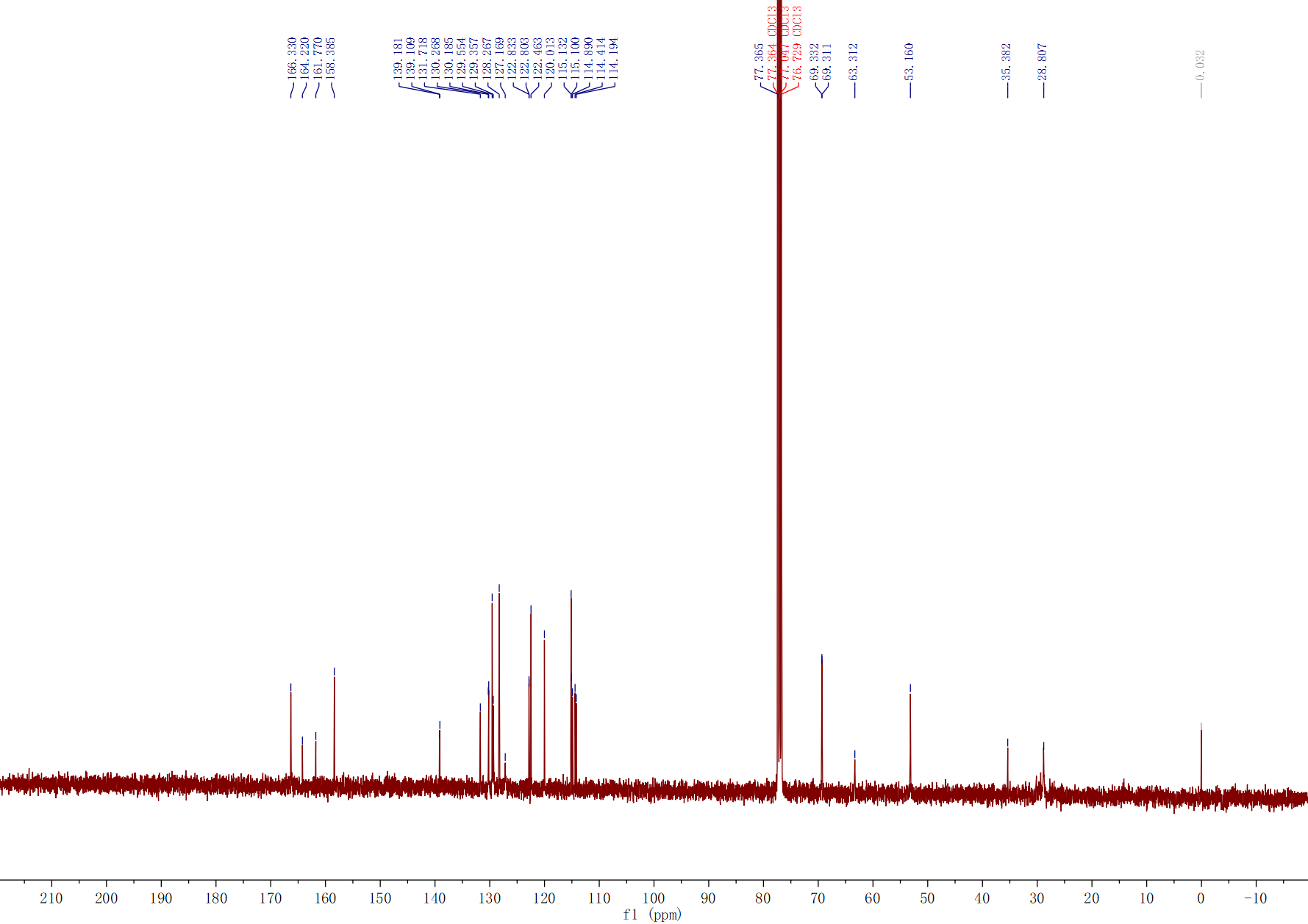


**^13^C NMR of compound 13 (100 MHz, CDCl_3_)**


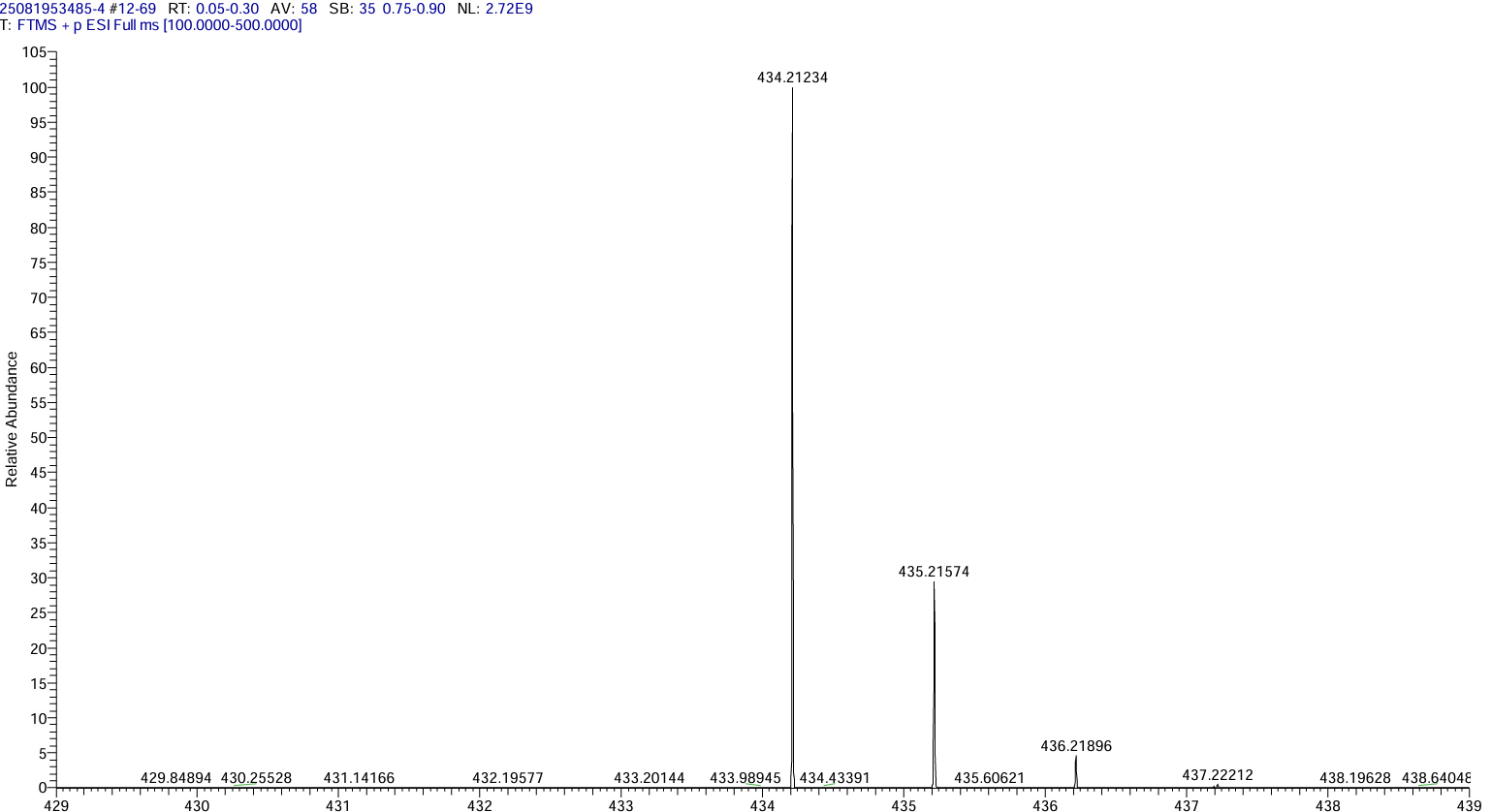


**HR-MS spectra of compound 13**


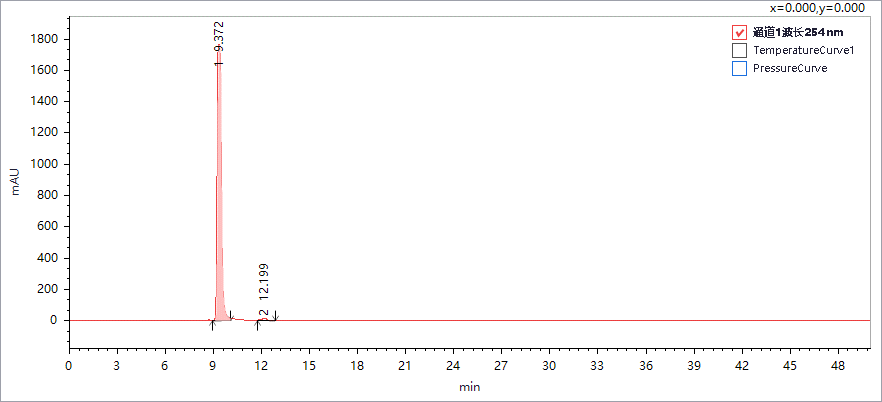


**HPLC purity of compound 13**


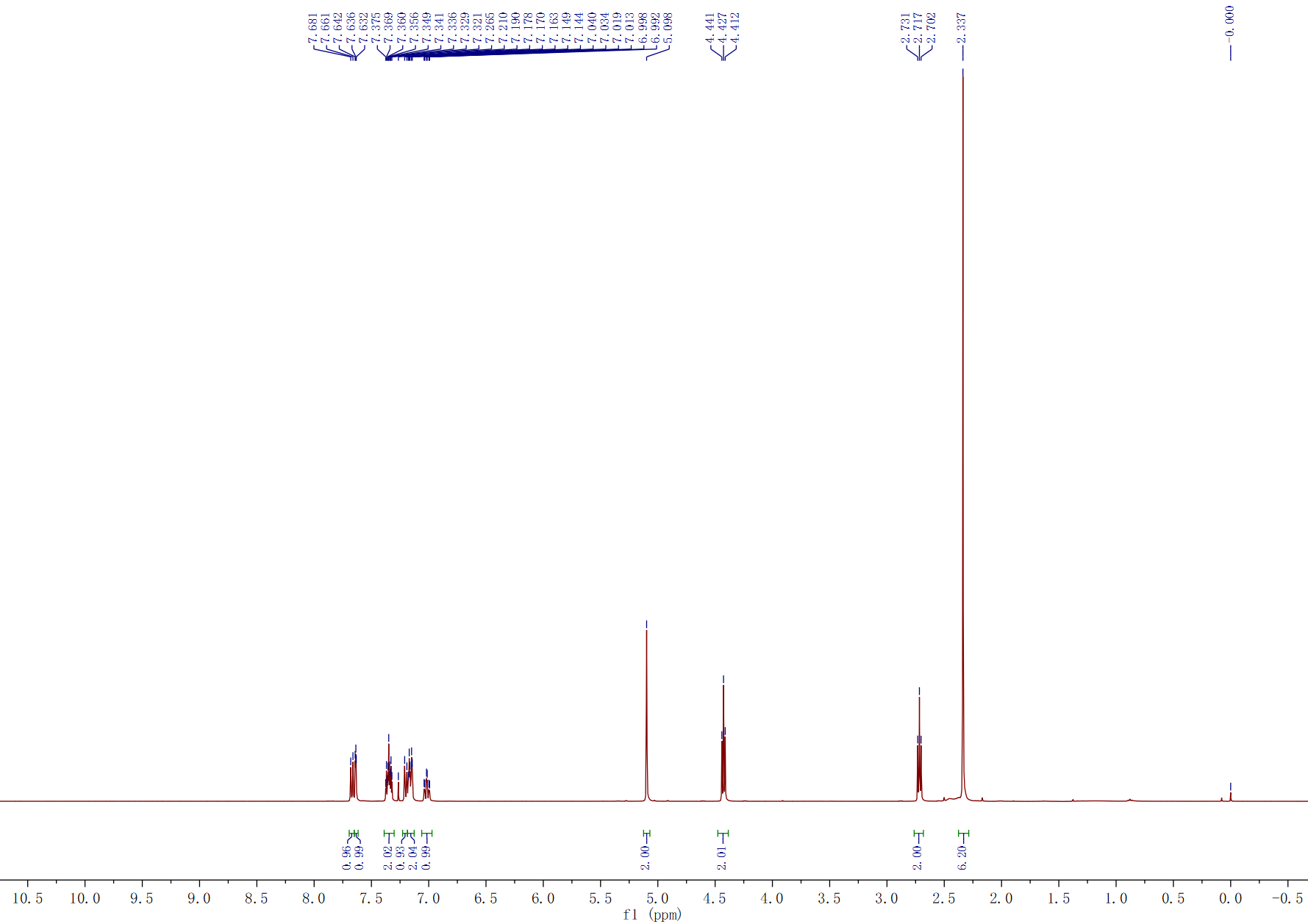


**^1^H NMR spectra of compound 14a (400 MHz, CDCl_3_)**


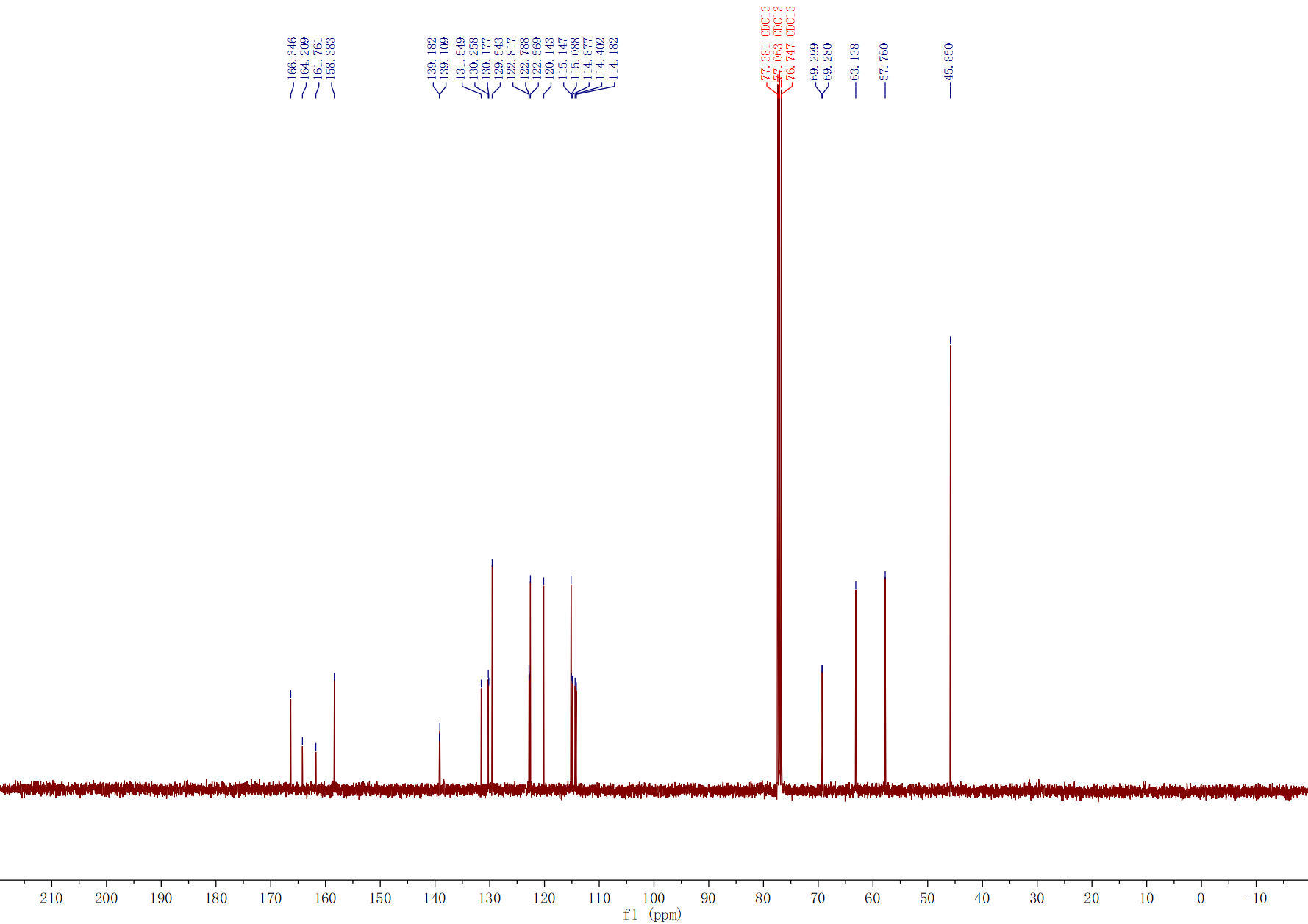


**^13^C NMR of compound 14a (100 MHz, CDCl_3_)**


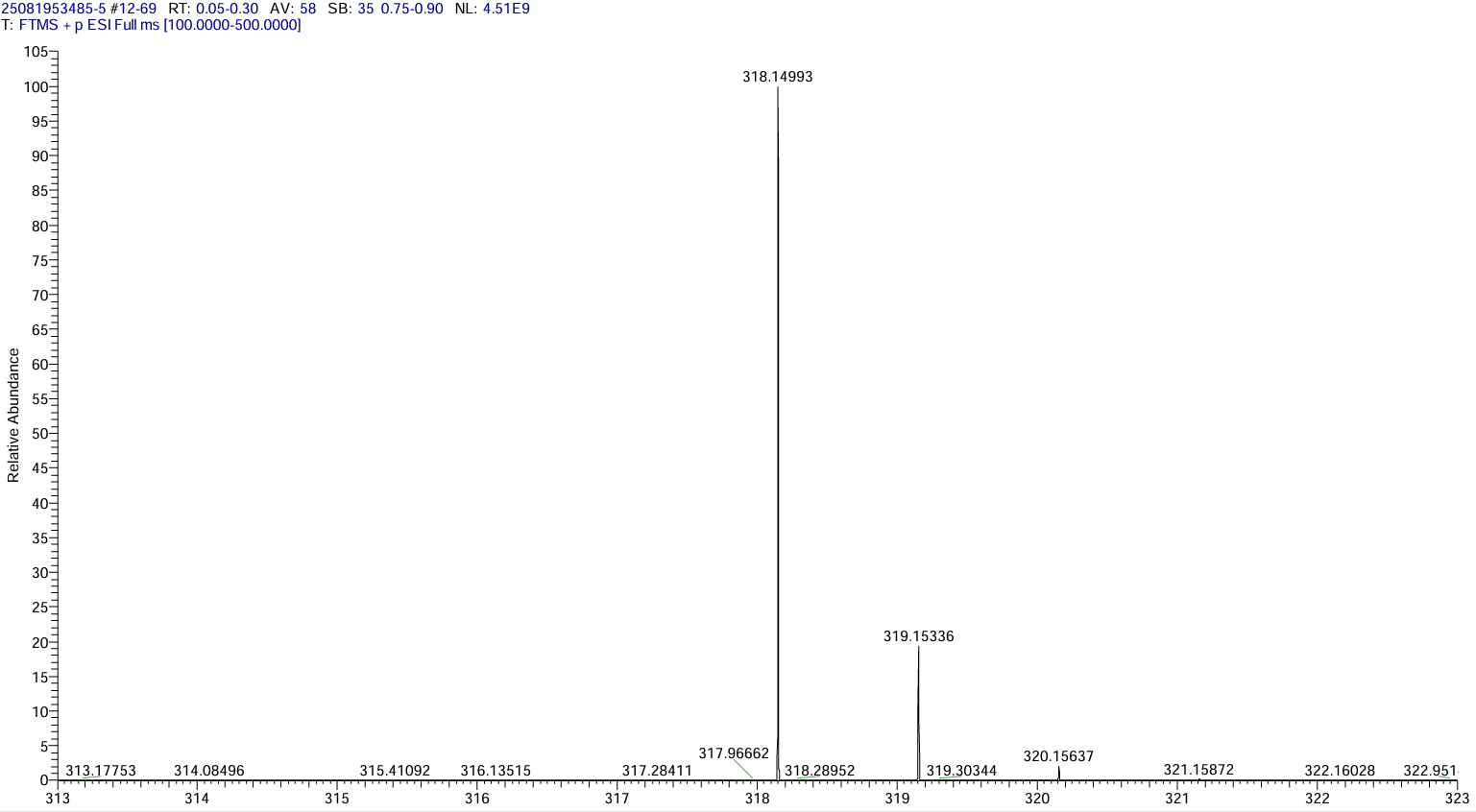


**HR-MS spectra of compound 14a**


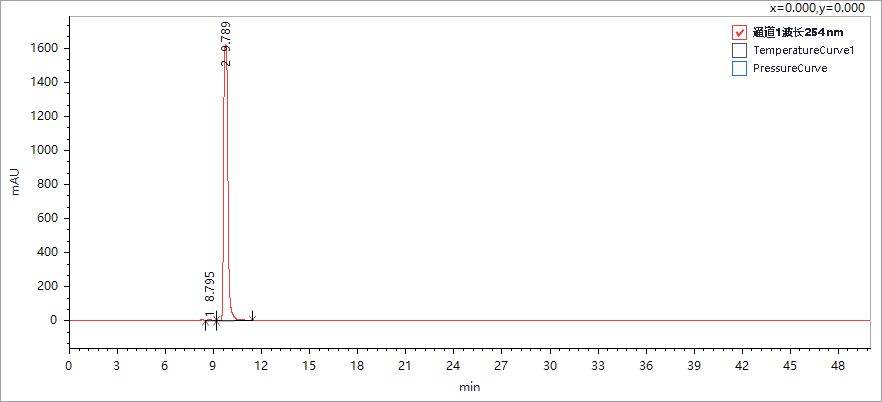


**HPLC purity of compound 14a**


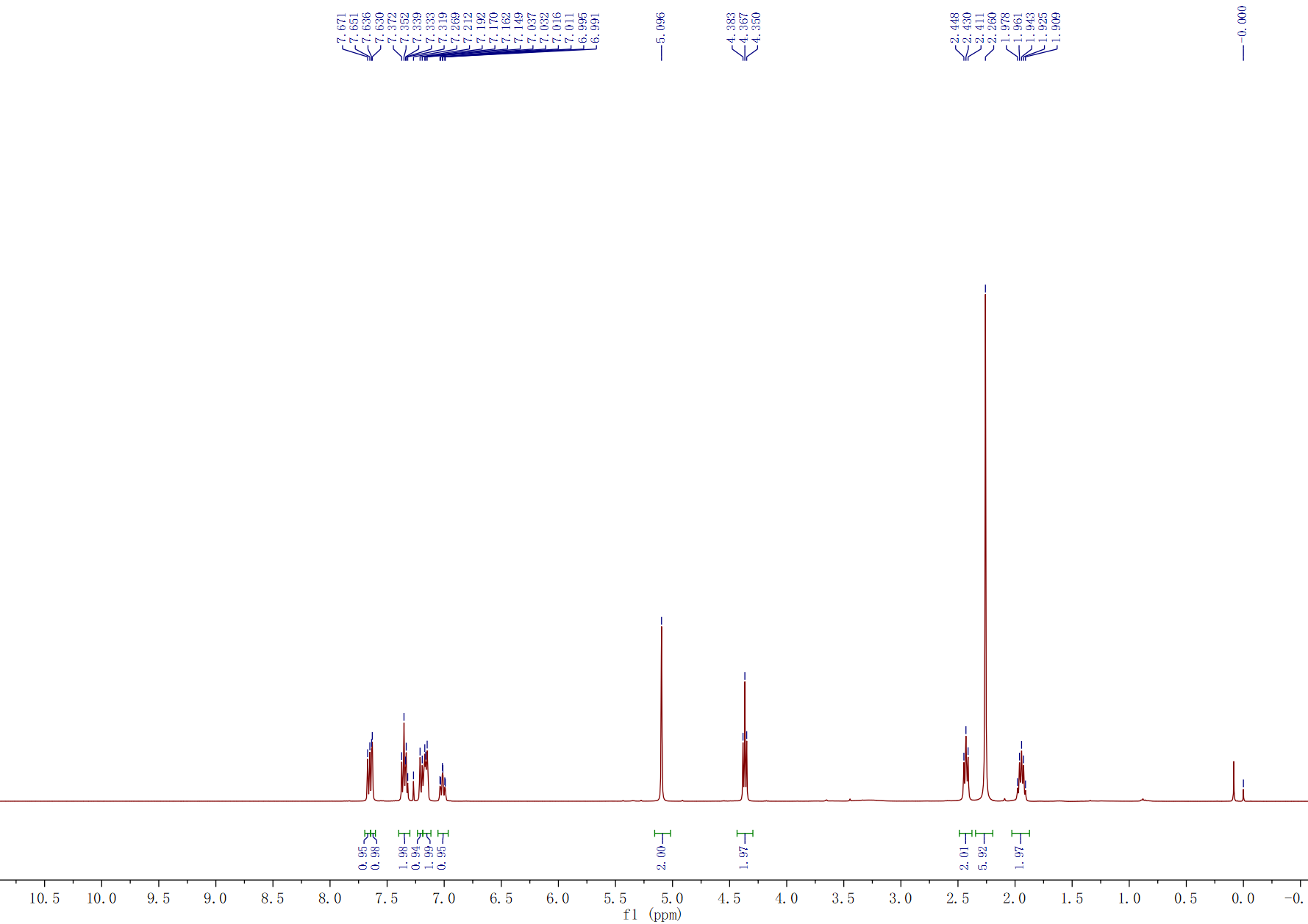


**^1^H NMR spectra of compound 15a (400 MHz, CDCl_3_)**


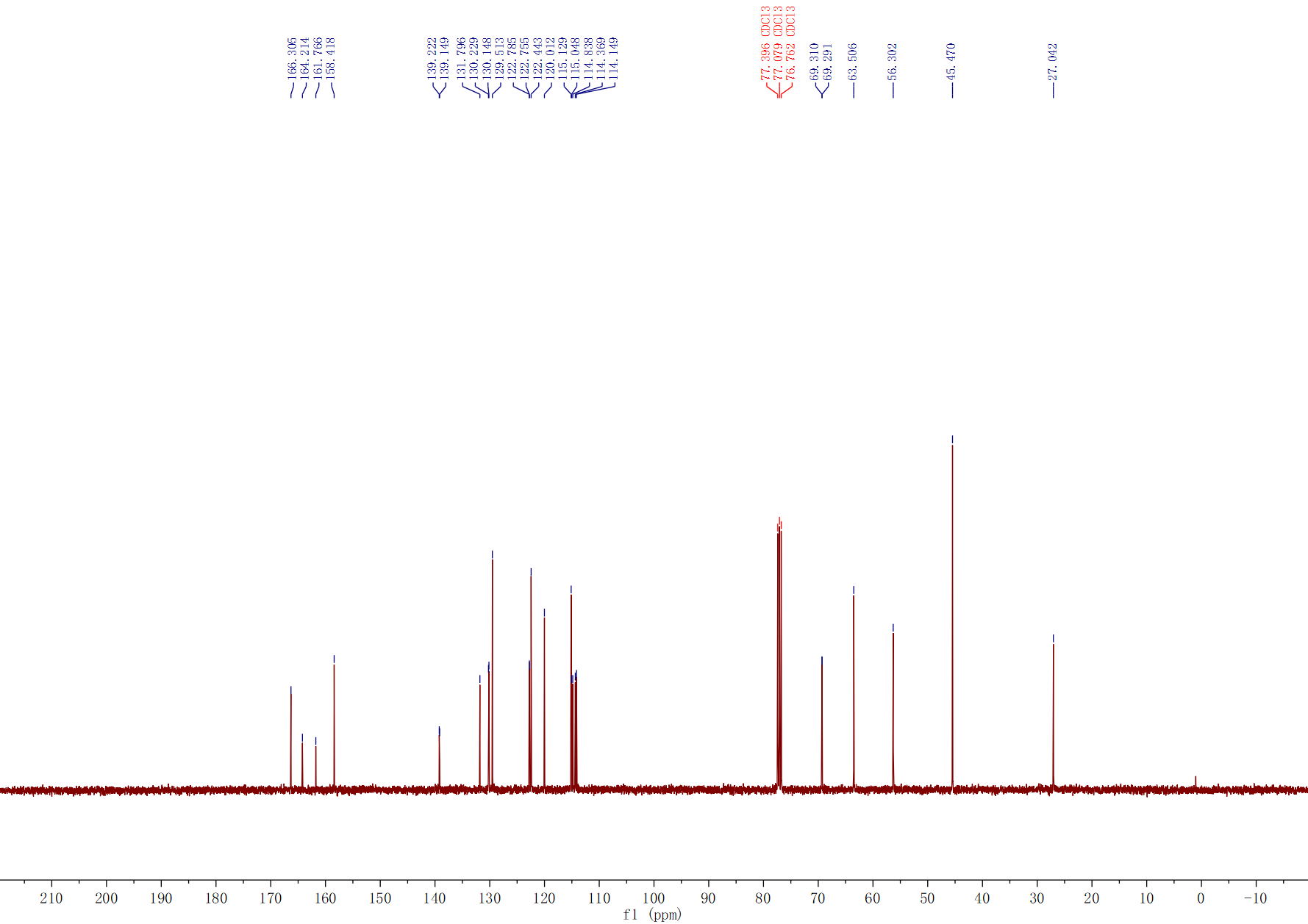


**^13^C NMR of compound 15a (100 MHz, CDCl_3_)**


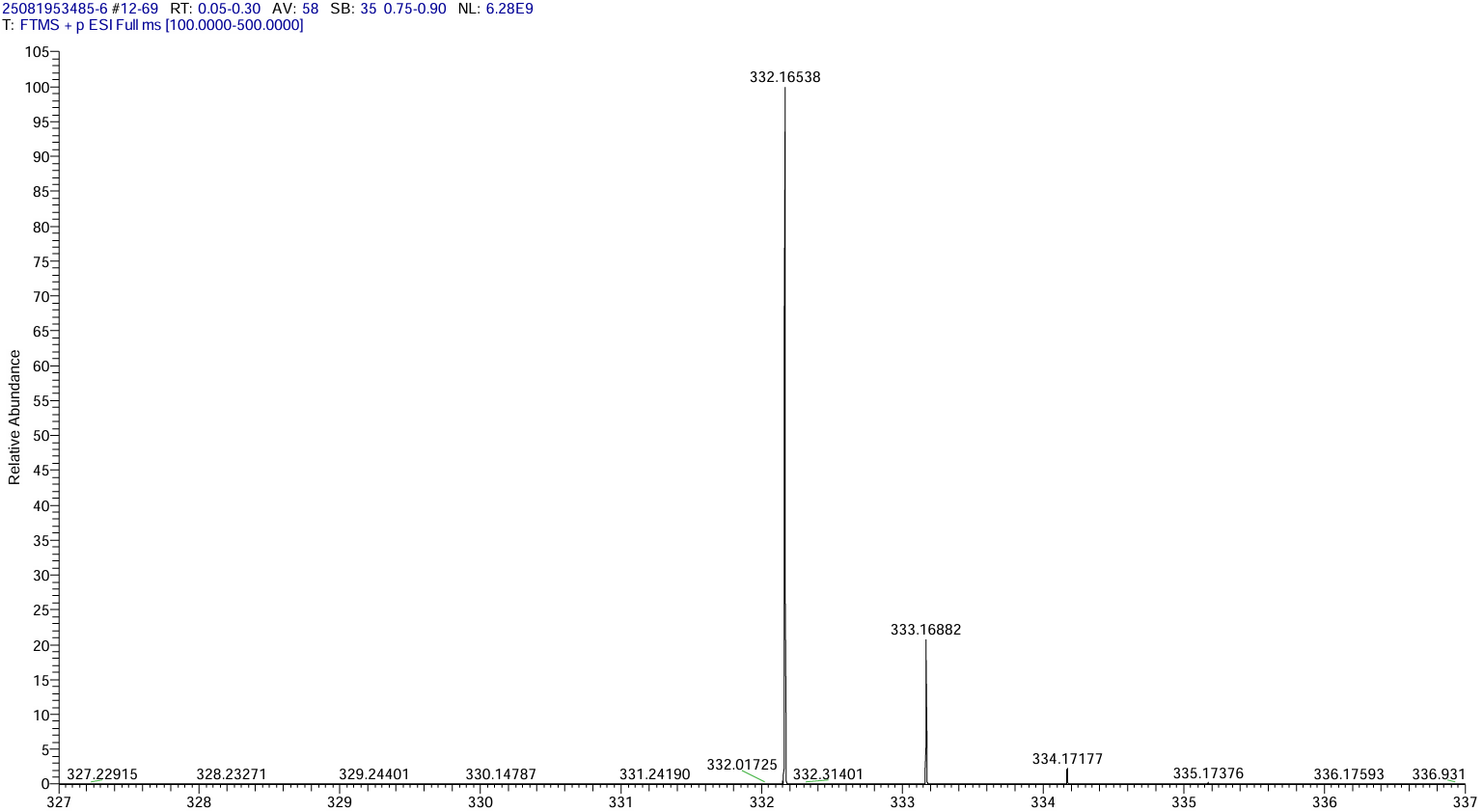


**HR-MS spectra of compound 15a**


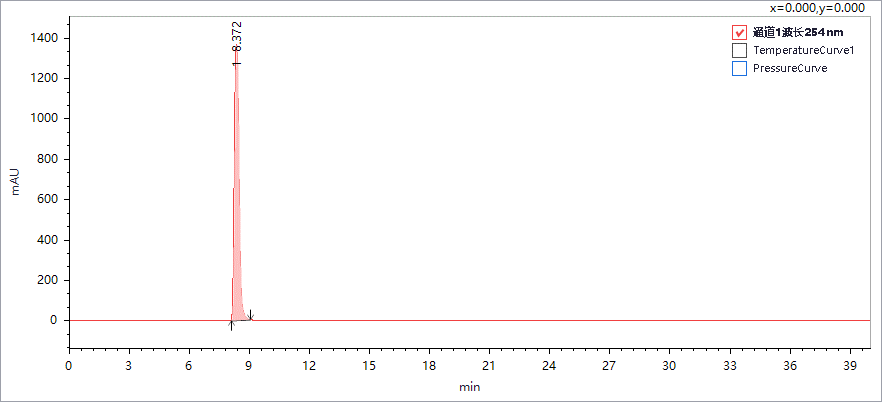


**HPLC purity of compound 15a**


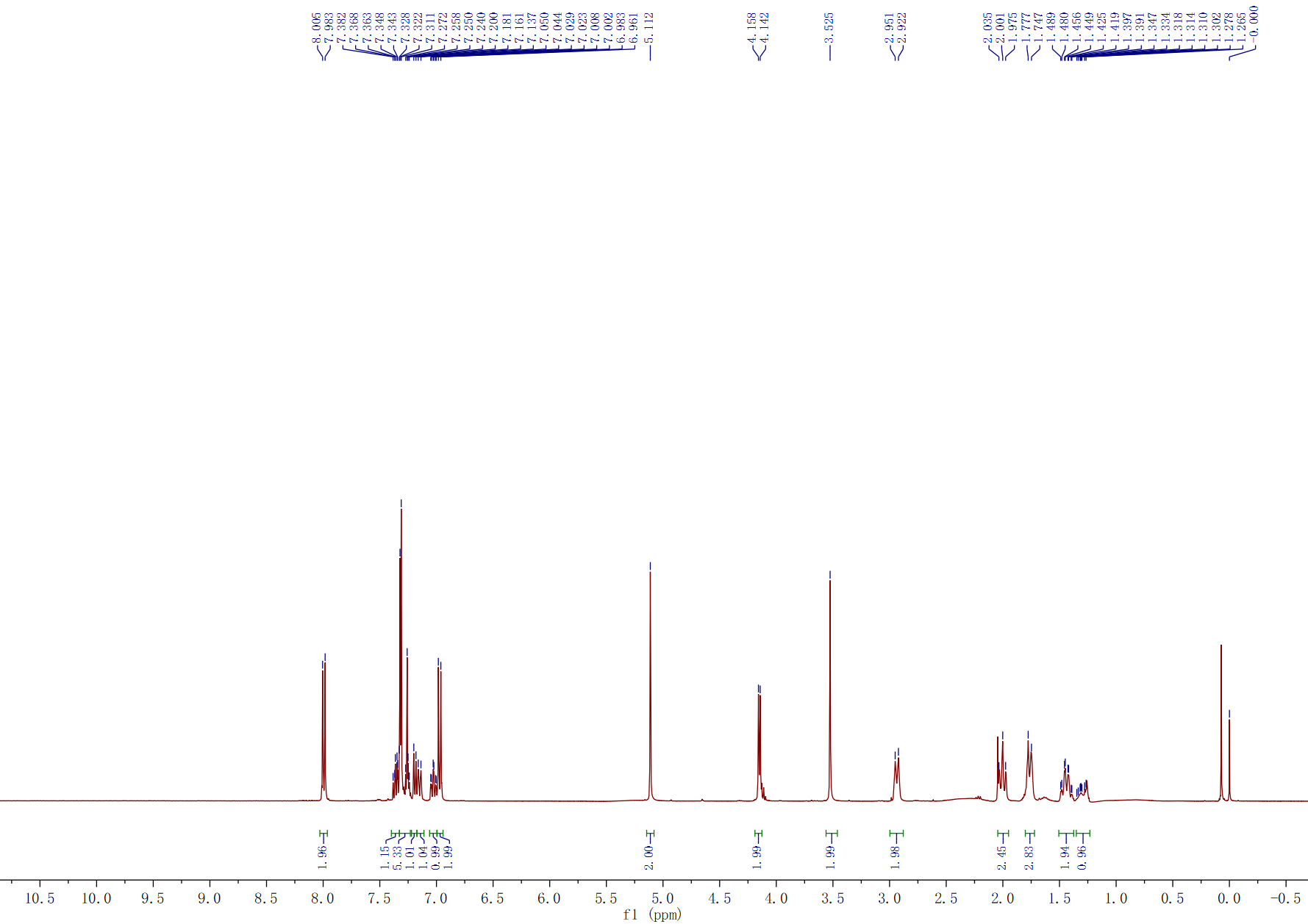


**^1^H NMR spectra of compound 19 (400 MHz, CDCl_3_)**


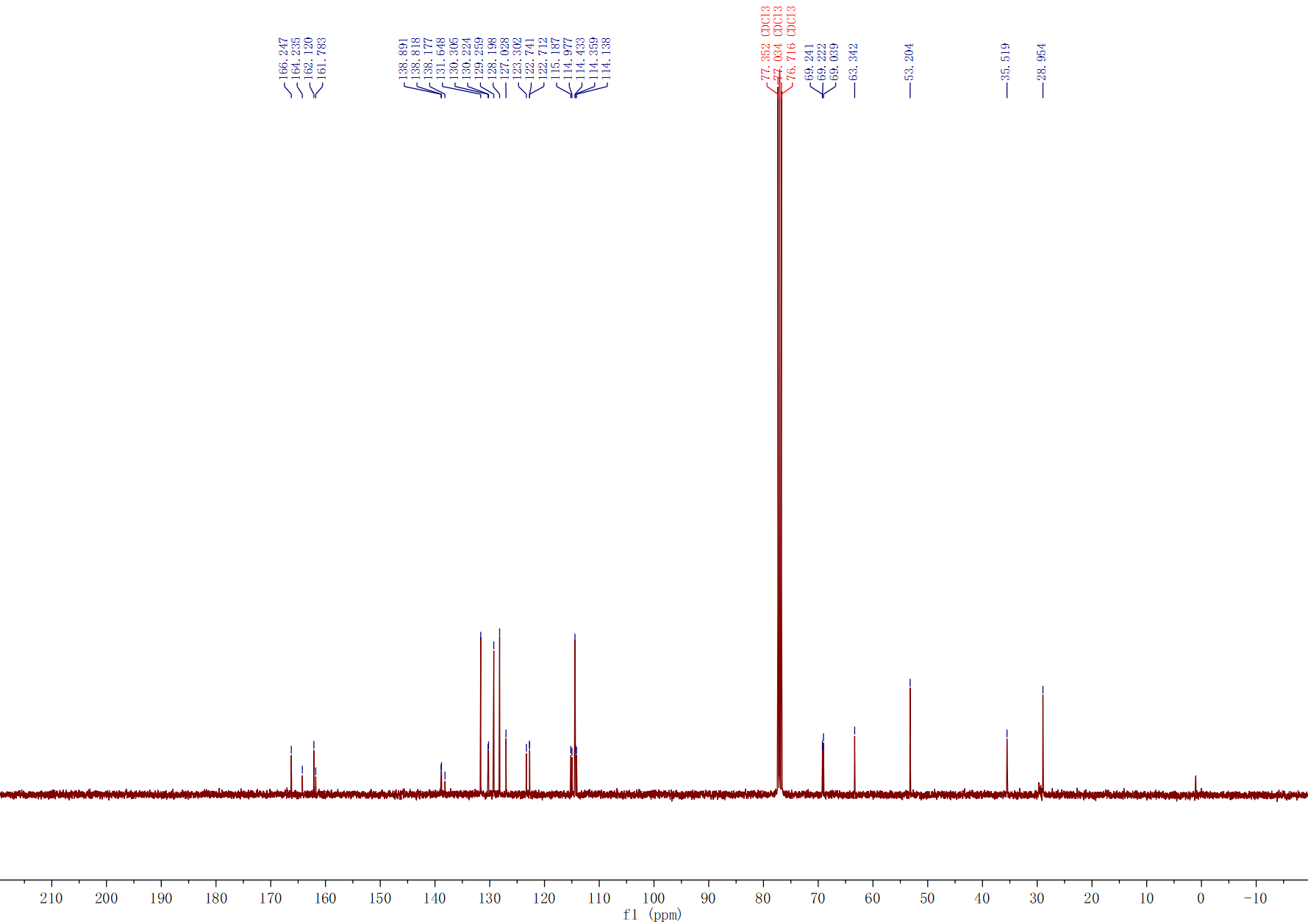


**^13^C NMR of compound 19 (100 MHz, CDCl_3_)**


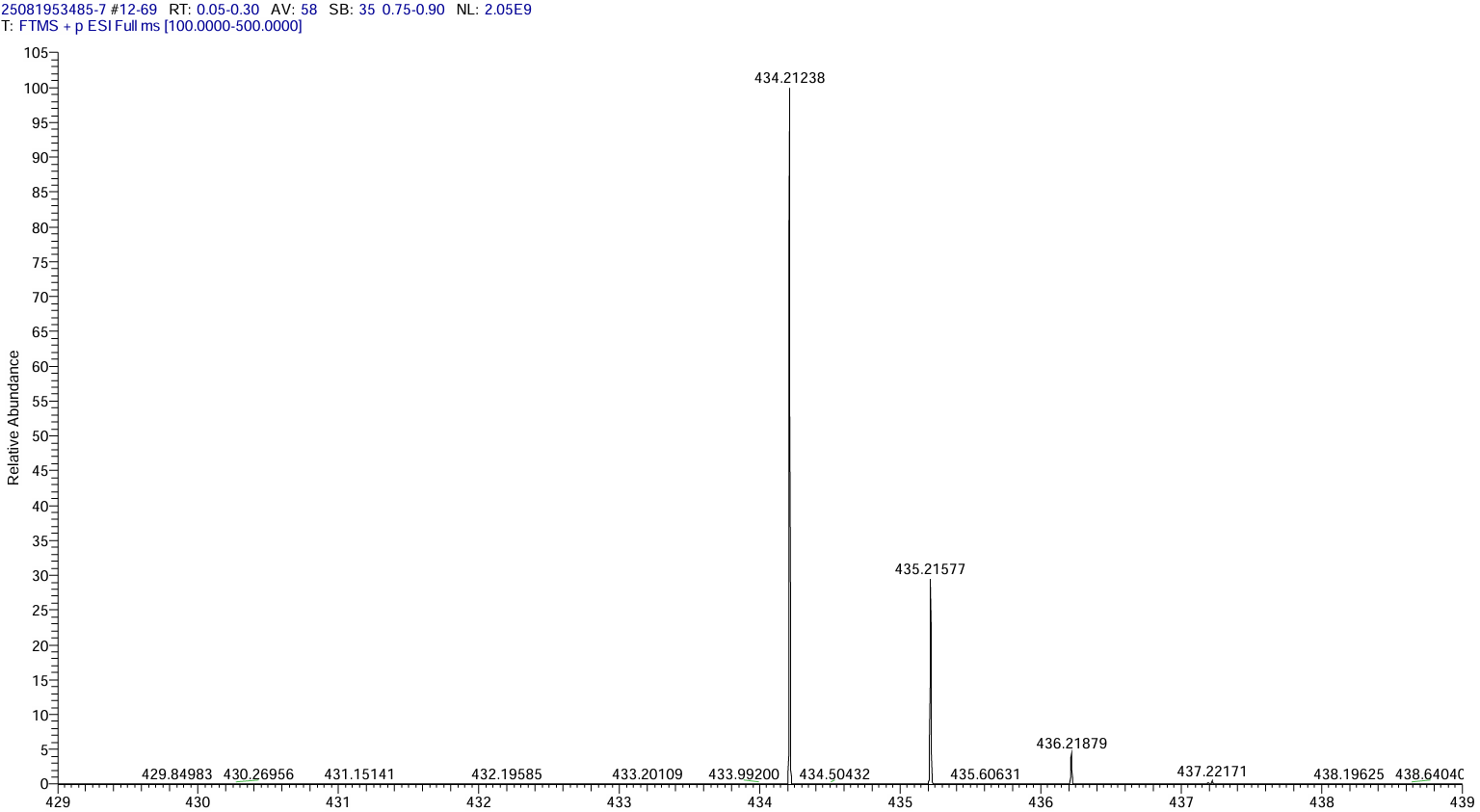


**HR-MS spectra of compound 19**


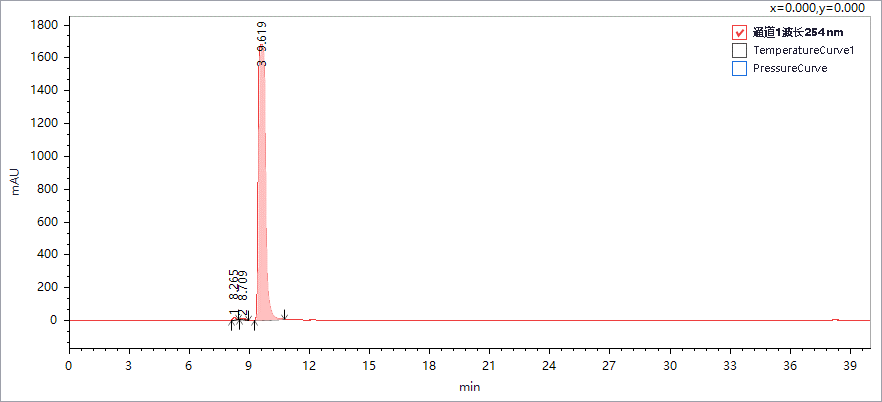


**HPLC purity of compound 19**


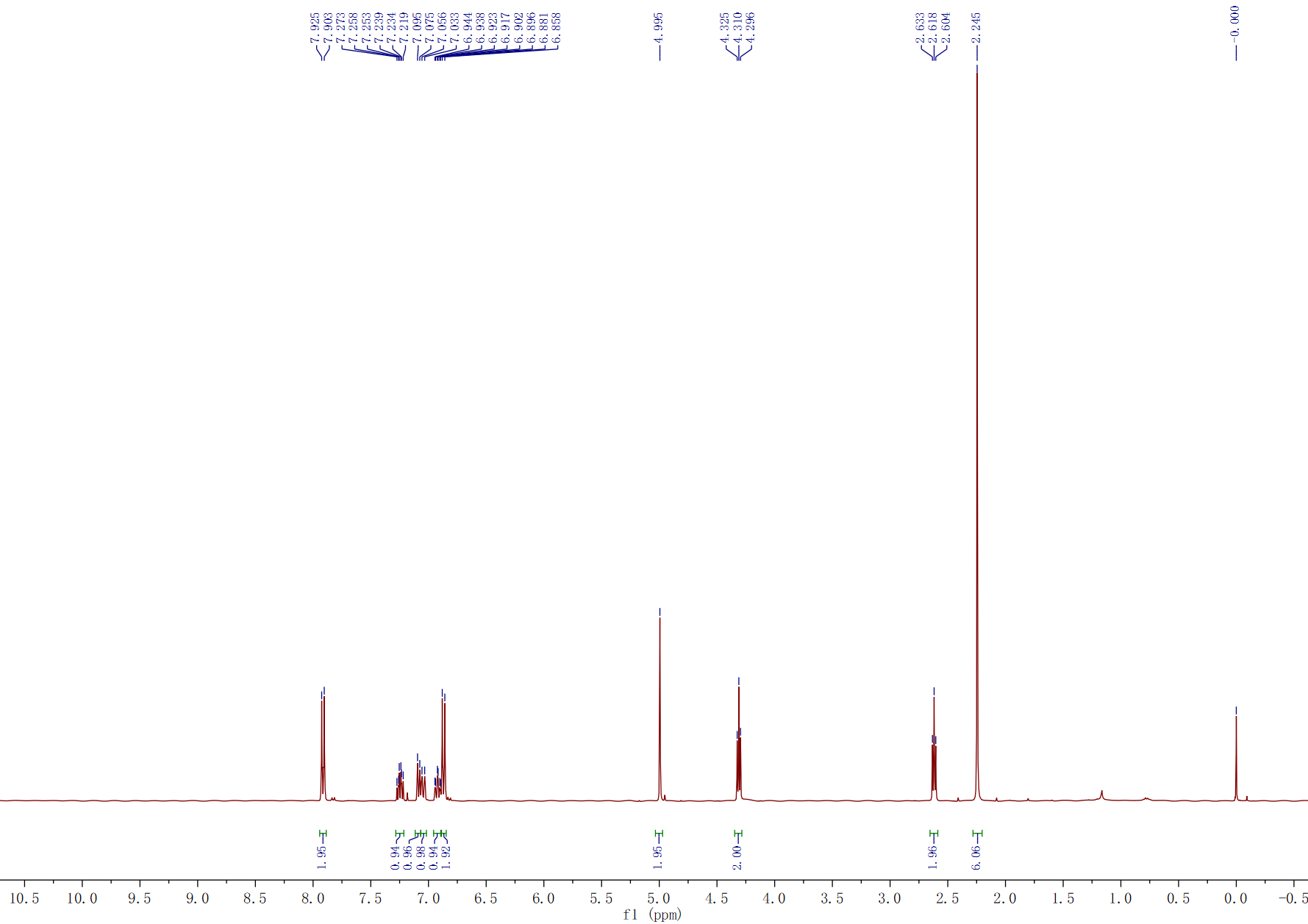


**^1^H NMR spectra of compound 20a (400 MHz, CDCl_3_)**


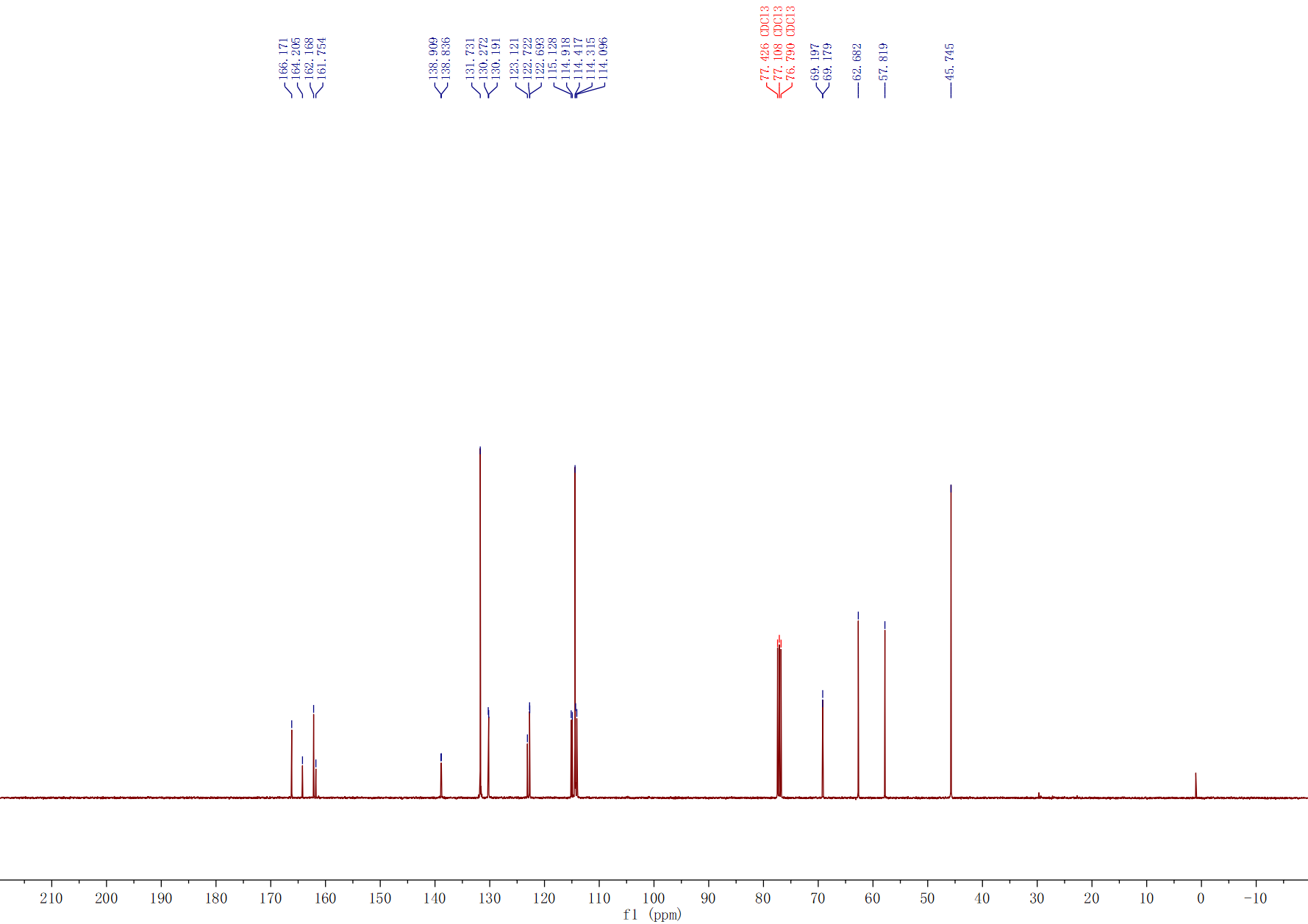


**^13^C NMR of compound 20a (100 MHz, CDCl_3_)**


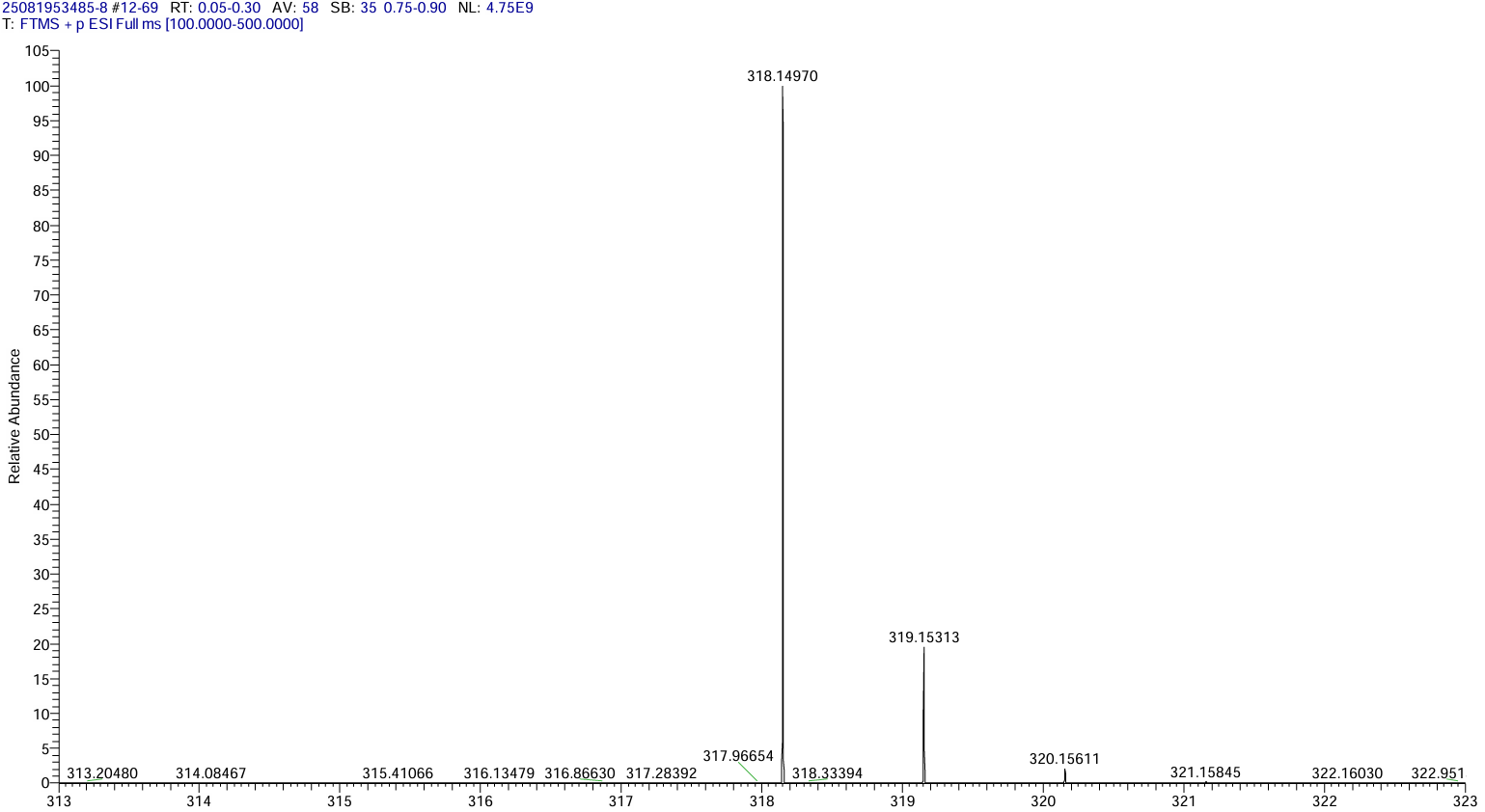


**HR-MS spectra of compound 20a**


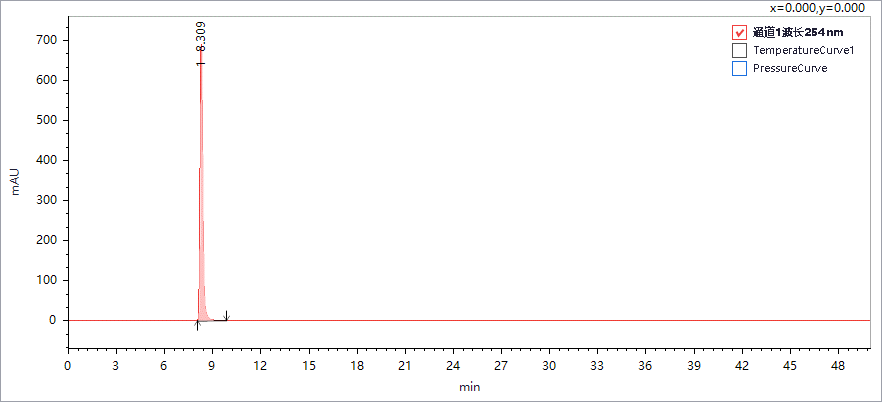


**HPLC purity of compound 20a**


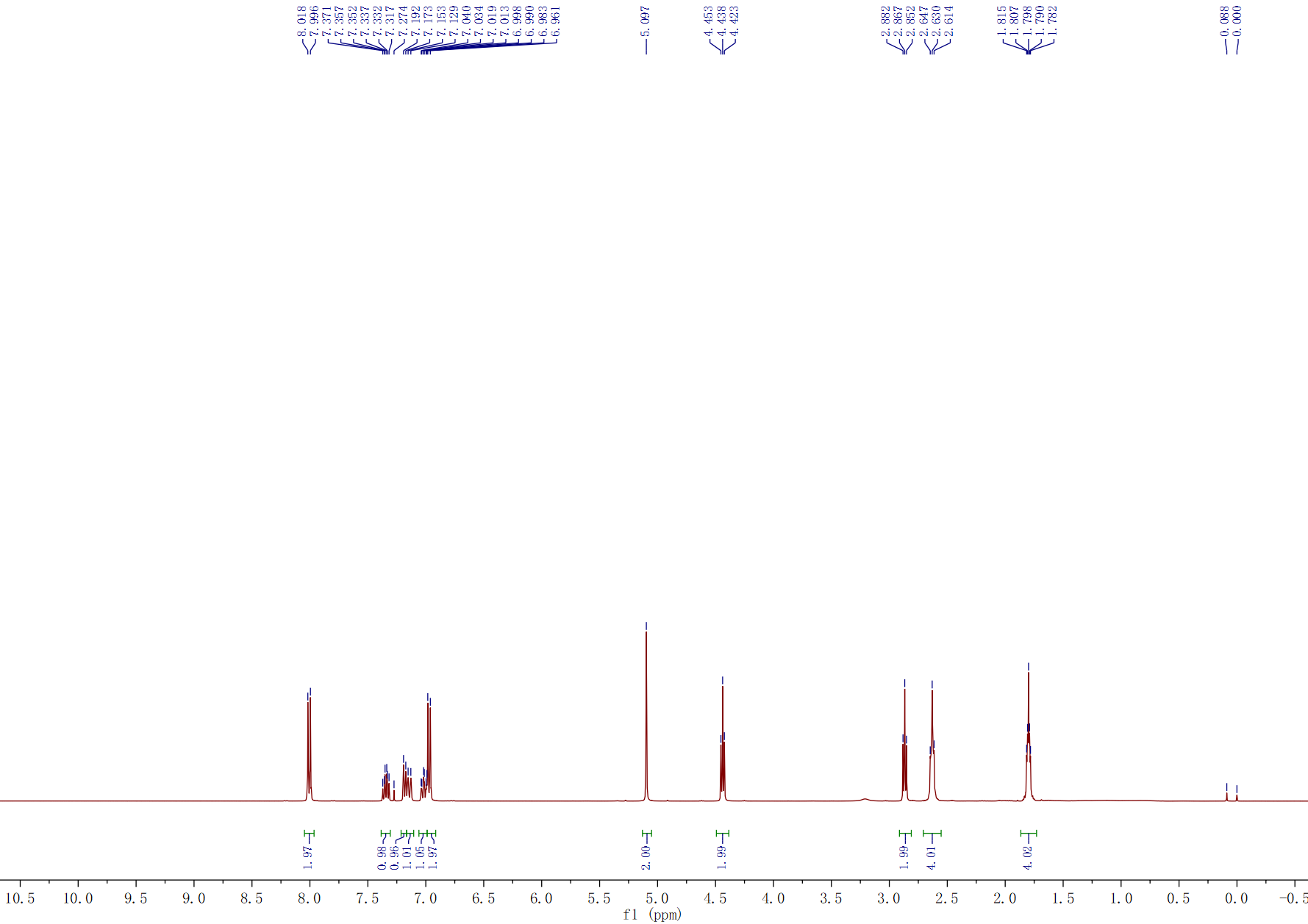


**^1^H NMR spectra of compound 20c (400 MHz, CDCl_3_)**


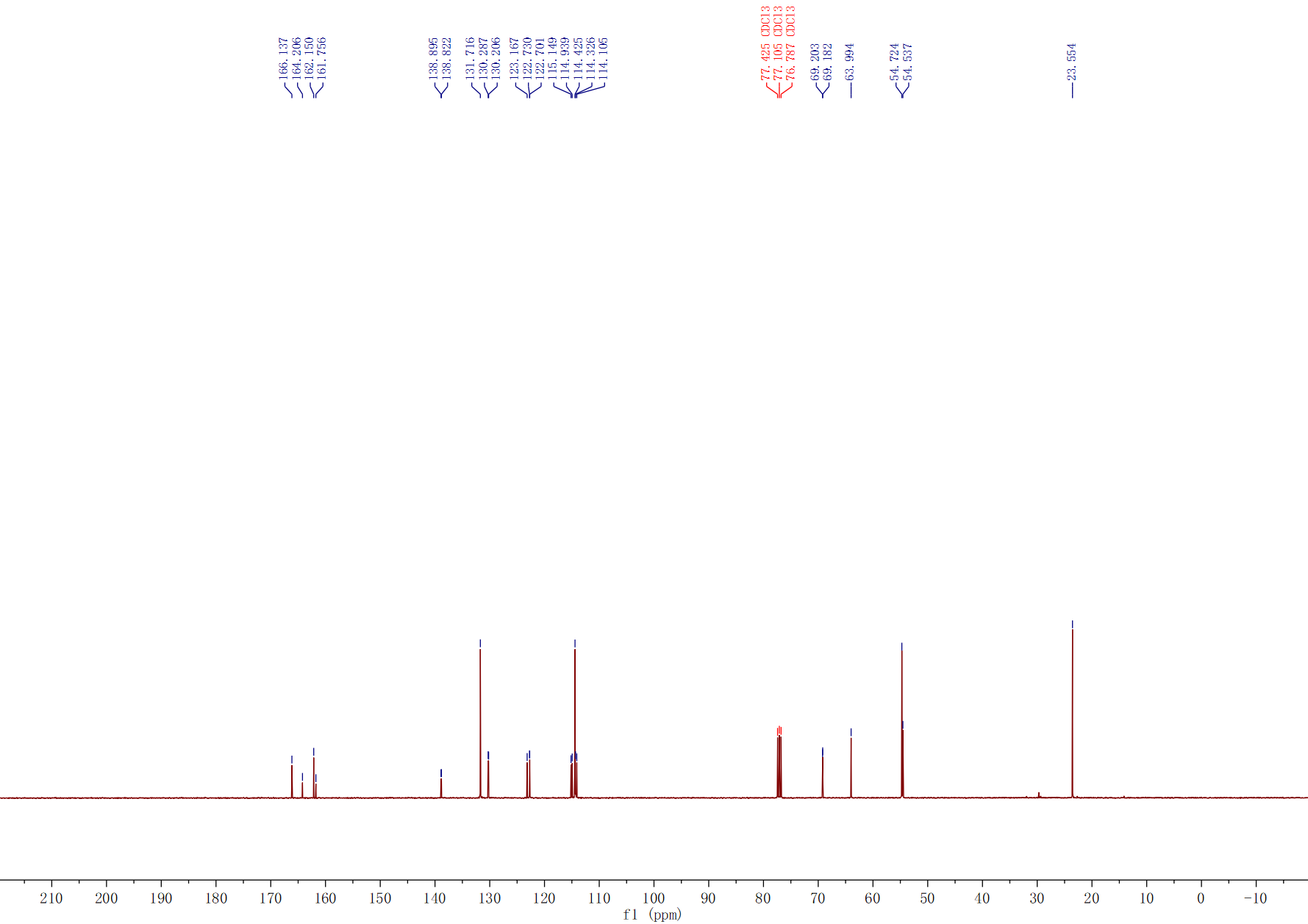


**^13^C NMR of compound 20c (100 MHz, CDCl_3_)**


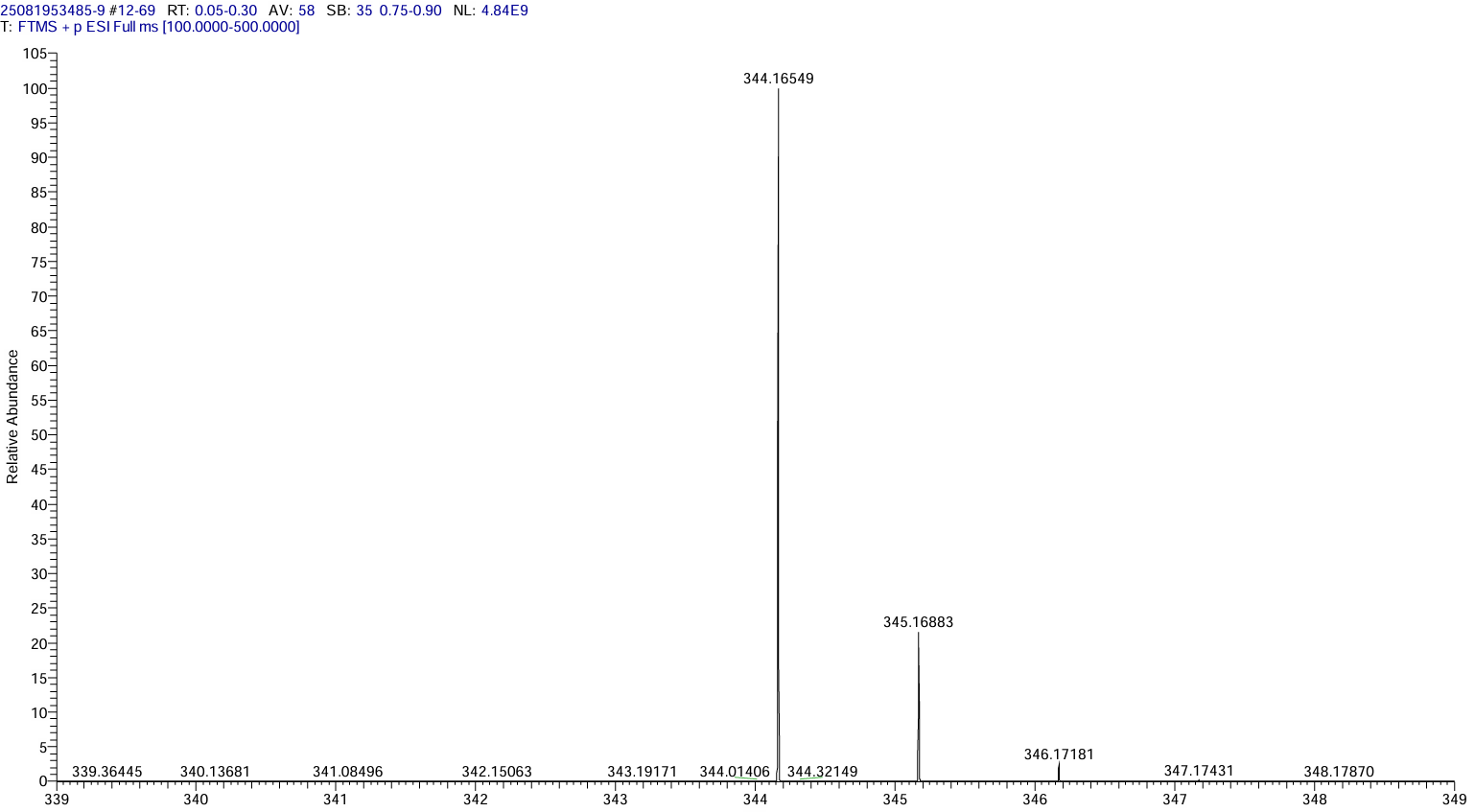


**HR-MS spectra of compound 20c**


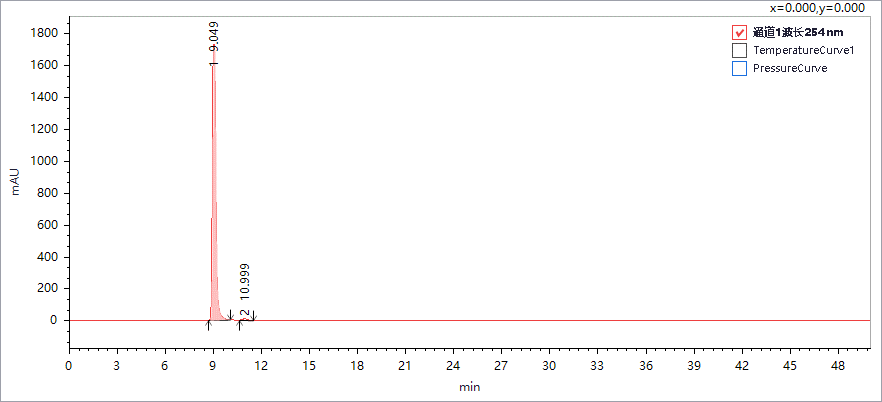


**HPLC purity of compound 20c**


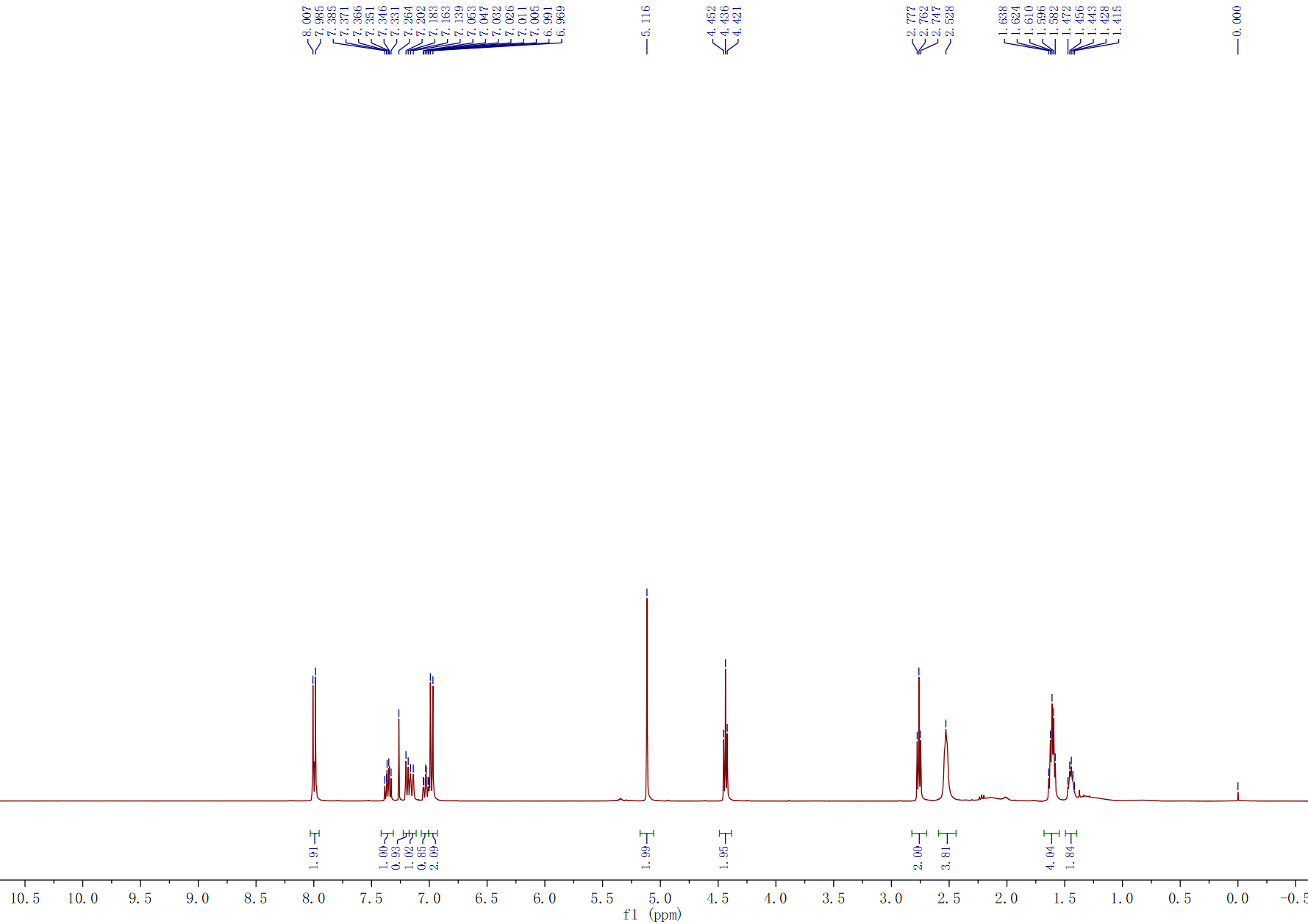


**^1^H NMR spectra of compound 20d (400 MHz, CDCl_3_)**


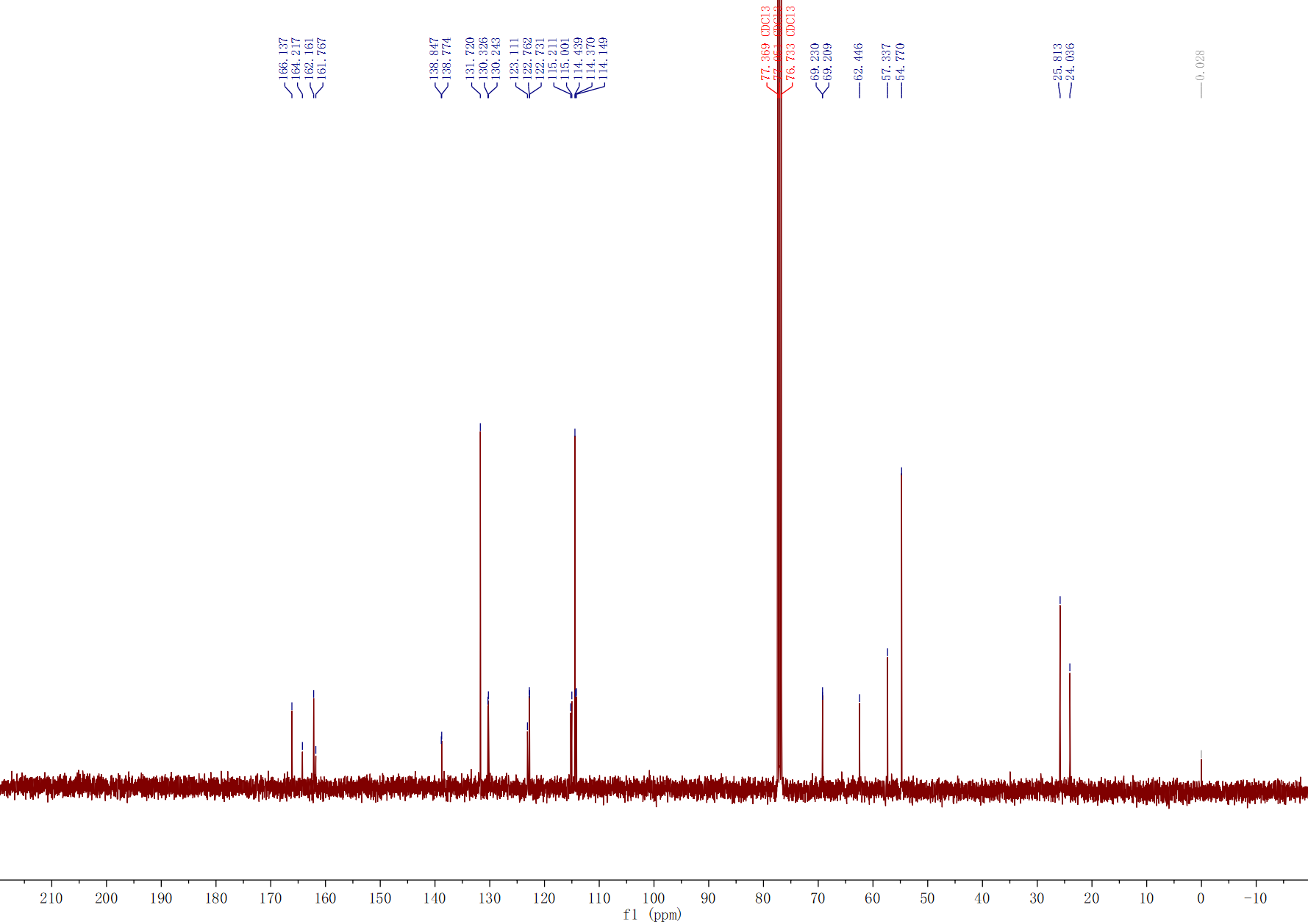


**^13^C NMR of compound 20c (100 MHz, CDCl_3_)**


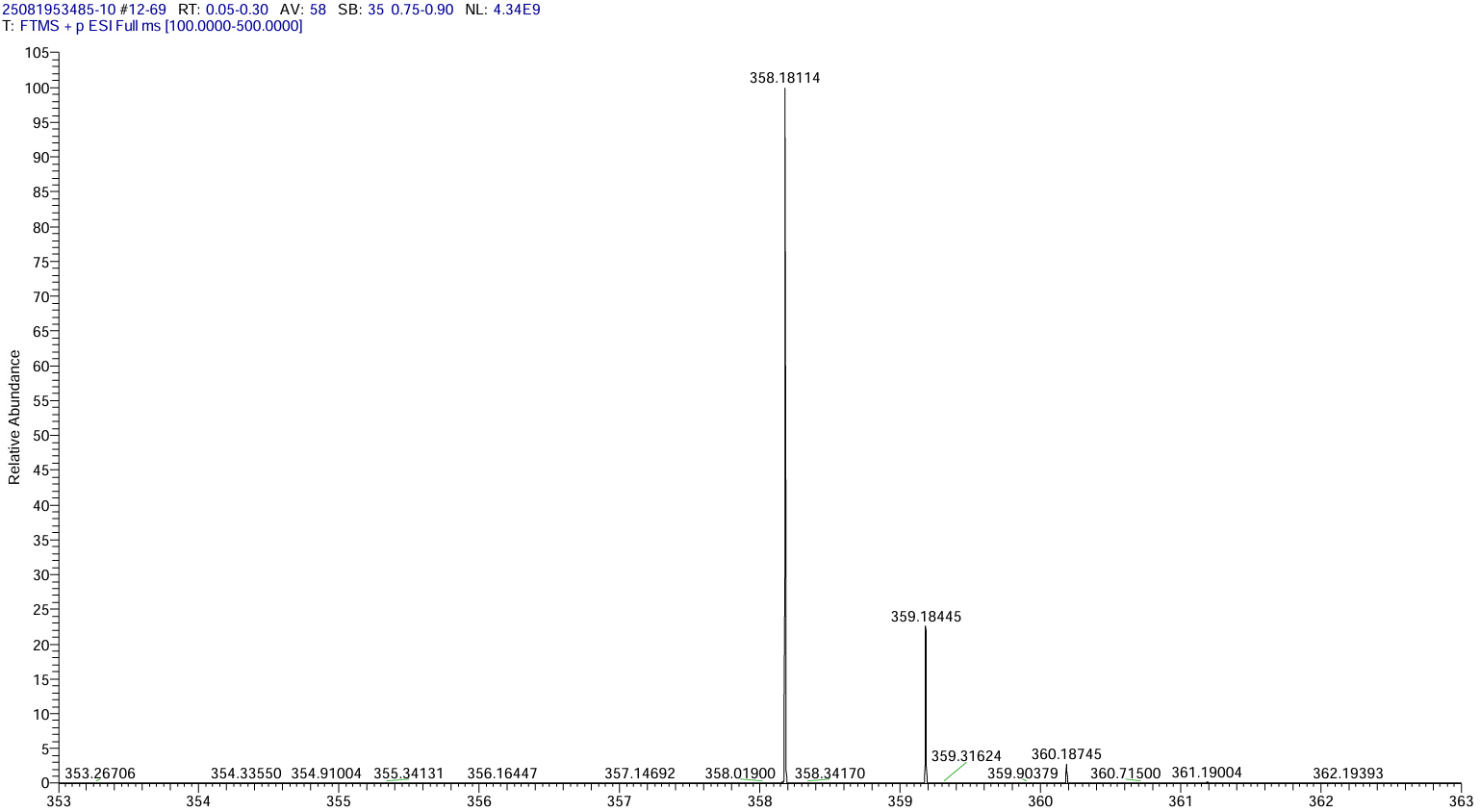


**HR-MS spectra of compound 20d**


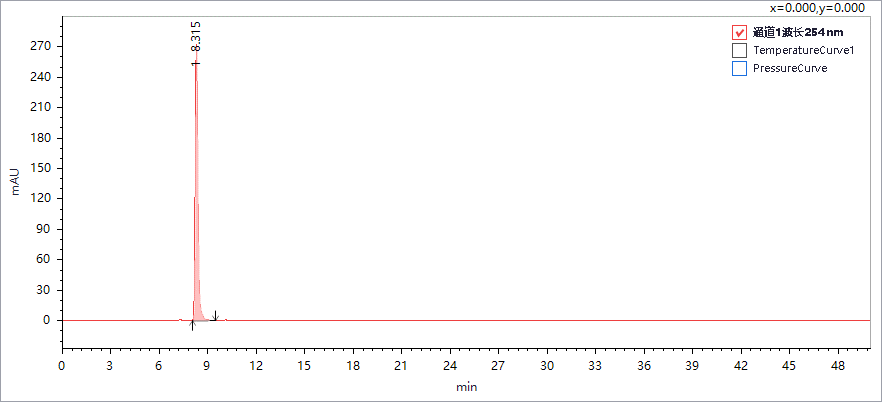


**HPLC purity of compound 20d**


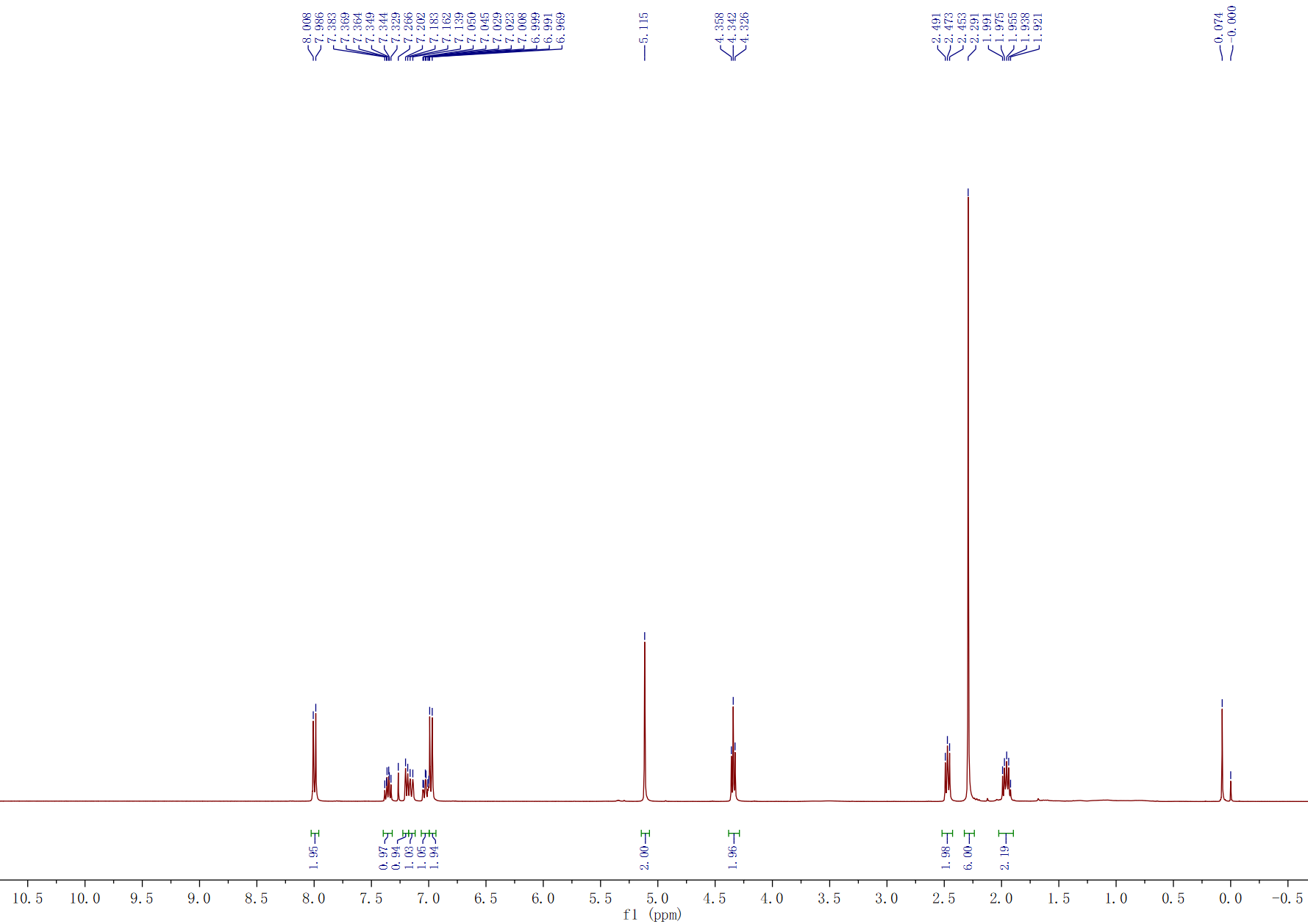


**^1^H NMR spectra of compound 21a (400 MHz, CDCl_3_)**


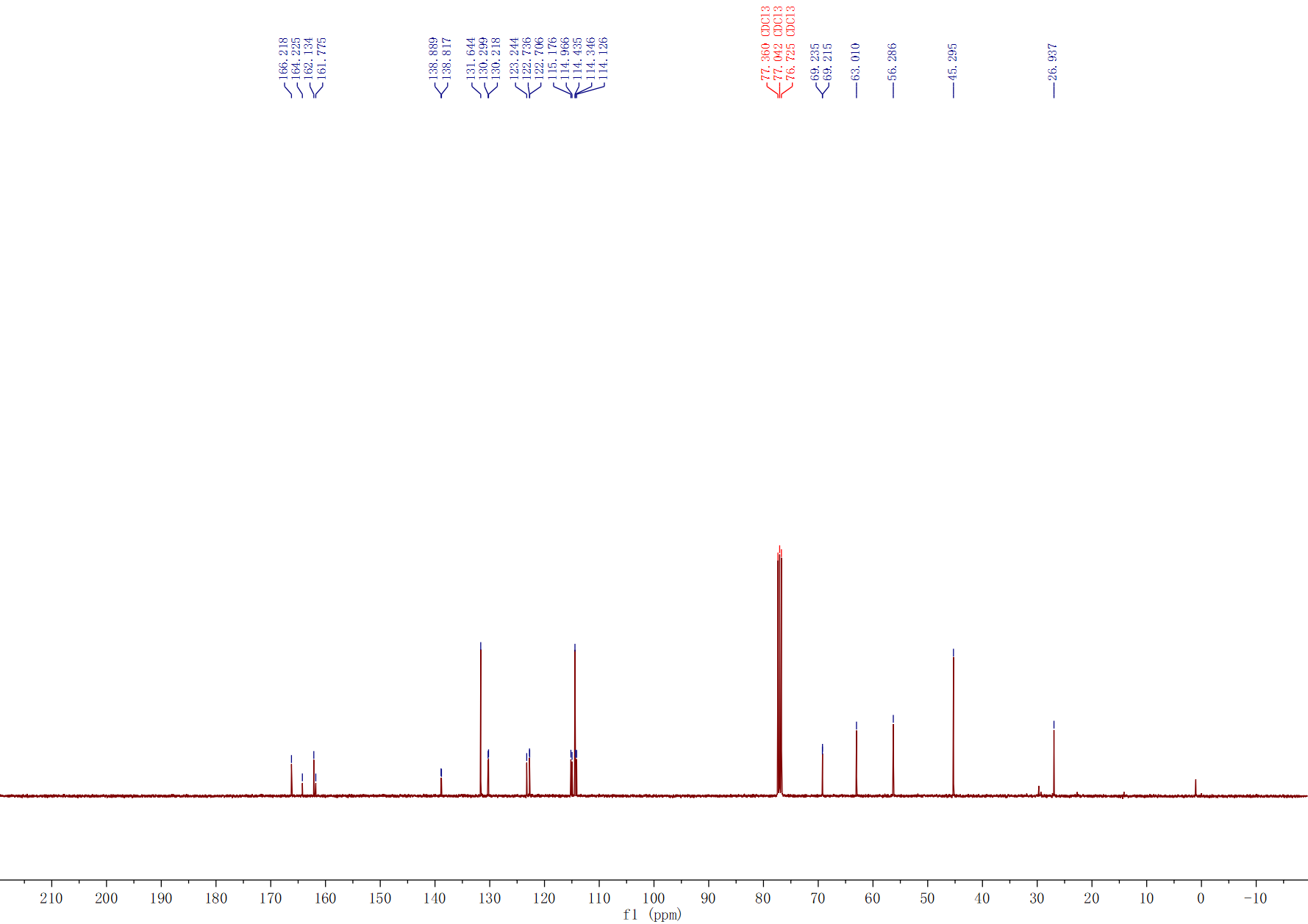


**^13^C NMR of compound 21a (100 MHz, CDCl_3_)**


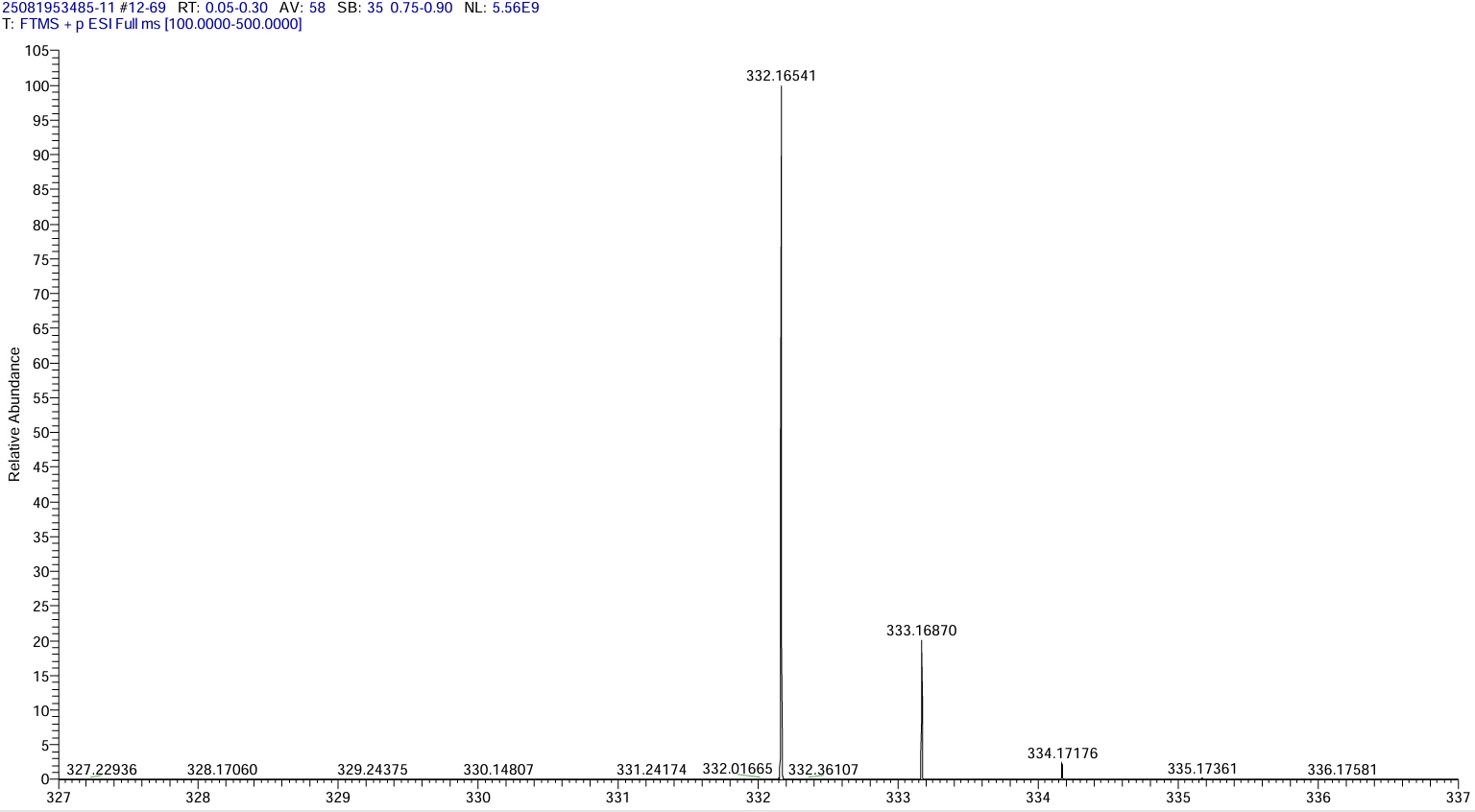


**HR-MS spectra of compound 21a**


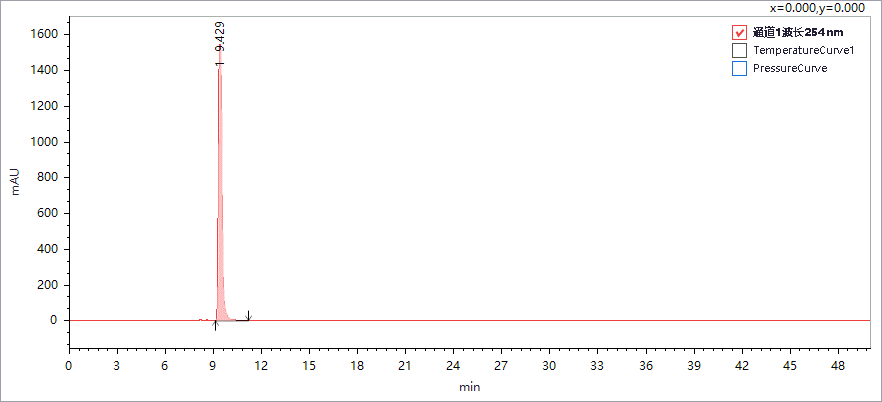


**HPLC purity of compound 21a**


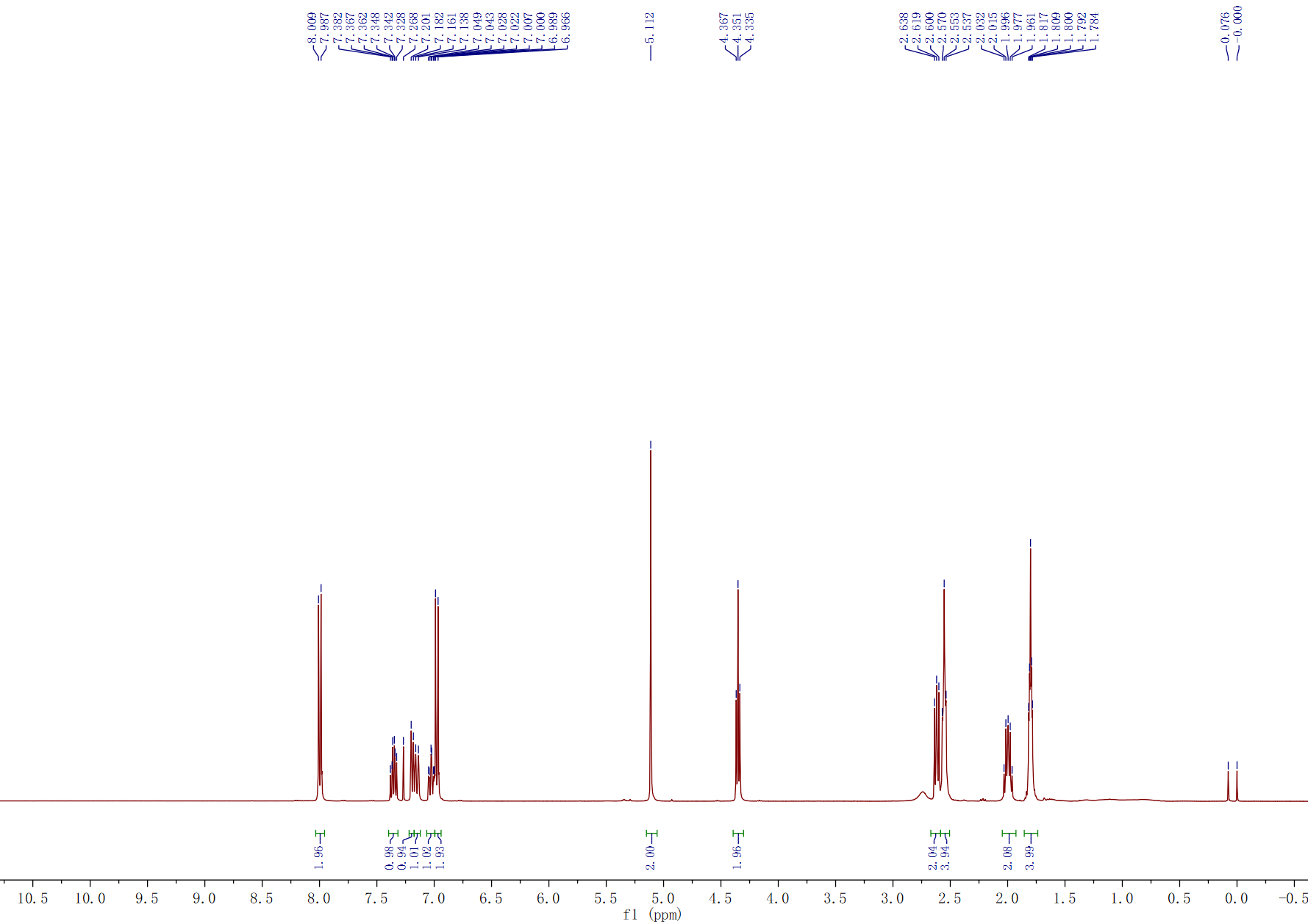


**^1^H NMR spectra of compound 21c (400 MHz, CDCl_3_)**


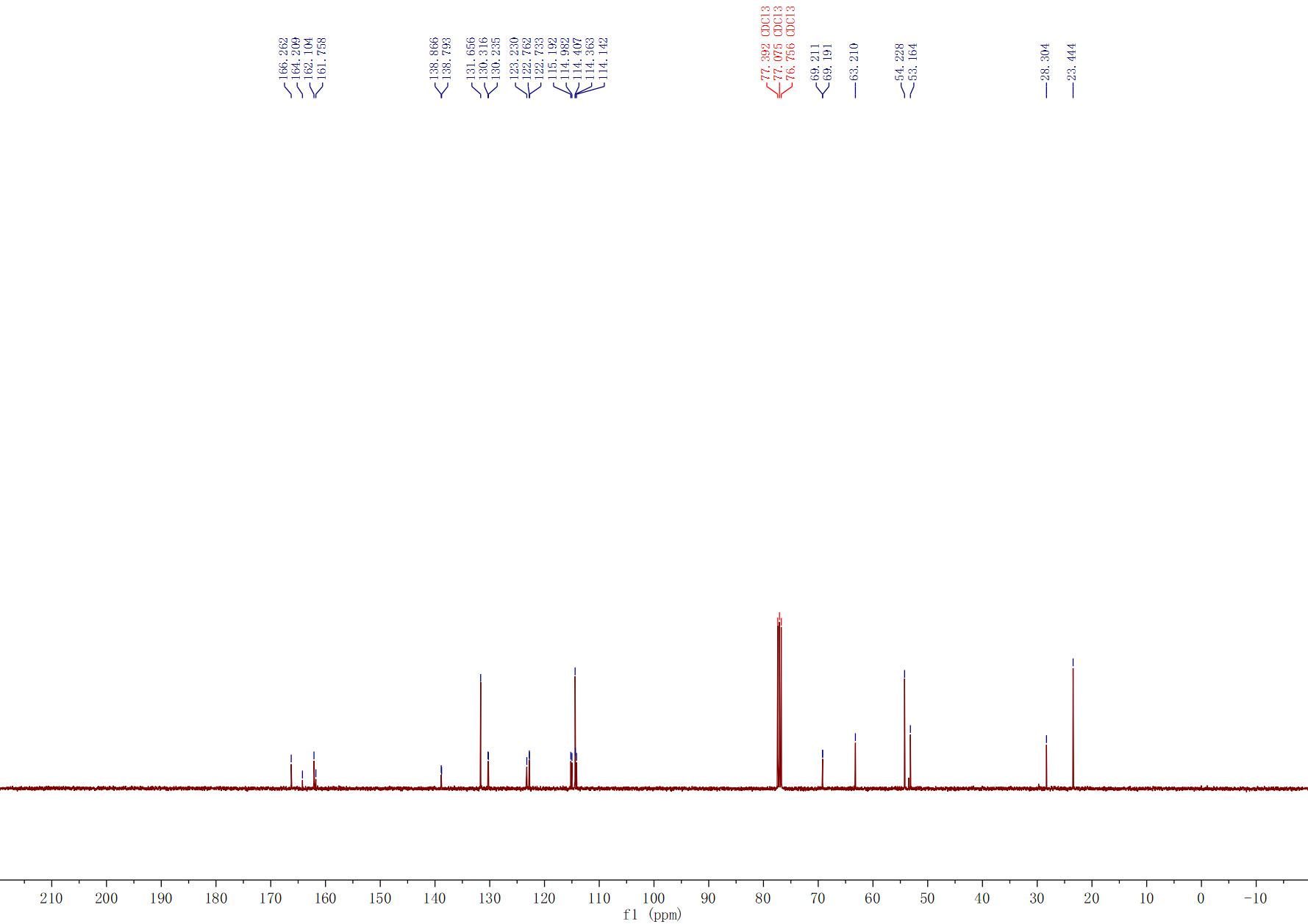


**^13^C NMR of compound 21c (100 MHz, CDCl_3_)**


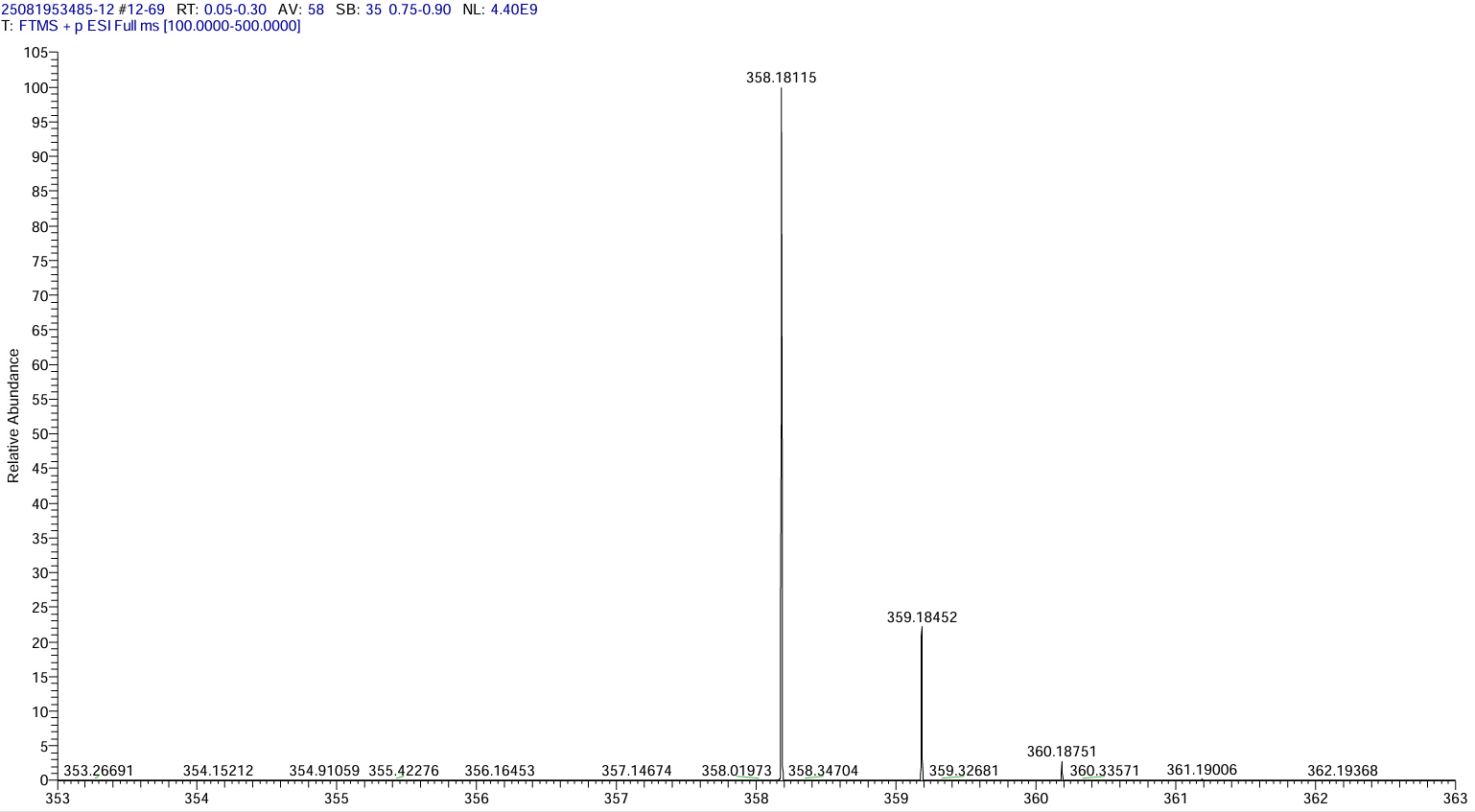


**HR-MS spectra of compound 21c**


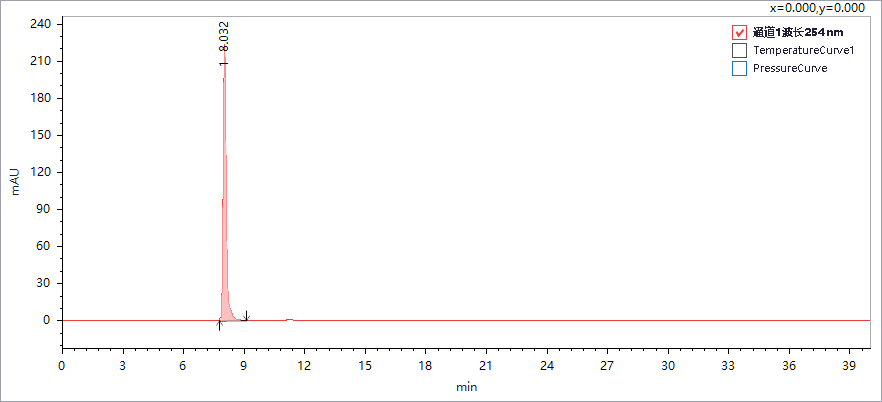


**HPLC purity of compound 21c**


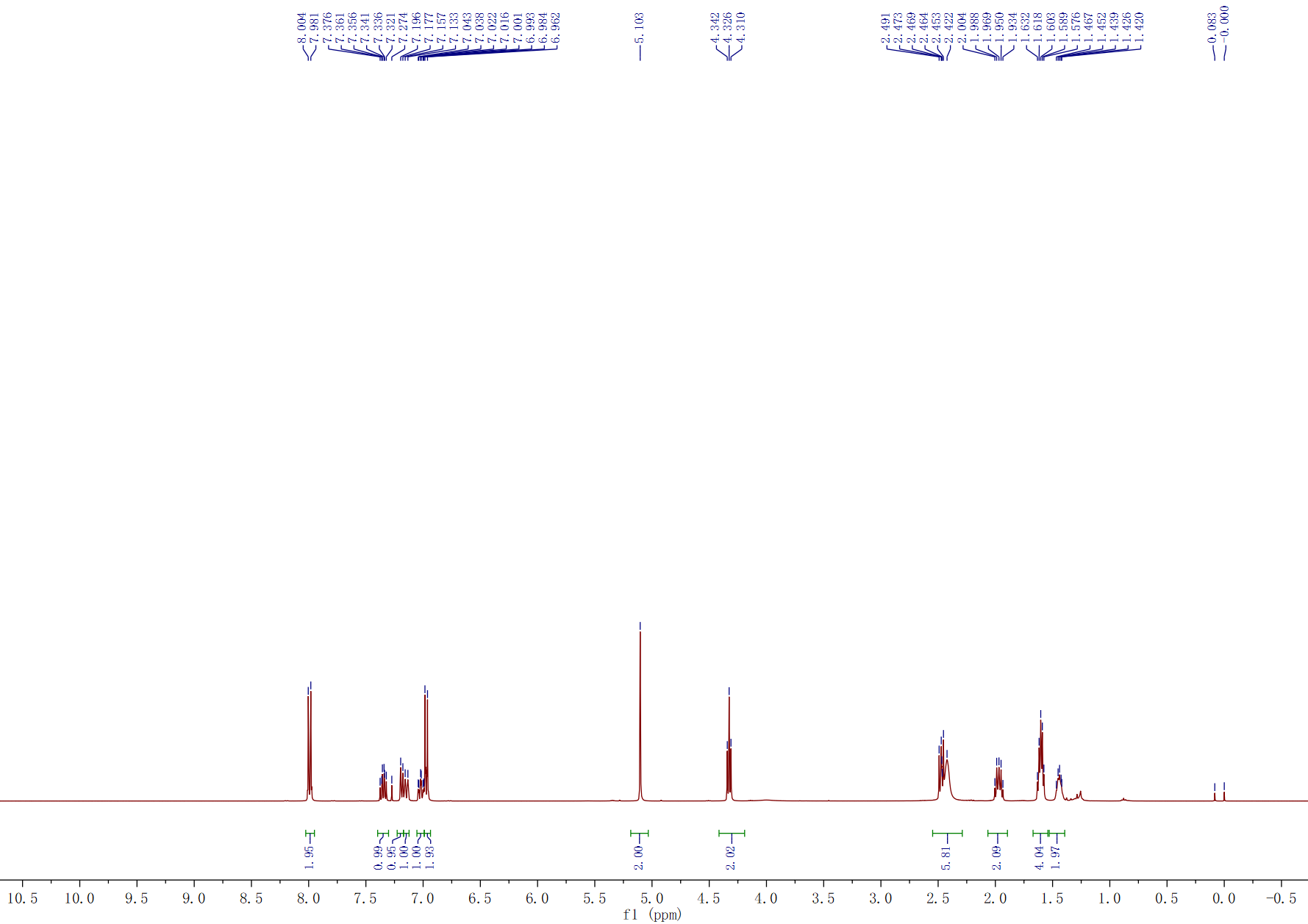


**^1^H NMR spectra of compound 21d (400 MHz, CDCl_3_)**


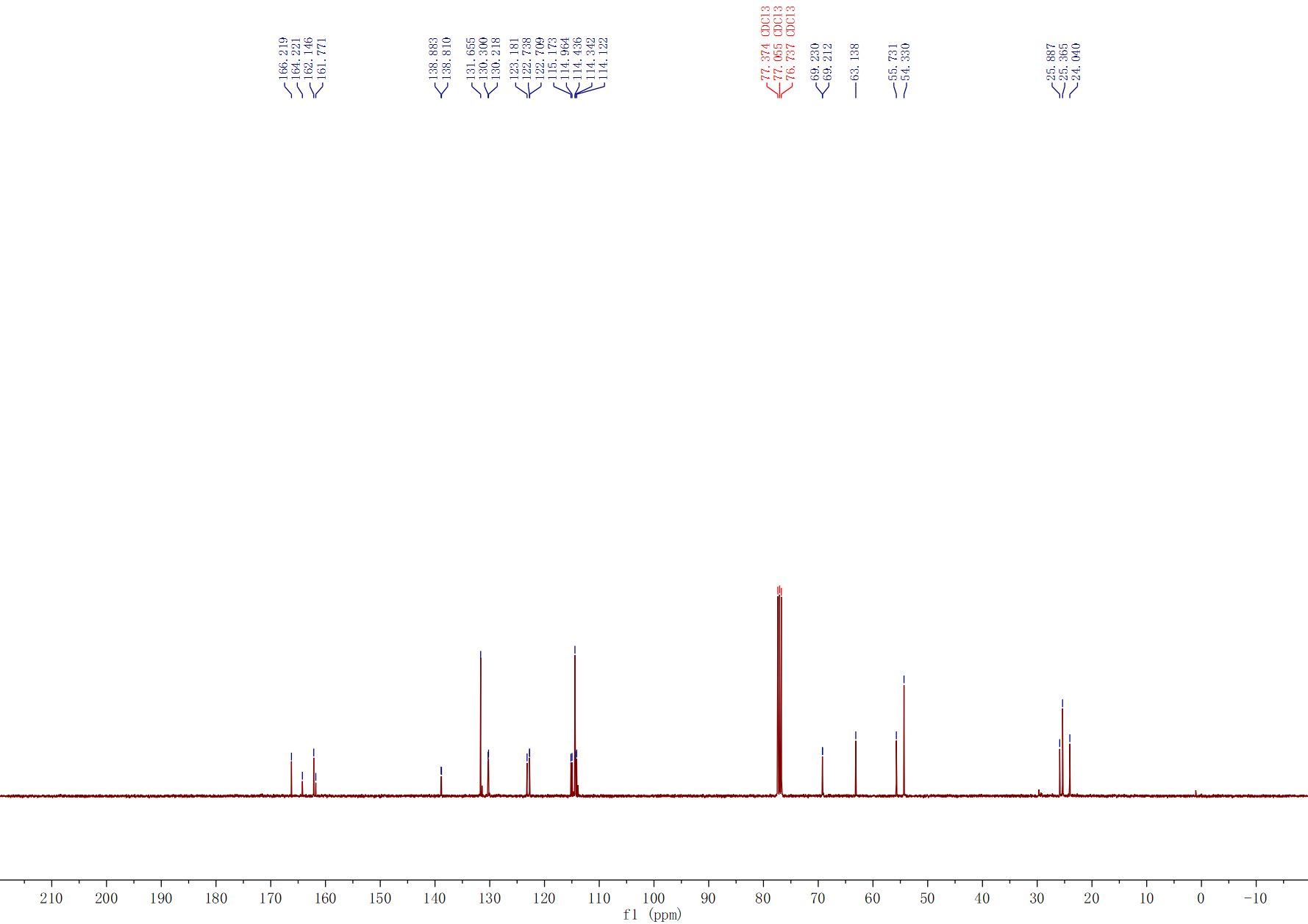


**^13^C NMR of compound 21d (100 MHz, CDCl_3_)**


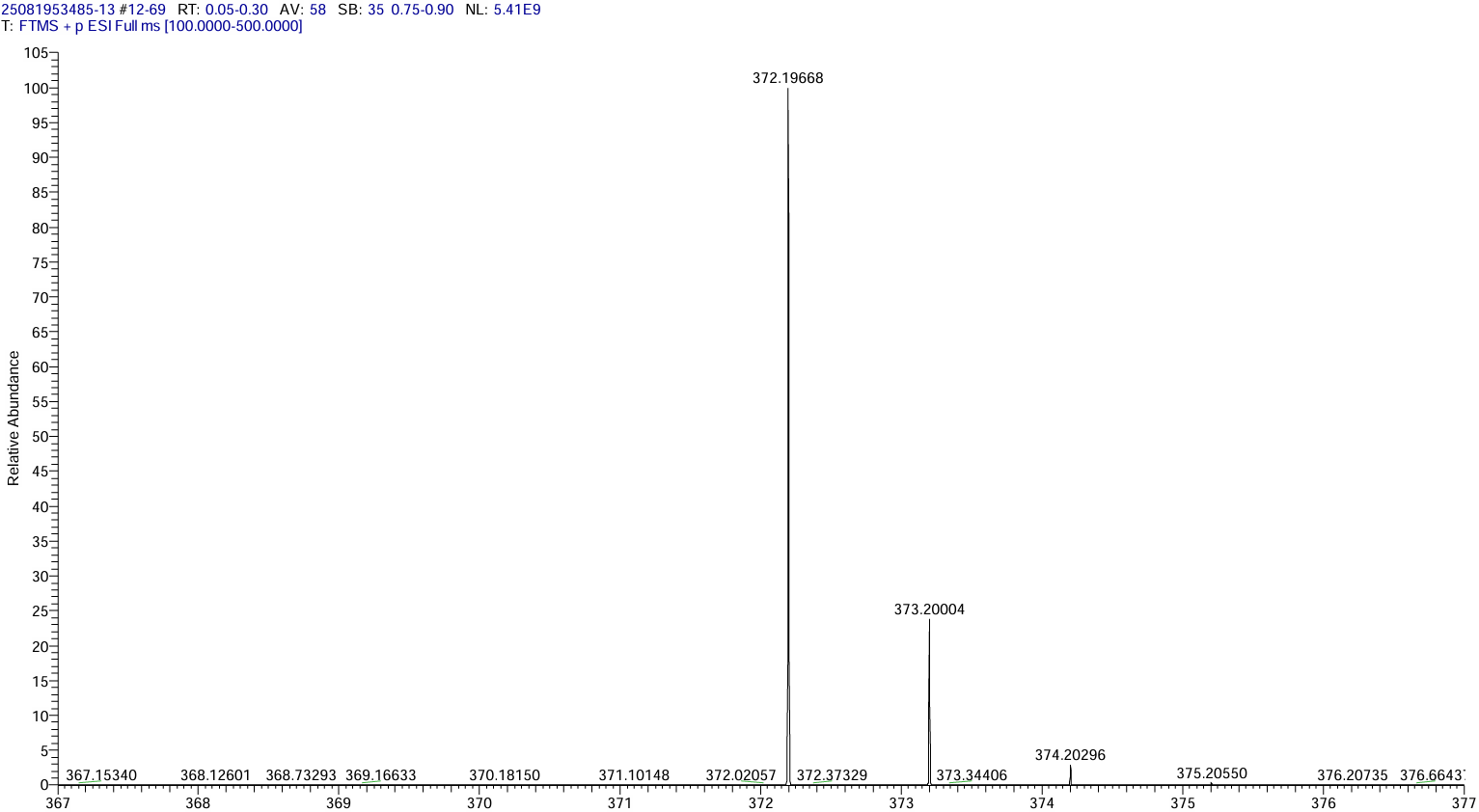


**HR-MS spectra of compound 21d**


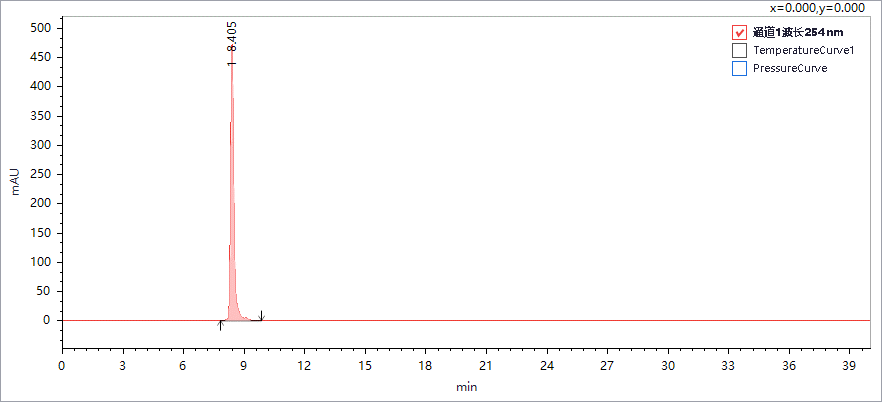


**HPLC purity of compound 21d**


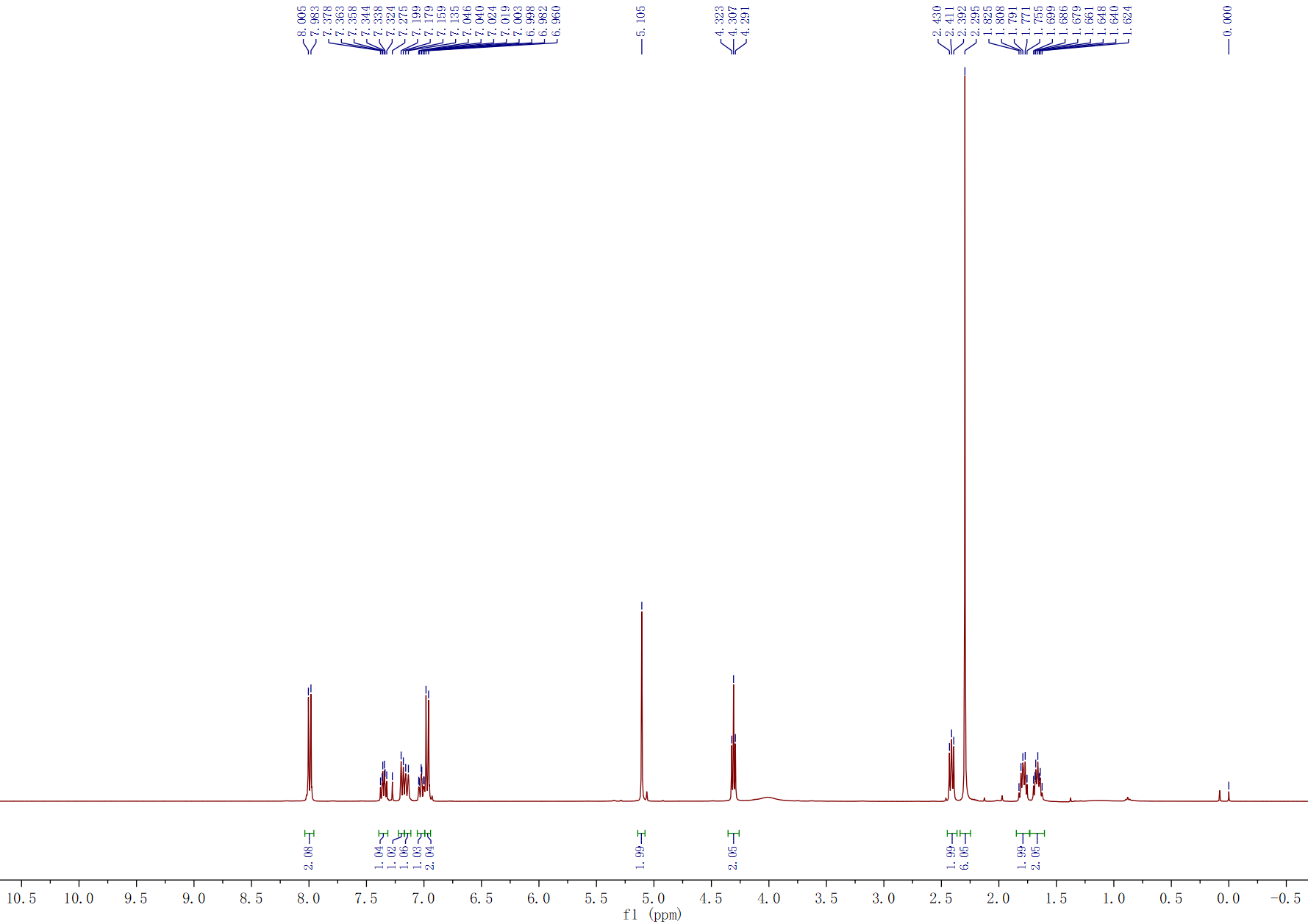


**^1^H NMR spectra of compound 22a (400 MHz, CDCl_3_)**


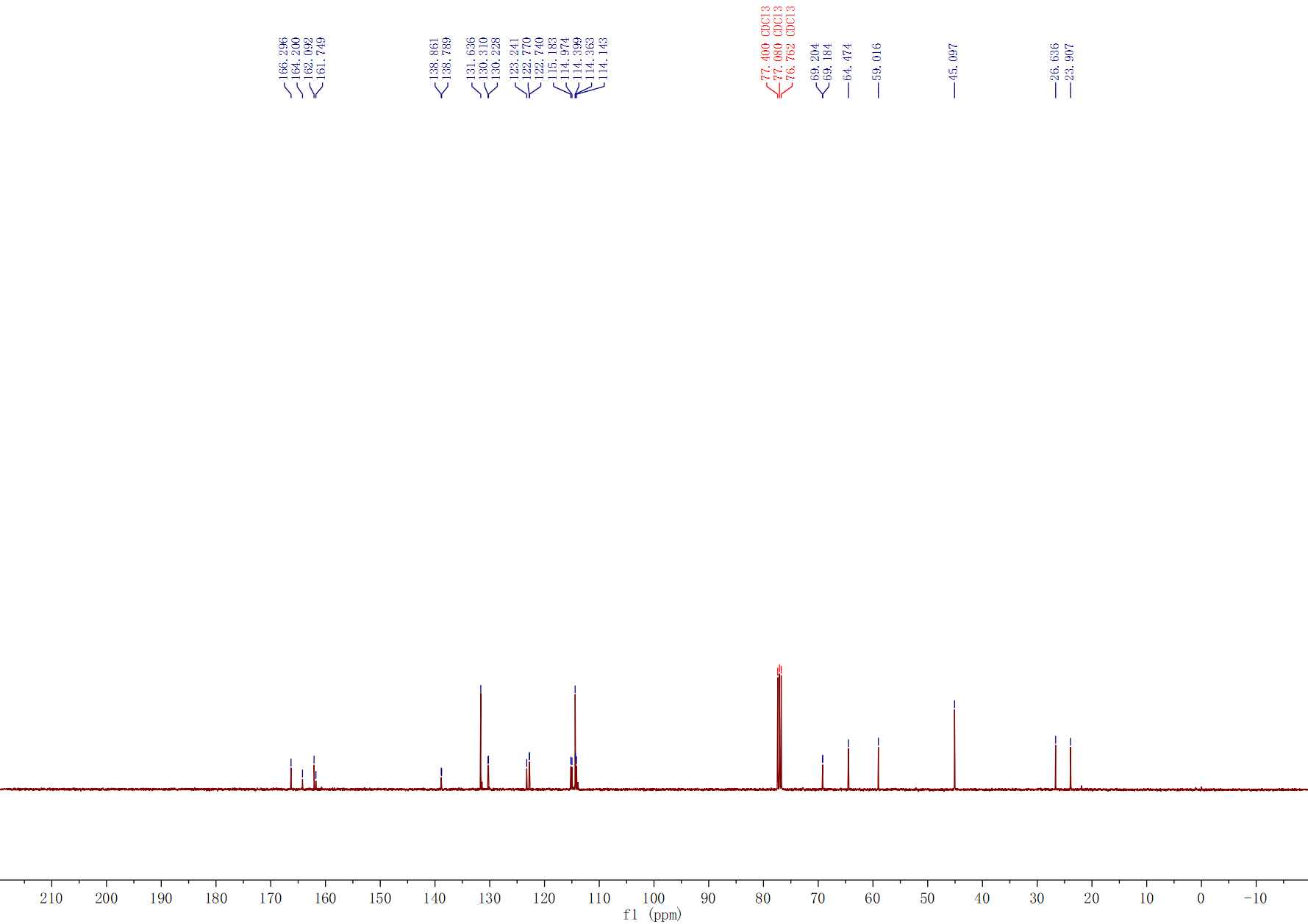


**^13^C NMR of compound 22a (100 MHz, CDCl_3_)**


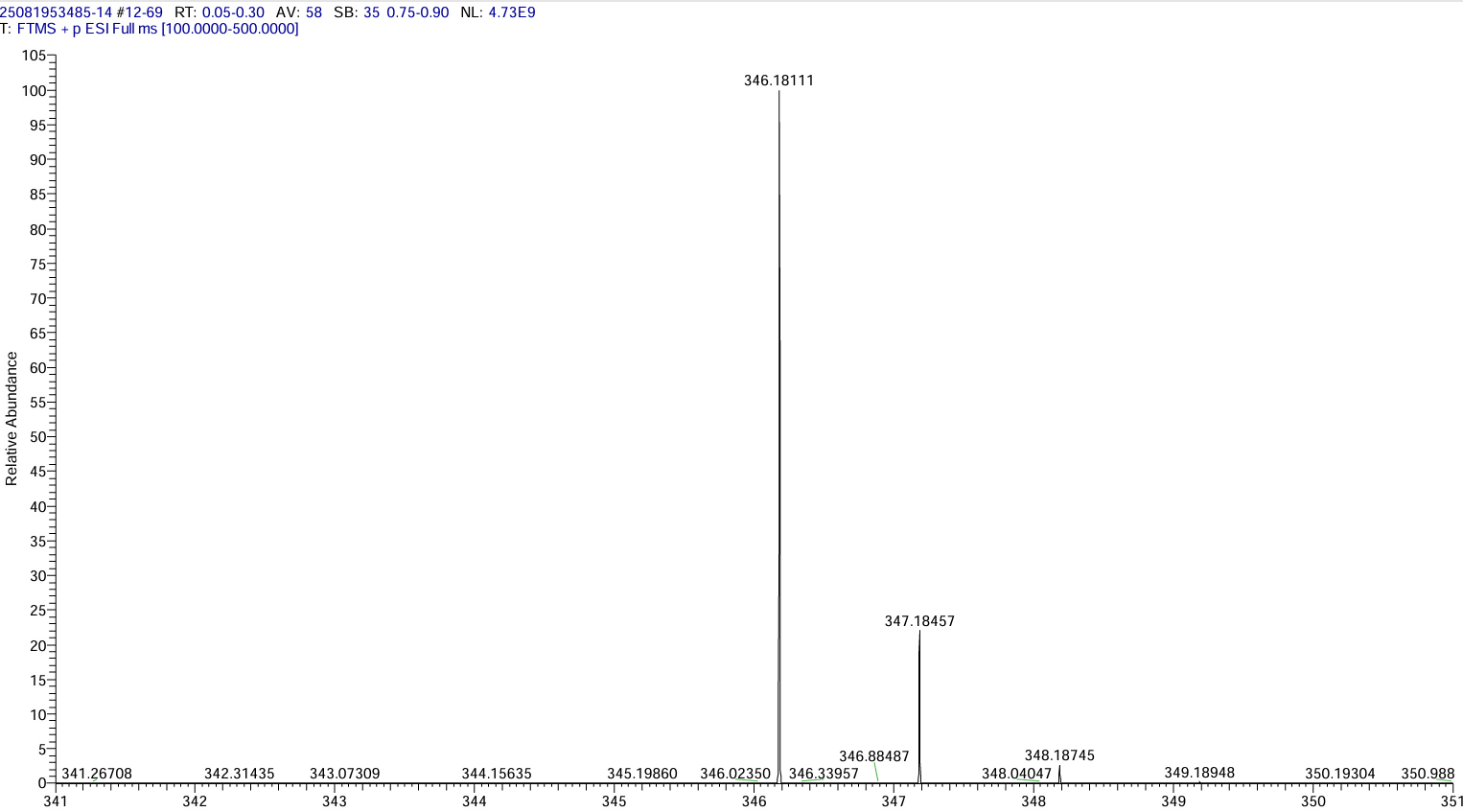


**HR-MS spectra of compound 22a**


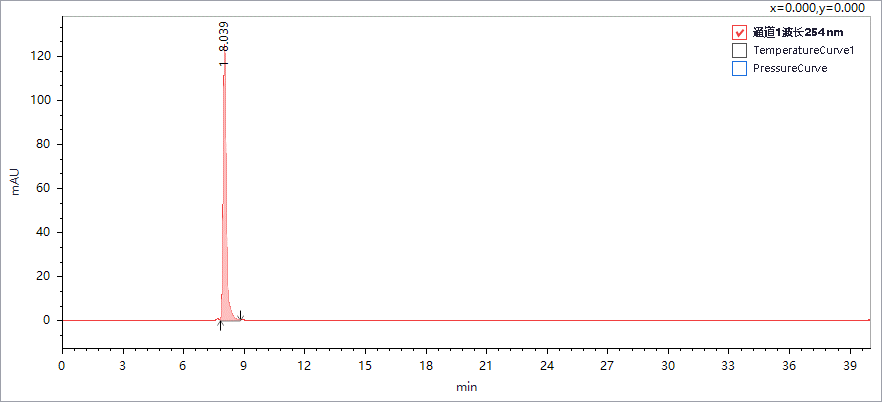


**HPLC purity of compound 22a**


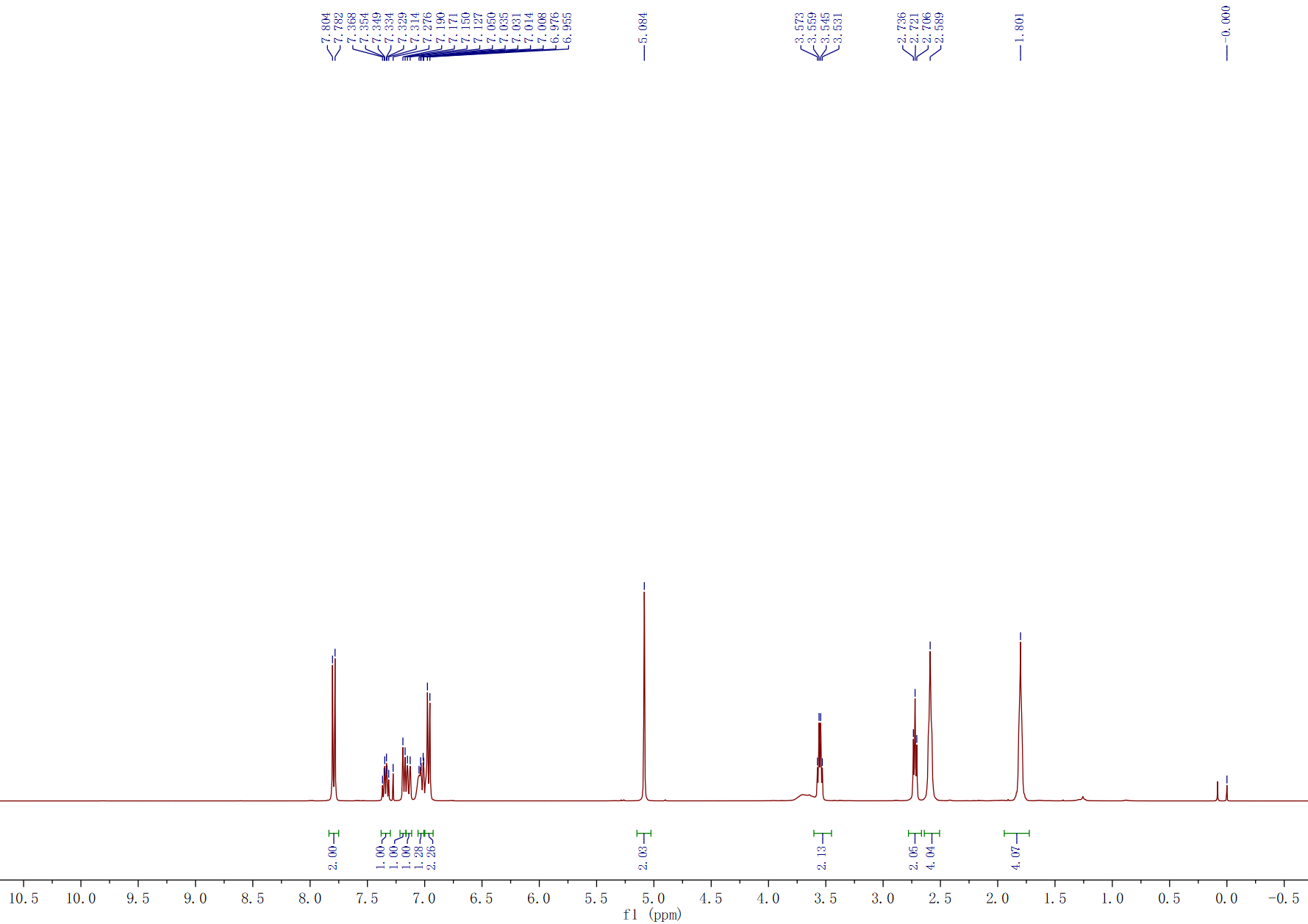


**^1^H NMR spectra of compound 23c (400 MHz, CDCl_3_)**


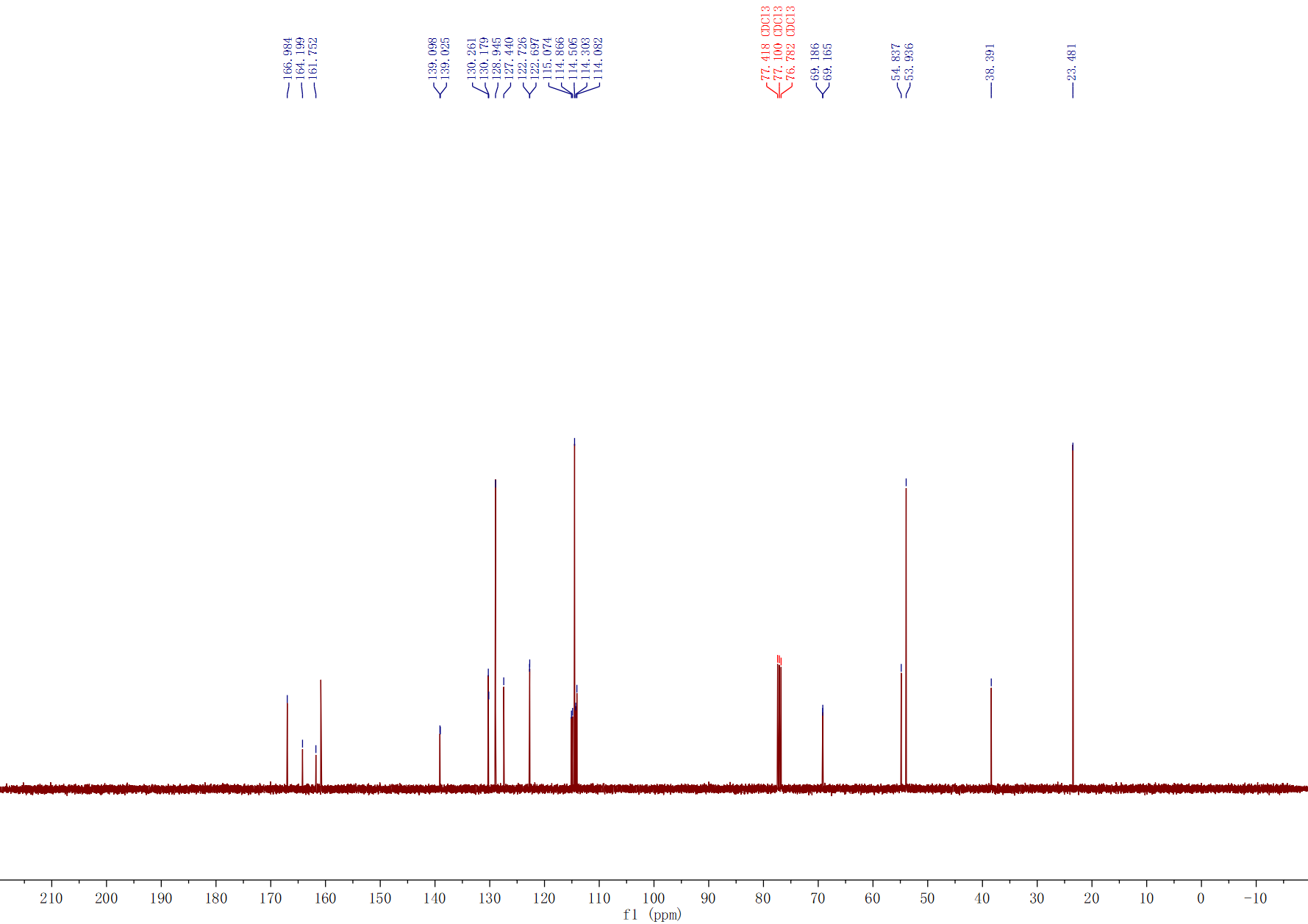


**^13^C NMR of compound 23c (100 MHz, CDCl_3_)**


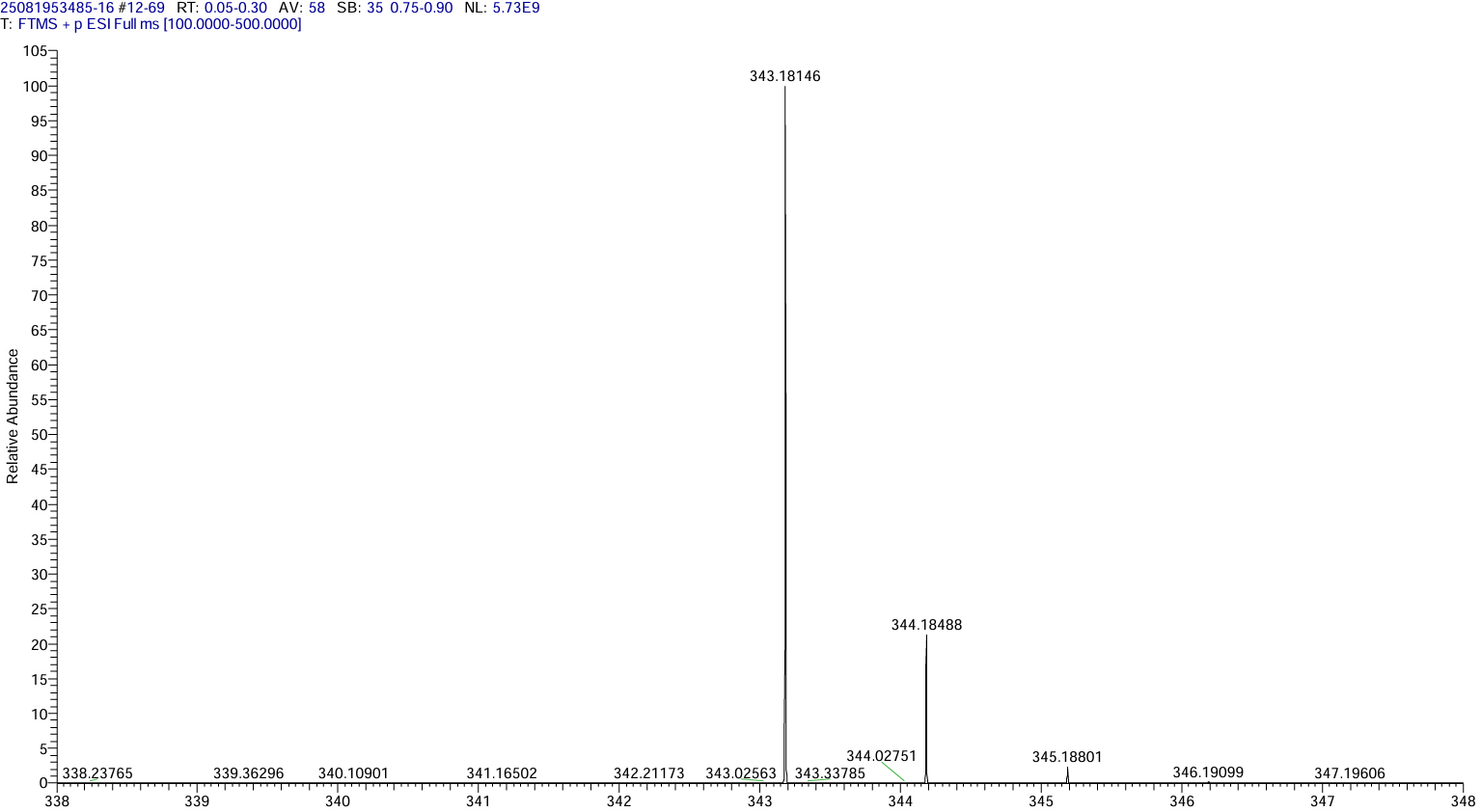


**HR-MS spectra of compound 23c**


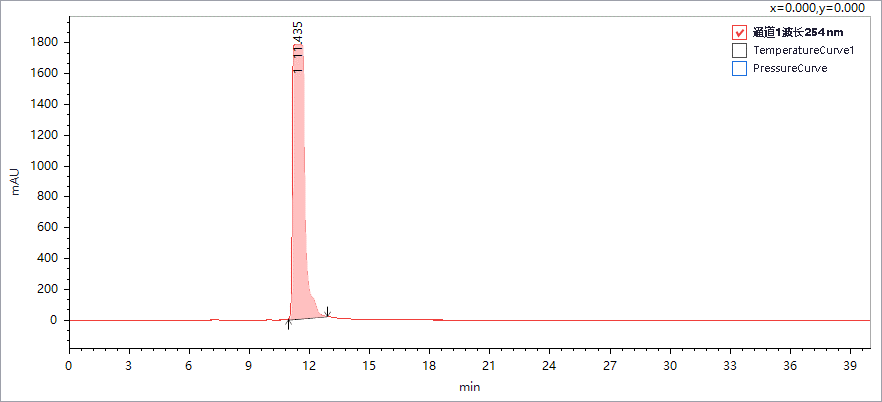


**HPLC purity of compound 23c**


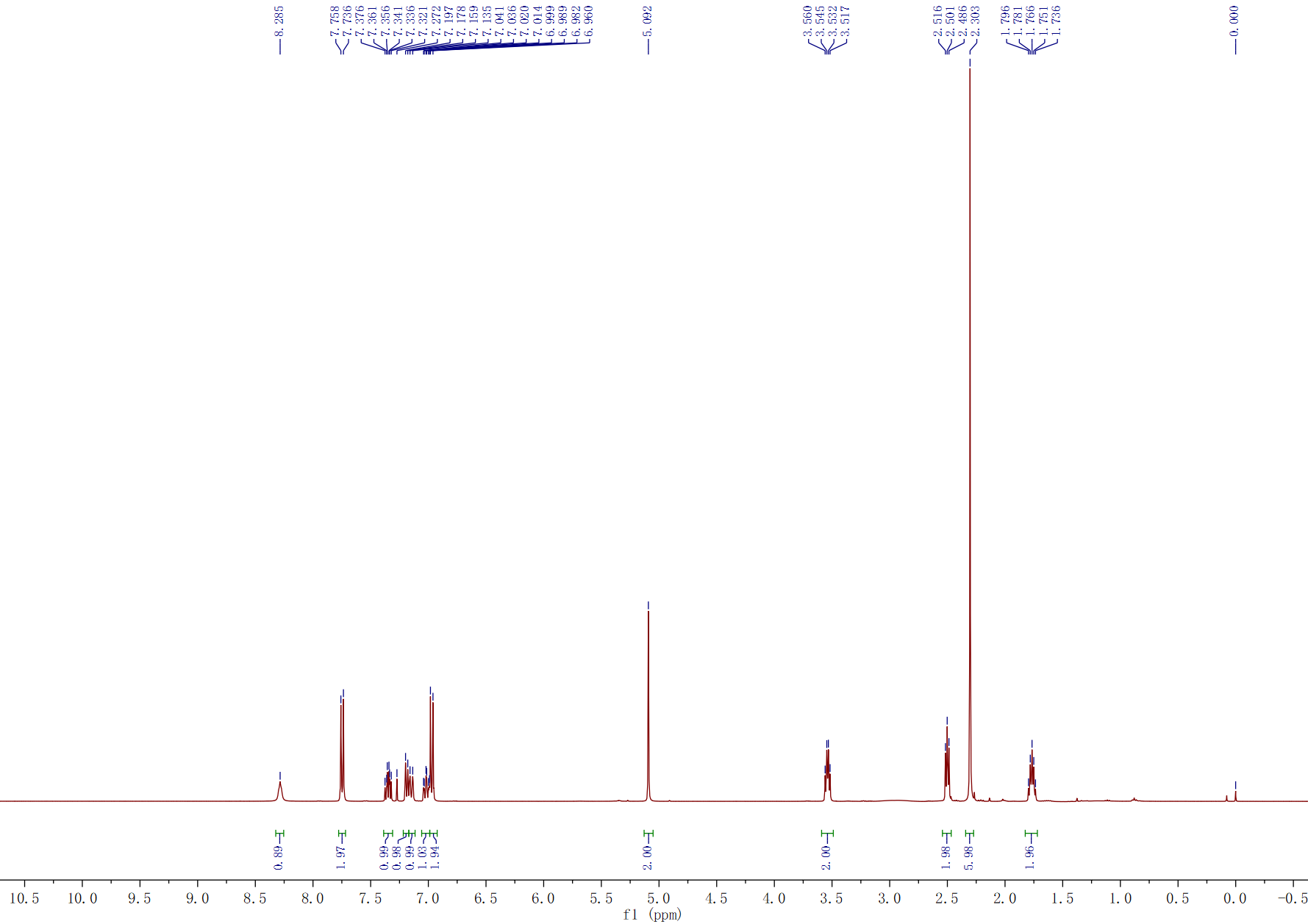


**^1^H NMR spectra of compound 24a (400 MHz, CDCl_3_)**


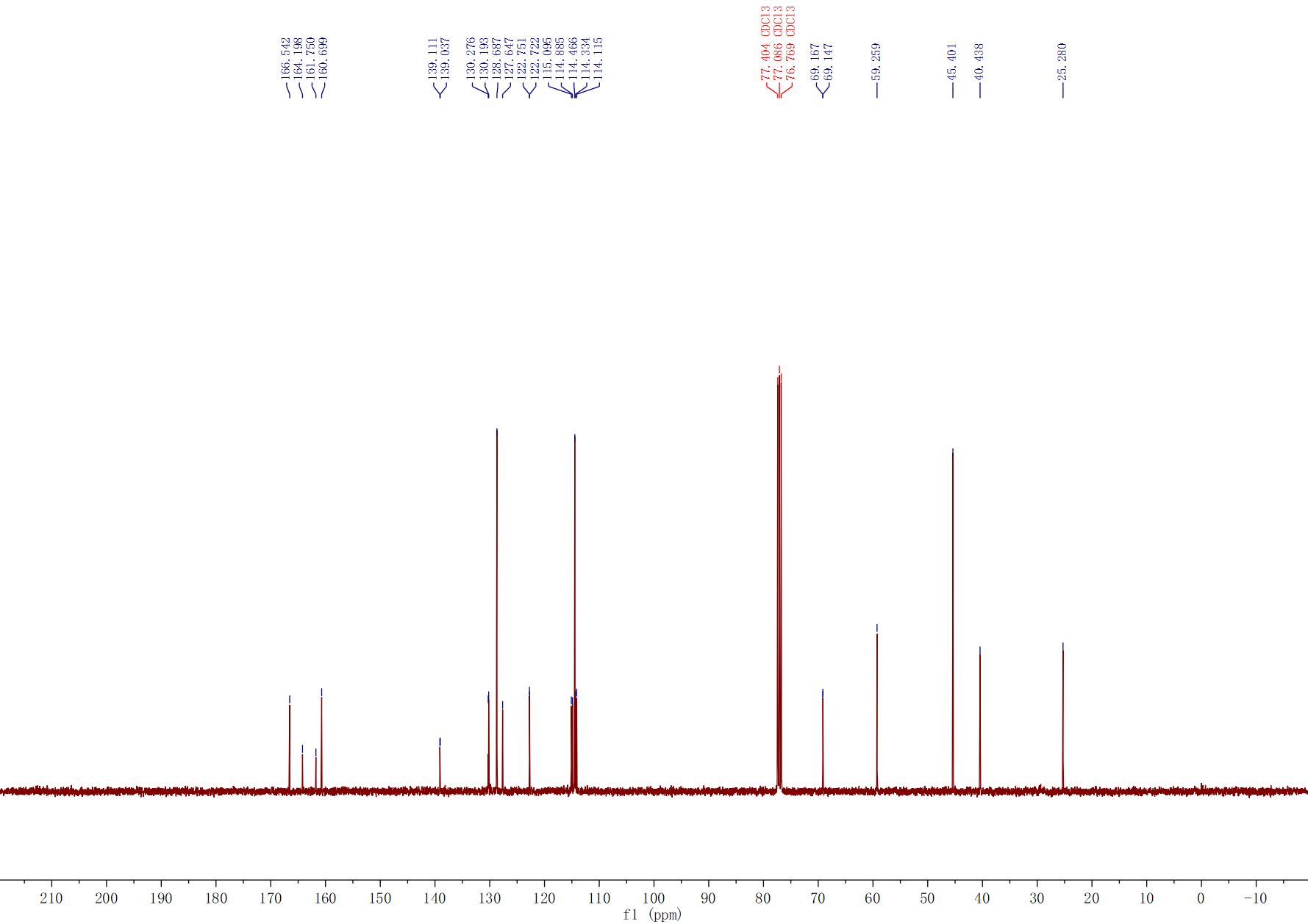


**^13^C NMR of compound 24a (100 MHz, CDCl_3_)**


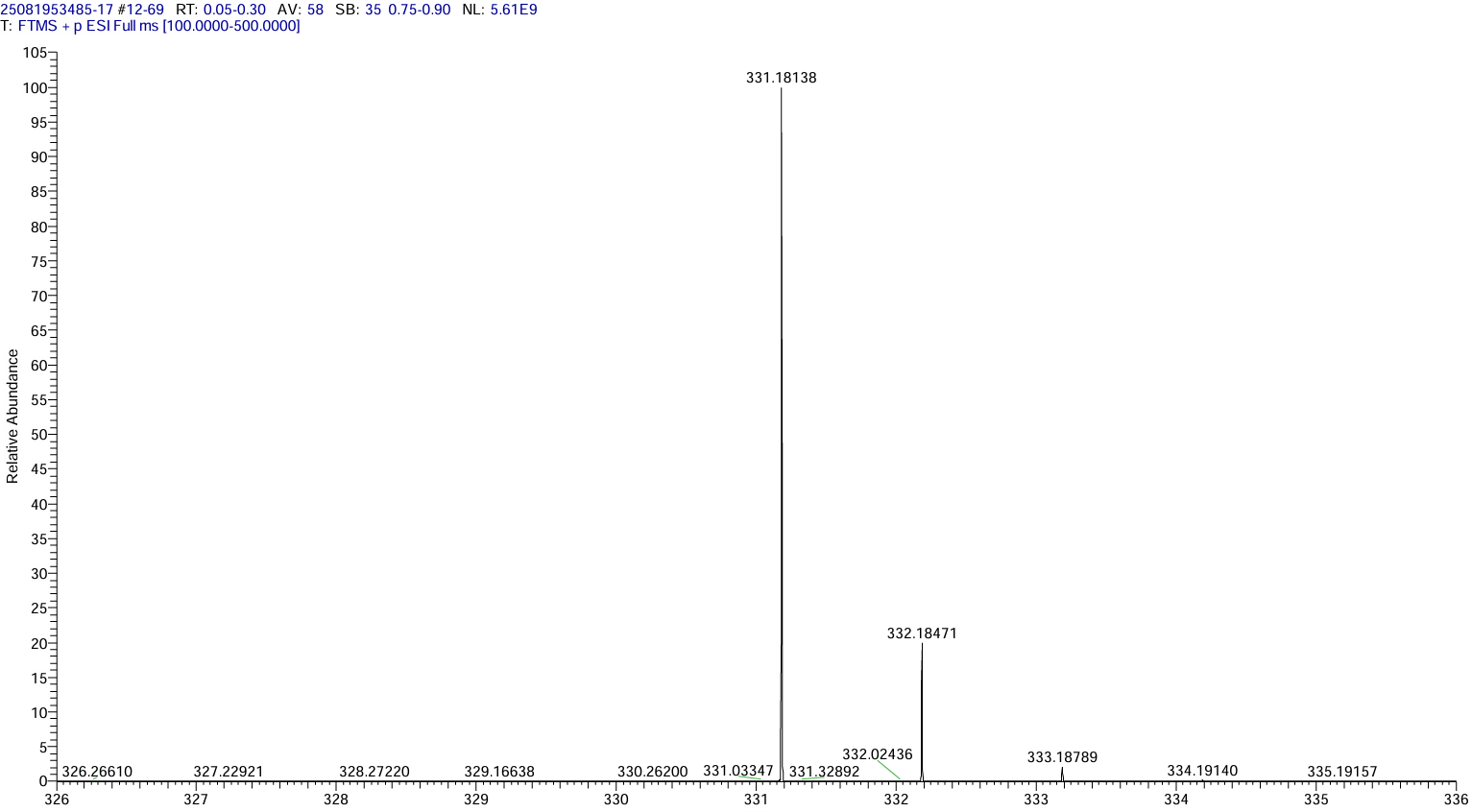


**HR-MS spectra of compound 24a**


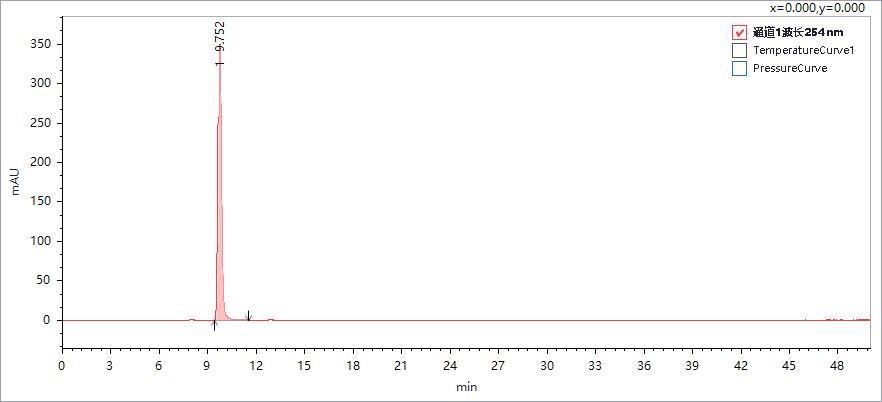


**HPLC purity of compound 24a**


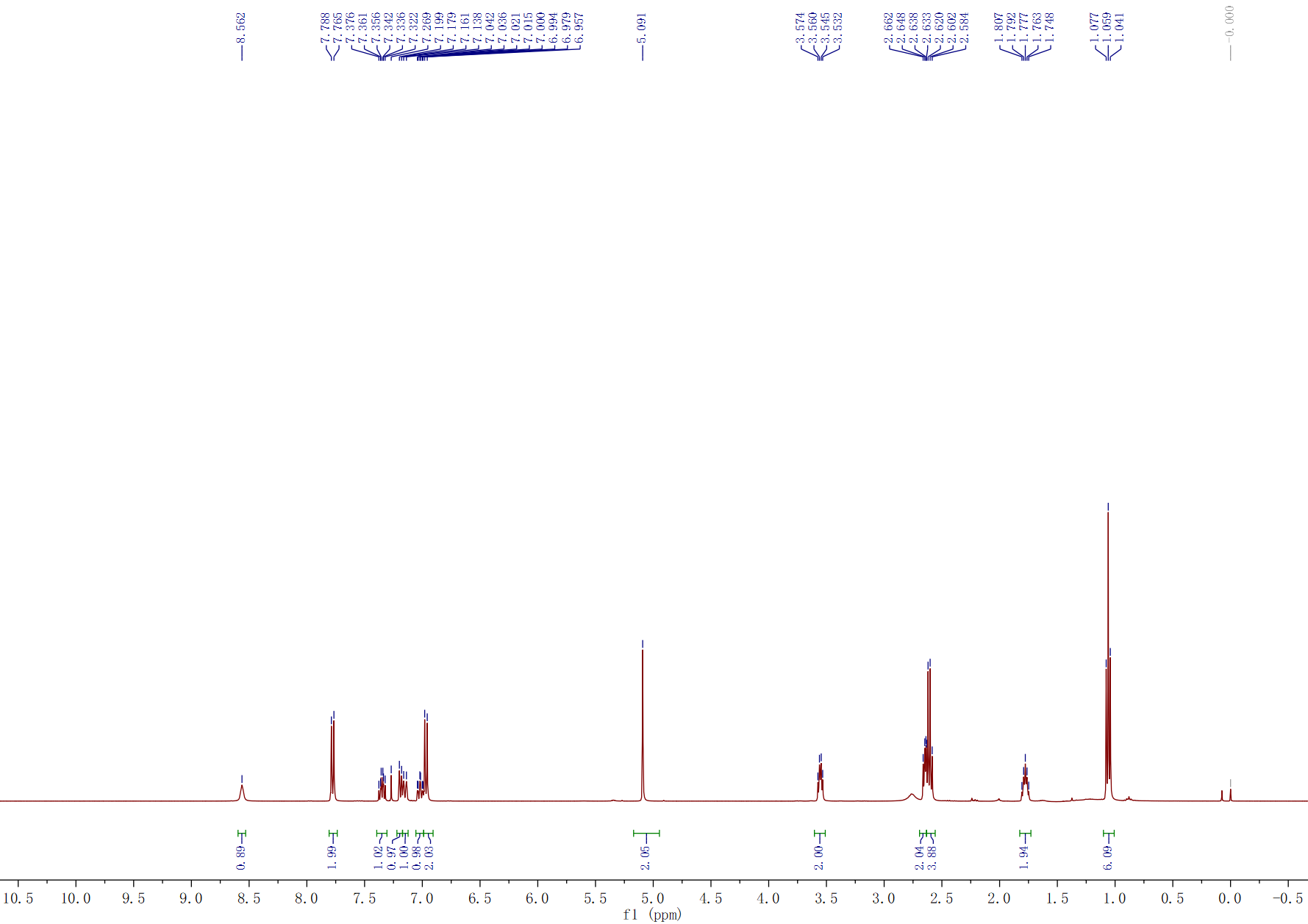


**^1^H NMR spectra of compound 24b (400 MHz, CDCl_3_)**


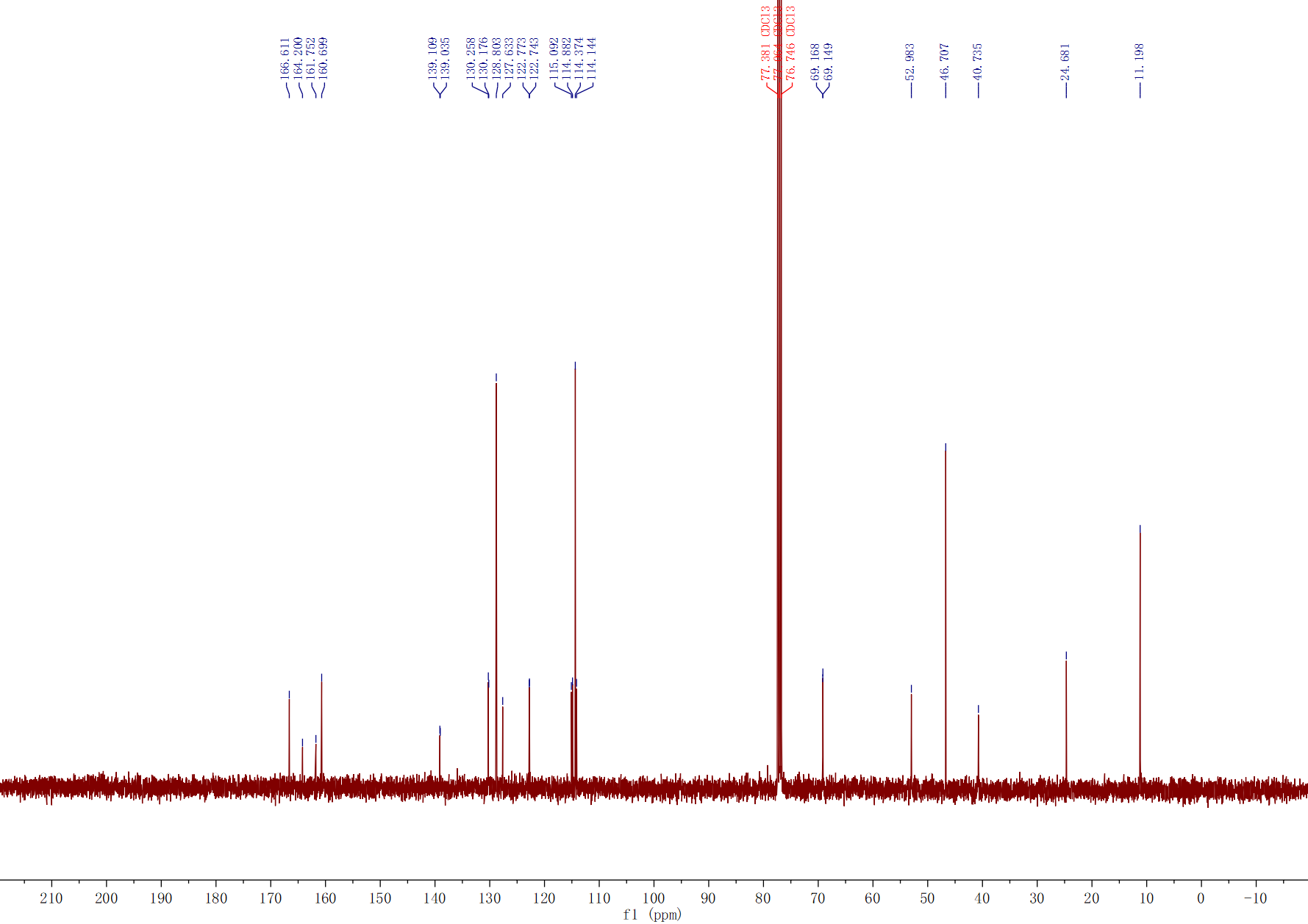


**^13^C NMR of compound 24b (100 MHz, CDCl_3_)**


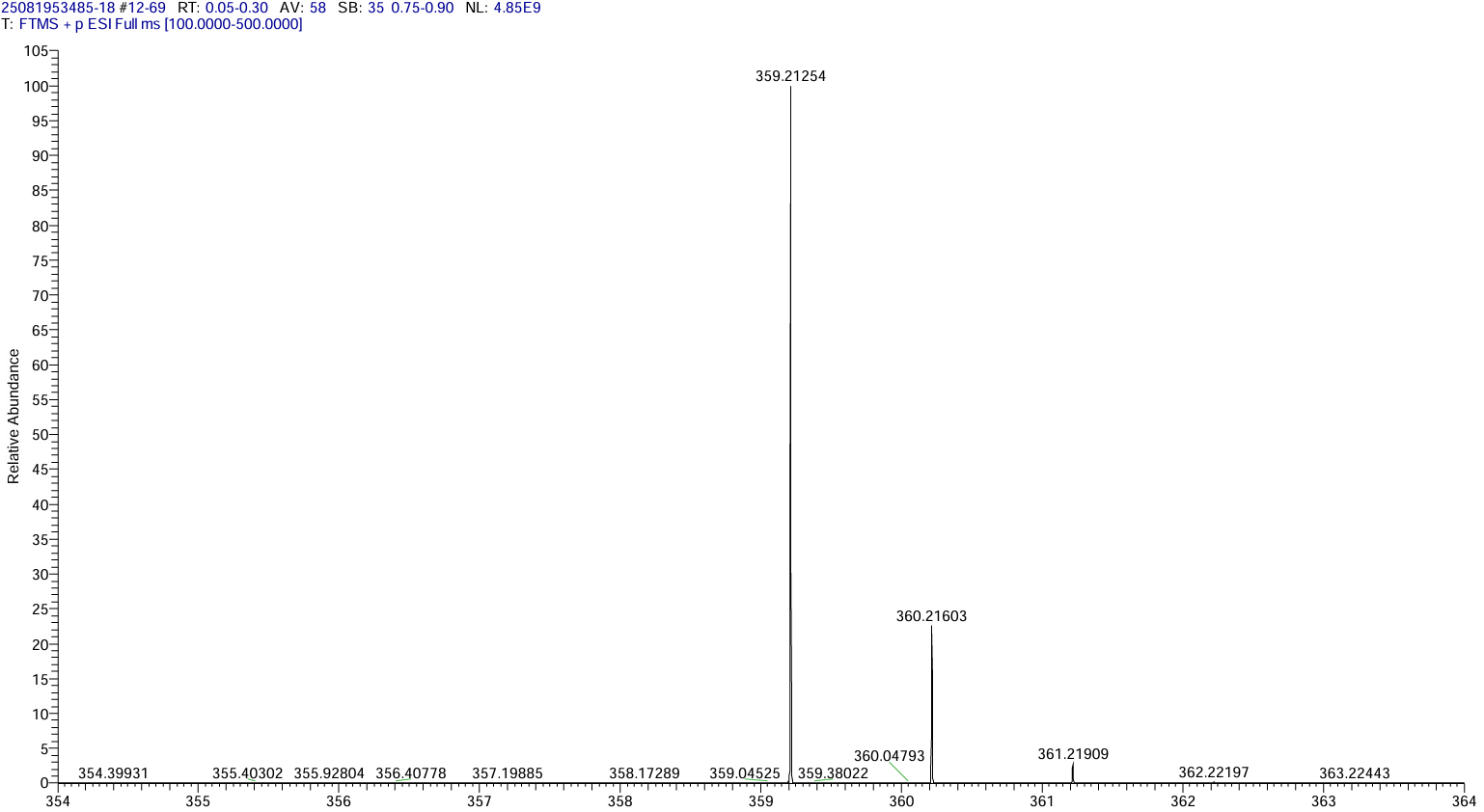


**HR-MS spectra of compound 24b**


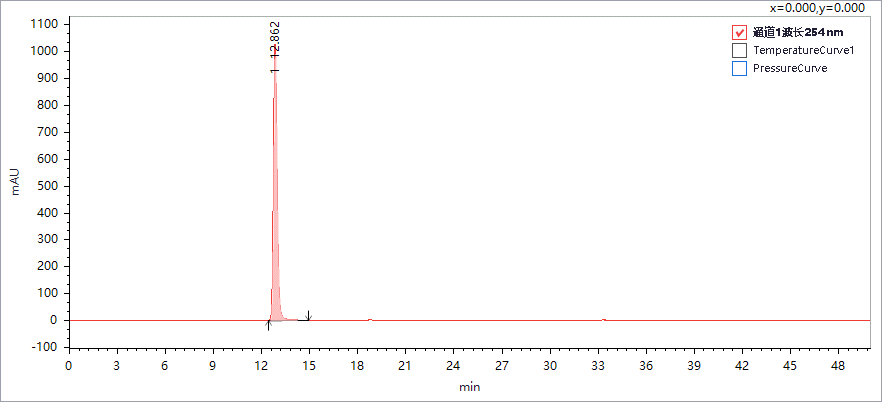


**HPLC purity of compound 24b**


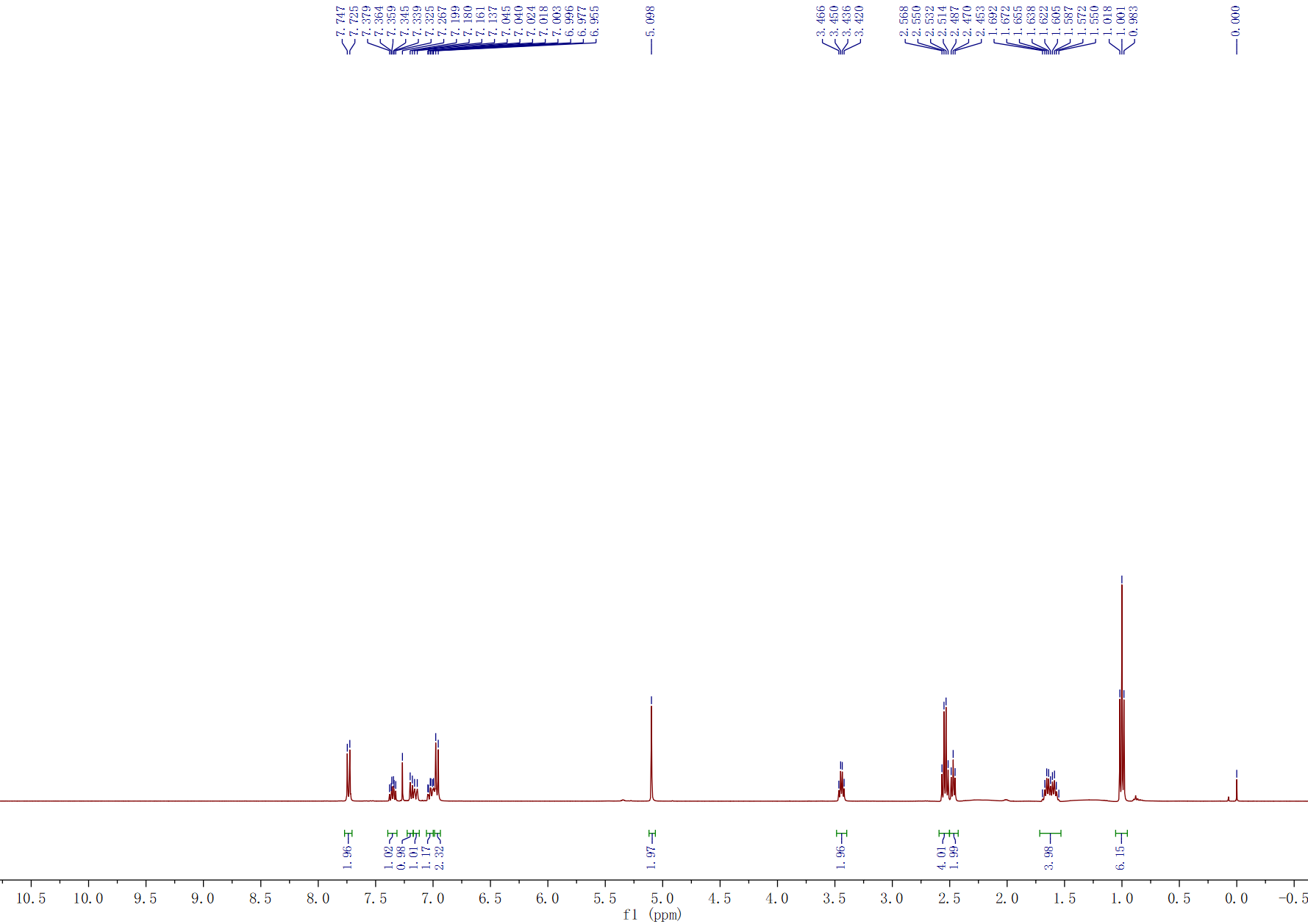


**^1^H NMR spectra of compound 25b (400 MHz, CDCl_3_)**


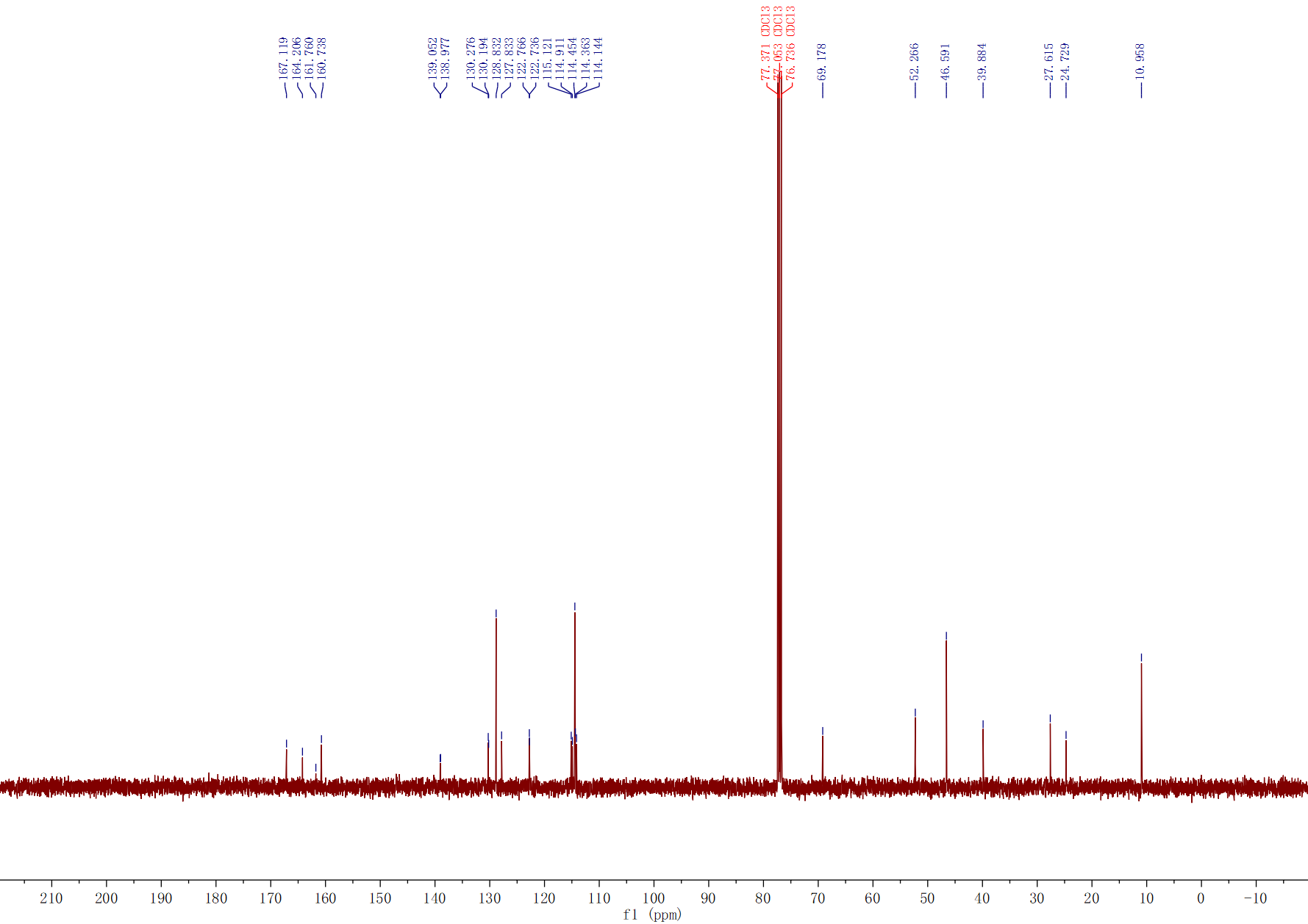


**^13^C NMR of compound 25b (100 MHz, CDCl_3_)**


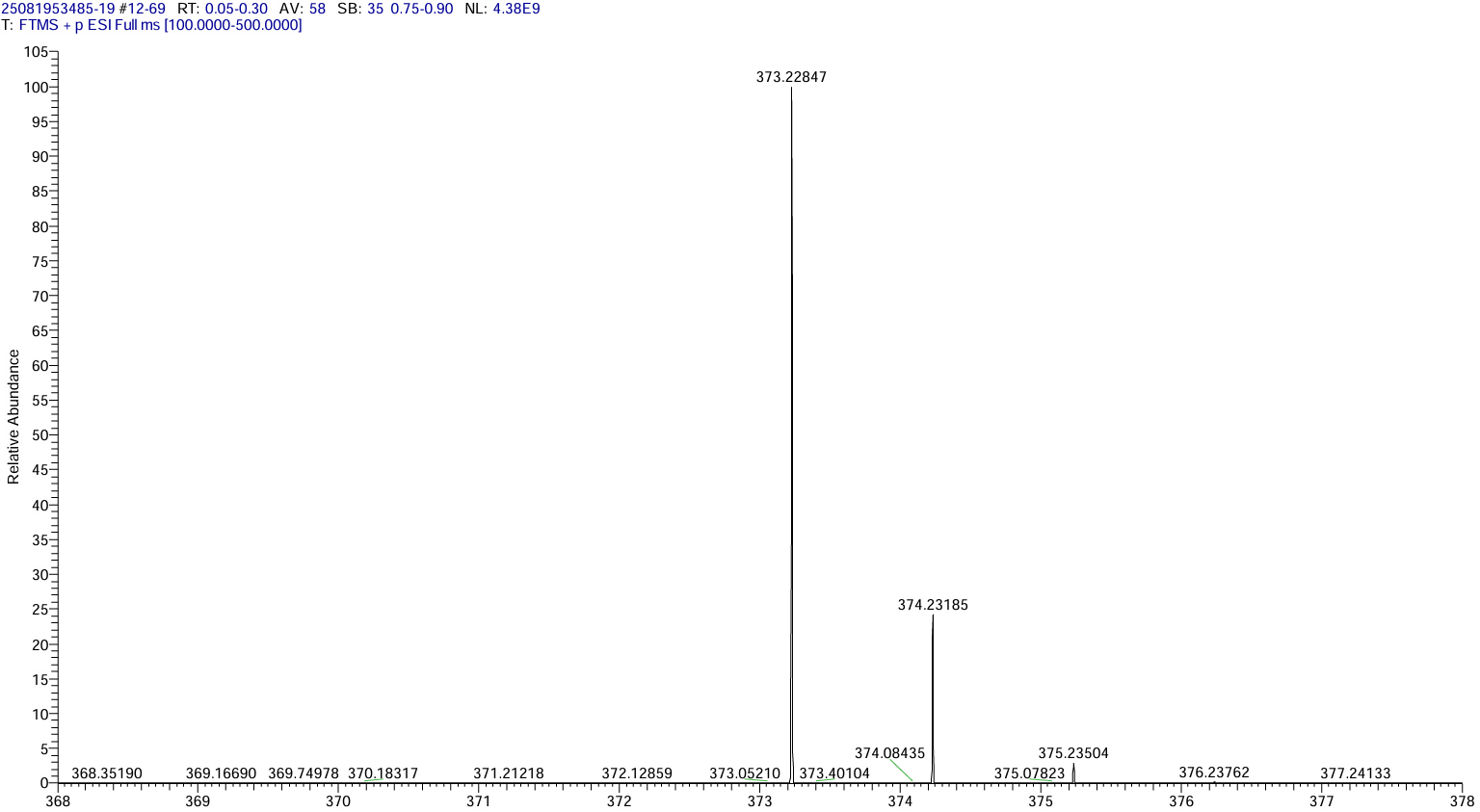


**HR-MS spectra of compound 25b**


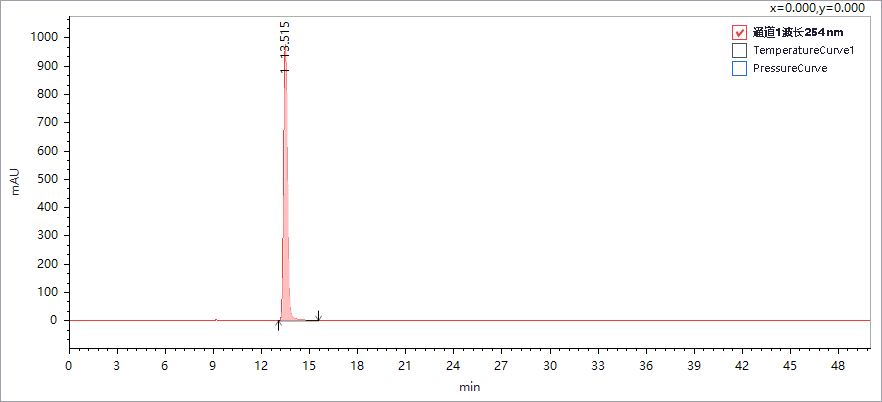


**HPLC purity of compound 25b**


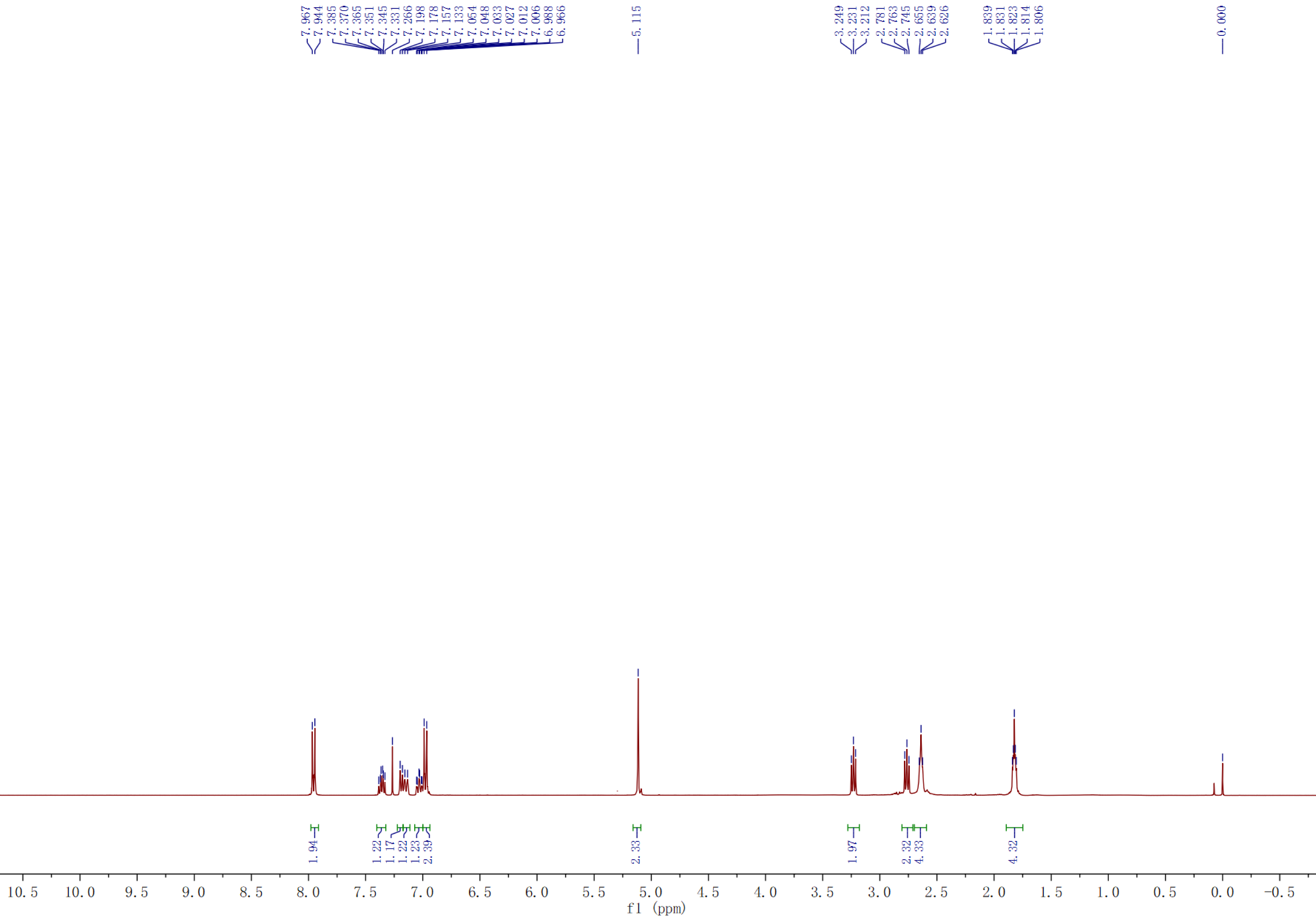


**^1^H NMR spectra of compound 28c (400 MHz, CDCl_3_)**


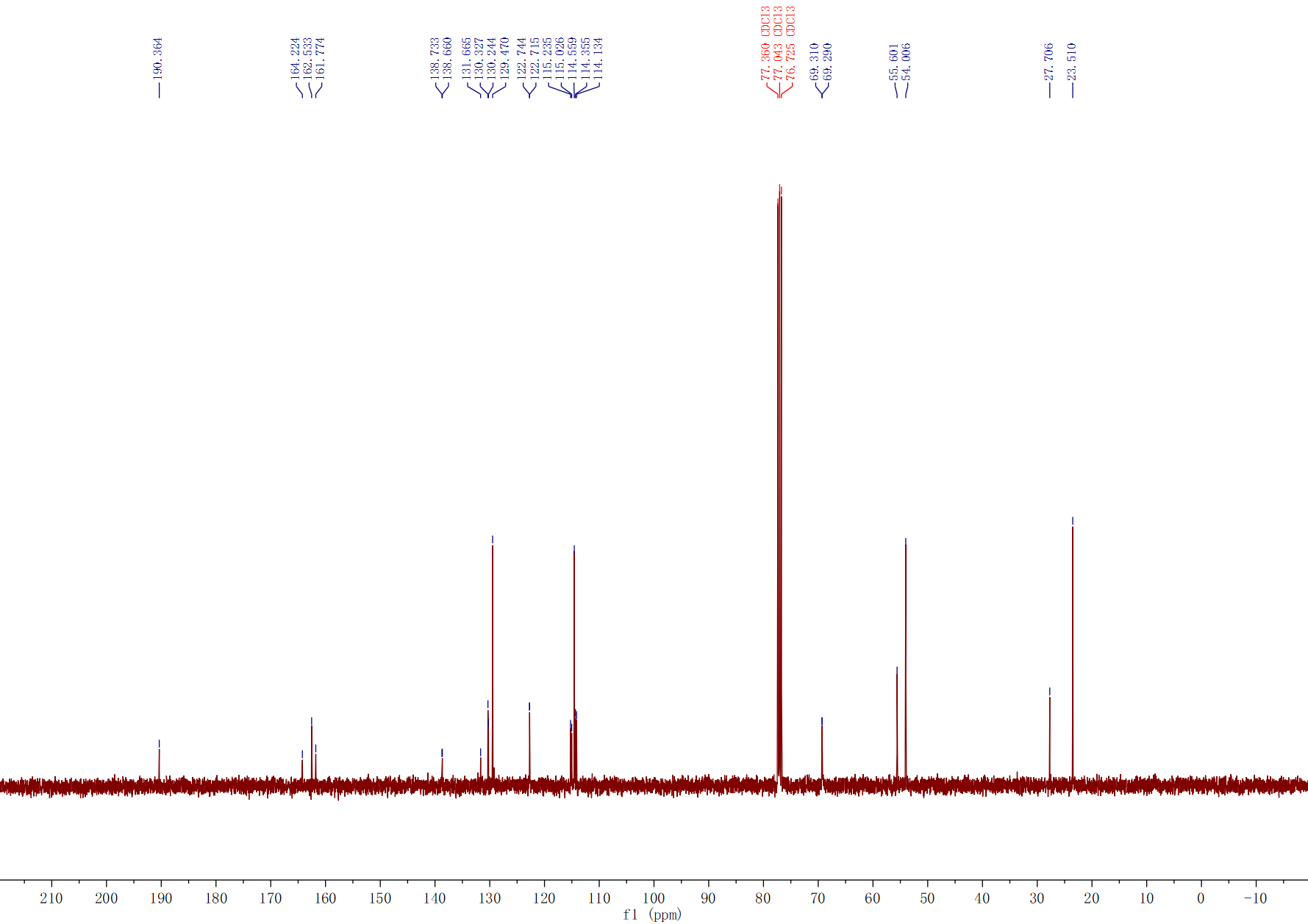


**^13^C NMR of compound 28c (100 MHz, CDCl_3_)**


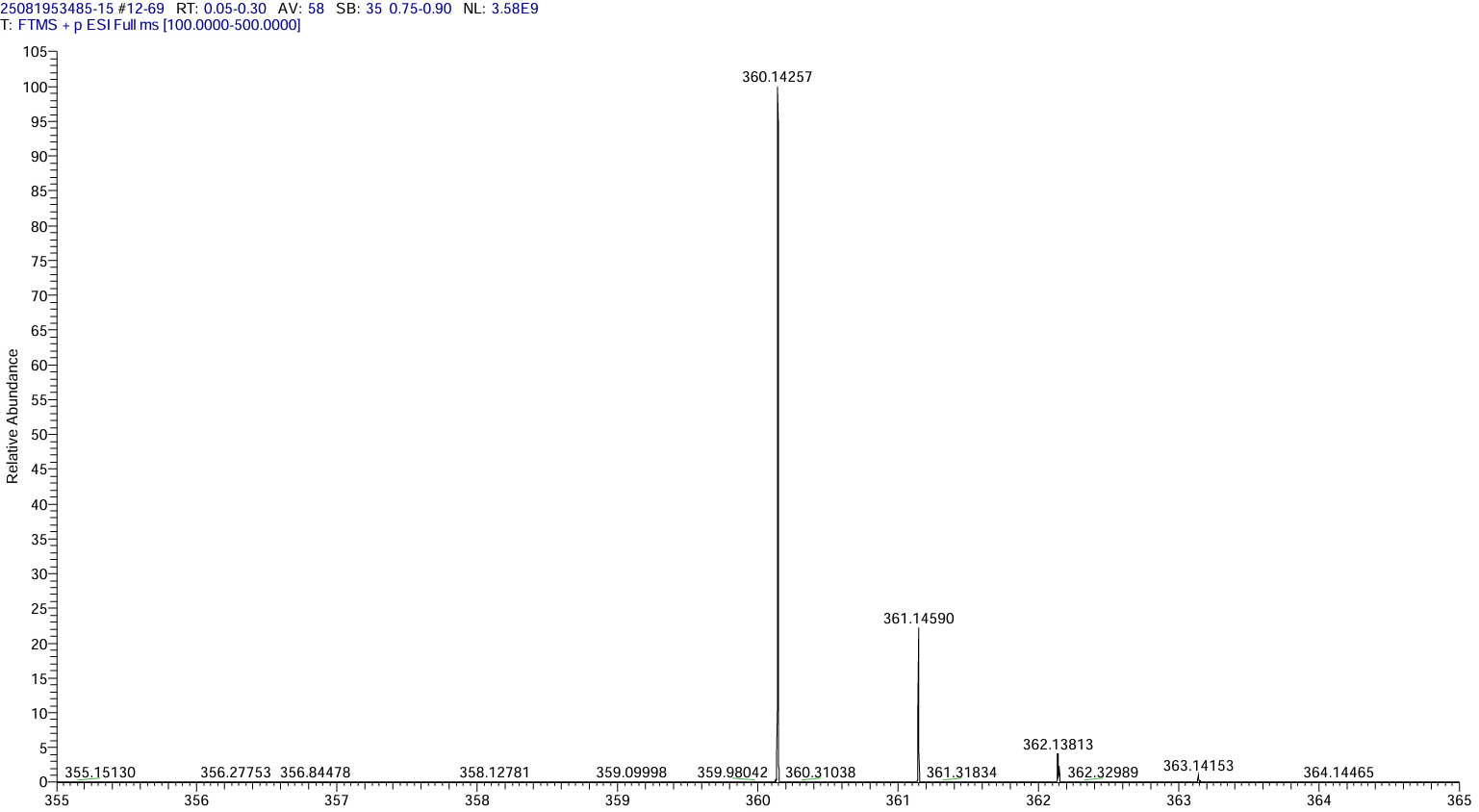


**HR-MS spectra of compound 28c**


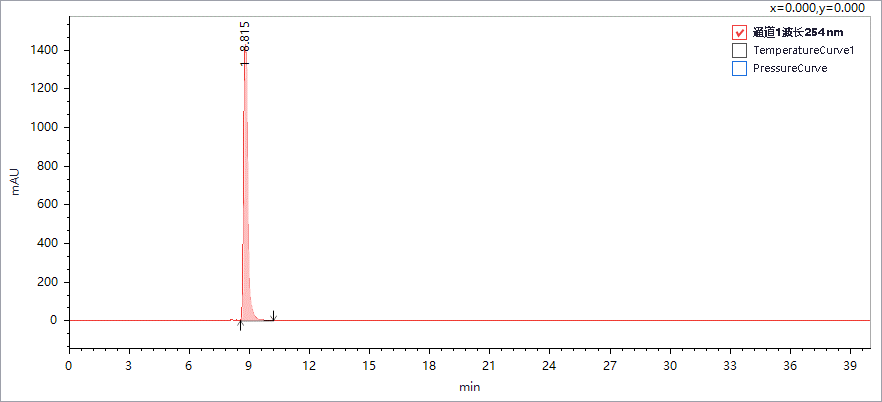


**HPLC purity of compound 28c**
